# Discovery of genetic biomarkers contributing to variation in drug response of cytidine analogues using human lymphoblastoid cell lines

**DOI:** 10.1186/1471-2164-15-93

**Published:** 2014-02-01

**Authors:** Liang Li, Brooke L Fridley, Krishna Kalari, Nifang Niu, Gregory Jenkins, Anthony Batzler, Ryan P Abo, Daniel Schaid, Liewei Wang

**Affiliations:** 1 Division of Clinical Pharmacology, Department of Molecular Pharmacology and Experimental Therapeutics, Mayo Clinic, Rochester, Minnesota, USA; 2 Division of Biomedical Statistics and Informatics, Department of Health Sciences Research, Mayo Clinic, Rochester, Minnesota, USA; 3 Current affiliation: Department of Biostatistics, University of Kansas Medical Center, Kansas City, Kansas, USA

**Keywords:** Cytidine analogues, Gemcitabine, Cytosine arabinoside, Lymphoblastoid cell lines, Expression array, Genome-wide SNPs, Genome-wide association study, Functional genomics, Translational research

## Abstract

**Background:**

Two cytidine analogues, gemcitabine and cytosine arabinoside (AraC), are widely used in the treatment of a variety of cancers with a large individual variation in response. To identify potential genetic biomarkers associated with response to these two drugs, we used a human lymphoblastoid cell line (LCL) model system with extensive genomic data, including 1.3 million SNPs and 54,000 basal expression probesets to perform genome-wide association studies (GWAS) with gemcitabine and AraC IC50 values.

**Results:**

We identified 11 and 27 SNP loci significantly associated with gemcitabine and AraC IC50 values, respectively. Eleven candidate genes were functionally validated using siRNA knockdown approach in multiple cancer cell lines. We also characterized the potential mechanisms of genes by determining their influence on the activity of 10 cancer-related signaling pathways using reporter gene assays. Most SNPs regulated gene expression in a *trans* manner, except 7 SNPs in the *PIGB* gene that were significantly associated with both the expression of *PIGB* and gemcitabine cytotoxicity.

**Conclusion:**

These results suggest that genetic variation might contribute to drug response via either *cis*- or *trans*- regulation of gene expression. GWAS analysis followed by functional pharmacogenomics studies might help identify novel biomarkers contributing to variation in response to these two drugs and enhance our understanding of underlying mechanisms of drug action.

## Background

Both gemcitabine and AraC are widely used in the treatment of a variety of cancers and both display wide individual variation in drug response [1-6]. Pharmacogenomic studies have the potential to provide insight into mechanisms underlying individual variation in response to these two drugs [7-11]. Many previous pharmacogenetic studies focused on the bioactivation and metabolism pathways for cytidine analogues [12,13]. For example, SNPs in genes encoding ribonucleotide reductase (*RRM1*) and cytidine deaminase (*CDA*) were found to be associated with gemcitabine chemosensitivity in the NCI-60 cell lines or with active gemcitabine metabolite plasma levels [14-16]. Those findings provided the initial evidence that genetic variation might contribute to variation in cytidine analogue response. We previously used the “Human Variation Panel”, a genomic data-rich lymphoblastoid cell line model system, to identify markers that might contribute to variation in response to these two cytidine analogues [17,18]. These cell lines have proven to be a powerful tool for both the identification of pharmacogenomic hypotheses and for the pursuit of hypotheses from the clinical GWAS [19-21]. However, the earlier studies were performed with less dense SNP coverage, in the present study, we expanded our previous 550 K SNP data to include a total of 1.3 million SNPs obtained with both Illumina and Affymetrix SNP genotyping platforms in an attempt to identify additional genes or SNPs that might be associated with drug response. To follow-up the candidates, we performed functional studies using tumor cell lines in an attempt to determine possible underlying mechanisms that might help us to better understand mechanisms of action for these two drugs. The results of the comprehensive series of experiments described subsequently resulted in the identification of several novel SNPs and genes associated with gemcitabine and AraC drug response in these cell lines. These results could be tested in future clinical studies to determine whether they might help predict response to gemcitabine and AraC.

## Methods

### Cell lines

One hundred and seventy four human lymphoblastoid cell lines from 60 Caucasian-American (CA), 54 African-American (AA) and 60 Han Chinese-American (HCA) (sample sets HD100CAU, HD100AA, and HD100CHI) subjects were purchased from the Coriell Cell Repository (Camden, NJ). All of these cell lines had been obtained and anonymized by the National Institute of General Medical Sciences prior to deposit, and all subjects had provided written consent for the use of their DNA and cells for experimental purposes. Human SU86 pancreatic cancer cell lines were a gift from Dr. Daniel D. Billadeau (Department of Immunology and Division of Oncology Research, Mayo Clinic College of Medicine). Human breast cancer MDA-MB-231 and leukemia BDCM and THP-1 cell lines were purchased from the American Type Culture Collection (ATCC) (Manassas, VA) and were cultured in DMEM with 1% L-glutamine (Mediatech) supplemented with 10% FBS (Mediatech). Other cell lines were maintained in RPMI medium 1640 with 1% L-glutamine (Mediatech) supplemented with 10% FBS (Mediatech).

### Drugs and cell proliferation assays

Gemcitabine was provided by Eli Lilly (Indianapolis, IN). AraC was purchased from Sigma-Aldrich (St. Louis, MO). Cytotoxicity assays with the lymphoblastoid and tumor cell lines were performed with the CellTiter 96® AQ_ueous_ Non-Radioactive Cell Proliferation Assay (Promega Corporation, Madison, WI) as previously described [17]. IC_50_ values were calculated using a three or four parameter logistic model with the R package “drc” (http://cran.r-project.org/web/packages/drc/drc.pdf), as described previously [18].

### SNP genotyping

In order to validate the imputation results, six top imputed SNPs (rs10447475, rs4621668, rs11215400, rs10926784, rs3196512 and rs7762319) were selected for genotyping using Applied Biosystems TaqMan technology (Life Technologies, Grand Island, NY). One SNP (rs7762319) was not genotyped because the assay failed functional test, and four of the remaining five SNPs were successfully genotyped. Among these four SNPs, rs11215400 was a pre-designed assay, while the remaining three SNPs (rs10447475, rs4621668 and rs10926784) were customized assays designed with Custom TaqMan® Assay Design Tool (Life Technologies, Grand Island, NY). Primer and probe sequences for these assays are available upon request. PCR protocols were followed according to the manufacturer’s guidelines for the 384-well format. PCR amplifications were performed using Applied Biosystems® TaqMan® Genotyping Master Mix with an Applied Biosystems® Veriti® 384-Well Thermal Cycler (Life Technologies, Grand Island, NY), and PCR products were analyzed on an Applied Biosystems 7900HT [22].

### Transient transfection and RNA interference

Specific siGENOME siRNA SMARTpool® reagents against a given gene, as well as a negative control, siGENOME Non-Targeting siRNA Pool #2, were purchased from Dharmacon Inc. (Lafayette, CO). Human pancreatic cancer SU86 and breast cancer MDA-MB-231 cell lines were used to perform the siRNA knockdown studies. The lipofectamine RNAiMAX transfection reagent (Invitrogen, Carlsbad, CA) was used for siRNA reverse or forward transfection. Specifically, cells were seeded into 96-well plates and were mixed with siRNA-complex consisting of 20–50 nM of specific siGENOME siRNA SMARTpool or non-targeting negative control (Dharmacon) and the lipofectamine RNAiMAX transfection reagent. The human leukemia cell lines, BDCM and THP-1, were transfected with electroporation using the Nucleofector System with 500 nM of specific or negative siRNA (Lonza Inc., Basel, Switzerland).

### Quantitative reverse transcription-PCR (QRT-PCR)

Total RNA was isolated from cultured cells with the Qiagen RNeasy kit (QIAGEN Inc. Valencia, CA), followed by QRT-PCR performed with the 1-step, Brilliant SYBR Green QRT-PCR master mix kit (Stratagene, La Jolla, CA). Specifically, primers purchased from Qiagen were used to perform QRT-PCR with the Stratagene Mx3005P™ Real-Time PCR detection system (Stratagene). All experiments were performed in triplicate with β-actin as an internal control. Reverse transcribed Universal Human reference RNA (Stratagene) was used to generate a standard curve. Control reactions lacked RNA template.

### Caspase-3/7 activity assay

Caspase-3/7 activity was measured with the Caspase-Glo®3/7 Assay kit (Promega). Specifically, siRNA-transfected cells (100 μl) were seeded overnight into 96-well plates at a density of 10,000 cells per well and were then treated with DMSO or increasing concentrations of gemcitabine or AraC for 48 h. 100 μL of Caspase-Glo® 3/7 Reagent was then added to each well, and the cells were incubated at room temperature for 1 h, followed by the measurement of luminescence with a Safire^2^ microplate reader (Tecan Trading AG, Switzerland). The luminescent signal was proportional to caspase-3/7 activity and was used as a measure of apoptosis. Wells containing only culture medium were used as controls.

### Cancer cignal finder 10-pathway reporter array

The Cignal Finder Arrays consist of 10 dual-luciferase reporter assays for ten cancer-related signaling pathways. Each reporter construct is a mixture of an inducible transcription factor (TF) responsive firefly luciferase reporter and a constitutively expressing Renilla construct at a ratio of 40:1, respectively (SABioscience Co., Frederick, MD). Specifically, cells were reversely transfected with 30 nM of specific siRNA pools in 96-well plates using Lipofectamine RNAiMAX reagent (Invitrogen) for 24 h, followed by transfection with 100 ng of each reporter construct. Forty-eight h after the transfection, a dual-luciferase assay was performed with the Dual-Luciferase Reporter Assay System (Promega) in a Safire^2^ microplate reader (Tecan).

### Electrophoresis mobility shift assays (EMSA)

Based on the genome-wide association results, we performed EMSA for SNPs in potential regulatory regions of genes that were associated with the measured phenotypes. Specifically, double-strand probes were 5′-end labeled with biotin and electrophoresis was performed with 5% acrylamide gels, followed by autoradiography. Competition experiments were performed with excess non-labeled probe.

### Genome-wide gene expression and SNP analysis

Expression array data were obtained for all 174 lymphoblastoid cell lines (LCLs) as previously described [17]. Illumina HumanHap550K and 510S BeadChips, which assayed 561,298 and 493,750 SNPs, respectively, were used to obtain genome-wide SNP data for these LCLs [23]. Genotyping was performed in the Genotype Shared Resource (GSR) at the Mayo Clinic, Rochester, MN. We also obtained publicly available Affymetrix SNP Array 6.0 Chip SNP data which involved 643,600 SNPs unique to the Affymetrix SNP array for the same cell lines. After quality control (QC), SNPs with call rates <0.95, Hardy-Weinberg Equilibrium (HWE) P values < 0.001, or MAFs <5% were excluded, as were DNA samples with call rates <0.95. A total of 1,348,798 SNPs that passed QC were used to perform the association studies.

### Imputation analysis

SNPs not genotyped were imputed across a region 200 kb up or downstream of the selected genes harboring or close to the SNPs associated with drug response in the LCLs. Imputation was performed using Beagle (v3.3.1) [24] with the 11/23/2010 release of the 1000 Genomes project as a reference population [25]. Imputed SNPs with a dosage R^2^ quality measure of less than 0.3, and SNPs with MAF <0.01 were not included in the analysis. Four of the imputed SNPs were genotyped for validation, the average squared difference between the count of the same allele in the imputed and genotyped version of these SNPs was computed to measure the concordance of the imputed genotype with actual genotype, a smaller difference indicating greater concordance.

### Statistical methods

Partial Pearson correlations were used to quantify the association between: SNPs and mRNA expression; SNPs and IC_50_; and mRNA expression and IC_50_. IC_50_ was transformed to remove skewness using a log transformation for gemcitabine and van der Waerden rank transformation for AraC. The adjustment variables in the partial correlation were race and gender if SNPs were not involved; or race, gender and five eigenvectors controlling for population stratification as described previously [23]. These partial correlations were tested using a Wald test, false discovery q-values [26] were also computed for each test.

## Results

### Genome-wide SNP vs. drug cytotoxicity association study and imputation analysis

**Figure 1:**
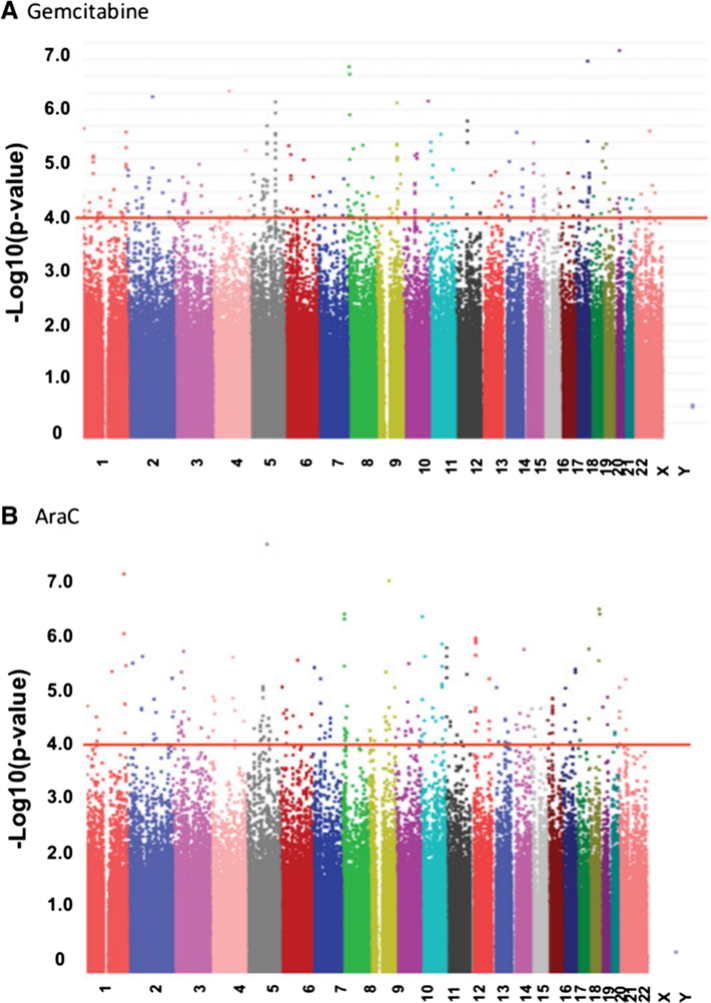
**GWAS findings. (A)**. Manhattan plot of 1.3 million SNPs with gemcitabine IC50 values. **(B)**. Manhattan plot of 1.3 million SNPs with gemcitabine IC50 values. SNPs are plotted on the x-axis based on their chromosomal locations. P values of 10^-4^ are highlighted with a red line.

Previously, we had performed GWAS using only the 550 k SNP data set for this cell line system [18]. In the current study, we expand the SNPs studies to include additional Illumina SNPs as well as publically available SNP data obtained with Affymetrix 6.0 SNP data for the same cell lines to identify additional novel potential pharmacogenomic biomarkers. As a result, we performed an analysis for the association of 1,348,798 SNPs with IC50 values for gemcitabine and AraC (Figure 1A and 1B). The most significant SNP for gemcitabine was rs1598848 with a P value 7.08 × 10^-7^ (r = −0.391, MAF = 0.473), while the most significant SNP for AraC was rs4078252 with a P value 1.54 × 10^-7^ (r = 0.405, MAF = 0.198) (Additional file 1: Table S1A and B). Fourteen SNPs for gemcitabine and 34 for AraC had P values <10^-5^, and 143 SNPs for gemcitabine and 204 SNPs for AraC had P values <10^-4^, respectively. One hundred and twenty six SNPs with P < 10^-3^ were common to both drugs (Additional file 1: Table S1C). To explore ungenotyped SNPs that might be functional, we imputed SNPs surrounding the selected genes (+/−200 kb) harboring or close to the most significant SNP loci using 1000 Genomes data as a reference (Additional file 1: Figure S1A, B, and C). Besides the “observed SNPs” on the genotyping platforms, there were 23 imputed SNPs for gemcitabine and 35 for AraC, respectively, that were also associated with drug response IC50 values (P < 10^-4^) (Additional file 1: Table S2A and B). In order to determine the accuracy of imputation, we selected 6 imputed SNPs (rs10447475, rs4621668, rs11215400, rs10926784, rs3196512 and rs7762319) that were among the top two SNPs from each gene region associated with drug response IC50s (P < 10^-3^) to genotype using Taqman assay. Four SNPs ((rs10447475, rs4621668, rs11215400 and rs10926784) were successfully genotyped. The average squared difference between the count of the same allele in the imputed and genotyped version of these 4 SNPs was low ranging from 0.02-0.065 indicating that the concordance was high (Additional file 1: Figure S2).

### “Integrated analyses” of SNP loci, basal expression and drug cytotoxicity

We also performed integrated analyses of SNPs, expression array and cytotoxicity data [18,23]. To do that, we began with SNPs that had P values <10^-3^. We selected a less stringent P value cutoff to include as many potential candidates as possible for follow-up functional genomic studies. Next, we tested expression probe sets that were associated with these SNPs, followed by association of those expression probe sets with drug IC50 values, ie we performed an “integrated analysis”. In these analyses, we used SNP loci, defined as a region that contained at least 2 SNPs with P < 10^-4^ or 1 SNP with P < 10^-4^, plus 3 additional SNPs with P < 10^-3^ within +/−100 kb surrounding the most significant SNPs. All of the SNPs within each of those loci are listed in Additional file 1: Table S2, which includes genotyped as well as imputed SNPs. We identified 11 loci containing 166 SNPs for gemcitabine and 23 loci with 187 SNPs for AraC that were associated with IC50 values for these two drugs, respectively (Table 1). Four loci containing 4 genes – *HLA-DRA, ZNF215, MASS1*, and *PLD5* – were common to both drugs (Table 1).

**Table 1:** The top 11 loci for Gemcitabine (A) and the top 27 loci for AraC (B) that were associated with drug response-IC50 values

** Top SNP rsID**	** Lowest R**	** Lowest P**	** Nearby gene**	** Chr**	** Position**	** MAF**	**Region**	**Number of SNPs in each locus**
**(A) Gemcitabine**								
rs10513968	−0.382	1.04E-06	*DOK6*	18	65434121	0.445	Intron	9
rs7719624	−0.370	4.22E-06	*TGFBI*	5	135405465	0.448	Intron	28
rs2274870	0.362	4.33E-06	*NIPSNAP3A*	9	106555035	0.371	Coding	7
rs316838	−0.345	1.21E-05	PLD5*	1	240387283	0.093	Intron	12
rs2638094	−0.339	1.72E-05	ZNF215*	11	6938064	0.679	Flanking_3UTR	5
rs2290344	0.339	1.75E-05	*PIGB*	15	53407088	0.253	Coding	9
rs3129890	0.332	2.67E-05	HLA-DRA*	6	32522251	0.340	Flanking_3UTR	23
rs2928445	−0.331	2.76E-05	*IPMK*	10	58816782	0.267	Flanking_3UTR	23
rs4392391	−0.318	5.78E-05	*C3orf23*	3	44247970	0.419	Downstream	15
rs12522395	0.318	6.58E-05	MASS1*	5	90246274	0.215	Intron	4
rs13171512	−0.313	7.857E-05	*PIK3R1*	5	68000787	0.462	Flanking_3UTR	4
**(B) AraC**								
rs10495285	0.393	4.13E-07	*KIAA0133 (URB2)*	1	227871618	0.171	Downstream	2
rs1450679	0.389	5.20E-07	*SMC2L1*	9	106066633	0.098	Flanking_3UTR	8
rs6128386	0.375	1.38E-06	*LOC149773*	20	56626955	0.466	Upstream	4
rs2172820	−0.373	1.63E-06	*TUSC3*	8	15370039	0.256	Flanking_5UTR	13
rs2857891	−0.372	1.75E-06	ZNF215*	11	6919533	0.101	Intron	6
rs9512755	−0.361	3.67E-06	*LNX2*	13	27056229	0.055	Flanking_5UTR	4
rs888468	−0.356	5.08E-06	*FGF6*	12	4403640	0.184	Flanking_3UTR	5
rs2653165	−0.347	9.28E-06	PLD5*	1	240432905	0.207	Intron	7
rs11215416	−0.346	9.57E-06	*IGSF4*	11	114582537	0.147	Intron	7
rs10060641	0.335	1.90E-05	MASS1*	5	90249006	0.216	Intron	7
rs5989586	0.336	1.94E-05	*HDHD1A*	23	6757306	0.457	Flanking_3UTR	8
rs216465	−0.330	2.76E-05	*GOSR1*	17	25880801	0.494	Flanking_3UTR	12
rs856548	−0.326	3.29E-05	*TNS3*	7	46730993	0.296	Flanking_3UTR	8
rs6689258	0.325	3.40E-05	*GPR137B*	1	234362702	0.267	Flanking_5UTR	2
rs3794794	−0.325	3.61E-05	*CPD*	17	25744867	0.477	Intron	10
rs7192	0.322	4.10E-05	HLA-DRA*	6	32519624	0.371	Coding	11
rs4572738	0.322	4.45E-05	*CAST1*	3	55799452	0.321	Intron	8
rs1433446	0.319	5.05E-05	*TMEM83*	15	85351874	0.109	Flanking_5UTR	5
rs300962	0.320	5.47E-05	*LOC51334*	5	119909842	0.269	Intron	15
rs6441911	0.315	6.20E-05	*RIS1*	3	45322523	0.055	Flanking_5UTR	6
rs1159388	0.314	6.61E-05	*DCAMKL1*	13	35544706	0.172	Intron	11
rs718979	0.313	6.82E-05	*UBE3A*	15	23325370	0.394	Flanking_5UTR	6
rs1955412	0.309	8.74E-05	*FLRT2*	14	85082547	0.21	Intron	11

Each locus contained at least 2 SNPs within 50 kb with P values <10^-4^. R values represent correlation coefficients for the association. Number of SNPs indicates the number of significantly associated SNPs in the locus. rsID indicates the most significant SNP associated with drug cytotoxicity in the locus. * = common to both gemcitabine and AraC.

The integrated analyses identified 66 SNPs in 6 loci that were associated with gemcitabine IC50 values and the expression of 12 genes represented by 17 probesets. Those 17 probesets were also associated with gemcitabine IC50 values (P < 10^-4^). The integrated analyses also identified 36 SNPs in 3 loci that were associated with AraC IC50 values and the expression of 9 genes (10 probesets) (Table 2). For gemcitabine, 19 SNPs were within *cis*-regulatory regions for *PIGB* or *C3orf23*. No *cis*- regulation between SNP and gene expression was identified for AraC. Of interest, SNPs in *PIGB* were associated with the expression of that gene (lowest P = 5.97 × 10^-9^) as well as the expression of *FKBP5* (lowest P = 2.70 × 10^-6^), a gene that we previously reported to play an important role in response to gemcitabine and AraC as well as many other chemotherapeutic agents including gemcitabine and AraC [17,27]. We next moved to further analyses of candidate genes identified during the integrated analysis.

**Table 2:** Integrated analyses for drug response for either (A) Gemcitabine or (B) AraC

**SNP**						**Probe set**			**GWAS**					
**rslD**	**Closest gene**	**MAF**	**Chr**	**Position**	**Region**	**Probeset**	**Gene symbol**	**Chr**	**R value (SNP vs IC50)**	**P value (SNP vs IC50)**	**R value (SNP vs Exp)**	**P value (SNP vs Exp)**	**R value (EXP vs IC50)**	**P value (EXP vs IC50)**
**(A) Gemcitabine**														
rs316871	*PLD5*	0.097	1	240409507	Intron	225532_at	*CABLES1*	18	-0.326	4.14E–05	-0.242	6.54E–05	0.347	3.12E–06
rs316823	*PLD5*	0.093	1	240422651	Intron	225532_at	*CABLES1*	18	-0.288	2.88E–04	-0.257	2.05E–05	0.347	3.12E–06
rs402098	*PLD5*	0.093	1	240430321	Intron	225532_at	*CABLES1*	18	-0.288	2.88E–04	-0.257	2.05E–05	0.347	3.12E–06
rs427498	*PLD5*	0.087	1	240424078	Intron	225532_at	*CABLES1*	18	-0.280	4.43E–04	-0.256	2.28E–05	0.347	3.12E–06
rs316823	*PLD5*	0.093	1	240422651	Intron	225531_at	*CABLES1*	18	-0.288	2.88E–04	-0.239	7.69E–05	0.321	1.72E–05
rs402098	*PLD5*	0.093	1	240430321	Intron	225531_at	*CABLES1*	18	-0.288	2.88E–04	-0.239	7.69E–05	0.321	1.72E-05
rs4392391	*C3orf23*	0.419	3	44247970	Downstream	1555906_s_at	*C3orf23*	3	-0.318	5.78E–05	-0.250	3.46E–05	0.263	4.86E–04
rs1565214	*C3orf23*	0.425	3	44309303	Upstream	1555906_s_at	*C3orf23*	3	-0.308	1.35E–04	-0.251	4.09E–05	0.263	4.86E–04
rs9809107	*C3orf23*	0.419	3	44257938	Flanking_5UTR	1555906_s_at	*C3orf23*	3	-0.301	1.46E–04	-0.242	5.97E–05	0.263	4.86E–04
rs967285	*C3orf23*	0.422	3	44250280	Downstream	1555906_s_at	*C3orf23*	3	-0.301	1.48E–04	-0.247	4.36E–05	0.263	4.86E–04
rs9821268	*C3orf23*	0.413	3	44278128	Upstream	1555906_s_at	*C3orf23*	3	-0.292	2.43E–04	-0.241	6.29E–05	0.263	4.86E–04
rs1565215	*C3orf23*	0.418	3	44309103	Upstream	1555906_s_at	*C3orf23*	3	-0.291	2.64E–04	-0.246	4.62E–05	0.263	4.86E–04
rs10510741	*C3orf23*	0.445	3	44239198	Flanking_5UTR	1555906_s_at	*C3orf23*	3	-0.283	3.68E–04	-0.275	4.71E–06	0.263	4.86E–04
rs9852733	*C3orf23*	0.451	3	44252343	Flanking_5UTR	1555906_s_at	*C3orf23*	3	-0.279	4.59E–04	-0.268	8.53E–06	0.263	4.86E–04
rs6808448	*C3orf23*	0.451	3	44218611	Flanking_5UTR	1555906_s_at	*C3orf23*	3	-0.273	6.19E–04	-0.273	5.38E–06	0.263	4.86E–04
rs9846155	*C3orf23*	0.456	3	44257844	Flanking_5UTR	1555906_s_at	*C3orf23*	3	-0.272	6.33E–04	-0.262	1.38E–05	0.263	4.86E–04
rs9284879	*C3orf23*	0.433	3	44259588	Flanking_5UTR	1555906_s_at	*C3orf23*	3	-0.264	9.43E–04	-0.253	2.65E–05	0.263	4.86E–04
rs12486452	*C3orf23*	0.433	3	44299569	Flanking_5UTR	1555906_s_at	*C3orf23*	3	-0.264	9.43E–04	-0.241	6.45E–05	0.263	4.86E–04
rs12631341	*C3orf23*	0.433	3	44284272	Flanking_5UTR	1555906_s_at	*C3orf23*	3	-0.264	9.43E–04	-0.241	6.45E–05	0.263	4.86E–04
rs7631790	*C3orf23*	0.433	3	44274209	Flanking_5UTR	1555906_s_at	*C3orf23*	3	-0.264	9.43E–04	-0.241	6.45E–05	0.263	4.86E–04
rs4392391	*C3orf23*	0.419	3	44247970	Downstream	227978_s_at	*ZADH2*	18	-0.318	5.78E–05	0.251	3.11E–05	-0.351	2.42E–06
rs1565214	*C3orf23*	0.425	3	44309303	Upstream	227978_s_at	*ZADH2*	18	–0.308	1.35E–04	0.246	5.9E–05	-0.351	2.42E–06
rs9809107	*C3orf23*	0.419	3	44257938	Flanking_5UTR	227978_s_at	*ZADH2*	18	-0.301	1.46E–04	0.273	5.72E–06	-0.351	2.42E–06
rs967285	*C3orf23*	0.422	3	44250280	Downstream	227978_s_at	*ZADH2*	18	-0.301	1.48E–04	0.254	2.58E–05	-0.351	2.42E–06
rs9821268	*C3orf23*	0.413	3	44278128	Upstream	227978_s_at	*ZADH2*	18	-0.292	2.43E–04	0.247	4.17E–05	-0.351	2.42E–06
rs1565215	*C3orf23*	0.418	3	44309103	Upstream	227978_s_at	*ZADH2*	18	-0.291	2.64E–04	0.250	3.46E–05	-0.351	2.42E–06
rs4682949	*C3orf23*	0.398	3	44220159	Flanking_5UTR	227978_s_at	*ZADH2*	18	-0.287	3.01E–04	0.247	4.14E–05	–0.351	2.42E–06
rs10510741	*C3orf23*	0.445	3	44239198	Flanking_5UTR	227978_s_at	*ZADH2*	18	-0.283	3.68E–04	0.251	3.03E–05	–0.351	2.42E–06
rs9852733	*C3orf23*	0.451	3	44252343	Flanking_5UTR	227978_s_at	*ZADH2*	18	–0.279	4.59E–04	0.239	7.38E–05	–0.351	2.42E–06
rs6808448	*C3orf23*	0.451	3	44218611	Flanking_5UTR	227978_s_at	*ZADH2*	18	-0.273	6.19E–04	0.260	1.62E–05	-0.351	2.42E–06
rs9846155	*C3orf23*	0.456	3	44257844	Flanking_5UTR	227978_s_at	*ZADH2*	18	-0.272	6.33E–04	0.260	1.58E–05	-0.351	2.42E–06
rs12486452	*C3orf23*	0.433	3	44299569	Flanking_5UTR	227978_s_at	*ZADH2*	18	-0.264	9.43E–04	0.239	7.52E–05	-0.351	2.42E–06
rs12631341	*C3orf23*	0.433	3	44284272	Flanking_5UTR	227978_s_at	*ZADH2*	18	-0.264	9.43E–04	0.239	7.52E–05	-0.351	2.42E–06
rs7631790	*C3orf23*	0.433	3	44274209	Flanking_5UTR	227978_s_at	*ZADH2*	18	-0.264	9.43E–04	0.239	7.52E–05	-0.351	2.42E–06
rs12188464	*PIK3RI*	0.459	5	67999705	Flanking_5UTR	225935_at	*–––*	7	-0.309	9.69E–05	0.366	2.47E–06	-0.296	7.99E–05
rs13171512	*PIK3RI*	0.462	5	68000787	Flanking_5UTR	236170_x_at	*–––*	7	-0.313	7.86E–05	0.349	7.69E–06	-0.299	6.75E–05
rs13171512	*PIK3RI*	0.462	5	68000787	Flanking_5UTR	225935_at	*–––*	7	-0.313	7.86E–05	0.375	1.33E–06	-0.296	7.99E–05
rs7713001	*PIK3RI*	0.459	5	67999371	Flanking_5UTR	225935_at	*–––*	7	-0.309	9.69E–05	0.366	2.47E–06	-0.296	7.99E–05
rs12188464	*PIK3RI*	0459	5	67999705	Flanking_5UTR	201334_s_at	*ARHGEF12*	11	-0.309	9.69E–05	0.380	9.03E–07	-0.339	5.35E–06
rs13171512	*PIK3RI*	0.462	5	68000787	Flanking_5UTR	201334_s_at	*ARHGEF12*	11	-0.313	7.86E–05	0.372	1.59E–06	-0.339	5.35E–06
rs7713001	*PIK3RI*	0.459	5	67999371	Flanking_5UTR	201334_s_at	*ARHGEF12*	11	-0.309	9.69E–05	0.380	9.03E–07	-0.339	5.35E–06
rs12188464	*PIK3RI*	0.459	5	67999705	Flanking_3UTR	238012_at	*DPP7*	9	-0.309	9.69E–05	0.372	1.63E–06	-0.324	1.45E–05
rs13171512	*PIK3RI*	0.462	5	68000787	Flanking_3UTR	238012_at	*DPP7*	9	-0.313	7.86E–05	0.378	1.08E–06	-0.324	1.45E–05
rs7713001	*PIK3RI*	0.459	5	67999371	Flanking_3UTR	238012_at	*DPP7*	9	-0.309	9.69E–05	0.372	1.63E–06	-0.324	1.45E–05
rs12188464	*PIK3RI*	0.459	5	67999705	Flanking_3UTR	225086_at	*FAM98B*	15	-0.309	9.69E–05	0.345	9.5E–06	-0.352	2.18E–06
rs13171512	*PIK3RI*	0.462	5	68000787	Flanking_3UTR	225086_at	*FAM98B*	15	-0.313	7.86E–05	0.354	5.38E–06	-0.352	2.18E–06
rs3135351	*HLA–DRA*	0.137	6	32500923	Flanking_5UTR	1566082_at	*–––*	10	0.265	9.48E–04	0.277	4.24E–06	0.320	1.88E–05
rs2472476	*NIPSNAP3B*	0.389	9	106571777	Intron	200988_s_at	*PSME3*	17	0.338	1.92E–05	-0.239	7.6E–05	-0.350	.60E–06
rs2928445	*IPMK*	0.267	10	58816782	Flanking_3UTR	227482_at	*ADCK1*	14	-0.331	2.76E–05	0.247	4.09E–05	-0.293	9.45E–05
rs12244977	*IPMK*	0.195	10	58762688	Flanking_3UTR	1569396_at	*RAB40C*	16	-0.308	1.03E–04	-0.243	5.72E–05	0.303	5.47E–05
rs12256364	*IPMK*	0.195	10	58765694	Flanking_3UTR	1569396_at	*RAB40C*	16	-0.308	1.03E–04	-0.243	5.72E–05	0.303	5.47E–05
rs2928445	*IPMK*	0.267	10	58816782	Flanking_3UTR	226987_at	*RBM15B*	3	-0.331	2.76E–05	0.263	1.2E–05	-0.354	1.90E–06
rs4774760	*PIGB*	0.416	15	53376504	upstreaintronm	224856_at	*FKBP5*	6	0.326	3.63E–05	-0.241	6.7E–05	-0.411	2.15E–08
rs7174876	*PIGB*	0.416	15	53406853	Upstream	224856_at	*FKBP5*	6	0.284	4.06E–04	-0.237	9.71E–05	-0.411	2.15E–08
rs2290344	*PIGB*	0.253	15	53407088	Coding	224856_at	*FKBP5*	6	0.339	1.75E–05	-0.266	9.87E–06	-0.393	9.66E–08
rs4774760	*PIGB*	0.416	15	53376504	Upstream	204560_at	*FKBP5*	6	0.326	3.63E–05	-0.282	2.7E–06	-0.393	9.66E–08
rs12050587	*PIGB*	0.45	15	53414820	Intron	224856_at	*FKBP5*	6	0.305	1.23E–04	-0.256	2.27E–05	-0.393	9.66E–08
rs28668016	*PIGB*	0.365	15	53398725	5UTR	204560_at	*FKBP5*	6	0.304	1.31E–04	-0.261	1.49E–05	-0.393	9.66E–08
rs11636687	*PIGB*	0.421	15	53392444	Flanking_5UTR	224856_at	*FKBP5*	6	0.302	1.53E–04	-0.252	3.34E–05	-0.393	9.66E–08
rs2414409	*PIGB*	0.448	15	53419009	Intron	204560_at	*FKBP5*	6	0.299	1.62E–04	-0.264	1.17E–05	-0.393	9.66E–08
rs7183960	*PIGB*	0.451	15	53409987	Intron	224856_at	*FKBP5*	6	0.292	2.37E–04	-0.262	1.37E–05	-0.393	9.66E–08
rs7174876	*PIGB*	0.423	15	53406853	Intron	204560_at	*FKBP5*	6	0.284	4.06E–04	-0.274	6.06E–06	-0.393	9.66E–08
rs2290344	*PIGB*	0.253	15	53407088	Coding	224856_at	*PIGB*	15	0.339	1.75E–05	-0.301	4.81E–07	-0.294	8.98E–05
rs4774760	*PIGB*	0.416	15	53376504	Upstream	242760_x_at	*PIGB*	15	0.326	3.63E–05	-0.240	6.85E–05	-0.294	8.98E–05
rs8024695	*PIGB*	0.285	15	53426597	Intron	242760_x_at	*PIGB*	15	0.321	5.00E–05	-0.282	2.56E–06	-0.294	8.98E–05
rs28668016	*PIGB*	0.365	15	53398725	5UTR	242760_x_at	*PIGB*	15	0.304	1.31E–04	-0.274	5.28E–06	-0.294	8.98E–05
**(B) AraC**														
rs10495285	*KIAA0133 (URB2)*	0.171	1	227871618	Downstream	219526_at	*C14orf169*	14	0.393	4.13E–07	-0.240	6.97E–05	-0.318	1.86E–05
rs9861198	Probable leucyl–tRNA synthetase	0.086	3	45310394	Upstream	235936_at	*LOC254559*	15	0.308	9.07E–05	0.274	5.12E–06	0.294	8.27E–05
rs9816196	U3 small nucleolar RNA	0.055	3	45321382	Upstream	235936_at	*LOC254559*	15	0.315	6.70E–05	0.242	7.05E–05	0.294	8.27E–05
rs6441911	*RIS1*	0.055	3	45322523	Flanking_5UTR	235936_at	*LOC254559*	15	0.315	6.20E–05	0.250	3.26E–05	0.294	8.27E–05
rs9878275	Probable leucyl–tRNA synthetase	0.055	3	45331043	Upstream	235936_at	*LOC254559*	15	0.311	8.31E–05	0.249	3.79E–05	0.294	8.27E–05
rs9846284	U3 small nucleolar RNA	0.055	3	45331970	Upstream	235936_at	*LOC254559*	15	0.315	6.20E–05	0.250	3.37E–05	0.294	8.27E–05
rs9850725	*LARS2*	0.055	3	45332431	Flanking_5UTR	235936_at	*LOC254559*	15	0.315	6.20E–05	0.250	3.26E–05	0.294	8.27E–05
rs9861198	Probable leucyl–tRNA synthetase	0.086	3	45310394	Upstream	206603_at	*SLC2A4*	17	0.308	9.07E–05	0.239	7.63E–05	0.345	3.18E–06
rs9816196	U3 small nucleolar RNA	0.055	3	45321382	Upstream	206603_at	*SLC2A4*	17	0.315	6.70E–05	0.294	1.21E–06	0.345	3.18E–06
rs6441911	*RIS1*	0.055	3	45322523	Flanking_5UTR	206603_at	*SLC2A4*	17	0.315	6.20E–05	0.301	4.80E–07	0.345	3.18E–06
rs9878275	Probable leucyl–tRNA synthetase	0.055	3	45331043	Upstream	206603_at	*SLC2A4*	17	0.311	8.31E–05	0.300	5.67E–07	0.345	3.18E–06
rs9846284	U3 small nucleolar RNA	0.055	3	45331970	Upstream	206603_at	*SLC2A4*	17	0.315	6.20E–05	0.301	5.03E–07	0.345	3.18E–06
rs9850725	*LARS2*	0.055	3	45332431	Flanking_5UTR	206603_at	*SLC2A4*	17	0.315	6.20E–05	0.301	4.80E–07	0.345	3.18E–06
rs9816196	U3 small nucleolar RNA	0.055	3	45321382	Upstream	1553755_at	*NXNL1*	19	0.315	6.70E–05	0.237	9.98E–05	0.312	2.82E–05
rs6441911	*RIS1*	0.055	3	45322523	Flanking_5UTR	1553755_at	*NXNL1*	19	0.315	6.20E–05	0.245	4.73E–05	0.312	2.82E–05
rs9878275	Probable leucyl–tRNA synthetase	0.055	3	45331043	Upstream	1553755_at	*NXNL1*	19	0.311	8.31E–05	0.244	5.26E–05	0.312	2.82E–05
rs9846284	U3 small nucleolar RNA	0.055	3	45331970	Upstream	1553755_at	*NXNL1*	19	0.315	6.20E–05	0.245	4.88E–05	0.312	2.82E–05
rs9850725	*LARS2*	0.055	3	45332431	Flanking_5UTR	1553755_at	*NXNL1*	19	0.315	6.20E–05	0.245	4.73E–05	0.312	2.82E–05
rs17564430	*IGSF4 (CADM1)*	0.155	11	114548784	Flanking_3UTR	206571_s_at	*MAP4K4*	2	-0.337	1.74E–05	0.237	8.48E–05	-0.322	1.51E–05
rs11215406	*IGSF4 (CADM1)*	0.138	11	Intron114570292	Intron	206571_s_at	*MAP4K4*	2	-0.335	1.92E–05	0.248	3.93E–05	-0.322	1.51E–05
rs11215406	*IGSF4 (CADM1)*	0.138	11	114570292	Intron	204201_s_at	*PTPN13*	4	-0.335	1.92E–05	0.243	5.49E–05	-0.301	5.30E–05
rs17564430	*IGSF4 (CADM1)*	0.155	11	114548784	Flanking_3UTR	204880_at	*MGMT*	10	-0.337	1.74E–05	-0.236	9.07E–05	0.332	7.44E–06
rs17564430	*IGSF4 (CADM1)*	0.155	11	114548784	Flanking_3UTR	1556095_at	*UNC13C*	15	-0.337	1.74E–05	0.256	2.21E–05	-0.354	1.60E–06
rs17564430	*IGSF4 (CADM1)*	0.155	11	114548784	Flanking_3UTR	1556096_s_at	*UNC13C*	15	-0.337	1.74E–05	0.240	7.07E–05	-0.337	5.47E–06
rs17564430	*IGSF4 (CADM1)*	0.155	11	114548784	Flanking_3UTR	1569969_a_at	*UNC13C*	15	-0.337	1.74E–05	0.257	1.93E–05	-0.332	7.56E–06
rs11215406	*IGSF4 (CADM1)*	0.138	11	114570292	Intron	1556095_at	*UNC13C*	15	-0.335	1.92E–05	0.276	4.27E–06	-0.354	1.60E–06
rs11215406	*IGSF4 (CADM1)*	0.138	11	114570292	Intron	1556096_s_at	*UNC13C*	15	-0.335	1.92E–05	0.255	2.29E–05	-0.337	5.47E–06
rs11215406	*IGSF4 (CADM1)*	0.138	11	114570292	Intron	1569969_a_at	*UNC13C*	15	-0.335	1.92E–05	0.270	7.08E–06	-0.332	7.56E–06
rs11215416	*IGSF4 (CADM1)*	0.147	11	114582537	Intron	1556095_at	*UNC13C*	15	-0.346	9.57E–06	0.260	1.59E–05	-0.354	1.60E–06
rs11215416	*IGSF4 (CADM1)*	0.147	11	114582537	Intron	1556096_s_at	*UNC13C*	15	-0.346	9.57E–06	0.242	6.21E–05	-0.337	5.47E–06
rs11215416	*IGSF4 (CADM1)*	0.147	11	114582537	Intron	1569969_a_at	*UNC13C*	15	-0.346	9.57E–06	0.258	1.84E–05	-0.332	7.56E–06
rs2008801	*IGSF4 (CADM1)*	0.126	11	114513385	Flanking_3UTR	225532_at	*CABLES1*	18	-0.317	5.42E–05	-0.254	2.54E–05	0.271	2.92E–04
rs2507905	*IGSF4 (CADM1)*	0.126	11	114513815	Downstream	225532_at	*CABLES1*	18	-0.317	5.42E–05	-0.254	2.54E–05	0.271	2.92E–04
rs7122402	*IGSF4 (CADM1)*	0.126	11	114521517	Downstream	225532_at	*CABLES1*	18	-0.317	5.42E–05	-0.261	1.43E–05	0.271	2.92E–04
rs4938179	*IGSF4 (CADM1)*	0.132	11	114538635	Downstream	225532_at	*CABLES1*	18	-0.289	2.56E–04	-0.247	4.34E–05	0.271	2.92E–04
rs17564430	*IGSF4 (CADM1)*	0.155	11	114548784	Flanking_3UTR	225532_at	*CABLES1*	18	-0.337	1.74E–05	-0.242	6.19E–05	0.271	2.92E–04

“Integrated analyses” with the top expression probe sets that were associated with SNPs within loci with gemcitabine IC50 (SNP vs. Expression P value <10^–4^, and Expression vs. IC50 P value < 10^–4^), except for C3orf23 (SNP vs. Expression P value < 10^–4^, and Expression vs. IC50 P value ≈ 10^–4^). Eighteen unique probe sets, representing 12 annotated genes that were associated with 66 SNPs within 7 loci and were significantly correlated with gemcitabine IC50 values are listed. (B) “Integrated analyses” for AraC. Eleven unique probe sets, representing 6 annotated genes that were associated with 15 SNPs within 3 loci and were significantly correlated with AraC IC50 values are listed. R values represent correlation coefficients for each association.

### Follow-up functional validation of candidate genes in cancer cells

**Figure 2:**
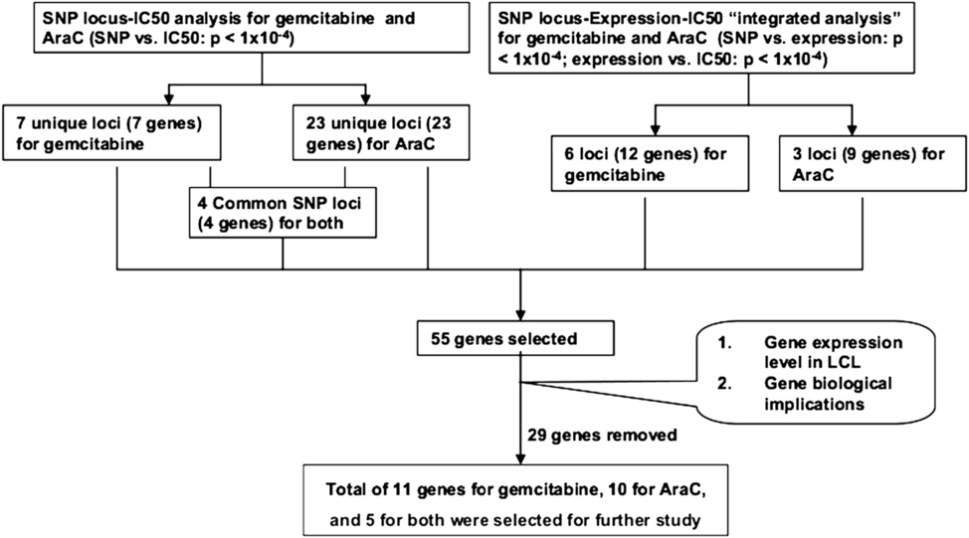
**Schematic diagram of the strategy for candidate gene selection for functional validation.** Genome-wide association studies for either gemcitabine or AraC cytotoxicity were performed with 1.3 million SNPs or 54,000 expression probe sets. “Integrated analyses” were performed using SNP “loci” that contained at least 2 SNPs (P < 10^-4^) or 1 SNP (P < 10^-4^) plus 3 SNPs (P < 10^-3^) within 100 kb surrounding the top significant SNPs, 54,000 expression probe sets and IC50 values to identify SNPs associated with drug IC50 values through their influence on gene expression (SNP-Expression P value <10^-4^, Expression-IC50 P value <10^-4^). Finally, 26 candidate genes, including 11 for gemcitabine, 10 for AraC, and 5 for both, were selected for functional validation studies that were performed with multiple cancer cell lines.

**Figure 3:**
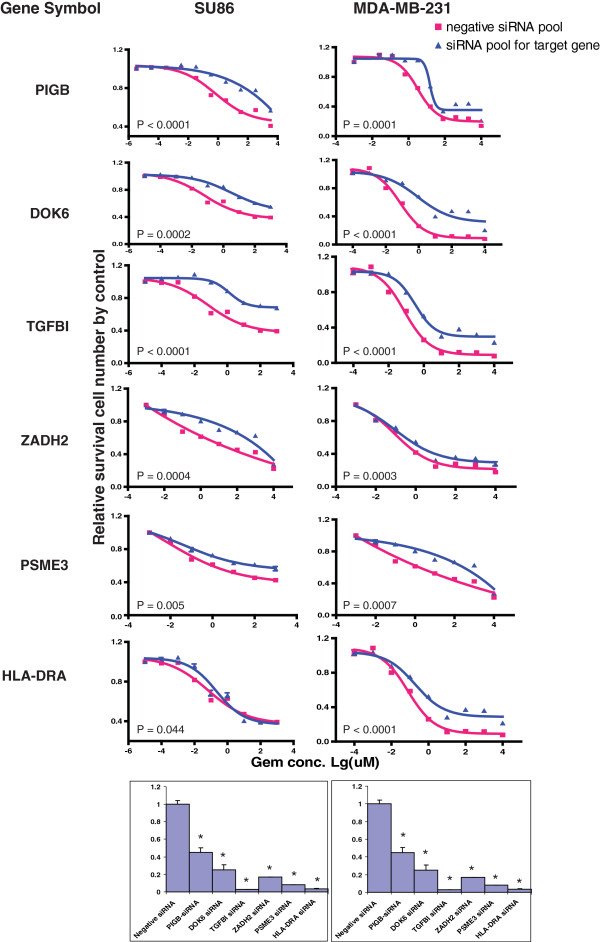
**Functional characterization of candidate genes.** Knockdown of individual genes in cancer cell lines followed by MTS assays to determine the effect of the candidate gene on gemcitabine. Data are shown for 11 validated out of the 26 candidate genes tested in SU86, MDA-MB-231, BDCM, and THP-1 cancer cell lines by MTS assay after siRNA knockdown performed with a pool of 4 specific siRNAs. The drug dose response curves were obtained with the MTS assays. Red lines indicate the negative control siRNA pool, while blue lines indicate data obtained with specific siRNA pool. The x-axis indicates the log10 gemcitabine concentration and the y-axis indicates the proportion of cells surviving after drug exposure. The bar graphs at the bottom show knockdown efficiency tested by QRT-PCR assay using the same transfected cells as those used to perform the MTS assays. The y-axis indicates relative gene expression after siRNA knockdown when compared with negative control siRNA. The experiments were repeated 3 times and the error bar represents SEM. Each of the genes was significantly knocked down when compared to the negsiRNA control, P<0.05.

**Figure 4:**
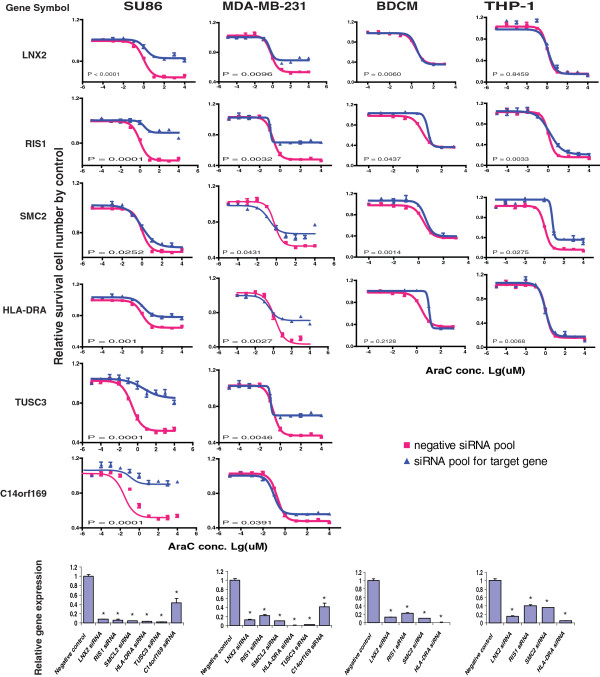
**Functional characterization of candidate genes.** Knockdown of individual genes in cancer cell lines followed by MTS assays to determine the effect of the candidate gene on AraC. Data are shown for 11 validated out of the 26 candidate genes tested in SU86, MDA-MB-231, BDCM, and THP-1 cancer cell lines by MTS assay after siRNA knockdown performed with a pool of 4 specific siRNAs. The drug dose response curves were obtained with the MTS assays. Red lines indicate the negative control siRNA pool, while blue lines indicate data obtained with specific siRNA pool. The x-axis indicates the log10 AraC concentration and the y-axis indicates the proportion of cells surviving after drug exposure. The bar graphs at the bottom show knockdown efficiency tested by QRT-PCR assay using the same transfected cells as those used to perform the MTS assays. The y-axis indicates relative gene expression after siRNA knockdown when compared with negative control siRNA. The experiments were repeated 3 times and the error bar represents SEM. Each of the genes was significantly knocked down when compared to the negsiRNA control, P<0.05.

Since the regulation of gene expression is tissue specific [28], we wanted to functionally validate in cancer cell lines candidate genes selected based on our analysis performed with LCLs. The tumor cell lines that we selected were based on the expression of the genes of interest and on the clinical uses of these two drugs. Gemcitabine is used in the treatment of pancreatic cancer but it is also used to treat other solid tumors such as breast cancer, while AraC is first-line therapy for acute myelogenous leukemia (AML). Therefore, we selected one human pancreatic cancer cell line, SU86, one breast cancer cell line, MDA-MB-231 and two leukemia cell lines, BDCM and THP1, to functionally characterize the genes of interest. Twenty-six genes were selected based on a series of criteria including association P value, SNP locus, whether the gene was expressed in LCLs and the biological function of the genes (Table 3 and Figure 2). To determine the functional impact of those genes, we used specific siRNA pools to knockdown the 26 candidate genes, followed by QRT-PCR and MTS cytotoxicity assay. Eleven genes showed an effect on gemcitabine cytotoxicity, 10 on AraC and 5 were common to both drugs. Knockdown of PIGB, ZADH2, PSME3, DOK6, TGFBI, and HLA-DRA in both SU86 and MDA-MB-231 cells significantly desensitized the cells to gemcitabine (Table 3 and Figure 3), consistent with the association study results. Knockdown of C4orf169, TUSC3, LNX2, RIS1, and SMC2 and HLA-DRA in both SU86 and MDA-MB-231 cells significantly desensitized the cells to AraC (Table 3 and Figure 4). Finally, knockdown of HLA-DRA in THP-1 leukemia cells, LNX2 in BDCM cells, and SMC2 and RIS1 in both THP1 and BDCM cells also desensitized the cells to AraC, results that were also consistent with our association results (Figure 4).

**Table 3:** Functional studies of candidate genes

**Gene symbol**	**Selection strategy**			**Cytotoxicity validation in cancer cell lines**	
	**SNP vs. IC50**		**Integrated analysis**	**Both SU86 and MDA231**	**Either THP-1 or BDCM**
	**SNP Location**	**P < 10-4**	**SNP vs. Exp P < 10-4**	**(cancer cell lines)**	**(leukemia cell lines)**
			**Exp vs. IC50 P <10-4**		
***PIGB***	Intron/coding/5UTR/3UTR	Gem	Gem	Yes	NP
*C3orf23*	5UTR/upstream	Gem	Gem	No	NP
***ZADH2***			Gem	Yes	NP
*ARHGEF12*			Gem	No	NP
*DPP7*			Gem	No	NP
***PSME3***			Gem	Yes	NP
*NIPSNAP3B*	Intron/5UTR	Gem		No	NP
*PIK3R1*	Flanking_5UTR/3UTR	Gem		No	NP
***DOK6***	Intron	Gem		Yes	NP
***TGFBI***	Flanking_5UTR	Gem		Yes	NP
*UNC13C*			AraC	No	No
***C14orf169***			AraC	Yes	NP
*MAP4K4*			AraC	No	NP
*URB2*	Downstream	AraC		No	No
***TUSC3***	Intron/5UTR/3UTR	AraC		Yes	No
*LARS2*	5UTR	AraC		Yes	No
***RIS1 (TMEM158)***	5UTR	AraC		Yes	Yes
*IGSF4 (CADM1)*	Intron/downstream/3UTR	AraC		No	No
***LNX2***	Intron/5UTR/3UTR	AraC		Yes	No
***SMC2***	3UTR	AraC		Yes	Yes
*PLD5*	Intron	Both		No	NP
*GPR98*	Intron	Both		No	No
***HLA-DRA***	Intron/3UTR	Both		Yes	No
*ZNF215*	Flanking_5UTR/3UTR/coding	Both		No	No
*CABLES1*			Both	No	No

The table lists the top 26 candidate genes selected for siRNA screening for both gemcitabine and AraC, with MTS assay results as well as QRT-PCR assay results when appropriate.

A total of 26 genes were selected for siRNA screening, with 11 genes for gemcitabine, 10 for AraC, and 5 for both. For the validation assays with MTS and QRT-PCR, “Yes” indicates that knockdown of the gene altered drug cytotoxicity, while “No” indicates no alteration. “NP” indicates not performed. The genes that are bolded were functionally validated.

**Figure 5:**
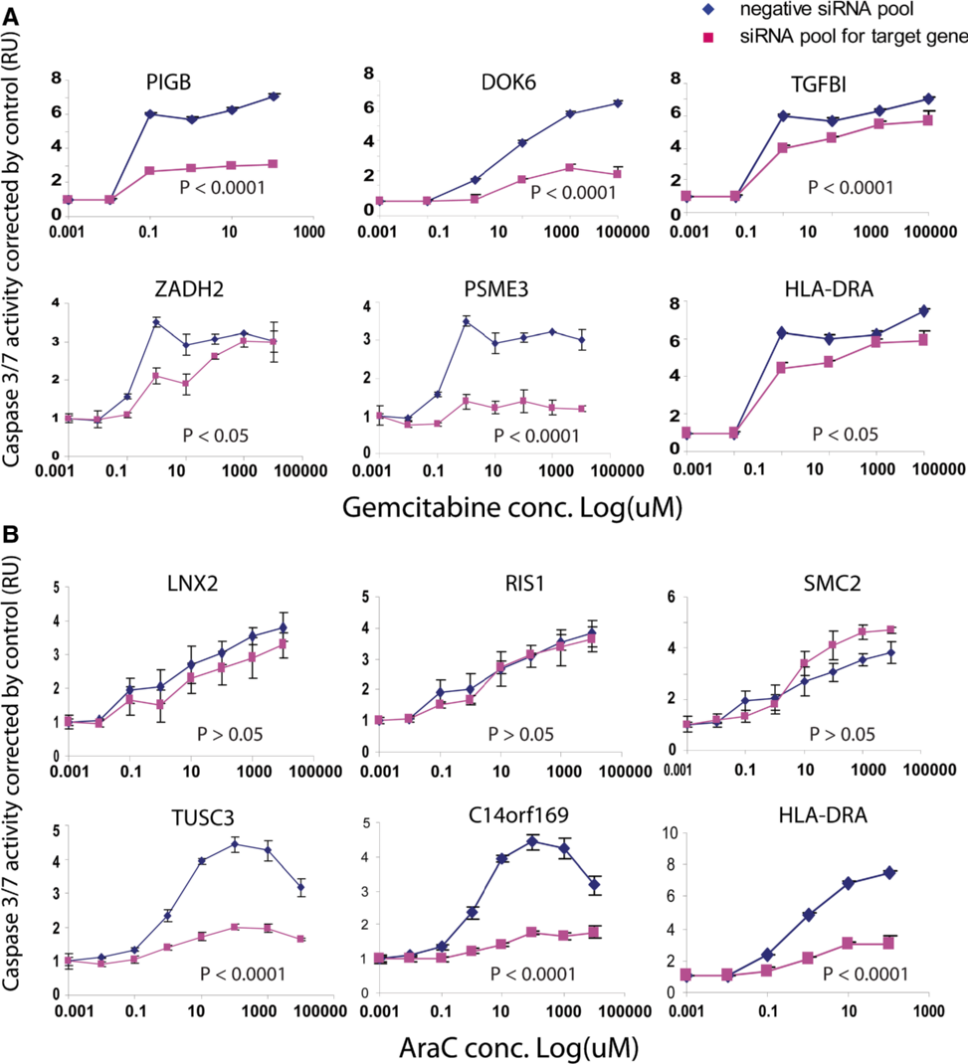
**Effect of candidate genes on apoptosis in SU86 cells.** Apoptosis was measured in SU86 cancer cell line after transfection with a pool of 4 specific siRNAs for 24 h and exposure to gemcitabine **(A)** or AraC **(B)** for an additional 48 h, followed by Caspase-3/7 assay. The x-axis indicates the drug dose and the y-axis represents the relative Caspase 3/7 activity normalized to nontreatment control. P values represented the significant difference in the AUC values derived from the curves between the control and specific knockdown.

We next wanted to determine whether the cytotoxic effects of those genes might involve apoptosis. Therefore, we performed caspase-3/7 activity assays after knockdown of the candidate genes in SU86 cells. As shown in Figure 5A and 5B, down-regulation of PIGB, DOK6, TGFBI, ZADH2, PSME3, and HLA-DRA in SU86 cells significantly decreased caspase-3/7 activity after treatment with gemcitabine as compared with negative control siRNA-treated cells. Similar results were also observed for AraC treatment following siRNA knockdown of TUSC3, C14orf169, and HLA-DRA. However, knockdown of LNX2, RIS1, and SMC2 did not alter the cellular caspase-3/7 activity (Table 4), suggesting that a different mechanism was involved.

**Table 4:** Functional studies of candidate genes

**Gene symbol**	**Caspase-3/7 activity**	**Cancer-related Cignal Reporter Array**									
		**Wnt (TCF/LEF)**	**Notch (RBP-Jκ)**	**p53/DNA damage (p53)**	**TGFβ (SMAD2/3/4)**	**cell cycle (E2F/DP1)**	**NFκB (NFκB)**	**c-Myc (Myc/Max)**	**Hypoxia (HIF1A)**	**MAPK /ERK (Elk-1/SRF)**	**MAPK /JNK (AP-1)**
*PIGB*	Decrease	No	No	No	No	No	Decrease	Decrease	No	Decrease	Decrease
*ZADH2*	Decrease	No	No	No	No	No	No	Increase	No	No	No
*PSME3*	Decrease	No	Increase	No	Decrease	No	No	Increase	Decrease	No	No
*DOK6*	Decrease	No	No	No	No	No	Decrease	No	No	No	Decrease
*TGFBI*	Decrease	No	No	Increase	No	No	Decrease	Decrease	Decrease	Decrease	Decrease
*C14orf169*	Decrease	No	Increase	Increase	No	No	Increase	Increase	No	Increase	Increase
*TUSC3*	Decrease	No	No	No	Decrease	Decrease	No	No	Decrease	No	No
*RIS1 (TMEM158)*	No	No	No	No	No	No	No	Increase	No	No	No
*LNX2*	No	No	No	No	No	No	No	No	No	Increase	No
*SMC2*	No	No	No	No	No	No	No	No	No	No	No
*HLA-DRA*	Decrease	No	Increase	No	No	No	Increase	No	No	No	No

Functional characterization of 11 genes using the Cancer-related Cignal Reporter Array (SABioscience).

Transcription factors for each signal pathway are listed in parenthesis. “Increase” means at least a 2-fold increase of luciferase activity in cells treated with specific siRNA compared with cells treated with negative control siRNA; while “decrease” indicates at least a 50% decrease of luciferase activity in control cells. “No” means no alteration in luciferase activity after siRNA knockdown.

**Figure 6:**
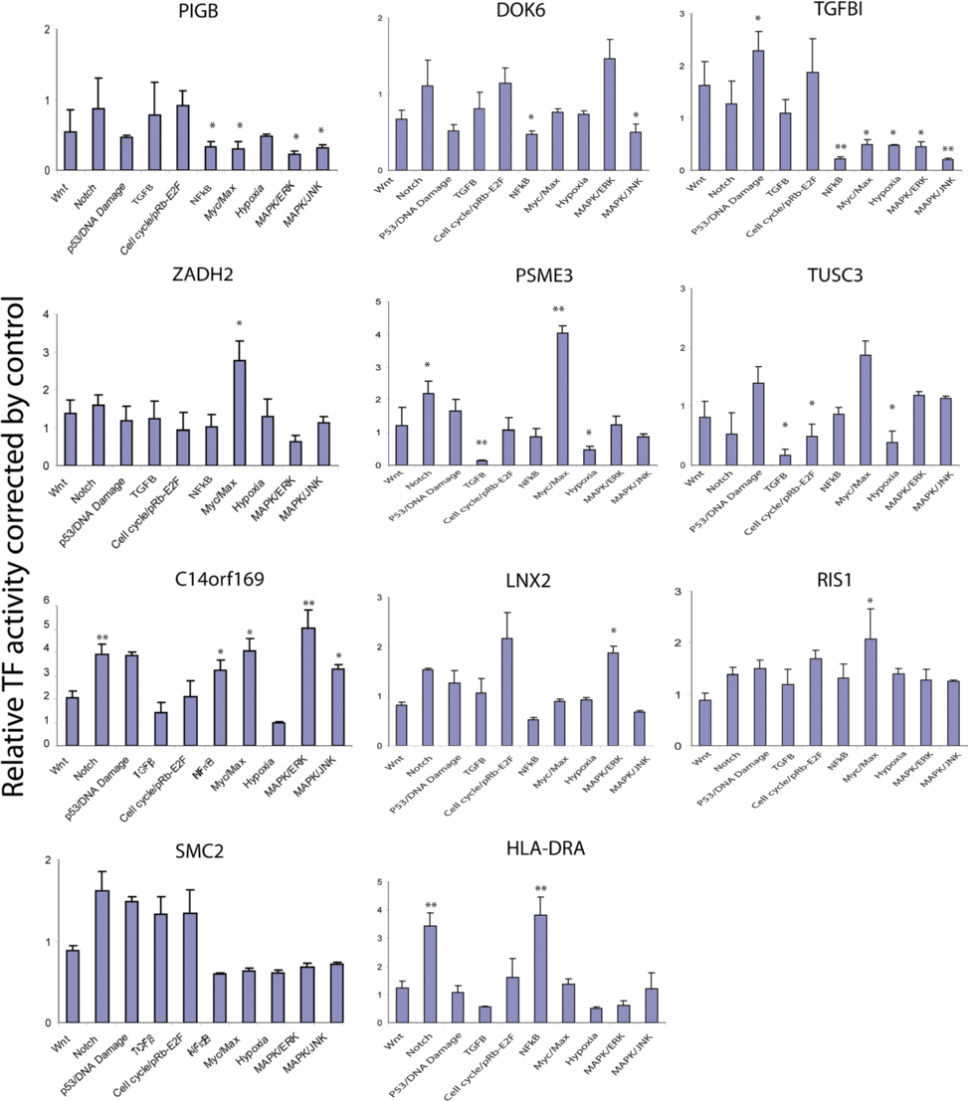
**Effect of candidate gene knockdown on 10 cancer related pathways in SU86 cells.** The Cancer Cignal Reporter assay was used to determine the effect of candidate gene knockdown on 10 cancer related signaling pathways. Each column indicates relative luciferase activity of the transcription factor-transfected SU86 cells. The x-axis indicates individual TF dependent cancer signaling pathways. * indicates P < 0.05 as compared to control cells, while ** indicates P < 0.01.

Finally, we used the Cancer Cignal Finder Array (SABioscience) that consists of 10 dual-luciferase reporter gene assays to determine whether our candidate genes might affect any of the 10 cancer-related signaling pathways in SU86 cells by measuring changes in transcriptional activities of 10 key transcription factors (TF) after knockdown of each candidate gene. We observed changes in transcriptional activity of several TFs after knockdown of specific genes in SU86 cells, suggesting that these genes might be involved in the regulation of a particular cancer-related signaling pathway or pathways that might contribute to resistance to gemcitabine and AraC (Figure 6 and Table 3). For example, knockdown of PIGB resulted in a decrease in transcriptional activity of Elk-1/SRF, AP1, NFκB, and Myc/MAX in SU86 cells, indicating a down-regulation of these signaling pathways. Knockdown of DOK6 dramatically decreased the transcription activities of both NFκB and AP1 in the NFκB and MAPK/JNK pathways, while the activity of the transcription factor Myc/MAX that is involved in the c-Myc pathway was increased significantly after ZADH2 knockdown. However, we did not observe any significant changes after SMC2 knockdown.

### Functional characterization of *PIGB* SNPs

**Figure 7:**
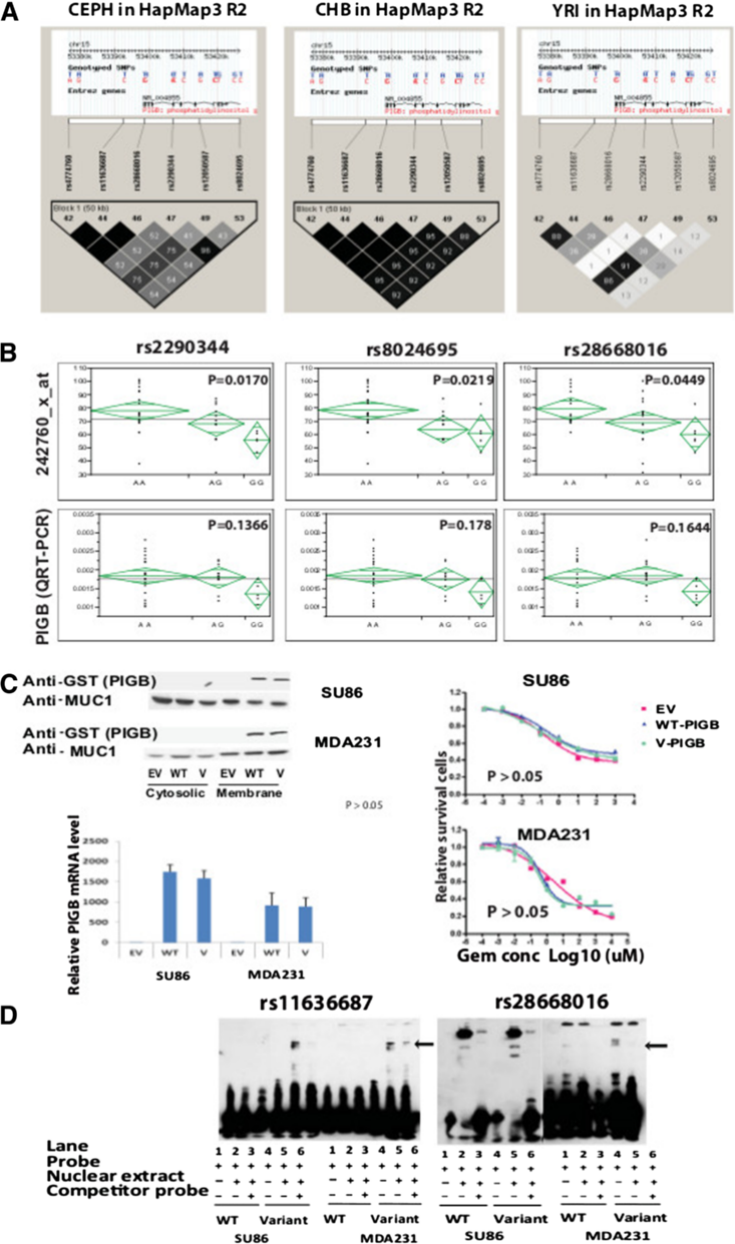
**Functional characterization of SNPs in the *PIGB *gene. (A)** Patterns of linkage disequilibrium (LD) within ~200 Kb surrounding the rs3797418 SNP among three different ethnic groups using HapMap3 R2 data as a reference. The top 7 SNPs in PIGB are arranged in order from 5′ to 3′, as shown in the gene structure above each plot. Black indicates combinations where R^2^ = 1 and linkage of disequilibrium (LOD) ≥ 2; light grey, combinations where 0 < R^2^ < 1 and LOD ≥ 2; white, where R^2^ = 0 and LOD < 2. **(B)** Association between SNPs and PIGB expression measured by QRT-PCR assay and microarray using 37 randomly selected LCLs. **(C)** Effect of the nonsynonymous coding SNP (rs2290344, M161T) on PIGB mRNA expression, protein expression and response to gemcitabine. PIGB mRNA and protein levels were determined in SU86 and MDA-MB-231 cells transfected with either PIGB wild-type or variant constructs with GST-tags. Antibody against GST (Antibody #2622, Cell Signaling Inc.) was used for detection of *PIGB* expression, and Antibody against MUC1 (VU4H5 Mouse mAb #4538, Cell Signaling Inc.) served as a loading control in Western Blot assay. Gemcitabine cytotoxicity performed with the MTS assay was performed in cells transfected with WT and variant constructs. No differences were observed between WT and variant SNP for any of the phenotypes tested. **(D)** EMSAs were performed for two regulatory SNPs in *PIGB* gene. The arrows indicate different binding pattern between WT and variant sequences for rs11636687 and rs28668016.

When we performed integrated analysis among SNPs, gene expression and gemcitabine cytotoxicity, we found that the only *cis-*regulated SNPs mapped to *PIGB*. Knockdown of PIGB resulted in desensitization of cancer cells to gemcitabine. *PIGB* contained 7 SNPs that were associated both with gemcitabine response (P < 10^-3^) and with its own gene expression (P < 10^-4^) (Table 5). PIGB expression was also significantly correlated with gemcitabine cytotoxicity (P = 8.95 × 10^-5^ and P = 5.31 × 10^-3^ for two different probe sets for PIGB mRNA). We also determined LD patterns for those 7 SNPs using HapMap data for each ethnic group. As shown in Figure 7A, LD patterns differed among the three ethnic groups. In both CHB/JPT and CEPH groups, those 7 SNPs were in tight LD, while there was not significant linkage among the SNPs in the YRI population. The top 3 SNPs in *PIGB,* including rs2290344, a nonsynonymous coding SNP (M161T) in exon 4, rs28668016 in the 5′-UTR, and rs11636687 in the 5′ flanking region (Table 5) were selected for further functional characterization.

**Table 5:** **The top seven SNPs in *PIGB *** **that were associated with gemcitabine cytotoxicity and its expression in LCLs by integrated analysis**

**SNP**					**Gene**			**SNP vs. IC50**		**SNP vs. Expression**		**Expression vs. IC50**	
** rsID**	**Chr**	**Position**	**Region**	**MAF**	** Probeset**	**Gene symbol**	** Chr**	**R value**	** P value**	** R value**	** P value**	** R value**	** P value**
rs2290344	15	53407088	Coding	0.253	205452_at	*PIGB*	15	0.339	1.75E-05	−0.340	1.06E-08	−0.212	5.31E-03
rs2290344	15	53407088	Coding	0.253	242760_x_at	*PIGB*	15	0.339	1.75E-05	−0.301	4.81E-07	−0.294	8.98E-05
rs4774760	15	53376504	5′-upstream	0.416	205452_at	*PIGB*	15	0.326	3.63E-05	−0.283	2.48E-06	−0.212	5.31E-03
rs4774760	15	53376504	5′-upstream	0.416	242760_x_at	*PIGB*	15	0.326	3.63E-05	−0.240	6.85E-05	−0.294	8.98E-05
rs8024695	15	53426597	Intron	0.285	205452_at	*PIGB*	15	0.321	5.00E-05	−0.326	4.60E-08	−0.212	5.31E-03
rs8024695	15	53426597	Intron	0.285	242760_x_at	*PIGB*	15	0.321	5.00E-05	−0.282	2.56E-06	−0.294	8.98E-05
rs12050587	15	53414820	Intron	0.450	205452_at	*PIGB*	15	0.305	1.23E-04	−0.246	4.54E-05	−0.212	5.31E-03
rs28668016	15	53398725	5′-UTR	0.365	205452_at	*PIGB*	15	0.304	1.31E-04	−0.346	5.97E-09	−0.212	5.31E-03
rs28668016	15	53398725	5′-UTR	0.365	242760_x_at	*PIGB*	15	0.304	1.31E-04	−0.274	5.28E-06	−0.294	8.98E-05
rs11636687	15	53392444	5′-upstream	0.421	205452_at	*PIGB*	15	0.302	1.53E-04	−0.287	2.22E-06	−0.212	5.31E-03
rs7174876	15	53406853	Intron	0.423	205452_at	*PIGB*	15	0.284	4.06E-04	−0.261	1.64E-05	−0.212	5.31E-03

R values represent correlation coefficients for associations. We performed integrated analysis among the SNPs and mRNA expression of the *PIGB* gene as well as gemcitabine cytotoxicity. The only *cis-*associated SNPs with *PIGB* gene expression were listed.

We first determined *PIGB* expression levels in 37 LCLs selected on the basis of genotypes for those 3 SNPs using both QRT-PCR assay and expression array data to confirm the association between the SNPs and *PIGB* expression. Cells carrying the variant alleles showed significantly lower expression levels than did WT cells (Figure 7B). We next determined the functional impact of these 3 SNPs. As shown in Figure 7C, overexpression of a construct for the *PIGB* coding SNP (rs2290344, M161T) in SU86 and MDA-MB-231 cells did not alter either mRNA or protein levels, nor did it have an effect on gemcitabine cytotoxicity. We then determined whether the two SNPs in regulatory regions, rs11636687 and rs28668016, might have functional impact. We performed electrophoresis mobility shift assays (EMSAs) for these two SNPs to determine whether there might be differences in binding patterns for possible transcription factors. Interestingly, the results from EMSA showed that DNA-protein binding was significantly increased for the probe containing the variant sequences for these two SNPs in both SU86 and MDA-MB-231 cells (Figure 7D). These results suggested that these two SNPs might alter the binding of transcription factors and, as a result, affect PIGB expression level.

## Discussion

We previously performed a genome wide SNP association study with 550 K SNPs obtained with Illumina HumanHap550 BeadChips for the same cell lines to identify common polymorphisms that might influence both gene expression and response to these two drugs [18]. In the present study, we expanded the number of SNPs from 550 K to include over 1.3 million SNPs and selected candidate genes for functional follow-up studies based on SNP loci. This dense SNP coverage made it possible to identify many more candidates for functional follow-ups. That enabled us to take a different approach by focusing on “SNP loci” instead of single SNPs. The results listed in Table 3 show that 11 of 26 candidate genes selected in this fashion were validated functionally, while only two other genes from the previous 550 k studies were functionally validated [18].

We also tested the concordance of the results generated with 550 K and 1.3 million SNPs if we had used the same strategy as we did in the current study, i.e. using SNP loci to perform the association studies. The majority of top SNP peaks from the 550 K SNP data for both drugs displayed less significant SNPs for each locus as compared to the 1.3 million SNP data (Additional file 1: Table S3). These observations illustrate the advantage of the present selection strategy for candidate identification, as well as the advantage of using denser SNP coverage.

Of the 26 candidate genes that we identified for further siRNA screening followed by MTS assay, eleven candidate genes, including PIGB, TGFBI, DOK6, PSME3, ZADH2, TUSC3, C14orf169, SMC2, LNX2, RIS1, and HLA-DRA, showed a significant effect on response to gemcitabine and/or AraC in SU86 and/or MDA-MB231 cells. To identify potential pathways with which these genes might be involved, we used a dual luciferase reporter gene assay to assess the impact of these genes on 10 major cancer-related signaling pathways. As shown in Figure 6 and Table 3, except for the SMC2 gene, knockdown of the other 10 genes in SU86 cells significantly altered activities, based on the luciferase assay for at least one of the 10 cancer related signaling pathways. Genes such as TGFB1 showed changes for the most pathways. While TGFB1 has been well studied, genes such as C14orf169, an unknown gene, also showed increased activity in 7 of the 10 pathways.

We also observed that the activities of the Elk-1/SRF, AP1, NFκB, and Myc/MAX pathways were significantly decreased in SU86 cells when PIGB was down-regulated by a specific siRNA. PIGB, a gene of the phosphatidylinositol glycan (PIG) class B, encodes an enzyme involved in the synthesis of a glycosylphosphatidylinositol (GPI) anchor that is a membrane attachment structure for many proteins, including membranous enzymes, receptors, differentiation antigens, and other biologically active proteins [29]. GPI anchoring is essential for the expression of many of those proteins in either biological processes or cancer progression [30,31]. The PIGB protein is a GPI mannosyltransferase III and is required for the transfer of the third mannose into the core structure of the GPI anchor [29,32]. Previous studies have demonstrated that other PIG class members, such as PIGU and PIGT, are oncogenes in either human bladder cancer or breast cancer, respectively [33,34]. Our findings indicate that PIGB is involved in sensitizing cancer cells to both gemcitabine and AraC, suggesting a possible role in oncogenic pathways as well as chemoresistance. The 8 *PIGB* SNPs were also associated with the expression of FKBP5, a gene that we previously reported to be important for gemcitabine and AraC response [17,27]. Furthermore, PIGB expression itself is also correlated with FKBP5 gene expression. Although down regulation of PIGB altered FKBP5 mRNA level, overexpression of FKBP5 in PIGB stable knockdown cell lines did not change response to gemcitabine or AraC (Additional file 1: Figure S3). These observations indicate that PIGB influences the cytotoxicity of the two cytidine analogues through mechanisms that differ from FKBP5, in spite of the correlation of their expression levels observed in the LCLs. The exact mechanisms by which PIGB affects gemcitabine and AraC cytotoxicity need to be explored in the course of future experiments.

In addition to the characterization of candidate genes, we also focused on SNPs in the *PIGB* gene that showed *cis*-regulation of PIGB expression. SNPs in regulatory regions can influence drug response through an influence on gene expression. During our analysis, we found that most SNP associations with expression were through *trans-*regulation. The reason that we focused on SNPs in *PIGB* is because those SNPs displayed *cis-*regulations of PIGB and knockdown of PIGB showed an effect on cytotoxicity. The EMSA results also demonstrated “shifts” for the variant SNP sequences (Figure 7D), suggesting that PIGB gene expression might be regulated through binding to those transcription factors.

Previous studies demonstrated that one mechanism by which SNPs might influence drug cytotoxicity is through transcription regulation in either a *cis*- or *trans*-manner [18,35-37]. In this analysis, we found SNPs that could both have cis or trans relationship. In addition to the SNPs that cis regulate PIGB, we also found that SNPs close to *C3orf23* were not only cis-associated with its own gene expression, but also trans-correlated with the expression of *ZADH2* which was confirmed to affect drug response of gemcitabine in our functional validation study. How those genetic variations located in the upstream of C3orf23 affect the expression of *ZADH2* gene in a trans- manner remains unknown. One mechanism might be that those SNPs nearby *C3orf23* could alter DNA sequence binding to transcription factors (TFs), microRNA, or other long non-coding RNA (lnc RNA), thus affect transcriptional regulation of their target genes including *ZADH2* gene, which could in turn, affect gemcitabine response.

## Conclusions

In summary, this study performed with LCLs followed by functional characterization has enhanced our understanding of the action of gemcitabine and AraC in the therapy of cancer. Although there are limitations associated with the use of LCLs [38,39], this system has proven to be extremely useful, both to generate pharmacogenomic hypothesis and to test pharmacogenomic signals identified during the clinical GWAS [19-21]. Future studies using patient samples will now be required to confirm the candidates identified during this study.

## Abbreviations

AraC: Cytosine arabinoside; LCL: Lymphoblastoid cell line; RRM1: Ribonucleotide reductase; CDA: Cytidine deaminase; CA: Caucasian-American; AA: African-American; HCA: African-American; EMSA: Electrophoresis mobility shift assays; QRT-PCR: Quantitative reverse transcription-PCR; QC: Quality control; HWE: Hardy-Weinberg equilibrium; AML: Acute myelogenous leukemia; TF: Transcription factors; GWAS: Genome-wide association studies; LD: Linkage disequilibrium.

## Competing interests

The authors declare that they have no competing interests.

## Authors’ contributions

LL, NN and LW designed the study and wrote the manuscript. LL and NN performed the experiments. LL, BF, KK, GJ, AB and LW analyzed the data. LL, NN, BK and LW wrote the manuscript. All authors read and approved the final manuscript.

## Supplementary Material

Additional file 1 **Contains supplementary figures and tables. Figure S1.** Imputation analysis for top SNP loci associated with the response of (A) gemcitabine, (B) AraC, and (C) both drugs. **Figure S2.** Validation of imputed genotypes. The x-axis indicates actual genotype by TaqMan assay. The y-axis represents imputed genotype, which was estimated as the count of a particular allele. The squared difference between the imputed genotype and actual genotype was calculated based on counting the same allele. Avg sq difference = average squared difference. **Figure S3.** Effect of FKBP5 on cellular sensitivity to either gemcitabine or AraC in which PIGB was stably knock down cell lines. **Table S1.** Top SNPs that were associated with (A) Gemcitabine, (B) AraC, or (C) both drugs with P values <10^-3^ during the GWAS. **Table S2.** SNPs associated with both expression and cytotoxicity data for either (A) Gemcitabine or (B) AraC from the “integrated analyses” with P values <10^-3^. **Table S3.** The top 9 loci for Gemcitabine (A) and the top 9 loci for AraC (B) that were associated with drug response-IC50 values using our previous 550,000 SNP array data. Click here for file
