# Supplementary material for: Discovery of genetic biomarkers contributing to variation in drug response of cytidine analogues using human lymphoblastoid cell lines
Source: BMC Genomics. 2014 Feb 1;15:93. doi: 10.1186/1471-2164-15-93 (PMC3930546; doi:10.1186/1471-2164-15-93)
Supplement: Additional file 1 — Contains supplementary figures and tables. Figure S1. Imputation analysis for top SNP loci associated with the response of (A) gemcitabine, (B) AraC, and (C) both drugs. Figure S2. Validation of imputed genotypes. The x-axis indicates actual genotype by TaqMan assay. The y-axis represents imputed genotype, which was estimated as the count of a particular allele. The squared difference between the imputed genotype and actual genotype was calculated based on counting the same allele. Avg sq difference = average squared difference. Figure S3. Effect of FKBP5 on cellular sensitivity to either gemcitabine or AraC in which PIGB was stably knock down cell lines. Table S1. Top SNPs that were associated with (A) Gemcitabine, (B) AraC, or (C) both drugs with P values <10-3 during the GWAS. Table S2. SNPs associated with both expression and cytotoxicity data for either (A) Gemcitabine or (B) AraC from the “integrated analyses” with P values <10-3. Table S3. The top 9 loci for Gemcitabine (A) and the top 9 loci for AraC (B) that were associated with drug response-IC50 values using our previous 550,000 SNP array data. [file 1471-2164-15-93-S1.pdf]

SUPPLEMENTARY MATERIAL

Discovery of genetic biomarkers contributing to variation in drug response of cytidine analogues using human lymphoblastoid cell lines

Legends for supplementary figures and tables

Figure S1. Imputation analysis for top SNP loci associated with the response of (A) gemcitabine, (B) AraC, and (C) both drugs. The x-axis indicates genomic regions surrounding +/-200kb regions of top SNP loci. The y-axis represents -log10 (p value) for the association of each SNP with drug IC50 values. Red dots indicate imputed SNPs while black dots indicate genotyped SNPs.

Figure S2. **Validation of imputed genotypes.** The x-axis indicates actual genotype by TaqMan assay. The y-axis represents imputed genotype, which was estimated as the count of a particular allele. The squared difference between the imputed genotype and actual genotype was calculated based on counting the same allele. Avg sq difference = average squared difference.

Figure S3. Effect of FKBP5 on cellular sensitivity to either gemcitabine or AraC in which PIGB was stably knock down cell lines. Either an FKBP5 construct or empty vector (EV) was mixed with lipofectamine 2000 transfection reagent (Invitrogen, Carlsbad, CA) for transient overexpression in SU86 (left) and MDA-MB-231 (right) cell lines that were stably transfected with PIGB-shRNA, followed by MTS assay (upper panels) and QRT-PCR analysis (lower panels).

Table S1. Top SNPs that were associated with (A) Gemcitabine, (B) AraC, or (C) both drugs with P values $<10^{-3}$ during the GWAS. R values represent correlation coefficients for associations. (A) The top 1280 SNPs that were associated with gemcitabine IC50 values with P values $<10^{-3}$. The 143 top SNPs in the list were significantly associated with gemcitabine IC50 values with P values $<10^{-4}$. (B) The top 1572 SNPs that were associated with AraC IC50 values with P values $<10^{-3}$. The 204 top SNPs in the list were significantly associated with AraC IC50 values with P values $<10^{-4}$. (C) The top 17 SNPs that were associated with both gemcitabine and AraC IC50 values with P values $<10^{-3}$. “Gem” indicates gemcitabine.

Table S2. SNPs associated with both expression and cytotoxicity data for either (A) Gemcitabine or (B) AraC from the “integrated analyses” with P values $<10^{-3}$. The lists include genotyped SNPs on the Affymetrix or Illumina platforms as well as imputed SNPs. "O" indicates SNPs observed during genotyping using either Affymetrix or Illumina SNP arrays; while "I" indicates imputed SNPs using HapMap R22 data as a reference.

Table S3. The top 9 loci for Gemcitabine (A) and the top 9 loci for AraC (B) that were associated with drug response-IC50 values using our previous 550,000 SNP array data. Each locus contained at least 2 SNPs within 50kb with P values $<10^{-4}$. R values represent correlation coefficients for the association. Number of SNPs indicates the number of significantly associated SNPs in the locus. rsID indicates the most significant SNP associated with drug cytotoxicity in the locus.

Figure S1

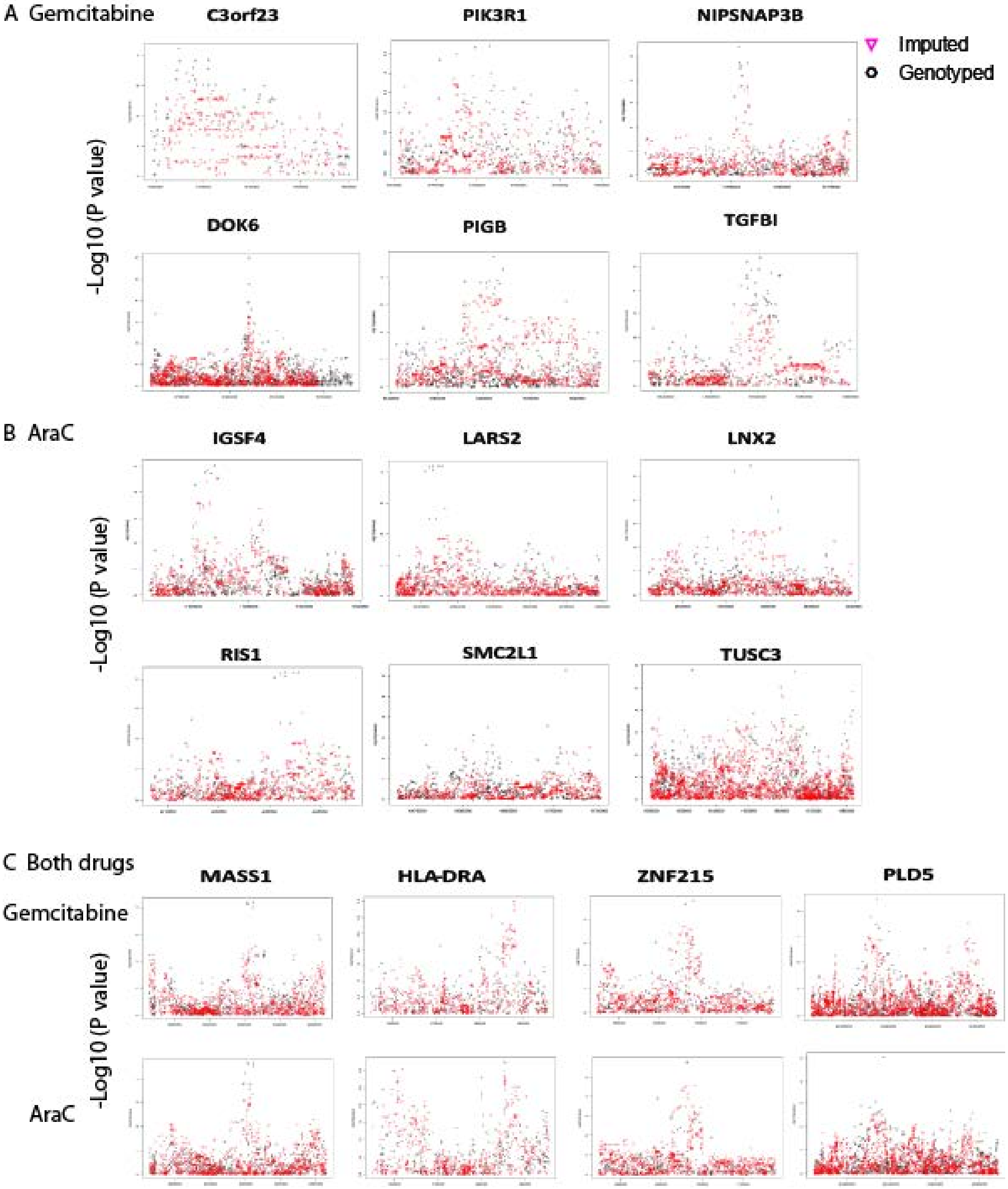
A Gemcitabine
C3orf23
PIK3R1
NIPSNAP3B
Imputed
Genotyped
-Log10 (P value)
DOK6
PIGB
TGFBI
B AraC
IGSF4
LARS2
LNX2
-Log10 (P value)
RIS1
SMC2L1
TUSC3
C Both drugs
MASS1
HLA-DRA
ZNF215
PLD5
Gemcitabine
-Log10 (P value)
AraC

Figure S2

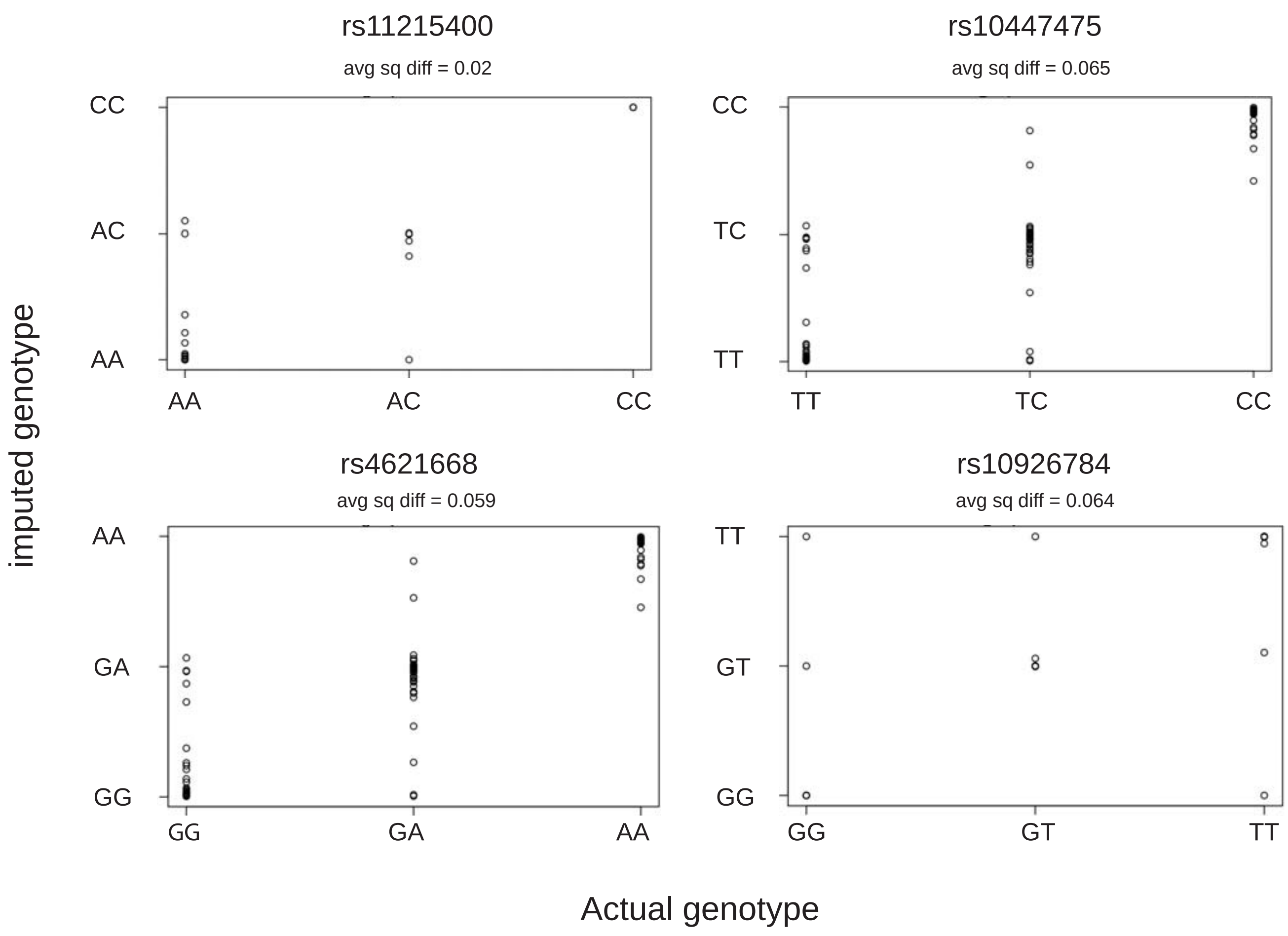

rs11215400
avg sq diff = 0.02
rs10447475
avg sq diff = 0.065
rs4621668
avg sq diff = 0.059
rs10926784
avg sq diff = 0.064
imputed genotype
Actual genotype
CC
AC
AA
TC
TT
GA
GG
GT

Figure S3

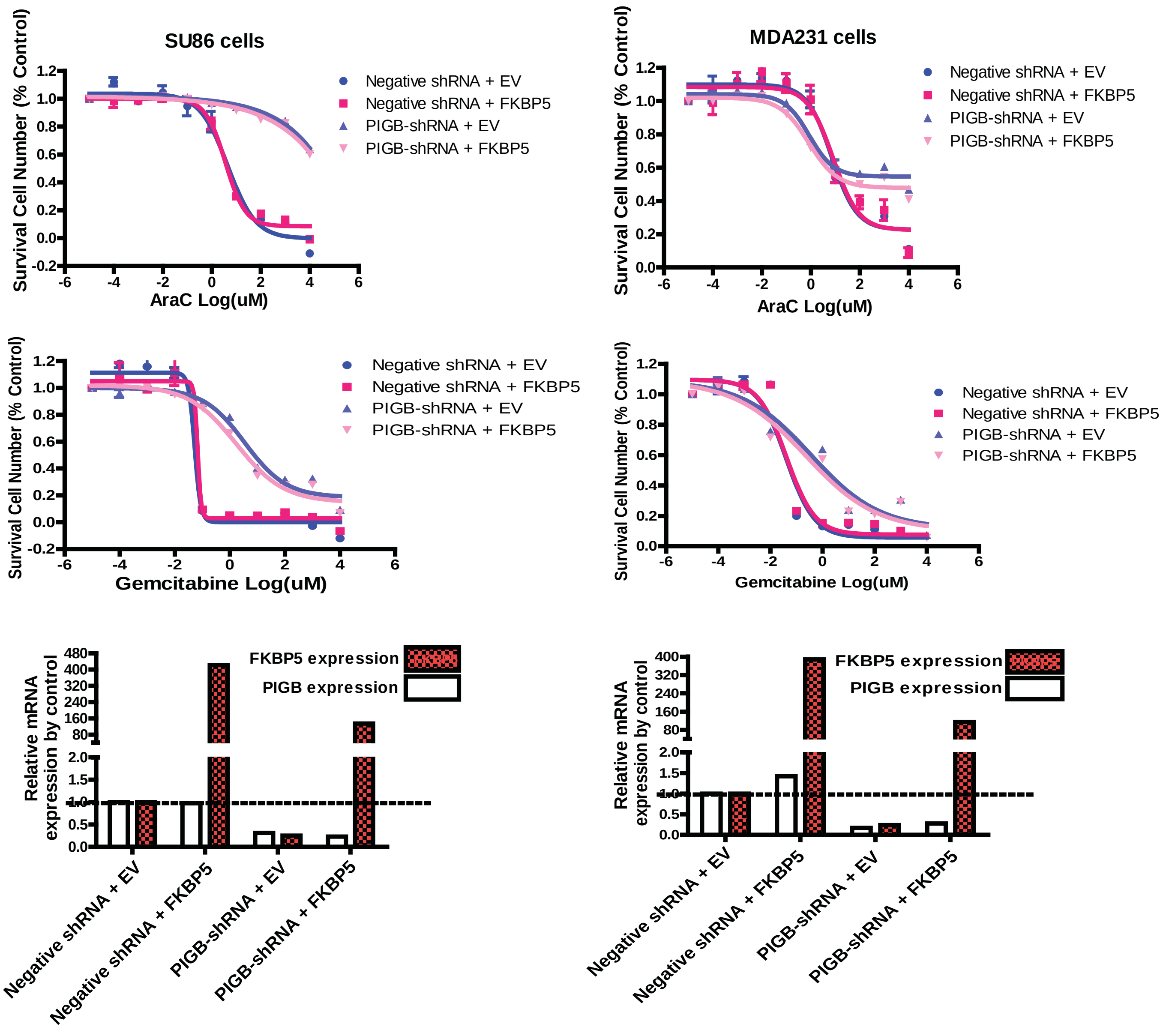
SU86 cells
Survival Cell Number (% Control)
AraC Log(uM)
Negative shRNA + EV
Negative shRNA + FKBP5
PIGB-shRNA + EV
PIGB-shRNA + FKBP5
MDA231 cells
Survival Cell Number (% Control)
AraC Log(uM)
Negative shRNA + EV
Negative shRNA + FKBP5
PIGB-shRNA + EV
PIGB-shRNA + FKBP5
Survival Cell Number (% Control)
Gemcitabine Log(uM)
Negative shRNA + EV
Negative shRNA + FKBP5
PIGB-shRNA + EV
PIGB-shRNA + FKBP5
Survival Cell Number (% Control)
Gemcitabine Log(uM)
Negative shRNA + EV
Negative shRNA + FKBP5
PIGB-shRNA + EV
PIGB-shRNA + FKBP5
Relative mRNA expression by control
FKBP5 expression
PIGB expression
Negative shRNA + EV
Negative shRNA + FKBP5
PIGB-shRNA + EV
PIGB-shRNA + FKBP5
Relative mRNA expression by control
FKBP5 expression
PIGB expression
Negative shRNA + EV
Negative shRNA + FKBP5
PIGB-shRNA + EV
PIGB-shRNA + FKBP5

Table S1A Gemcitabine

rsID	MAF	Chr	Position	R value	P value	Q value	Gene Symbol	Location
rs1598848	0.473	21	27868147	-0.391	7.08E-07	0.544	---	upstream
rs10513968	0.445	18	65434121	-0.382	1.04E-06	0.544	*DOK6*	intron
rs13269021	0.265	8	6384171	0.379	1.25E-06	0.544	*ANGPT2*	intron
rs4272382	0.131	8	8470898	-0.375	1.61E-06	0.544	*CLDN23*	flanking_5UTR
rs3775182	0.108	4	87198607	0.368	2.84E-06	0.583	*MAPK10*	intron
rs4662945	0.439	2	129932513	-0.364	3.48E-06	0.583	*LOC151121*	flanking_5UTR
rs4662945	0.439	2	129932543	-0.364	3.48E-06	0.583	*LOC151121*	flanking_5UTR
rs2275540	0.221	10	128687707	0.362	4.08E-06	0.583	*DOCK1*	intron
rs7719624	0.448	5	135405465	-0.370	4.22E-06	0.583	*TGFBI*	intron
rs2274870	0.371	9	106555035	0.362	4.33E-06	0.583	*NIPSNAP3A*	coding
rs2107331	0.453	5	135405248	-0.355	6.26E-06	0.679	*TGFBI*	intron
rs12544932	0.157	8	8467010	-0.354	6.62E-06	0.679	*CLDN23*	flanking_5UTR
rs790006	0.177	12	62892706	-0.351	8.20E-06	0.679	*FLJ32549*	intron
rs10052296	0.14	5	89242556	-0.349	9.73E-06	0.679	*CETN3*	downstream
rs2301475	0.442	1	20371755	-0.347	1.06E-05	0.679	*UBXD3*	flanking_5UTR
rs812590	0.195	12	62893749	-0.345	1.16E-05	0.679	*FLJ32549*	intron
rs5967860	0.272	23	86738879	-0.346	1.18E-05	0.679	*KLHL4*	intron
rs316838	0.093	1	240387283	-0.345	1.21E-05	0.679	*PLD5*	intron
rs17104354	0.081	14	71444537	0.344	1.22E-05	0.679	*SIPA1L1*	downstream
rs4141306	0.433	5	135403372	-0.344	1.27E-05	0.679		intron
rs11229994	0.07	11	59060178	0.343	1.31E-05	0.679	*OR4D9*	flanking_3UTR
rs2237072	0.339	5	135414699	0.344	1.34E-05	0.679	*TGFBI*	intron
rs9953044	0.398	18	65434000	-0.339	1.67E-05	0.679	*DOK6*	intron
rs12456443	0.398	18	65434430	-0.339	1.67E-05	0.679	*DOK6*	intron
rs2638094	0.105	11	6938064	-0.339	1.72E-05	0.679	*ZNF215*	flanking_3UTR
rs2290344	0.253	15	53407088	0.339	1.75E-05	0.679	*PIGB*	coding
rs6873061	0.158	5	89189083	-0.340	1.75E-05	0.679	*CETN3*	flanking_3UTR
rs790007	0.19	12	62891060	-0.339	1.77E-05	0.679	*FLJ32549*	intron
rs1558095	0.477	5	135457539	-0.339	1.79E-05	0.679	*TGFBI*	flanking_3UTR
rs10474308	0.145	5	89165454	-0.338	1.81E-05	0.679	*CETN3*	flanking_3UTR
rs10474308	0.145	5	89175680	-0.338	1.81E-05	0.679	*CETN3*	flanking_3UTR
rs10761082	0.358	9	106555990	0.338	1.83E-05	0.679	*NIPSNAP3A*	intron

rs17754749	0.056	20	22680451	0.339	1.85E-05	0.679	---	downstream
rs10504609	0.061	8	77208628	-0.337	1.91E-05	0.679	---	upstream
rs2472476	0.389	9	106571777	0.338	1.92E-05	0.679	*NIPSNAP3B*	intron
rs12193608	0.195	6	22732796	-0.337	1.96E-05	0.679	*HDGFL1*	downstream
rs695021	0.093	1	240394845	-0.336	2.08E-05	0.679	*PLD5*	intron
rs297717	0.244	20	4338249	-0.336	2.10E-05	0.679	*PRNP*	upstream
rs297715	0.244	20	4338433	-0.336	2.10E-05	0.679	*ADRA1D*	upstream
rs297718	0.244	20	4338527	-0.336	2.10E-05	0.679	*ADRA1D*	flanking_5UTR
rs327215	0.078	8	26548879	0.335	2.17E-05	0.679	*DPYSL2*	intron
rs2282791	0.48	5	135405629	0.334	2.28E-05	0.679	*TGFBI*	intron
rs4976472	0.474	5	135446616	-0.334	2.29E-05	0.679	*SMAD5*	upstream
rs2215011	0.474	5	135448034	-0.334	2.29E-05	0.679	*SMAD5*	upstream
rs4295223	0.085	4	170466749	0.335	2.31E-05	0.679	*SH3MD2*	flanking_5UTR
rs2857891	0.102	11	6919533	-0.334	2.37E-05	0.679	*ZNF215*	intron
rs2595500	0.102	11	6919741	-0.334	2.37E-05	0.679	*ZNF215*	intron
rs12252832	0.244	10	71022032	0.332	2.61E-05	0.719	*NEUROG3*	flanking_5UTR
rs6889835	0.16	5	89184631	-0.332	2.61E-05	0.719	*MEF2C*	upstream
rs3129890	0.34	6	32522251	0.332	2.67E-05	0.719	*HLA-DRA*	flanking_3UTR
rs2928445	0.267	10	58816782	-0.331	2.76E-05	0.720	*IPMK*	flanking_3UTR
rs800930	0.111	1	69899195	0.337	2.77E-05	0.720	---	intron
rs12023616	0.088	1	69893856	0.332	2.93E-05	0.731	---	intron
rs10979372	0.317	9	110299166	-0.330	2.93E-05	0.731	*ACTL7B*	flanking_3UTR
rs1989972	0.418	5	135405701	-0.331	3.00E-05	0.731	*TGFBI*	intron
rs922369	0.247	10	71020137	0.329	3.03E-05	0.731	*NEUROG3*	flanking_5UTR
rs4595128	0.201	8	8471286	-0.329	3.13E-05	0.732	*CLDN23*	flanking_5UTR
rs595074	0.302	6	98347163	-0.329	3.18E-05	0.732	*C6orf167*	flanking_5UTR
rs10820722	0.336	9	106557501	0.329	3.20E-05	0.732	*NIPSNAP3A*	intron
rs200885	0.497	20	1715896	0.329	3.33E-05	0.749	*PTPNS1*	flanking_5UTR
rs4647899	0.308	14	32363282	-0.327	3.39E-05	0.749	*AKAP6*	coding
rs12044761	0.104	1	69893703	0.337	3.44E-05	0.749	---	intron
rs4774760	0.416	15	53376504	0.326	3.63E-05	0.767	*PIGB*	upstream
rs9832418	0.096	3	128961356	0.326	3.69E-05	0.767	*MGLL*	intron
rs316833	0.142	1	240414231	-0.326	3.69E-05	0.767	*PLD5*	intron
rs7113935	0.427	11	17519298	-0.325	3.83E-05	0.783	*USH1C*	intron

rs316871	0.097	1	240409507	-0.326	4.14E-05	0.817	*PLD5*	intron
rs16979106	0.108	20	18355117	0.325	4.16E-05	0.817	*C20orf12*	intron
rs10174187	0.509	2	129917395	-0.324	4.18E-05	0.817	*LOC151121*	flanking_5UTR
rs2476522	0.337	14	101406854	0.323	4.39E-05	0.832	*PPP2R5C*	intron
rs11219196	0.16	11	122929644	0.323	4.45E-05	0.832	*SCN3B*	flanking_3UTR
rs17017879	0.09	2	3691533	-0.322	4.52E-05	0.832	*ALLC*	intron
rs2237065	0.323	5	135395119	-0.322	4.53E-05	0.832	*TGFBI*	intron
rs1929734	0.297	13	75691821	-0.322	4.77E-05	0.832	*LMO7*	flanking_3UTR
rs8024695	0.285	15	53426597	0.321	5.00E-05	0.832	*PIGB*	intron
rs2848961	0.443	18	72258874	0.325	5.04E-05	0.832	---	intron
rs3851781	0.506	17	42246300	0.322	5.07E-05	0.832	*WNT3*	intron
rs678870	0.465	18	72296065	0.320	5.12E-05	0.832	---	intron
rs7515290	0.105	1	69889413	0.320	5.15E-05	0.832	*LRRC7*	flanking_5UTR
rs2599492	0.292	5	16028021	-0.321	5.28E-05	0.832	---	downstream
rs10760365	0.491	9	126270712	-0.326	5.30E-05	0.832	*GPR144*	coding
rs453169	0.055	16	1021189	-0.319	5.46E-05	0.832	*FLJ32252*	flanking_3UTR
rs4941694	0.064	13	50688689	0.319	5.49E-05	0.832	*FLJ30707*	flanking_5UTR
rs17445240	0.096	2	3680916	-0.319	5.49E-05	0.832	*ALLC*	flanking_5UTR
rs7192	0.372	6	32519624	0.319	5.51E-05	0.832	*HLA-DRA*	coding
rs7194	0.372	6	32520458	0.319	5.51E-05	0.832	*HLA-DRA*	3UTR
rs7195	0.372	6	32520517	0.319	5.51E-05	0.832	*HLA-DRA*	3UTR
rs998818	0.044	18	42818160	0.318	5.72E-05	0.832	*TCEB3B*	flanking_5UTR
rs2848958	0.453	18	72286056	0.318	5.77E-05	0.832	*FLJ44313*	flanking_5UTR
rs661838	0.451	6	149669970	-0.318	5.78E-05	0.832	*MAP3K7IP2*	flanking_5UTR
rs4392391	0.419	3	44247970	-0.318	5.78E-05	0.832	---	downstream
rs10500812	0.424	11	15232135	-0.318	5.86E-05	0.832	*LOC387755*	flanking_3UTR
rs2554830	0.445	18	72256690	0.318	5.86E-05	0.832	---	intron
rs2590662	0.172	8	126019390	-0.318	5.92E-05	0.832	*ZNF572*	flanking_5UTR
rs4849124	0.337	2	113293373	0.318	5.95E-05	0.832	*IL1B*	flanking_3UTR
rs4849125	0.334	2	113293386	0.318	5.97E-05	0.832	*IL1A*	upstream
rs2282790	0.395	5	135391774	-0.317	6.14E-05	0.832	*TGFBI*	flanking_5UTR
rs706578	0.395	7	136949450	0.318	6.14E-05	0.832	*DGKI*	intron
rs295692	0.09	5	73740682	-0.317	6.22E-05	0.832	*ENC1*	flanking_3UTR
rs2213586	0.374	6	32521072	0.318	6.29E-05	0.832	*HLA-DRA*	flanking_3UTR

rs2213585	0.374	6	32521128	0.318	6.29E-05	0.832	*HLA-DRA*	flanking_3UTR
rs2227139	0.374	6	32521437	0.318	6.29E-05	0.832	*HLA-DRA*	flanking_3UTR
rs12466426	0.058	2	214134965	0.316	6.55E-05	0.853	*SPAG16*	intron
rs12522395	0.215	5	90246274	0.318	6.58E-05	0.853	*GPR98*	intron
rs10060641	0.215	5	90249006	0.315	6.79E-05	0.865	*MASS1*	intron
rs17506115	0.076	5	24219088	0.315	6.90E-05	0.865	*CDH10*	downstream
rs2612699	0.172	2	111302685	-0.315	7.03E-05	0.865	*ACOXL*	intron
rs790450	0.087	12	91141234	-0.315	7.06E-05	0.865	*CLLU1OS*	downstream
rs17789963	0.317	3	70600224	-0.315	7.11E-05	0.865	*MITF*	downstream
rs6134030	0.052	20	10696899	-0.314	7.13E-05	0.865	*JAG1*	upstream
rs2928472	0.173	10	58767800	-0.315	7.23E-05	0.865	*ZWINT*	upstream
rs10818977	0.453	9	126269939	0.314	7.28E-05	0.865	*GPR144*	intron
rs3805700	0.392	5	135406680	0.314	7.59E-05	0.865	*TGFBI*	intron
rs7945461	0.349	11	40536109	0.313	7.62E-05	0.865	*LRRC4C*	flanking_5UTR
rs12249393	0.174	10	58765337	-0.313	7.67E-05	0.865	*IPMK*	flanking_3UTR
rs12241948	0.174	10	58765460	-0.313	7.67E-05	0.865	*ZWINT*	upstream
rs2072174	0.172	8	186274	0.313	7.69E-05	0.865	*ZNF596*	coding
rs9820131	0.471	3	140090699	0.315	7.79E-05	0.865	*FOXL2*	flanking_3UTR
rs5951381	0.41	23	100828017	0.313	7.82E-05	0.865	*ARMCX2*	upstream
rs5951290	0.41	23	100850029	0.313	7.82E-05	0.865	*ARMCX2*	flanking_5UTR
rs5951406	0.41	23	100862192	0.313	7.82E-05	0.865	*ARMCX2*	flanking_5UTR
rs13171512	0.462	5	68000787	-0.313	7.86E-05	0.865	*PIK3R1*	flanking_3UTR
rs12054681	0.206	5	90253683	0.313	7.89E-05	0.865	*MASS1*	intron
rs17193120	0.317	16	6590859	-0.312	7.98E-05	0.868	*A2BP1*	flanking_5UTR
rs1862893	0.448	2	45399610	0.312	8.22E-05	0.882	*FLJ10379*	flanking_3UTR
rs2144986	0.352	14	101409314	0.312	8.30E-05	0.882	*PPP2R5C*	intron
rs689902	0.448	18	72248957	0.312	8.31E-05	0.882	*FLJ44313*	flanking_5UTR
rs3809714	0.327	17	69709970	0.312	8.45E-05	0.890	*RPL38*	flanking_5UTR
rs1554006	0.358	2	111320584	-0.311	8.63E-05	0.901	*ACOXL*	intron
rs10979374	0.295	9	110312797	-0.312	8.69E-05	0.901	---	upstream
rs4887981	0.07	16	77359617	0.311	8.80E-05	0.904	*WWOX*	intron
rs2281805	0.061	1	178175699	-0.311	8.87E-05	0.904	*CEP350*	flanking_5UTR
rs609487	0.451	18	72263370	0.310	8.91E-05	0.904	---	intron
rs3792900	0.387	5	135421653	-0.310	9.33E-05	0.932	*TGFBI*	intron

rs17139597	0.067	7	64369181	0.309	9.61E-05	0.932	*FLJ25037*	flanking_3UTR
rs12249388	0.177	10	58765294	-0.309	9.64E-05	0.932	*ZWINT*	upstream
rs7713001	0.459	5	67999371	-0.309	9.69E-05	0.932	*PIK3R1*	flanking_3UTR
rs12188464	0.459	5	67999705	-0.309	9.69E-05	0.932	*PIK3R1*	flanking_3UTR
rs2049451	0.243	8	47752605	0.310	9.82E-05	0.932	*CEBPD*	flanking_3UTR
rs10052015	0.214	5	90244819	0.314	9.96E-05	0.932	*GRP98*	intron
rs6718469	0.07	2	182424571	-0.308	9.97E-05	0.932	*SSFA2*	flanking_5UTR
rs7050617	0.121	23	115794924	-0.311	9.97E-05	0.932	*LOC203413*	flanking_5UTR
rs680253	0.471	18	72296415	0.308	9.99E-05	0.932	---	intron
rs861145	0.125	13	108986420	0.308	1.00E-04	0.932	*IRS2*	flanking_3UTR
rs4239061	0.113	17	1818222	-0.308	1.01E-04	0.932	*OVCA2*	upstream
rs2186397	0.259	8	108303893	0.308	1.02E-04	0.932	*ANGPT1*	flanking_3UTR
rs815440	0.36	3	55713787	-0.309	1.03E-04	0.932	*ERC2*	intron
rs12244977	0.195	10	58762688	-0.308	1.03E-04	0.932	*IPMK*	flanking_3UTR
rs12256364	0.195	10	58765694	-0.308	1.03E-04	0.932	*IPMK*	flanking_3UTR
rs10521427	0.087	23	42615327	-0.307	1.05E-04	0.933	---	upstream
rs8013347	0.23	14	100985774	-0.307	1.07E-04	0.933	*DIO3*	flanking_5UTR
rs454012	0.07	5	73748503	-0.307	1.08E-04	0.933	*ENC1*	flanking_3UTR
rs153087	0.064	16	13126755	-0.307	1.08E-04	0.933	*FLJ11151*	flanking_5UTR
rs2355111	0.279	7	11849447	0.307	1.10E-04	0.933	*TMEM106B*	flanking_5UTR
rs815437	0.448	3	55714400	-0.307	1.10E-04	0.933	*CAST1*	intron
rs533886	0.055	5	73654548	-0.307	1.10E-04	0.933	*ENC1*	flanking_3UTR
rs10059539	0.061	5	7881404	0.306	1.11E-04	0.933	*ADCY2*	3UTR
rs774015	0.192	3	139652806	0.306	1.11E-04	0.933	*FAM62C*	intron
rs1371322	0.346	8	71089232	0.306	1.12E-04	0.933	*PRDM14*	downstream
rs2545904	0.215	19	20476510	-0.308	1.12E-04	0.933	---	downstream
rs443934	0.435	9	4821076	-0.309	1.12E-04	0.933	*RCL1*	intron
rs2057121	0.298	14	32363793	-0.307	1.12E-04	0.933	*AKAP6*	intron
rs681919	0.183	6	149777293	0.306	1.13E-04	0.933	*MAP3K7IP2*	downstream
rs17037598	0.044	1	12076000	0.306	1.13E-04	0.933	*TNFRSF8*	intron
rs611862	0.085	16	1032102	-0.307	1.16E-04	0.945	*FLJ32252*	flanking_3UTR
rs815425	0.451	3	55716181	-0.305	1.17E-04	0.945	*ERC2*	intron
rs6583070	0.436	1	108558213	0.305	1.18E-04	0.945	*NBPF4*	flanking_3UTR
rs2039270	0.25	21	27533146	0.305	1.19E-04	0.945	*ADAMTS5*	upstream

rs4936323	0.137	11	114596329	-0.305	1.20E-04	0.945	*CADM1*	intron
rs11215427	0.137	11	114598648	-0.305	1.20E-04	0.945	*CADM1*	intron
rs7560244	0.25	2	74368666	0.305	1.20E-04	0.945	*SLC4A5*	intron
rs294986	0.201	5	73718795	-0.305	1.20E-04	0.945	*ENC1*	flanking_3UTR
rs1473056	0.213	4	141024853	-0.306	1.22E-04	0.948	*MAML3*	intron
rs174335	0.291	22	16396508	0.305	1.23E-04	0.948	*SLC25A18*	flanking_5UTR
rs12050587	0.45	15	53414820	0.305	1.23E-04	0.948	*PIGB*	intron
rs6102131	0.2	20	38817758	0.306	1.26E-04	0.960	*MAFB*	flanking_5UTR
rs10488368	0.177	8	180568	0.304	1.27E-04	0.960	*ZNF596*	intron
rs1402912	0.25	5	62211571	-0.304	1.27E-04	0.960	*IPO11*	flanking_3UTR
rs2334104	0.117	22	49353582	-0.309	1.28E-04	0.960	*CPT1B*	flanking_3UTR
rs12326139	0.25	18	65429505	-0.304	1.29E-04	0.960	*DOK6*	intron
rs28668016	0.365	15	53398725	0.304	1.31E-04	0.960	*PIGB*	5UTR
rs1891186	0.365	1	157895645	0.304	1.31E-04	0.960	*CRP*	flanking_3UTR
rs10211393	0.244	2	238259527	-0.303	1.31E-04	0.960	*LRRFIP1*	upstream
rs12712963	0.273	2	46148485	0.303	1.32E-04	0.960	*PRKCE*	intron
rs815422	0.447	3	55717106	-0.304	1.33E-04	0.960	*ERC2*	intron
rs1990201	0.381	5	135423102	-0.303	1.33E-04	0.960	*TGFBI*	intron
rs7992272	0.401	13	92895509	-0.303	1.34E-04	0.960	*GPC6*	intron
rs6506895	0.357	18	27025841	0.307	1.34E-04	0.960	*DSG1*	upstream
rs4332201	0.076	9	112528505	-0.304	1.35E-04	0.960	*MUSK*	intron
rs1565214	0.425	3	44309303	-0.308	1.35E-04	0.960	*C3orf23*	upstream
rs12050885	0.38	15	53345916	0.304	1.37E-04	0.966	*RAB27A*	intron
rs2237066	0.358	5	135395502	-0.302	1.38E-04	0.966	*TGFBI*	intron
rs9953470	0.235	18	39461657	-0.302	1.38E-04	0.966	*SYT4*	flanking_5UTR
rs4891761	0.433	18	65448824	-0.303	1.39E-04	0.966	*DOK6*	intron
rs4976933	0.151	8	43815274	0.302	1.40E-04	0.966	*POTE8*	flanking_3UTR
rs11807353	0.105	1	51387611	-0.302	1.40E-04	0.966	---	downstream
rs2824330	0.224	21	17779779	0.302	1.44E-04	0.981	*CXADR*	upstream
rs11077017	0.297	16	6224888	-0.302	1.45E-04	0.981	*ALG1*	downstream
rs9809107	0.419	3	44257938	-0.301	1.46E-04	0.981	*C3orf23*	flanking_5UTR
rs891878	0.439	2	1971631	-0.301	1.47E-04	0.981	*MYT1L*	intron
rs6564074	0.294	16	83343751	-0.301	1.48E-04	0.981	*USP10*	intron
rs967285	0.422	3	44250280	-0.301	1.48E-04	0.981	---	downstream

rs12548772	0.11	8	32847268	-0.301	1.49E-04	0.981	---	downstream
rs4259484	0.09	9	112525823	-0.301	1.50E-04	0.981	*MUSK*	intron
rs17331811	0.055	8	77224205	-0.301	1.50E-04	0.981	---	upstream
rs7585834	0.189	2	78994696	-0.304	1.51E-04	0.981	*REG3G*	upstream
rs9514827	0.247	13	107717404	-0.301	1.52E-04	0.981	*TNFSF13B*	flanking_5UTR
rs11636687	0.421	15	53392444	0.302	1.53E-04	0.981	*PIGB*	flanking_5UTR
rs17334720	0.049	8	77364377	-0.300	1.53E-04	0.981	*ZFHX4*	upstream
rs9637784	0.081	5	24208009	0.300	1.53E-04	0.981	*LOC439936*	flanking_3UTR
rs9918503	0.397	7	38133649	-0.302	1.55E-04	0.981	*STARD3NL*	upstream
rs11766960	0.096	7	96421268	-0.300	1.55E-04	0.981	*DLX5*	flanking_3UTR
rs2302038	0.387	5	135424917	-0.300	1.57E-04	0.981	*TGFBI*	intron
rs2253804	0.183	17	45710559	-0.300	1.58E-04	0.981	*TMEM92*	intron
rs2794498	0.166	1	157902740	0.300	1.58E-04	0.981	*CRP*	flanking_3UTR
rs4883998	0.214	13	74928511	0.305	1.62E-04	0.981	*TBC1D4*	intron
rs2414409	0.448	15	53419009	0.299	1.62E-04	0.981	*PIGB*	intron
rs7237341	0.41	18	65436664	0.299	1.62E-04	0.981	*DOK6*	intron
rs12435942	0.148	14	43981436	0.302	1.62E-04	0.981	*C14orf155*	flanking_3UTR
rs4790850	0.326	17	1817265	0.299	1.63E-04	0.981	*DPH1*	upstream
rs17123485	0.091	11	119297383	-0.301	1.64E-04	0.981	*TRIM29*	flanking_3UTR
rs17744505	0.176	8	169693	0.301	1.65E-04	0.981		upstream
rs17009792	0.081	2	74342831	-0.299	1.68E-04	0.981	*SLC4A5*	coding
rs188404	0.343	5	73712195	-0.298	1.70E-04	0.981	*ENC1*	flanking_3UTR
rs5937118	0.067	23	68042811	-0.298	1.71E-04	0.981	*EFNB1*	flanking_3UTR
rs17041533	0.044	2	51374304	0.299	1.72E-04	0.981	---	upstream
rs8140044	0.157	22	38436928	-0.298	1.72E-04	0.981	*CACNA1I*	flanking_3UTR
rs13204330	0.064	6	10702246	0.298	1.72E-04	0.981	*GCNT2*	intron
rs9466910	0.064	6	10707265	0.298	1.72E-04	0.981	*GCNT2*	intron
rs9466912	0.064	6	10707306	0.298	1.72E-04	0.981	*GCNT2*	intron
rs2217881	0.08	1	240385094	-0.301	1.72E-04	0.981	*PLD5*	intron
rs10820723	0.208	9	106559270	0.304	1.74E-04	0.981	*NIPSNAP3A*	intron
rs10867403	0.221	9	71159900	0.298	1.74E-04	0.981	*C9orf61*	intron
rs7754768	0.465	6	32528157	0.300	1.76E-04	0.981	*HLA-DRA*	flanking_3UTR
rs2824333	0.227	21	17795347	0.298	1.76E-04	0.981	*CXADR*	flanking_5UTR
rs1122269	0.198	20	59677231	-0.298	1.78E-04	0.981	*CDH4*	intron

rs9362642	0.405	6	65025162	0.305	1.79E-04	0.981	*PHF3*	flanking_3UTR
rs1687349	0.221	5	73713465	-0.297	1.80E-04	0.981	*ENC1*	flanking_3UTR
rs2928464	0.164	10	58765948	-0.301	1.83E-04	0.981	*IPMK*	flanking_3UTR
rs4440156	0.363	16	53351602	-0.297	1.84E-04	0.981	*IRX5*	flanking_5UTR
rs17331643	0.053	8	77216734	-0.298	1.86E-04	0.981	*ZFHX4*	flanking_5UTR
rs2301478	0.235	1	20374561	-0.297	1.87E-04	0.981	*UBXD3*	flanking_5UTR
rs1901314	0.453	4	187523301	0.297	1.88E-04	0.981	*F11*	flanking_3UTR
rs12156009	0.334	8	11322629	0.297	1.88E-04	0.981	*C8orf13*	intron
rs1060433	0.398	5	135413599	0.296	1.90E-04	0.981	*TGFBI*	intron
rs692062	0.256	15	53560064	0.296	1.90E-04	0.981	*DYX1C1*	intron
rs4638930	0.321	3	70611523	-0.300	1.90E-04	0.981	*LOC401072*	flanking_3UTR
rs3748615	0.124	1	51526310	-0.298	1.91E-04	0.981	*RNF11*	flanking_3UTR
rs10484461	0.093	6	113834023	0.296	1.91E-04	0.981	---	downstream
rs11747093	0.218	5	159794088	-0.296	1.91E-04	0.981	*PTTG1*	downstream
rs6754459	0.398	2	3685298	-0.296	1.92E-04	0.981	*ALLC*	intron
rs5967919	0.09	23	87286806	-0.296	1.92E-04	0.981	*CPXCR1*	upstream
rs7262615	0.07	20	37344709	0.296	1.94E-04	0.981	*DHX35*	downstream
rs1818553	0.398	3	189849946	-0.297	1.95E-04	0.981	*LPP*	intron
rs1509865	0.119	1	211907610	-0.300	1.95E-04	0.981	---	upstream
rs12198900	0.205	6	47793614	-0.297	1.96E-04	0.981	*GPR115*	intron
rs242367	0.198	21	27787843	-0.296	1.97E-04	0.981	---	upstream
rs811484	0.09	6	121541279	-0.296	1.97E-04	0.981	*C6orf170*	intron
rs2831023	0.488	21	27879104	0.296	1.97E-04	0.981	*C21orf94*	flanking_5UTR
rs2372479	0.413	2	215650735	0.296	1.98E-04	0.981	*ABCA12*	intron
rs6694641	0.433	1	108557105	0.295	1.99E-04	0.981	*NBPF4*	flanking_3UTR
rs12776457	0.065	10	7436610	-0.298	2.00E-04	0.981	*SFMBT2*	intron
rs6486477	0.231	12	128543407	0.296	2.01E-04	0.981	*KIAA1944*	intron
rs4950059	0.474	1	95323717	0.296	2.01E-04	0.981	*ALG14*	flanking_5UTR
rs7719125	0.477	5	176621772	-0.295	2.02E-04	0.981	*NSD1*	intron
rs12485563	0.427	3	142888398	-0.296	2.02E-04	0.981	*RNF7*	flanking_5UTR
rs12402910	0.064	1	219562110	0.296	2.02E-04	0.981	*C1orf140*	flanking_3UTR
rs9820110	0.311	3	70552648	-0.295	2.03E-04	0.981	*LOC401072*	flanking_3UTR
rs7867387	0.116	9	81306761	0.295	2.04E-04	0.981	*TLE4*	flanking_5UTR
rs4935699	0.169	10	58762331	-0.295	2.04E-04	0.981	*IPMK*	flanking_3UTR

rs10509072	0.169	10	58765573	-0.295	2.04E-04	0.981	*ZWINT*	upstream
rs6722230	0.082	2	74342570	-0.297	2.06E-04	0.981	*SLC4A5*	intron
rs2547319	0.358	19	56574961	-0.295	2.06E-04	0.981	*LIM2*	flanking_3UTR
rs7132165	0.233	12	128543562	0.295	2.07E-04	0.981	*KIAA1944*	intron
rs4390610	0.07	16	77971170	0.294	2.11E-04	0.981	*WWOX*	flanking_3UTR
rs7102246	0.474	11	17519214	-0.294	2.11E-04	0.981	*USH1C*	intron
rs951614	0.34	12	59824993	0.294	2.12E-04	0.981	*FAM19A2*	flanking_3UTR
rs7094380	0.206	10	11763263	-0.294	2.13E-04	0.981	*ECHDC3*	flanking_5UTR
rs16871811	0.096	5	73746540	-0.295	2.13E-04	0.981	---	downstream
rs4511535	0.201	16	5506393	0.294	2.15E-04	0.981	*FAM86A*	flanking_5UTR
rs2305776	0.411	19	13890269	0.297	2.17E-04	0.981	*CC2D1A*	intron
rs9555490	0.218	13	108100521	-0.296	2.17E-04	0.981	*MYR8*	intron
rs3134012	0.227	8	94824402	-0.294	2.18E-04	0.981	*RBM12B*	flanking_5UTR
rs2241728	0.221	7	131765600	0.294	2.19E-04	0.981	*PLXNA4B*	flanking_3UTR
rs4507794	0.126	8	1619932	-0.295	2.19E-04	0.981	*DLGAP2*	intron
rs6462812	0.41	7	38144732	0.293	2.21E-04	0.981	*STARD3NL*	flanking_5UTR
rs12654722	0.079	5	24233989	0.294	2.21E-04	0.981	*LOC439936*	flanking_3UTR
rs4789636	0.483	17	69710434	-0.293	2.22E-04	0.981	*RPL38*	flanking_5UTR
rs9929156	0.259	16	6589736	0.293	2.23E-04	0.981	*A2BP1*	flanking_5UTR
rs11604322	0.292	11	82527700	-0.294	2.23E-04	0.981	*PCF11*	flanking_5UTR
rs12563154	0.471	1	95334775	0.293	2.24E-04	0.981	*TMEM56*	flanking_5UTR
rs917303	0.314	5	135445931	0.293	2.24E-04	0.981	*TGFBI*	flanking_3UTR
rs8005034	0.093	14	25540300	-0.298	2.25E-04	0.981	---	downstream
rs2172820	0.259	8	15370039	-0.293	2.25E-04	0.981	*TUSC3*	flanking_5UTR
rs533813	0.523	18	22436251	-0.294	2.25E-04	0.981	*KCTD1*	intron
rs10802112	0.249	1	119892957	0.294	2.25E-04	0.981	*HSD3B1*	flanking_3UTR
rs12566799	0.171	1	18995620	0.295	2.26E-04	0.981	*TAS1R2*	flanking_3UTR
rs10259079	0.108	7	8393063	0.293	2.26E-04	0.981	*NXPH1*	upstream
rs6604882	0.459	1	222307562	-0.293	2.28E-04	0.981	*FBXO28*	flanking_5UTR
rs2575675	0.221	4	87154914	0.295	2.28E-04	0.981	*MAPK10*	flanking_3UTR
rs9314442	0.189	8	181226	0.293	2.29E-04	0.981	*ZNF596*	intron
rs1425419	0.166	4	124785414	0.293	2.31E-04	0.981	*SPRY1*	flanking_3UTR
rs4849134	0.334	2	111331486	-0.293	2.32E-04	0.981	*ACOXL*	intron
rs2685419	0.375	8	27862326	-0.292	2.33E-04	0.981	*SCARA5*	intron

rs4489930	0.09	14	25535906	-0.292	2.33E-04	0.981	*NOVA1*	flanking_3UTR
rs10780297	0.474	9	71125397	-0.292	2.34E-04	0.981	*C9orf61*	flanking_5UTR
rs250490	0.34	5	16028251	-0.292	2.34E-04	0.981	*FBXL7*	flanking_3UTR
rs12401403	0.326	1	18736089	0.292	2.34E-04	0.981	*KLHDC7A*	flanking_3UTR
rs3867426	0.067	5	133491168	-0.293	2.34E-04	0.981	*TCF7*	intron
rs2318064	0.235	6	124231122	0.292	2.34E-04	0.981	*TCBA1*	intron
rs10820726	0.211	9	106572138	0.293	2.35E-04	0.981	*NIPSNAP3B*	intron
rs813068	0.09	6	121561278	-0.292	2.35E-04	0.981	*C6orf170*	intron
rs3008770	0.154	10	58793223	-0.292	2.36E-04	0.981	*ZWINT*	upstream
rs9549172	0.433	13	39792002	-0.293	2.36E-04	0.981	*FOXO1A*	flanking_3UTR
rs4924564	0.395	15	39697085	-0.292	2.37E-04	0.981	---	upstream
rs7183960	0.451	15	53409987	0.292	2.37E-04	0.981	*PIGB*	intron
rs1880130	0.433	2	215655497	0.292	2.38E-04	0.981	*ABCA12*	intron
rs7881987	0.079	23	87868970	-0.293	2.39E-04	0.981	*CPXCR1*	upstream
rs3776820	0.148	5	149623588	-0.292	2.39E-04	0.981	*CAMK2A*	intron
rs6569179	0.087	6	121572611	-0.292	2.39E-04	0.981	*C6orf170*	intron
rs4527742	0.087	6	121603783	-0.292	2.39E-04	0.981	*C6orf170*	intron
rs4585603	0.087	6	121642237	-0.292	2.39E-04	0.981	*C6orf170*	intron
rs6933489	0.087	6	121655240	-0.292	2.39E-04	0.981	*C6orf170*	intron
rs6569194	0.087	6	121660856	-0.292	2.39E-04	0.981	*C6orf170*	intron
rs10499105	0.087	6	121667531	-0.292	2.39E-04	0.981	*C6orf170*	intron
rs4663780	0.105	2	238243104	-0.292	2.39E-04	0.981	*LRRFIP1*	flanking_5UTR
rs1375668	0.366	8	6384278	-0.292	2.39E-04	0.981	*ANGPT2*	intron
rs12640749	0.336	4	174503801	0.293	2.40E-04	0.981	*HMGB2*	flanking_5UTR
rs1533298	0.352	2	111332775	-0.292	2.41E-04	0.981	*ACOXL*	intron
rs13019266	0.355	2	111321113	-0.292	2.41E-04	0.981	*ACOXL*	intron
rs7842329	0.374	8	63940907	0.293	2.41E-04	0.981	*FAM77D*	intron
rs672605	0.116	6	150762132	0.292	2.41E-04	0.981	*C6orf71*	3UTR
rs9821268	0.413	3	44278128	-0.292	2.43E-04	0.983	*C3orf23*	upstream
rs327224	0.087	8	26538939	0.292	2.44E-04	0.983	*DPYSL2*	intron
rs9302665	0.349	16	53898068	-0.292	2.44E-04	0.983	*IRX6*	upstream
rs6919522	0.358	6	44577483	-0.291	2.47E-04	0.987	*CDC5L*	flanking_3UTR
rs7279549	0.27	21	33838483	-0.291	2.48E-04	0.987	*SON*	intron
rs4562502	0.287	23	68951103	-0.292	2.48E-04	0.987	*EDA*	intron

rs3008784	0.14	10	58809955	-0.291	2.49E-04	0.987	*IPMK*	flanking_3UTR
rs13296612	0.262	9	3748657	-0.291	2.49E-04	0.987	*RFX3*	upstream
rs12259466	0.166	10	58741893	-0.291	2.50E-04	0.988	*ZWINT*	upstream
rs10817087	0.099	9	112530597	-0.292	2.53E-04	0.994	*MUSK*	intron
rs11995740	0.374	8	63958194	0.292	2.53E-04	0.994	*FAM77D*	intron
rs7204799	0.093	16	10912050	-0.291	2.55E-04	0.994	*CIITA*	intron
rs7512953	0.474	1	95326882	0.291	2.55E-04	0.994	*TMEM56*	upstream
rs9344936	0.433	6	65029561	0.292	2.56E-04	0.994	---	downstream
rs17623941	0.137	4	166270716	0.292	2.56E-04	0.994	---	upstream
rs12562498	0.474	1	95327753	0.291	2.58E-04	0.994	*TMEM56*	upstream
rs4243311	0.419	18	65444059	-0.290	2.60E-04	0.994	*DOK6*	intron
rs9654535	0.327	5	100661966	-0.291	2.60E-04	0.994	---	upstream
rs7009676	0.049	8	92218924	0.290	2.61E-04	0.994	*OTUD6B*	flanking_3UTR
rs17041165	0.23	3	4695206	-0.290	2.61E-04	0.994	*ITPR1*	intron
rs1416483	0.337	6	150770255	-0.290	2.62E-04	0.994	*PLEKHG1*	upstream
rs151222	0.142	16	20581993	0.290	2.62E-04	0.994	*ACSM1*	intron
rs8005039	0.314	14	32339539	-0.290	2.63E-04	0.994	*AKAP6*	intron
rs1565215	0.418	3	44309103	-0.291	2.64E-04	0.994	*C3orf23*	upstream
rs12505935	0.067	4	37400385	0.290	2.64E-04	0.994	*PGM2*	flanking_5UTR
rs12557613	0.078	23	115779418	-0.290	2.64E-04	0.994	*LOC203413*	flanking_5UTR
rs8035855	0.427	15	39865253	-0.291	2.65E-04	0.996	*MAPKBP1*	intron
rs2842169	0.067	10	128320703	-0.290	2.66E-04	0.998	*C10orf90*	flanking_5UTR
rs221378	0.206	23	27515921	0.290	2.68E-04	1.000	*FLJ32867*	flanking_5UTR
rs11587664	0.081	1	86486934	0.290	2.71E-04	1.000	*COL24A1*	flanking_5UTR
rs895581	0.247	1	157904611	0.295	2.71E-04	1.000	*CRP*	flanking_3UTR
rs1552314	0.328	8	93173981	-0.289	2.72E-04	1.000	*RUNX1T1*	intron
rs1226498	0.485	6	47065584	0.289	2.72E-04	1.000	*GPR116*	upstream
rs1153942	0.117	1	222037368	0.290	2.74E-04	1.000	*TP53BP2*	intron
rs7461004	0.198	8	93158004	-0.289	2.74E-04	1.000	*RUNX1T1*	intron
rs2072239	0.14	5	135420406	0.289	2.74E-04	1.000	*TGFBI*	intron
rs17605893	0.061	4	37411627	0.289	2.75E-04	1.000	---	upstream
rs2928426	0.127	10	58801359	-0.292	2.76E-04	1.000	*IPMK*	flanking_3UTR
rs13144325	0.381	4	82653764	0.289	2.76E-04	1.000	*RASGEF1B*	flanking_5UTR
rs52308	0.302	5	90817903	0.289	2.76E-04	1.000	*ARRDC3*	flanking_5UTR

rs4974292	0.355	3	70599373	-0.289	2.77E-04	1.000	---	downstream
rs1971863	0.167	1	157905555	0.290	2.78E-04	1.000	*CRP*	downstream
rs818704	0.084	9	115188137	-0.289	2.79E-04	1.000	*ALAD*	flanking_3UTR
rs2029495	0.183	4	71358655	-0.289	2.80E-04	1.000	*MUC7*	flanking_5UTR
rs3827410	0.346	22	45258351	-0.289	2.80E-04	1.000	*CELSR1*	intron
rs10204598	0.096	2	38205473	0.289	2.81E-04	1.000	*MGC34824*	flanking_5UTR
rs327227	0.064	8	26539395	0.289	2.84E-04	1.000	*DPYSL2*	intron
rs2180038	0.477	6	65029844	0.289	2.84E-04	1.000	---	upstream
rs17062850	0.237	3	60332422	-0.291	2.85E-04	1.000	*FHIT*	intron
rs10200191	0.422	2	62350674	-0.288	2.88E-04	1.000	---	downstream
rs1616725	0.485	18	22438804	-0.288	2.88E-04	1.000	*KCTD1*	intron
rs7928356	0.259	11	96252552	-0.288	2.88E-04	1.000	---	upstream
rs316823	0.093	1	240422651	-0.288	2.88E-04	1.000	*PLD5*	intron
rs402098	0.093	1	240430321	-0.288	2.88E-04	1.000	*PLD5*	intron
rs11870163	0.337	17	14154605	0.288	2.88E-04	1.000	*HS3ST3B1*	intron
rs9511784	0.206	13	24866976	-0.288	2.89E-04	1.000	*NUPL1*	flanking_3UTR
rs1888012	0.302	23	138598616	0.288	2.90E-04	1.000	*ATP11C*	flanking_3UTR
rs1494219	0.416	3	22370283	-0.288	2.91E-04	1.000	*ZNF659*	upstream
rs2958692	0.206	8	134203855	-0.288	2.91E-04	1.000	*TG*	intron
rs7049592	0.091	23	87858882	-0.290	2.92E-04	1.000	*CPXCR1*	flanking_5UTR
rs13404551	0.396	2	136438465	0.291	2.94E-04	1.000	*DARS*	intron
rs7106302	0.471	11	17520065	-0.288	2.95E-04	1.000	*USH1C*	intron
rs685272	0.378	11	63993609	0.288	2.96E-04	1.000	*SLC22A11*	upstream
rs400838	0.25	1	240464782	-0.288	2.97E-04	1.000	*PLD5*	intron
rs6894815	0.419	5	135423763	-0.288	2.97E-04	1.000	*TGFBI*	intron
rs2021421	0.541	18	22434295	-0.289	2.97E-04	1.000	*KCTD1*	intron
rs4245508	0.131	6	121643326	-0.288	2.97E-04	1.000	*C6orf170*	intron
rs4682949	0.398	3	44220159	-0.287	3.01E-04	1.000	*C3orf23*	flanking_5UTR
rs7757305	0.5	6	65092239	0.287	3.02E-04	1.000	---	downstream
rs4849135	0.332	2	111331550	-0.289	3.03E-04	1.000	*ACOXL*	intron
rs8133982	0.16	21	33940340	-0.287	3.04E-04	1.000	*ITSN1*	intron
rs17675810	0.064	5	172656880	0.287	3.04E-04	1.000	*STC2*	downstream
rs1966265	0.299	5	176449237	-0.287	3.05E-04	1.000	*FGFR4*	coding
rs1966265	0.299	5	176454062	-0.287	3.05E-04	1.000	*FGFR4*	coding

rs1275282	0.201	10	58736257	-0.287	3.05E-04	1.000	*ZWINT*	upstream
rs1580578	0.18	4	71353399	-0.287	3.05E-04	1.000	*MUC7*	flanking_5UTR
rs4694065	0.18	4	71356062	-0.287	3.05E-04	1.000	*PROL1*	downstream
rs2002866	0.201	7	133340918	0.287	3.08E-04	1.000	*EXOC4*	intron
rs7785128	0.23	7	133335531	0.287	3.08E-04	1.000	*SEC8L1*	intron
rs10831663	0.435	11	11658545	0.290	3.08E-04	1.000	*GALNTL4*	flanking_5UTR
rs10268602	0.105	7	8392321	0.287	3.11E-04	1.000	*ICA1*	upstream
rs705349	0.273	7	90511244	-0.287	3.13E-04	1.000	*PFTK1*	intron
rs1495310	0.358	11	40538079	0.287	3.14E-04	1.000	*LRRC4C*	flanking_5UTR
rs9268980	0.302	6	32543101	0.289	3.15E-04	1.000	*HLA-DRA*	flanking_3UTR
rs7986860	0.193	13	80952378	-0.287	3.17E-04	1.000	*SPRY2*	flanking_5UTR
rs9269043	0.299	6	32546576	0.291	3.19E-04	1.000	*HLA-DRA*	flanking_3UTR
rs3213313	0.064	19	48758842	-0.286	3.19E-04	1.000	*XRCC1*	intron
rs3768597	0.105	1	181464636	0.286	3.20E-04	1.000	*LAMC2*	intron
rs1377045	0.299	9	28158157	-0.286	3.20E-04	1.000	*LINGO2*	intron
rs12473388	0.378	2	45402809	0.286	3.22E-04	1.000	*SRBD1*	downstream
rs17732411	0.073	13	89705804	-0.286	3.24E-04	1.000	*GPC5*	flanking_5UTR
rs10155331	0.335	4	11147099	0.288	3.24E-04	1.000	*HS3ST1*	flanking_5UTR
rs6760606	0.215	2	105102269	-0.286	3.25E-04	1.000	*GPR45*	upstream
rs7567068	0.215	2	105108563	-0.286	3.25E-04	1.000	*MRPS9*	flanking_3UTR
rs12616980	0.215	2	105113227	-0.286	3.25E-04	1.000	*MRPS9*	downstream
rs7018025	0.374	8	63941141	0.287	3.26E-04	1.000	*FAM77D*	intron
rs616315	0.336	1	55051429	0.287	3.26E-04	1.000	*C1orf177*	intron
rs1277213	0.439	1	109161025	-0.287	3.27E-04	1.000	*STXBP3*	flanking_3UTR
rs8084	0.39	6	32519013	0.286	3.29E-04	1.000	*HLA-DRA*	coding
rs9828929	0.294	3	29019230	-0.286	3.29E-04	1.000	*RBMS3*	flanking_5UTR
rs6876085	0.206	5	113486888	-0.286	3.29E-04	1.000	---	upstream
rs4140875	0.404	17	67900113	-0.286	3.29E-04	1.000	*SLC39A11*	flanking_3UTR
rs7915278	0.202	10	58754215	-0.287	3.30E-04	1.000	*IPMK*	flanking_3UTR
rs13252280	0.19	8	93157327	-0.289	3.30E-04	1.000	*RUNX1T1*	intron
rs9918379	0.09	6	113836873	0.286	3.30E-04	1.000	*MARCKS*	flanking_5UTR
rs6505037	0.221	17	51511242	-0.287	3.30E-04	1.000	*PCTP*	downstream
rs6880837	0.413	5	135424568	-0.285	3.32E-04	1.000	*TGFBI*	intron
rs4703054	0.279	5	98576215	-0.285	3.33E-04	1.000	*CHD1*	flanking_5UTR

rs1121306	0.279	5	98580084	-0.285	3.33E-04	1.000	*CHD1*	flanking_5UTR
rs10095936	0.37	8	63953433	0.288	3.34E-04	1.000	*FAM77D*	intron
rs4536103	0.439	10	71002210	-0.285	3.35E-04	1.000	*NEUROG3*	coding
rs7676073	0.064	4	37400567	0.285	3.35E-04	1.000	---	upstream
rs6860369	0.404	5	135419225	0.285	3.36E-04	1.000	*TGFBI*	intron
rs408858	0.203	13	59271902	-0.292	3.36E-04	1.000	*DIAPH3*	intron
rs10084092	0.267	18	65430704	-0.285	3.39E-04	1.000	*DOK6*	intron
rs6689733	0.102	1	12602767	-0.285	3.40E-04	1.000	*DHRS3*	flanking_5UTR
rs898988	0.183	8	142676731	0.290	3.40E-04	1.000	---	downstream
rs12946355	0.377	17	12705608	0.286	3.41E-04	1.000	*KIAA0672*	intron
rs376621	0.188	1	240412444	-0.287	3.42E-04	1.000	*PLD5*	intron
rs11088256	0.263	21	33865413	-0.286	3.44E-04	1.000	*SON*	intron
rs898987	0.198	8	142676785	0.285	3.45E-04	1.000	*FLJ43860*	flanking_5UTR
rs2006882	0.366	7	36837726	-0.285	3.45E-04	1.000	*AOAH*	upstream
rs3749249	0.128	3	198929608	-0.285	3.47E-04	1.000	*FYTTD1*	flanking_5UTR
rs10875069	0.265	1	97566363	0.285	3.48E-04	1.000	*DPYD*	intron
rs2950613	0.163	2	140629853	0.285	3.49E-04	1.000	---	downstream
rs575156	0.438	10	29538215	-0.286	3.49E-04	1.000	*LYZL1*	flanking_5UTR
rs609098	0.494	6	65143469	0.284	3.51E-04	1.000	*PHF3*	flanking_3UTR
rs3934191	0.366	1	19899159	0.284	3.51E-04	1.000	*TMCO4*	intron
rs2545902	0.231	19	20475270	-0.287	3.52E-04	1.000	---	downstream
rs9815785	0.395	3	189858646	-0.284	3.53E-04	1.000	*LPP*	intron
rs1275277	0.203	10	58739792	-0.284	3.54E-04	1.000	*IPMK*	flanking_3UTR
rs2607072	0.259	8	95324533	-0.284	3.54E-04	1.000	*CDH17*	upstream
rs17241824	0.113	18	69255018	-0.284	3.55E-04	1.000	*NETO1*	flanking_5UTR
rs10513969	0.218	18	65437954	-0.284	3.56E-04	1.000	*DOK6*	intron
rs10877489	0.419	12	59640726	0.284	3.56E-04	1.000	*FAM19A2*	flanking_3UTR
rs693535	0.442	18	22448482	-0.284	3.57E-04	1.000	*KCTD1*	intron
rs2954369	0.451	5	113497460	-0.284	3.57E-04	1.000	*YTHDC2*	downstream
rs6496376	0.267	15	85322907	0.284	3.57E-04	1.000	*AGBL1*	downstream
rs3133615	0.334	8	95691039	0.284	3.58E-04	1.000	*KIAA1429*	upstream
rs221356	0.213	23	27514441	0.285	3.59E-04	1.000	---	upstream
rs12254418	0.36	10	3775290	0.284	3.59E-04	1.000	*KLF6*	flanking_3UTR
rs4596804	0.395	23	68305854	-0.285	3.60E-04	1.000	*PJA1*	flanking_5UTR

rs9559278	0.163	13	107643003	-0.284	3.60E-04	1.000	*LIG4*	flanking_3UTR
rs12430757	0.25	13	89694885	-0.287	3.61E-04	1.000	*GPC5*	flanking_5UTR
rs930869	0.253	8	95329118	-0.284	3.62E-04	1.000	*GEM*	flanking_3UTR
rs7171557	0.299	15	77761077	-0.284	3.62E-04	1.000	*MTHFS*	flanking_3UTR
rs380005	0.27	1	240466936	-0.284	3.62E-04	1.000	*PLD5*	intron
rs16979132	0.108	20	18360208	0.284	3.62E-04	1.000	*C20orf12*	intron
rs1371237	0.397	4	44275069	0.286	3.62E-04	1.000	*YIPF7*	flanking_3UTR
rs17491111	0.07	2	38206991	0.284	3.63E-04	1.000	*MGC34824*	flanking_5UTR
rs17104037	0.157	5	145297151	-0.284	3.63E-04	1.000	*SH3RF2*	intron
rs13286706	0.23	9	3747758	-0.284	3.64E-04	1.000	*GLIS3*	flanking_3UTR
rs2928465	0.166	10	58765982	-0.284	3.65E-04	1.000	*ZWINT*	upstream
rs12252820	0.116	10	71021996	0.284	3.65E-04	1.000	*NEUROG3*	flanking_5UTR
rs12252986	0.116	10	71022062	0.284	3.65E-04	1.000	*NEUROG3*	flanking_5UTR
rs4391246	0.337	6	65010434	0.283	3.67E-04	1.000	*PHF3*	flanking_3UTR
rs10042825	0.418	5	135423983	-0.285	3.67E-04	1.000	*TGFBI*	intron
rs10510741	0.445	3	44239198	-0.283	3.68E-04	1.000	*C3orf23*	flanking_5UTR
rs234993	0.14	16	20555145	0.283	3.70E-04	1.000	*ACSM1*	intron
rs6882884	0.421	5	100664741	-0.284	3.72E-04	1.000	*ST8SIA4*	flanking_5UTR
rs2210382	0.398	10	34173005	0.283	3.72E-04	1.000	*PARD3*	flanking_3UTR
rs10200712	0.424	2	62351406	-0.284	3.74E-04	1.000	*B3GNT1*	flanking_3UTR
rs2211105	0.331	6	150768933	-0.286	3.74E-04	1.000	*C6orf71*	flanking_3UTR
rs1053889	0.34	9	4850980	-0.283	3.75E-04	1.000	*RCL1*	3UTR
rs3808256	0.051	7	90425787	-0.287	3.76E-04	1.000	*PFTK1*	intron
rs874083	0.154	5	149631118	-0.283	3.77E-04	1.000	*CAMK2A*	intron
rs6872201	0.468	5	176653372	-0.283	3.77E-04	1.000	*NSD1*	intron
rs9993029	0.287	4	3443437	-0.286	3.77E-04	1.000	*DOK7*	intron
rs11600380	0.058	11	116175392	0.284	3.80E-04	1.000	*APOA4*	downstream
rs4811058	0.401	20	48444908	0.283	3.80E-04	1.000	*LOC284751*	flanking_3UTR
rs17817199	0.328	16	6223229	-0.283	3.81E-04	1.000	*A2BP1*	intron
rs11063838	0.091	12	584837	-0.284	3.82E-04	1.000	*NINJ2*	intron
rs10837433	0.346	11	40516291	0.283	3.85E-04	1.000	---	downstream
rs1459583	0.422	5	100661637	-0.282	3.86E-04	1.000	---	upstream
rs10069443	0.422	5	100665166	-0.282	3.86E-04	1.000	*ST8SIA4*	flanking_5UTR
rs17816810	0.056	20	22675849	0.283	3.88E-04	1.000	*FOXA2*	flanking_5UTR

rs309164	0.432	2	136408295	0.284	3.90E-04	1.000	*DARS*	intron
rs2724989	0.235	8	4432981	-0.292	3.91E-04	1.000	---	downstream
rs668781	0.195	11	119321722	-0.285	3.93E-04	1.000	---	downstream
rs1572430	0.09	10	86189487	-0.282	3.94E-04	1.000	*KIAA1128*	intron
rs9298155	0.312	8	71090312	0.284	3.94E-04	1.000	*PRDM14*	downstream
rs6567192	0.346	18	56769040	0.282	3.95E-04	1.000	*CDH20*	flanking_5UTR
rs10094991	0.256	8	108317068	0.289	3.95E-04	1.000	*ANGPT1*	downstream
rs17303140	0.135	8	14391356	0.284	3.97E-04	1.000	*SGCZ*	intron
rs17795251	0.087	16	25576150	-0.282	3.98E-04	1.000	*HS3ST4*	upstream
rs17200180	0.087	16	25577051	-0.282	3.98E-04	1.000	*ZNF694*	flanking_5UTR
rs10732702	0.212	9	73454240	-0.282	3.99E-04	1.000		downstream
rs3008767	0.15	10	58781752	-0.284	3.99E-04	1.000	*IPMK*	flanking_3UTR
rs341508	0.198	13	59318107	-0.282	3.99E-04	1.000	*DIAPH3*	intron
rs3812067	0.325	5	179641760	0.283	4.00E-04	1.000	*MAPK9*	flanking_5UTR
rs4664396	0.41	2	161609896	-0.282	4.00E-04	1.000	*TANK*	upstream
rs1923864	0.48	13	103626083	0.283	4.00E-04	1.000	*DAOA*	upstream
rs1678022	0.349	14	101395490	0.282	4.00E-04	1.000	*PPP2R5C*	intron
rs17169786	0.363	5	135429577	0.282	4.03E-04	1.000	*TGFBI*	downstream
rs12195286	0.131	6	15473956	0.282	4.04E-04	1.000	*JARID2*	intron
rs2172006	0.186	4	71346235	-0.281	4.05E-04	1.000	*MUC7*	flanking_5UTR
rs17264406	0.079	23	13764240	0.282	4.06E-04	1.000	*GEMIN8*	downstream
rs12317773	0.167	12	128532989	0.282	4.06E-04	1.000	*KIAA1944*	intron
rs7174876	0.423	15	53406853	0.284	4.06E-04	1.000	*PIGB*	intron
rs705352	0.273	7	90536300	-0.281	4.08E-04	1.000	*PFTK1*	intron
rs2638100	0.216	11	6926252	-0.282	4.11E-04	1.000	*ZNF215*	intron
rs11964097	0.21	6	110388801	-0.284	4.11E-04	1.000	*FIG4*	downstream
rs2190626	0.285	17	14143843	0.281	4.12E-04	1.000	*HS3ST3B1*	flanking_5UTR
rs17742788	0.06	15	20414703	-0.285	4.12E-04	1.000	*TUBGCP5*	intron
rs413430	0.11	5	73746180	-0.281	4.14E-04	1.000	*ENC1*	flanking_3UTR
rs467147	0.11	5	73747302	-0.281	4.14E-04	1.000	*ENC1*	downstream
rs717512	0.14	6	21395972	0.281	4.15E-04	1.000	*CDKAL1*	flanking_3UTR
rs1471451	0.404	16	7151976	0.282	4.16E-04	1.000	*A2BP1*	intron
rs12923969	0.262	16	5495137	0.281	4.16E-04	1.000	---	downstream
rs10810197	0.061	9	14627704	0.281	4.16E-04	1.000	*ZDHHC21*	intron

rs1421331	0.459	8	30940357	-0.281	4.17E-04	1.000	*PURG*	flanking_3UTR
rs17564220	0.181	13	107686012	0.282	4.18E-04	1.000	*TNFSF13B*	upstream
rs1532370	0.123	12	79737766	-0.282	4.20E-04	1.000	*LIN7A*	intron
rs12187932	0.482	5	176575757	-0.282	4.20E-04	1.000	*NSD1*	intron
rs1410989	0.215	9	73455355	-0.281	4.20E-04	1.000	*TMEM2*	flanking_3UTR
rs7570283	0.225	2	136164177	-0.282	4.22E-04	1.000	*R3HDM1*	intron
rs1955011	0.45	11	81358036	-0.282	4.23E-04	1.000	*MGC33846*	flanking_3UTR
rs6615747	0.271	23	94960949	-0.282	4.23E-04	1.000	*DIAPH2*	flanking_5UTR
rs341521	0.247	13	59297046	-0.281	4.24E-04	1.000	*DIAPH3*	intron
rs3782169	0.12	12	79729821	-0.281	4.24E-04	1.000	*LIN7A*	intron
rs7826606	0.373	8	63944117	0.283	4.28E-04	1.000	*FAM77D*	intron
rs2007366	0.177	4	147274094	0.280	4.28E-04	1.000	*LSM6*	flanking_5UTR
rs7695415	0.177	4	147275685	0.280	4.28E-04	1.000	*LSM6*	upstream
rs6559467	0.477	9	71122977	0.280	4.29E-04	1.000	*TJP2*	downstream
rs2074811	0.406	5	135419846	0.282	4.30E-04	1.000	*TGFBI*	intron
rs4783057	0.291	16	83343173	-0.280	4.30E-04	1.000	*USP10*	intron
rs4572	0.358	5	135427351	0.280	4.31E-04	1.000	*TGFBI*	3UTR
rs16893187	0.218	5	24198300	0.280	4.31E-04	1.000	*CDH10*	downstream
rs13279826	0.213	8	47747730	0.285	4.31E-04	1.000	---	downstream
rs9344917	0.34	6	65013927	0.280	4.32E-04	1.000	*PHF3*	flanking_3UTR
rs4391105	0.162	4	57082463	0.282	4.32E-04	1.000	*ARL9*	intron
rs12025316	0.221	1	20367328	-0.280	4.32E-04	1.000	---	intron
rs6458390	0.247	6	44577007	0.280	4.32E-04	1.000	*CDC5L*	flanking_3UTR
rs11742191	0.387	5	135438762	0.280	4.34E-04	1.000	*TGFBI*	downstream
rs12597524	0.483	16	7153100	0.280	4.35E-04	1.000	*A2BP1*	upstream
rs12599645	0.483	16	7153393	0.280	4.35E-04	1.000	*A2BP1*	intron
rs2858162	0.395	23	100760488	0.280	4.36E-04	1.000	*ARMCX6*	flanking_5UTR
rs2858165	0.395	23	100761839	0.280	4.36E-04	1.000	*ARMCX6*	flanking_5UTR
rs2858167	0.395	23	100764000	0.280	4.36E-04	1.000	*ARMCX3*	flanking_5UTR
rs2106491	0.128	5	135376275	0.280	4.37E-04	1.000	*TGFBI*	flanking_5UTR
rs8019453	0.235	14	100980531	-0.280	4.39E-04	1.000	*DIO3*	flanking_5UTR
rs340222	0.298	13	59365374	-0.283	4.40E-04	1.000	*DIAPH3*	intron
rs2301472	0.219	1	20371501	-0.281	4.41E-04	1.000	*UBXD3*	flanking_5UTR
rs2125209	0.419	21	27873218	0.280	4.41E-04	1.000	*C21orf94*	flanking_5UTR

rs5936510	0.209	23	68973474	0.280	4.41E-04	1.000	*EDA*	intron
rs5980665	0.209	23	68974292	0.280	4.41E-04	1.000	*EDA*	intron
rs5936511	0.209	23	68980699	0.280	4.41E-04	1.000	*EDA*	intron
rs12983461	0.311	19	56572153	-0.280	4.42E-04	1.000	---	upstream
rs11060375	0.163	12	128536569	0.280	4.43E-04	1.000	*TMEM132D*	intron
rs427498	0.087	1	240424078	-0.280	4.43E-04	1.000	*PLD5*	intron
rs13047599	0.257	21	33848130	-0.284	4.44E-04	1.000	*SON*	coding
rs9638671	0.247	7	11519688	-0.280	4.45E-04	1.000	---	intron
rs16971976	0.384	15	39777218	-0.280	4.46E-04	1.000	*MAPKBP1*	flanking_5UTR
rs6496378	0.27	15	85323047	0.280	4.46E-04	1.000	*AGBL1*	downstream
rs607824	0.441	11	119260105	0.281	4.47E-04	1.000	*PVRL1*	flanking_5UTR
rs1648250	0.413	13	102550905	0.279	4.48E-04	1.000	*DAOA*	upstream
rs2455879	0.294	3	107180839	-0.279	4.49E-04	1.000	*CBLB*	flanking_5UTR
rs3017883	0.352	11	88915360	0.279	4.50E-04	1.000	*NOX4*	upstream
rs13361398	0.044	5	157453665	0.280	4.51E-04	1.000	*ENTH*	flanking_5UTR
rs7207536	0.398	17	67897746	-0.279	4.54E-04	1.000	*SLC39A11*	downstream
rs1451882	0.131	8	1070771	-0.279	4.55E-04	1.000	*DLGAP2*	flanking_5UTR
rs7881950	0.172	23	17110420	0.279	4.55E-04	1.000	*REPS2*	flanking_3UTR
rs7054725	0.172	23	17115557	0.279	4.55E-04	1.000	*REPS2*	flanking_3UTR
rs7881950	0.172	23	17118466	0.279	4.55E-04	1.000	*REPS2*	flanking_3UTR
rs7049284	0.172	23	17121599	0.279	4.55E-04	1.000	*REPS2*	downstream
rs7054725	0.172	23	17122359	0.279	4.55E-04	1.000	*REPS2*	flanking_3UTR
rs1903187	0.419	5	100658557	-0.279	4.57E-04	1.000	*ST8SIA4*	flanking_5UTR
rs12624625	0.07	20	56137806	0.280	4.57E-04	1.000	*C20orf85*	upstream
rs1016134	0.116	17	9867806	0.279	4.57E-04	1.000	*GAS7*	intron
rs1993397	0.383	12	112653773	-0.280	4.59E-04	1.000	*RBM19*	flanking_3UTR
rs9852733	0.451	3	44252343	-0.279	4.59E-04	1.000	*C3orf23*	flanking_5UTR
rs417407	0.243	5	71880756	0.280	4.60E-04	1.000	*ZNF366*	upstream
rs605142	0.52	12	128711711	-0.280	4.60E-04	1.000	*KIAA1944*	intron
rs11777472	0.201	8	142673630	0.279	4.61E-04	1.000	*TSNARE1*	downstream
rs1353613	0.113	3	131596772	-0.279	4.62E-04	1.000	*FLJ35880*	upstream
rs6128270	0.052	20	56115212	0.279	4.62E-04	1.000	*C20orf85*	upstream
rs9952054	0.218	18	6912019	-0.279	4.63E-04	1.000	*ARHGAP28*	flanking_3UTR
rs543642	0.125	6	74373314	0.279	4.64E-04	1.000	*SLC17A5*	intron

rs636166	0.125	6	74373717	0.279	4.64E-04	1.000	*SLC17A5*	intron
rs476838	0.125	6	74374563	0.279	4.64E-04	1.000	*SLC17A5*	intron
rs473078	0.125	6	74374986	0.279	4.64E-04	1.000	*SLC17A5*	intron
rs1691341	0.125	6	74376024	0.279	4.64E-04	1.000	*SLC17A5*	intron
rs472294	0.125	6	74410896	0.279	4.64E-04	1.000	*SLC17A5*	coding
rs1487222	0.459	5	113468791	-0.279	4.64E-04	1.000	*KCNN2*	flanking_5UTR
rs4710476	0.349	6	65019532	0.279	4.67E-04	1.000	*PHF3*	flanking_3UTR
rs12640355	0.307	4	5672721	0.282	4.67E-04	1.000	*EVC2*	intron
rs12057866	0.244	1	60045110	-0.279	4.68E-04	1.000	*HOOK1*	flanking_5UTR
rs9297952	0.285	8	96135054	0.279	4.69E-04	1.000	*C8orf38*	downstream
rs11107763	0.073	12	76916089	-0.279	4.69E-04	1.000	*NAV3*	intron
rs7064603	0.094	23	87833289	-0.279	4.70E-04	1.000	*CPXCR1*	flanking_5UTR
rs10827735	0.053	10	19976155	0.280	4.72E-04	1.000	---	downstream
rs10146815	0.154	14	43975942	0.278	4.73E-04	1.000	*C14orf155*	downstream
rs2355106	0.227	7	11840583	0.278	4.75E-04	1.000	---	upstream
rs10806410	0.465	6	65041677	0.279	4.76E-04	1.000	*PHF3*	flanking_3UTR
rs2241522	0.243	15	39915026	-0.279	4.79E-04	1.000	*PLA2G4B*	intron
rs7662264	0.183	4	71372365	-0.278	4.80E-04	1.000	*MUC7*	flanking_5UTR
rs17744517	0.182	8	172340	0.280	4.80E-04	1.000	*ZNF596*	flanking_5UTR
rs16856409	0.205	1	178966177	-0.279	4.82E-04	1.000	*XPR1*	intron
rs2861667	0.334	5	165444179	0.278	4.83E-04	1.000	*WWC1*	flanking_5UTR
rs2861667	0.334	5	165444270	0.278	4.83E-04	1.000	*WWC1*	flanking_5UTR
rs5014831	0.212	5	158974807	0.278	4.83E-04	1.000	*IL12B*	flanking_5UTR
rs731365	0.416	11	24290633	0.278	4.83E-04	1.000	*LUZP2*	flanking_5UTR
rs2290015	0.044	4	56453750	0.278	4.83E-04	1.000	*EXOC1*	intron
rs17262485	0.304	3	192541	0.282	4.83E-04	1.000	*CHL1*	upstream
rs12688186	0.209	23	68997861	0.280	4.84E-04	1.000	*EDA*	intron
rs879484	0.125	1	20601799	-0.278	4.85E-04	1.000	*FLJ32784*	flanking_3UTR
rs1363837	0.302	5	90731038	0.278	4.85E-04	1.000	*ARRDC3*	flanking_5UTR
rs13273012	0.202	8	93161513	-0.279	4.86E-04	1.000	*RUNX1T1*	intron
rs10847890	0.218	12	128551768	0.278	4.86E-04	1.000	*KIAA1944*	intron
rs4669	0.407	5	135420325	0.278	4.87E-04	1.000	*TGFBI*	coding
rs6871571	0.407	5	135421096	0.278	4.87E-04	1.000	*TGFBI*	intron
rs335231	0.352	8	18673635	-0.278	4.87E-04	1.000	*PSD3*	intron

rs219737	0.372	21	36781530	-0.281	4.88E-04	1.000	*CLDN14*	flanking_5UTR
rs7205632	0.169	16	83353903	-0.278	4.88E-04	1.000	*USP10*	intron
rs1221754	0.349	2	154081012	-0.278	4.89E-04	1.000	*RPRM*	flanking_5UTR
rs763933	0.383	14	57389591	-0.279	4.89E-04	1.000	*C14orf37*	flanking_3UTR
rs4079357	0.128	6	74371962	0.278	4.92E-04	1.000	*SLC17A5*	intron
rs1159918	0.398	4	100462032	-0.278	4.92E-04	1.000	*ADH1B*	flanking_5UTR
rs9296571	0.215	6	47761857	-0.279	4.93E-04	1.000	*GPR115*	intron
rs17634127	0.249	7	11529388	-0.278	4.94E-04	1.000	*PHF14*	flanking_3UTR
rs2037679	0.186	1	202349561	0.277	4.97E-04	1.000	*SOX13*	intron
rs9822272	0.19	3	10617523	0.278	4.98E-04	1.000	*ATP2B2*	flanking_5UTR
rs12038869	0.205	1	186735729	0.278	5.02E-04	1.000	*C1orf99*	flanking_3UTR
rs13418767	0.186	2	3682705	-0.277	5.02E-04	1.000	*ALLC*	flanking_5UTR
rs763416	0.105	6	107745473	-0.277	5.03E-04	1.000	*PDSS2*	intron
rs2889710	0.407	1	31454967	-0.282	5.03E-04	1.000	*FAM77C*	intron
rs7950019	0.311	11	18235488	-0.277	5.04E-04	1.000	*SAA2*	flanking_5UTR
rs7950019	0.311	11	18238492	-0.277	5.04E-04	1.000	*SAA2*	flanking_5UTR
rs6951842	0.076	7	3683418	0.277	5.04E-04	1.000	*SDK1*	intron
rs9697204	0.102	9	81294921	0.277	5.04E-04	1.000	*TLE4*	upstream
rs12337953	0.102	9	81299075	0.277	5.04E-04	1.000	*TLE4*	flanking_5UTR
rs17082505	0.102	9	81306088	0.277	5.04E-04	1.000	*TLE4*	upstream
rs1884009	0.371	14	85057358	0.278	5.04E-04	1.000	*FLRT2*	flanking_5UTR
rs10136865	0.282	14	48469198	0.277	5.05E-04	1.000	*RPS29*	flanking_3UTR
rs4656435	0.453	1	163441998	0.280	5.05E-04	1.000	*LMX1A*	intron
rs9268878	0.304	6	32539270	0.278	5.07E-04	1.000	*HLA-DRB5*	downstream
rs3767996	0.272	1	60054300	-0.278	5.08E-04	1.000	*HOOK1*	intron
rs6128386	0.465	20	56626955	0.277	5.08E-04	1.000	*APCDD1L*	upstream
rs7316874	0.36	12	112657941	0.277	5.11E-04	1.000	*RBM19*	downstream
rs3760501	0.109	17	9872847	0.279	5.11E-04	1.000	*GAS7*	intron
rs1480113	0.503	8	63901480	0.277	5.11E-04	1.000	*FAM77D*	intron
rs12932661	0.081	16	25566220	-0.277	5.11E-04	1.000	*ZNF694*	flanking_5UTR
rs244708	0.482	5	176522191	-0.278	5.12E-04	1.000	*NSD1*	intron
rs1370785	0.096	12	102656495	-0.277	5.12E-04	1.000	*STAB2*	intron
rs1922956	0.32	6	65056280	0.277	5.12E-04	1.000	*PHF3*	flanking_3UTR
rs12615624	0.433	2	136438073	0.277	5.13E-04	1.000	*DARS*	intron

rs6794298	0.368	3	67718196	0.278	5.13E-04	1.000	*SUCLG2*	intron
rs13255574	0.148	8	6386085	0.277	5.15E-04	1.000	*ANGPT2*	intron
rs4976912	0.268	8	43892463	0.278	5.15E-04	1.000	*POTE8*	flanking_3UTR
rs12603825	0.305	17	1620155	-0.277	5.16E-04	1.000	*SERPINF1*	intron
rs12056089	0.378	7	137050360	-0.277	5.16E-04	1.000	*DGKI*	intron
rs12773782	0.343	10	25404121	-0.282	5.17E-04	1.000	*GPR158*	upstream
rs686646	0.128	11	125564632	-0.277	5.18E-04	1.000	*RPUSD4*	flanking_3UTR
rs681664	0.128	11	125566048	-0.277	5.18E-04	1.000	*RPUSD4*	downstream
rs28044	0.453	5	58624026	0.277	5.18E-04	1.000	*PDE4D*	intron
rs3008769	0.183	10	58792835	-0.277	5.18E-04	1.000	*IPMK*	flanking_3UTR
rs2160420	0.265	15	34841212	-0.278	5.19E-04	1.000	*C15orf41*	intron
rs1906768	0.206	1	186756837	0.276	5.21E-04	1.000	*C1orf99*	flanking_3UTR
rs12022365	0.206	1	186775841	0.276	5.21E-04	1.000	*C1orf99*	flanking_3UTR
rs9268856	0.312	6	32537697	0.280	5.21E-04	1.000	*HLA-DRB5*	downstream
rs999803	0.366	4	104915369	-0.276	5.22E-04	1.000	*TACR3*	upstream
rs4606685	0.267	15	85323347	0.276	5.22E-04	1.000	*AGBL1*	downstream
rs9544385	0.134	13	76316442	0.276	5.24E-04	1.000	*KCTD12*	flanking_3UTR
rs1226500	0.485	6	47069911	0.281	5.27E-04	1.000	*GPR110*	flanking_3UTR
rs359294	0.154	10	17373316	0.276	5.27E-04	1.000	*ST8SIA6*	flanking_3UTR
rs359297	0.154	10	17373686	0.276	5.27E-04	1.000	*ST8SIA6*	downstream
rs10491859	0.137	9	1500299	-0.277	5.32E-04	1.000	---	upstream
rs328838	0.447	9	7693280	-0.277	5.33E-04	1.000	*C9orf123*	flanking_3UTR
rs6697388	0.154	1	202034445	0.276	5.35E-04	1.000	*ZC3H11A*	flanking_5UTR
rs7511676	0.154	1	202036833	0.276	5.35E-04	1.000	*ZC3H11A*	5UTR
rs1226485	0.465	6	47104177	-0.277	5.36E-04	1.000	*GPR110*	intron
rs1889436	0.113	6	125295231	-0.276	5.36E-04	1.000	*TCBA1*	downstream
rs6888165	0.253	5	84096779	0.278	5.37E-04	1.000	*EDIL3*	flanking_5UTR
rs2699332	0.279	13	102582789	0.276	5.39E-04	1.000	*SLC10A2*	flanking_5UTR
rs2298294	0.227	1	149967969	0.276	5.39E-04	1.000	*TNRC4*	flanking_5UTR
rs619476	0.166	1	55036139	0.276	5.39E-04	1.000	*TTC22*	intron
rs6001655	0.238	22	38433880	-0.276	5.40E-04	1.000	*CACNA1I*	downstream
rs7045305	0.233	9	94639646	-0.276	5.40E-04	1.000	*ANKRD19*	intron
rs9586032	0.233	13	102460525	0.276	5.41E-04	1.000	*SLC10A2*	flanking_3UTR
rs17281018	0.084	23	10341675	-0.276	5.42E-04	1.000	*CLCN4*	downstream

rs709339	0.378	3	55716934	-0.276	5.42E-04	1.000	*ERC2*	intron
rs7536235	0.262	1	154848158	0.276	5.44E-04	1.000	*HAPLN2*	flanking_5UTR
rs718261	0.485	9	108195921	-0.276	5.44E-04	1.000	---	downstream
rs2961911	0.323	5	159794153	-0.275	5.44E-04	1.000	*PTTG1*	flanking_3UTR
rs12818390	0.462	12	31723811	-0.275	5.45E-04	1.000	*LOC196394*	intron
rs10859789	0.068	12	76920104	-0.278	5.45E-04	1.000	*NAV3*	intron
rs5936752	0.23	23	68935388	0.275	5.46E-04	1.000	*EDA*	intron
rs13236072	0.401	7	101335506	-0.275	5.46E-04	1.000	*CUTL1*	intron
rs9520823	0.221	13	107671565	-0.275	5.46E-04	1.000	*C13orf6*	intron
rs3772325	0.395	3	1288419	0.276	5.47E-04	1.000	*CNTN6*	intron
rs11109583	0.067	12	97643486	0.275	5.48E-04	1.000	*APAF1*	intron
rs5951376	0.381	23	100802127	0.275	5.49E-04	1.000	*ARMCX2*	flanking_5UTR
rs6523526	0.381	23	100804566	0.275	5.49E-04	1.000	*ARMCX2*	flanking_5UTR
rs233992	0.336	4	104876519	-0.276	5.49E-04	1.000	*TACR3*	flanking_5UTR
rs4378828	0.203	2	105111965	-0.275	5.50E-04	1.000	---	downstream
rs673162	0.099	19	37063862	0.275	5.51E-04	1.000	*ZNF507*	flanking_5UTR
rs2113428	0.488	7	137043227	0.275	5.51E-04	1.000	*DGKI*	intron
rs10739905	0.206	9	106545943	0.275	5.53E-04	1.000	*NIPSNAP3A*	flanking_5UTR
rs7113061	0.342	11	40483594	0.276	5.55E-04	1.000	*LRRC4C*	flanking_5UTR
rs12048929	0.418	1	186731693	0.276	5.57E-04	1.000	*C1orf99*	flanking_3UTR
rs12498964	0.055	4	56374997	0.275	5.60E-04	1.000	---	upstream
rs7219745	0.18	17	76431503	-0.275	5.61E-04	1.000	*raptor*	intron
rs4910407	0.518	11	11657270	-0.276	5.66E-04	1.000	*GALNTL4*	flanking_5UTR
rs7766843	0.305	6	32538707	0.275	5.66E-04	1.000	*HLA-DRB5*	downstream
rs2157338	0.305	6	32547301	0.275	5.66E-04	1.000	*HLA-DRA*	flanking_3UTR
rs2187823	0.305	6	32547486	0.275	5.66E-04	1.000	*HLA-DRA*	flanking_3UTR
rs3850318	0.378	23	100825548	0.275	5.67E-04	1.000	*ARMCX2*	upstream
rs16909	0.468	2	45405034	0.275	5.68E-04	1.000	*FLJ10379*	flanking_3UTR
rs11687349	0.497	2	3695371	0.275	5.68E-04	1.000	*ALLC*	intron
rs7343048	0.198	18	18048293	0.277	5.70E-04	1.000	*GATA6*	flanking_3UTR
rs2454333	0.386	8	89961762	0.275	5.70E-04	1.000	*RIPK2*	upstream
rs7906235	0.177	10	58755852	-0.274	5.71E-04	1.000	*IPMK*	flanking_3UTR
rs671108	0.163	1	55039385	0.274	5.72E-04	1.000	*TTC22*	coding
rs10141935	0.305	14	28076505	-0.274	5.72E-04	1.000	*FOXG1B*	flanking_5UTR

rs4127784	0.305	14	28077046	-0.274	5.72E-04	1.000	*FOXG1*	upstream
rs2877929	0.305	14	28080063	-0.274	5.72E-04	1.000	*FOXG1*	upstream
rs12878206	0.305	14	28089805	-0.274	5.72E-04	1.000	*FOXG1*	upstream
rs11079738	0.448	17	42259048	0.274	5.72E-04	1.000	*WNT3*	flanking_5UTR
rs12610504	0.304	19	35756431	-0.275	5.74E-04	1.000	*ZNF536*	flanking_3UTR
rs2832489	0.285	21	30221341	-0.281	5.75E-04	1.000	*GRIK1*	intron
rs11185305	0.384	1	108565427	0.274	5.75E-04	1.000	*NBPF4*	flanking_3UTR
rs1741895	0.126	6	74372828	0.275	5.75E-04	1.000	*SLC17A5*	intron
rs7997280	0.216	13	59254739	-0.275	5.75E-04	1.000	*DIAPH3*	intron
rs1519103	0.45	2	66531385	0.275	5.76E-04	1.000	*MEIS1*	intron
rs7452947	0.128	6	121626069	-0.274	5.76E-04	1.000	*C6orf170*	intron
rs7312905	0.055	12	87986976	0.274	5.78E-04	1.000	---	downstream
rs1809180	0.174	15	99515505	-0.274	5.81E-04	1.000	*CHSY1*	flanking_3UTR
rs11869751	0.108	17	30742290	-0.274	5.82E-04	1.000	*FLJ34922*	flanking_5UTR
rs10797465	0.36	1	231700442	-0.274	5.82E-04	1.000	---	intron
rs3757108	0.128	6	74421899	0.274	5.83E-04	1.000	*CD109*	upstream
rs4417316	0.16	11	116157511	0.274	5.84E-04	1.000	*ZNF259*	intron
rs2152560	0.302	1	108563535	0.277	5.86E-04	1.000	*SLC25A24*	upstream
rs3770748	0.276	2	37449029	-0.276	5.86E-04	1.000	*QPCT*	intron
rs991858	0.335	7	78917432	0.276	5.87E-04	1.000	*MAGI2*	intron
rs6452189	0.206	5	24198866	0.274	5.88E-04	1.000	*LOC439936*	flanking_3UTR
rs10914424	0.372	1	31752885	0.274	5.89E-04	1.000	*TINAGL1*	upstream
rs2341635	0.41	19	51162274	-0.274	5.91E-04	1.000	*NOVA2*	intron
rs631090	0.227	1	22858990	0.274	5.91E-04	1.000	*C1QB*	intron
rs12965392	0.195	18	69244519	0.274	5.92E-04	1.000	*NETO1*	flanking_5UTR
rs12966046	0.195	18	69244710	0.274	5.92E-04	1.000	*NETO1*	upstream
rs11639680	0.195	16	5500905	0.274	5.92E-04	1.000	*FAM86A*	flanking_5UTR
rs12360697	0.126	11	5361781	-0.275	5.92E-04	1.000	*OR51M1*	flanking_5UTR
rs2008801	0.128	11	114513385	-0.274	5.93E-04	1.000	*IGSF4*	flanking_3UTR
rs2507905	0.128	11	114513815	-0.274	5.93E-04	1.000	*FAM55B*	downstream
rs2008801	0.128	11	114521517	-0.274	5.93E-04	1.000	*IGSF4*	flanking_3UTR
rs2274001	0.299	1	19337360	0.274	5.94E-04	1.000	*RBAF600*	intron
rs11100001	0.241	4	156599423	0.274	5.94E-04	1.000	---	exon
rs390956	0.273	1	240465026	-0.274	5.94E-04	1.000	*PLD5*	intron

rs11126079	0.442	2	66529871	0.274	5.96E-04	1.000	*MEIS1*	intron
rs6810842	0.378	4	100462468	-0.274	5.96E-04	1.000	*ADH1B*	flanking_5UTR
rs4631605	0.078	23	86698393	-0.274	5.97E-04	1.000	*KLHL4*	intron
rs7164080	0.351	15	31852090	-0.274	5.98E-04	1.000	*RYR3*	intron
rs601223	0.177	6	150762264	0.274	5.98E-04	1.000	*C6orf71*	3UTR
rs10426665	0.368	19	22220649	0.274	5.98E-04	1.000	---	upstream
rs2033290	0.161	2	141374348	0.274	5.98E-04	1.000	*LRP1B*	intron
rs11751092	0.078	6	150660443	-0.274	5.98E-04	1.000	*PPP1R14C*	flanking_3UTR
rs10877556	0.33	12	59817800	0.274	6.01E-04	1.000	*FAM19A2*	downstream
rs10505143	0.137	8	111349752	-0.273	6.03E-04	1.000	*KCNV1*	flanking_5UTR
rs2670854	0.233	17	38339209	0.273	6.03E-04	1.000	*AARSD1*	flanking_3UTR
rs16899418	0.067	5	24112943	0.273	6.03E-04	1.000	*LOC439936*	flanking_3UTR
rs16893121	0.067	5	24137801	0.273	6.03E-04	1.000	*LOC439936*	flanking_3UTR
rs13248	0.14	9	15454286	-0.273	6.04E-04	1.000	*PSIP1*	3UTR
rs856541	0.302	7	46722666	-0.273	6.07E-04	1.000	---	upstream
rs856554	0.302	7	46726654	-0.273	6.07E-04	1.000	*TNS3*	flanking_3UTR
rs3809263	0.366	12	643717	0.273	6.08E-04	1.000	*NINJ2*	flanking_5UTR
rs515145	0.334	6	11720961	-0.273	6.09E-04	1.000	*C6orf105*	flanking_3UTR
rs902891	0.235	12	128544470	0.273	6.10E-04	1.000	*TMEM132D*	intron
rs2065719	0.241	10	116910080	-0.273	6.11E-04	1.000	*ATRNL1*	intron
rs9406333	0.291	6	169460019	0.275	6.13E-04	1.000	*THBS2*	flanking_5UTR
rs12387561	0.16	23	17116894	0.273	6.14E-04	1.000	*REPS2*	downstream
rs4970810	0.453	1	109075133	-0.273	6.14E-04	1.000	*FNDC7*	intron
rs2291101	0.523	3	1399718	0.273	6.14E-04	1.000	*CNTN6*	CDS
rs6527782	0.161	23	17121441	0.274	6.14E-04	1.000	*REPS2*	downstream
rs7747074	0.323	6	65047051	0.273	6.18E-04	1.000	*PHF3*	flanking_3UTR
rs593493	0.323	6	65114770	0.273	6.18E-04	1.000	*PHF3*	flanking_3UTR
rs6727037	0.288	2	153000420	0.273	6.18E-04	1.000	*FMNL2*	intron
rs1956623	0.328	14	25532217	0.273	6.18E-04	1.000	---	downstream
rs6808448	0.451	3	44218611	-0.273	6.19E-04	1.000	*C3orf23*	flanking_5UTR
rs1504035	0.311	3	6110268	0.275	6.20E-04	1.000	*GRM7*	flanking_5UTR
rs325120	0.177	6	147860161	-0.273	6.20E-04	1.000	*SAMD5*	upstream
rs9369741	0.227	6	47810893	-0.273	6.22E-04	1.000	*OPN5*	upstream
rs9268862	0.307	6	32538145	0.274	6.22E-04	1.000	*HLA-DRA*	flanking_3UTR

rs9268977	0.307	6	32542917	0.274	6.22E-04	1.000	*HLA-DRA*	flanking_3UTR
rs1194588	0.306	1	152579856	-0.274	6.22E-04	1.000	*ATP8B2*	intron
rs9268832	0.427	6	32535767	0.273	6.23E-04	1.000	*HLA-DRA*	flanking_3UTR
rs1560625	0.48	2	1972031	0.273	6.23E-04	1.000	*MYT1L*	intron
rs12460783	0.067	19	14277651	-0.274	6.24E-04	1.000	---	downstream
rs300969	0.319	5	119906825	0.274	6.24E-04	1.000	*LOC51334*	intron
rs1608234	0.355	2	79156926	0.273	6.25E-04	1.000	*REG1B*	flanking_3UTR
rs6641783	0.32	23	3455846	0.277	6.26E-04	1.000	*MXRA5*	upstream
rs9868890	0.265	3	141336334	-0.273	6.27E-04	1.000	*CLSTN2*	intron
rs4538699	0.169	6	22733783	-0.273	6.28E-04	1.000	*HDGFL1*	flanking_3UTR
rs2341634	0.407	19	51162145	-0.272	6.30E-04	1.000	*NOVA2*	intron
rs7825422	0.1	8	55276794	0.279	6.30E-04	1.000	---	upstream
rs808241	0.339	14	57434616	-0.273	6.30E-04	1.000	*C14orf37*	flanking_3UTR
rs10244081	0.363	7	11860113	0.272	6.31E-04	1.000	*TMEM106B*	flanking_5UTR
rs12536311	0.477	7	38531706	0.272	6.31E-04	1.000	*AMPH*	intron
rs1884712	0.244	20	37347940	-0.272	6.32E-04	1.000	*DHX35*	flanking_3UTR
rs9846155	0.456	3	44257844	-0.272	6.33E-04	1.000	*C3orf23*	flanking_5UTR
rs9910052	0.331	17	72180396	0.272	6.34E-04	1.000	*MXRA7*	flanking_3UTR
rs4367521	0.297	8	40097317	0.274	6.34E-04	1.000	*C8orf4*	flanking_5UTR
rs13139732	0.302	4	122404117	-0.272	6.35E-04	1.000	---	downstream
rs5936753	0.177	23	68937321	0.277	6.36E-04	1.000	*EDA*	intron
rs982678	0.378	3	63063355	-0.272	6.36E-04	1.000	*CADPS*	flanking_5UTR
rs1836458	0.189	9	109938708	-0.272	6.36E-04	1.000	*KLF4*	flanking_5UTR
rs171633	0.358	5	16055472	-0.272	6.37E-04	1.000	*FBXL7*	downstream
rs10130237	0.307	14	28076692	-0.273	6.38E-04	1.000	*FOXG1*	upstream
rs12147817	0.287	14	48465623	0.273	6.38E-04	1.000	*RPS29*	flanking_3UTR
rs8047061	0.227	16	5503622	0.272	6.40E-04	1.000	*FAM86A*	upstream
rs342580	0.221	13	59194389	-0.272	6.40E-04	1.000	*DIAPH3*	intron
rs17092866	0.347	14	56654946	-0.277	6.40E-04	1.000	*SEC10L1*	flanking_3UTR
rs1556099	0.34	9	7154502	0.272	6.41E-04	1.000	*JMJD2C*	intron
rs2919999	0.337	18	27038340	0.272	6.41E-04	1.000	*DSC1*	flanking_5UTR
rs17648900	0.201	2	78988857	-0.272	6.42E-04	1.000	*REG3G*	flanking_5UTR
rs12878560	0.325	14	42154236	-0.280	6.43E-04	1.000	*LRFN5*	flanking_3UTR
rs585618	0.526	12	128711360	-0.274	6.44E-04	1.000	*TMEM132D*	intron

rs16897464	0.273	5	67530713	-0.272	6.45E-04	1.000	---	upstream
rs2618321	0.294	3	107181803	-0.272	6.45E-04	1.000	*CBLB*	upstream
rs1051009	0.328	17	4584635	-0.272	6.47E-04	1.000	*CXCL16*	UTR
rs11796706	0.093	23	87850347	-0.272	6.48E-04	1.000	*CPXCR1*	upstream
rs7057626	0.093	23	87856147	-0.272	6.48E-04	1.000	*CPXCR1*	upstream
rs7058384	0.093	23	87856687	-0.272	6.48E-04	1.000	*CPXCR1*	upstream
rs1326816	0.144	9	118658005	0.276	6.49E-04	1.000	*ASTN2*	intron
rs10747922	0.279	12	59825395	0.272	6.49E-04	1.000	*FAM19A2*	flanking_3UTR
rs9936351	0.23	16	6081380	-0.272	6.50E-04	1.000	*FAM86A*	upstream
rs10506882	0.226	12	81729283	0.277	6.51E-04	1.000	*TMTC2*	intron
rs2632115	0.384	5	113461692	-0.272	6.51E-04	1.000	---	downstream
rs2641688	0.17	16	83359252	-0.273	6.53E-04	1.000	*USP10*	intron
rs900334	0.201	1	186779065	0.274	6.55E-04	1.000	*FAM5C*	downstream
rs10809130	0.081	9	10588429	0.272	6.56E-04	1.000	*PTPRD*	flanking_5UTR
rs4947425	0.291	7	53289177	-0.272	6.57E-04	1.000	---	downstream
rs4264615	0.503	20	37648264	0.272	6.57E-04	1.000	*DHX35*	flanking_3UTR
rs210564	0.201	23	151030164	0.272	6.58E-04	1.000	*MAGEA5*	downstream
rs1428608	0.435	5	66727370	-0.274	6.58E-04	1.000	*PIK3R1*	upstream
rs2917538	0.369	11	88921422	0.272	6.59E-04	1.000	*NOX4*	flanking_5UTR
rs9268911	0.306	6	32540384	0.273	6.59E-04	1.000	*HLA-DRA*	flanking_3UTR
rs2354951	0.406	7	11535194	-0.272	6.59E-04	1.000	---	intron
rs1423300	0.474	5	33868190	0.271	6.60E-04	1.000	*ADAMTS12*	intron
rs4811754	0.238	20	54825581	0.271	6.61E-04	1.000	*TFAP2C*	flanking_3UTR
rs6552549	0.269	4	183466215	-0.272	6.62E-04	1.000	*MGC45800*	flanking_5UTR
rs11124194	0.36	2	239955431	0.271	6.62E-04	1.000	*HDAC4*	intron
rs16958237	0.199	15	71862661	0.275	6.63E-04	1.000	*CD276*	flanking_3UTR
rs11687473	0.122	2	2144526	0.271	6.63E-04	1.000	*MYT1L*	intron
rs359295	0.294	10	17373520	0.271	6.64E-04	1.000	*ST8SIA6*	downstream
rs2974486	0.462	5	113464788	-0.272	6.64E-04	1.000	*YTHDC2*	downstream
rs2958693	0.313	8	134204277	-0.272	6.64E-04	1.000	*TG*	intron
rs7945226	0.48	11	40536081	0.272	6.65E-04	1.000	*LRRC4C*	flanking_5UTR
rs7994174	0.122	13	35573018	-0.271	6.66E-04	1.000	*DCAMKL1*	intron
rs650535	0.234	11	116919595	-0.272	6.67E-04	1.000	*DSCAML1*	intron
rs1396980	0.195	8	131911818	-0.271	6.68E-04	1.000	*ADCY8*	intron

rs7185014	0.18	16	77427473	0.271	6.68E-04	1.000	*WWOX*	intron
rs12313805	0.067	12	16415189	0.271	6.68E-04	1.000	*MGST1*	flanking_3UTR
rs7654751	0.349	4	101899556	-0.271	6.69E-04	1.000	*EMCN*	upstream
rs7971987	0.334	12	59816779	0.271	6.71E-04	1.000	*FAM19A2*	flanking_3UTR
rs31042	0.477	16	53887102	-0.271	6.71E-04	1.000	*IRX6*	flanking_5UTR
rs763415	0.102	6	107745262	-0.271	6.71E-04	1.000	*PDSS2*	intron
rs341522	0.215	13	59293903	-0.271	6.73E-04	1.000	*DIAPH3*	intron
rs11215406	0.14	11	114570292	-0.271	6.73E-04	1.000	*IGSF4*	intron
rs9362531	0.337	6	64947857	0.271	6.75E-04	1.000	---	upstream
rs7064269	0.183	23	17096581	0.271	6.75E-04	1.000	*REPS2*	downstream
rs17011297	0.117	4	87152084	0.272	6.78E-04	1.000	*MAPK10*	flanking_3UTR
rs1909675	0.352	10	2980680	-0.271	6.78E-04	1.000	*PFKP*	flanking_5UTR
rs7747521	0.289	6	32539083	0.274	6.78E-04	1.000	*HLA-DRB5*	downstream
rs10810324	0.134	9	1500848	-0.271	6.80E-04	1.000	---	upstream
rs1585047	0.407	5	109438857	0.271	6.81E-04	1.000	---	downstream
rs2911293	0.477	16	61143163	0.271	6.82E-04	1.000	*CDH8*	flanking_5UTR
rs9830720	0.343	3	70625786	-0.271	6.82E-04	1.000	*LOC401072*	flanking_3UTR
rs2247142	0.442	8	14704946	-0.271	6.83E-04	1.000	*SGCZ*	flanking_5UTR
rs2931327	0.109	8	62277765	-0.273	6.85E-04	1.000	*MGC34646*	flanking_5UTR
rs7943518	0.131	11	96502522	0.271	6.86E-04	1.000	*JRKL*	flanking_3UTR
rs3795980	0.07	2	38204318	0.271	6.86E-04	1.000	*CYP1B1*	upstream
rs4755553	0.477	11	40535367	0.271	6.86E-04	1.000	---	downstream
rs17443522	0.138	16	64279119	-0.272	6.89E-04	1.000	*CDH11*	upstream
rs4957850	0.412	5	109397059	0.272	6.89E-04	1.000	---	upstream
rs16844383	0.222	4	3417041	-0.271	6.90E-04	1.000	*HGFAC*	intron
rs6637227	0.172	23	143302908	-0.270	6.92E-04	1.000	*UBE2NL*	flanking_3UTR
rs10797464	0.358	1	231700420	-0.270	6.93E-04	1.000	---	intron
rs7596295	0.512	2	112117068	-0.270	6.94E-04	1.000	*ANAPC1*	flanking_3UTR
rs16981331	0.096	23	87813946	-0.270	6.94E-04	1.000	*CPXCR1*	upstream
rs6102127	0.166	20	38809176	0.273	6.95E-04	1.000	*MAFB*	upstream
rs16972342	0.151	15	78862853	-0.270	6.96E-04	1.000	*KIAA1199*	intron
rs16897466	0.276	5	67530747	-0.270	6.99E-04	1.000	*PIK3R1*	flanking_5UTR
rs309134	0.436	2	136472154	0.270	6.99E-04	1.000	*DARS*	upstream
rs2089540	0.347	4	101918405	-0.275	7.00E-04	1.000	*PPP3CA*	flanking_3UTR

rs9853790	0.483	3	107847241	0.270	7.02E-04	1.000	*CCDC54*	flanking_5UTR
rs7731830	0.201	5	24191293	0.270	7.04E-04	1.000	*CDH10*	downstream
rs4950076	0.474	1	95349885	-0.270	7.04E-04	1.000	*ALG14*	upstream
rs7650967	0.36	3	140061705	0.270	7.05E-04	1.000	*FOXL2*	downstream
rs160974	0.388	5	146149704	0.272	7.06E-04	1.000	*PPP2R2B*	intron
rs1945607	0.471	11	15235997	0.270	7.06E-04	1.000	*SOX6*	downstream
rs2205353	0.488	3	60782868	-0.270	7.06E-04	1.000	---	upstream
rs9651539	0.308	10	13519502	-0.270	7.06E-04	1.000	*C10orf30*	flanking_3UTR
rs17056837	0.224	5	158973112	0.270	7.06E-04	1.000	---	upstream
rs10955775	0.122	8	117488126	-0.270	7.07E-04	1.000	*EIF3S3*	flanking_3UTR
rs943043	0.331	10	25434343	-0.270	7.08E-04	1.000	*GPR158*	flanking_5UTR
rs16894846	0.11	6	34176901	0.270	7.09E-04	1.000	*GRM4*	intron
rs174963	0.18	7	29888844	0.270	7.10E-04	1.000	---	intron
rs2332197	0.375	18	56868322	0.270	7.10E-04	1.000	*CDH20*	flanking_5UTR
rs11975851	0.052	7	20141033	-0.270	7.10E-04	1.000	*7A5*	flanking_3UTR
rs2753592	0.326	14	25532582	0.270	7.11E-04	1.000	---	upstream
rs2753593	0.326	14	25535330	0.270	7.11E-04	1.000	*NOVA1*	flanking_3UTR
rs1917611	0.273	7	46739580	-0.270	7.11E-04	1.000	*TNS3*	flanking_3UTR
rs7607897	0.512	2	165865187	0.270	7.11E-04	1.000	*SCN2A2*	intron
rs12649267	0.468	4	137475703	0.270	7.11E-04	1.000	---	upstream
rs2336820	0.243	3	132489348	-0.271	7.12E-04	1.000	*NEK11*	intron
rs16836269	0.241	3	132487760	-0.270	7.13E-04	1.000	*NEK11*	intron
rs10867192	0.11	9	80203646	0.270	7.14E-04	1.000	*PSAT1*	flanking_3UTR
rs267988	0.342	5	10763716	0.271	7.15E-04	1.000	*DAP*	intron
rs651855	0.442	11	63627386	-0.270	7.15E-04	1.000	*FLRT1*	flanking_5UTR
rs12609319	0.302	19	35756858	-0.270	7.16E-04	1.000	*ZNF536*	flanking_3UTR
rs17039313	0.087	2	2063971	0.270	7.16E-04	1.000	*MYT1L*	intron
rs7643412	0.094	3	104910640	0.271	7.16E-04	1.000	*ZPLD1*	flanking_3UTR
rs573698	0.48	18	22442202	-0.271	7.19E-04	1.000	*KCTD1*	intron
rs13265751	0.244	8	19384299	0.270	7.19E-04	1.000	*ChGn*	intron
rs6520623	0.102	23	39927477	-0.271	7.19E-04	1.000	*BCOR*	flanking_5UTR
rs2725201	0.398	4	89218330	0.270	7.19E-04	1.000	*PKD2*	flanking_3UTR
rs16984422	0.129	20	58882859	-0.271	7.20E-04	1.000	*CDH4*	flanking_5UTR
rs2076393	0.459	20	9407234	0.270	7.20E-04	1.000	*PLCB4*	intron

rs83	0.423	7	11571540	-0.273	7.20E-04	1.000	*THSD7A*	intron
rs7681177	0.439	4	189890618	0.270	7.22E-04	1.000	---	upstream
rs26086	0.087	5	14560905	0.270	7.22E-04	1.000	*TRIO*	intron
rs17131491	0.23	1	91790237	0.270	7.24E-04	1.000	---	upstream
rs1942478	0.159	11	116156673	0.271	7.24E-04	1.000	*ZNF259*	intron
rs3772324	0.387	3	1288477	0.273	7.26E-04	1.000	*CNTN6*	intron
rs5980666	0.181	23	69013405	0.270	7.31E-04	1.000	*EDA*	intron
rs2294972	0.387	22	36339981	-0.269	7.33E-04	1.000	*GGA1*	intron
rs7512040	0.23	1	97554228	0.269	7.35E-04	1.000	*DPYD*	intron
rs12665694	0.073	6	149714016	-0.269	7.39E-04	1.000	*MAP3K7IP2*	intron
rs10494535	0.2	1	179125082	-0.271	7.40E-04	1.000	*XPR1*	flanking_3UTR
rs2438416	0.52	18	22448774	-0.269	7.40E-04	1.000	*KCTD1*	intron
rs7651154	0.477	3	107831132	0.269	7.41E-04	1.000	---	downstream
rs2399116	0.477	3	107836693	0.269	7.41E-04	1.000	*CCDC54*	flanking_5UTR
rs4567954	0.209	2	105097418	-0.269	7.43E-04	1.000	*MRPS9*	downstream
rs2857901	0.218	11	6937087	-0.269	7.43E-04	1.000	*ZNF215*	flanking_3UTR
rs573845	0.123	18	22450928	-0.270	7.44E-04	1.000	*KCTD1*	intron
rs10242019	0.395	7	101314547	-0.269	7.44E-04	1.000	*CUTL1*	intron
rs10912417	0.445	1	186724101	0.269	7.45E-04	1.000	*C1orf99*	flanking_3UTR
rs2843163	0.32	1	2217282	0.269	7.45E-04	1.000	*SKI*	intron
rs6574101	0.401	14	72398589	-0.270	7.46E-04	1.000	*DPF3*	intron
rs7774545	0.305	6	65092143	0.269	7.46E-04	1.000	---	downstream
rs11641647	0.445	16	7134841	0.269	7.47E-04	1.000	*A2BP1*	intron
rs3094549	0.415	6	29463127	0.270	7.49E-04	1.000	*OR12D2*	flanking_5UTR
rs420458	0.366	19	5860780	-0.269	7.51E-04	1.000	*VMAC*	flanking_3UTR
rs10187137	0.18	2	103040866	0.269	7.52E-04	1.000	*TMEM182*	downstream
rs9450901	0.116	6	88936839	0.269	7.52E-04	1.000	*CNR1*	upstream
rs10485170	0.116	6	88939371	0.269	7.52E-04	1.000	*CNR1*	flanking_5UTR
rs4731992	0.131	7	133352637	0.269	7.53E-04	1.000	*EXOC4*	intron
rs12431547	0.186	14	100981155	-0.269	7.59E-04	1.000	*DIO3*	flanking_5UTR
rs4794623	0.235	17	51519463	-0.269	7.59E-04	1.000	*ANKFN1*	flanking_5UTR
rs11094136	0.227	23	68975766	0.269	7.59E-04	1.000	*EDA*	intron
rs6758822	0.404	2	67810327	-0.269	7.59E-04	1.000	*C1D*	flanking_3UTR
rs5924051	0.081	23	86699918	-0.269	7.60E-04	1.000	*KLHL4*	intron

rs6511901	0.422	19	13888652	0.268	7.61E-04	1.000	*CC2D1A*	intron
rs1239925	0.494	12	68689573	-0.271	7.61E-04	1.000	*C12orf28*	flanking_3UTR
rs1950214	0.294	14	32353508	-0.268	7.61E-04	1.000	*AKAP6*	intron
rs856548	0.299	7	46730993	-0.268	7.62E-04	1.000	*TNS3*	flanking_3UTR
rs2250952	0.379	3	67710973	0.270	7.62E-04	1.000	*SUCLG2*	intron
rs1883960	0.319	11	34171077	-0.269	7.63E-04	1.000	*ABTB2*	intron
rs9594542	0.108	13	40891091	0.269	7.64E-04	1.000	*RGC32*	flanking_5UTR
rs11001722	0.244	10	77727752	0.268	7.64E-04	1.000	*C10orf11*	intron
rs28468612	0.096	12	120497990	-0.269	7.65E-04	1.000	*FBXL10*	intron
rs2923126	0.09	11	10299616	-0.268	7.66E-04	1.000	*ADM*	flanking_3UTR
rs3101225	0.105	1	5056105	-0.268	7.66E-04	1.000	*AJAP1*	flanking_3UTR
rs1446975	0.422	1	157895966	0.268	7.67E-04	1.000	*CRP*	flanking_3UTR
rs2794500	0.422	1	157901645	0.268	7.67E-04	1.000	*CRP*	flanking_3UTR
rs731420	0.105	19	48749812	-0.269	7.70E-04	1.000	*XRCC1*	intron
rs7579031	0.459	2	215552199	0.268	7.70E-04	1.000	*ABCA12*	intron
rs2847197	0.216	18	6909628	-0.269	7.71E-04	1.000	*ARHGAP28*	flanking_3UTR
rs7740529	0.316	6	6555608	0.269	7.71E-04	1.000	*LY86*	intron
rs12379128	0.155	9	118340388	-0.272	7.75E-04	1.000	*ASTN2*	intron
rs2590556	0.421	13	92908199	-0.275	7.75E-04	1.000	*GPC6*	intron
rs5981225	0.453	23	68565483	0.268	7.76E-04	1.000	*TMEM28*	upstream
rs10747911	0.353	12	59802379	0.270	7.76E-04	1.000	*FAM19A2*	downstream
rs7656744	0.366	4	104921267	-0.268	7.76E-04	1.000	*TACR3*	flanking_5UTR
rs13168506	0.404	5	135424351	-0.269	7.77E-04	1.000	*TGFBI*	intron
rs1364095	0.447	16	78123651	-0.270	7.78E-04	1.000	*MAF*	downstream
rs1367731	0.129	6	33093177	0.269	7.78E-04	1.000	*HLA-DOA*	flanking_5UTR
rs10916782	0.122	1	20599143	-0.268	7.79E-04	1.000	*FLJ32784*	flanking_3UTR
rs7721079	0.381	5	5367703	-0.268	7.79E-04	1.000	*ADAMTS16*	flanking_3UTR
rs10484178	0.055	14	43966379	-0.268	7.80E-04	1.000	*C14orf155*	flanking_3UTR
rs2938330	0.235	1	114356641	0.268	7.81E-04	1.000	*OLFML3*	flanking_3UTR
rs1158117	0.436	5	113457883	-0.268	7.81E-04	1.000	*KCNN2*	flanking_5UTR
rs2837757	0.084	21	40923069	0.272	7.83E-04	1.000	*DSCAM*	intron
rs1689021	0.238	4	181293620	-0.270	7.84E-04	1.000	---	downstream
rs923381	0.381	3	8289924	0.268	7.85E-04	1.000	*LMCD1*	flanking_5UTR
rs10499807	0.183	7	68097670	0.268	7.86E-04	1.000	---	upstream

rs1537137	0.285	23	68443591	0.268	7.87E-04	1.000	*PJA1*	flanking_5UTR
rs7702107	0.494	5	113448986	-0.268	7.89E-04	1.000	*YTHDC2*	downstream
rs208785	0.131	2	230270700	-0.268	7.89E-04	1.000	*DNER*	intron
rs4266037	0.218	2	105097181	-0.268	7.92E-04	1.000	*MRPS9*	downstream
rs12364011	0.113	11	5359609	-0.268	7.92E-04	1.000	*HBE1*	intron
rs10837965	0.113	11	5359910	-0.268	7.92E-04	1.000	*HBE1*	intron
rs4515584	0.157	8	117499026	-0.268	7.92E-04	1.000	*EIF3S3*	flanking_3UTR
rs6989815	0.157	8	117499872	-0.268	7.92E-04	1.000	*EIF3H*	downstream
rs10429325	0.157	8	117509360	-0.268	7.92E-04	1.000	*EIF3S3*	flanking_3UTR
rs7007515	0.157	8	117513375	-0.268	7.92E-04	1.000	*EIF3H*	downstream
rs10474415	0.267	5	73730174	-0.268	7.93E-04	1.000	*ENC1*	flanking_3UTR
rs7859562	0.137	9	1500590	-0.268	7.94E-04	1.000	---	upstream
rs7019932	0.137	9	1501184	-0.268	7.94E-04	1.000	*DMRT2*	flanking_3UTR
rs12175881	0.34	6	64974739	0.268	7.96E-04	1.000	---	upstream
rs1992876	0.195	4	88092188	0.268	7.96E-04	1.000	*AFF1*	flanking_5UTR
rs6542705	0.189	2	4380057	-0.267	7.98E-04	1.000	*ALLC*	flanking_3UTR
rs9572975	0.143	13	71893968	-0.268	7.98E-04	1.000	---	upstream
rs1887392	0.116	9	19900154	0.267	7.98E-04	1.000	*MLLT3*	downstream
rs3765300	0.151	22	27958806	0.270	7.98E-04	1.000	*EMID1*	intron
rs11027263	0.105	11	23473763	-0.267	8.00E-04	1.000	*GAS2*	flanking_3UTR
rs6641574	0.314	23	3455456	0.267	8.01E-04	1.000	*PRKX*	flanking_3UTR
rs6641574	0.314	23	3456714	0.267	8.01E-04	1.000	*PRKX*	flanking_3UTR
rs6641786	0.314	23	3457762	0.267	8.01E-04	1.000	*PRKX*	flanking_3UTR
rs12463085	0.404	19	51161251	-0.268	8.01E-04	1.000	*NOVA2*	intron
rs11853643	0.134	15	60961100	0.267	8.01E-04	1.000	*TLN2*	flanking_3UTR
rs2630801	0.157	3	21862268	-0.267	8.05E-04	1.000	*ZNF659*	upstream
rs7830715	0.503	8	63920624	0.267	8.06E-04	1.000	*FAM77D*	intron
rs899680	0.503	8	63931285	0.267	8.06E-04	1.000	*FAM77D*	intron
rs4302833	0.503	8	63938162	0.267	8.06E-04	1.000	*FAM77D*	intron
rs6728854	0.148	2	34126640	0.267	8.07E-04	1.000	---	upstream
rs6543803	0.148	2	34126797	0.267	8.07E-04	1.000	*MYADML*	flanking_5UTR
rs632632	0.379	2	136354686	0.269	8.07E-04	1.000	*MCM6*	upstream
rs2938719	0.371	16	61131967	-0.268	8.10E-04	1.000	*CDH11*	downstream
rs7786462	0.123	7	89820495	0.268	8.10E-04	1.000	*GTPBP10*	intron

rs10427663	0.206	22	38447343	-0.267	8.12E-04	1.000	*FLJ25421*	flanking_3UTR
rs7002365	0.193	8	54061570	0.272	8.12E-04	1.000	*NPBWR1*	flanking_3UTR
rs17174150	0.161	23	70797363	-0.268	8.12E-04	1.000	*CXCR3*	flanking_5UTR
rs12149660	0.102	16	68866738	0.268	8.12E-04	1.000	*AARS*	intron
rs2049019	0.462	2	66525362	0.269	8.13E-04	1.000	*MEIS1*	intron
rs1179376	0.387	3	55718098	-0.267	8.14E-04	1.000	*ERC2*	intron
rs10818386	0.14	9	121603950	-0.268	8.14E-04	1.000	*DBC1*	flanking_5UTR
rs27479	0.105	5	14552395	0.267	8.17E-04	1.000	*TRIO*	intron
rs4785524	0.314	16	47177442	-0.267	8.19E-04	1.000	*N4BP1*	intron
rs340268	0.285	3	160077350	-0.267	8.20E-04	1.000	*MFSD1*	flanking_3UTR
rs4149227	0.052	1	224099153	-0.267	8.21E-04	1.000	*EPHX1*	intron
rs4149230	0.052	1	224099653	-0.267	8.21E-04	1.000	*EPHX1*	coding
rs5014833	0.222	5	158974923	0.268	8.22E-04	1.000	*IL12B*	flanking_5UTR
rs13098512	0.244	3	21846756	0.267	8.24E-04	1.000	*ZNF659*	upstream
rs10982177	0.225	9	116153989	0.268	8.24E-04	1.000	*AKNA*	intron
rs13387970	0.5	2	165902211	0.267	8.26E-04	1.000	*SCN2A2*	intron
rs6971210	0.172	7	109484757	0.267	8.27E-04	1.000	*LOC154907*	upstream
rs10482044	0.485	23	87556760	0.268	8.27E-04	1.000	*CPXCR1*	flanking_5UTR
rs320947	0.326	9	28859800	0.267	8.28E-04	1.000	*LRRN6C*	flanking_5UTR
rs10915971	0.338	1	224575268	0.268	8.28E-04	1.000	*LIN9*	flanking_5UTR
rs1874302	0.111	12	79745827	-0.268	8.29E-04	1.000	*LIN7A*	intron
rs12220191	0.34	10	2979572	-0.267	8.30E-04	1.000	*PFKP*	flanking_5UTR
rs12712962	0.515	2	46148315	-0.267	8.32E-04	1.000	*PRKCE*	intron
rs6866525	0.235	5	16087232	-0.267	8.33E-04	1.000	*FBXL7*	flanking_3UTR
rs9535611	0.064	13	50653320	0.266	8.36E-04	1.000	*FAM124A*	upstream
rs341557	0.235	13	59265015	-0.266	8.38E-04	1.000	*DIAPH3*	intron
rs2160891	0.241	12	67163504	0.266	8.38E-04	1.000	*MDM1*	upstream
rs4898763	0.31	14	37358763	-0.267	8.39E-04	1.000	*TTC6*	intron
rs10861740	0.073	12	106371718	0.266	8.40E-04	1.000	*BTBD11*	intron
rs12988076	0.371	2	136286318	0.267	8.41E-04	1.000	*LCT*	intron
rs2216963	0.15	2	34113240	0.268	8.42E-04	1.000	*MYADML*	flanking_5UTR
rs17089784	0.105	4	60298181	-0.266	8.43E-04	1.000	---	upstream
rs17089786	0.105	4	60299217	-0.266	8.43E-04	1.000	*LPHN3*	flanking_5UTR
rs17089787	0.105	4	60300136	-0.266	8.43E-04	1.000	---	downstream

rs1339377	0.419	6	81504086	0.266	8.43E-04	1.000	*BCKDHB*	flanking_3UTR
rs6859383	0.354	5	16009987	-0.267	8.44E-04	1.000	*FBXL7*	flanking_3UTR
rs4237482	0.058	10	129210364	-0.266	8.44E-04	1.000	*DOCK1*	downstream
rs9676752	0.288	19	35750907	-0.266	8.44E-04	1.000	*ZNF536*	flanking_3UTR
rs10244189	0.094	7	11887927	0.267	8.46E-04	1.000	*TMEM106B*	flanking_5UTR
rs2146866	0.355	1	19277824	0.266	8.46E-04	1.000	*UBR4*	intron
rs7816579	0.435	8	27861734	-0.268	8.47E-04	1.000	*SCARA5*	intron
rs1519102	0.43	2	66531320	0.267	8.47E-04	1.000	*MEIS1*	intron
rs16836493	0.288	3	132564794	-0.266	8.48E-04	1.000	*LOC152195*	intron
rs16888206	0.119	5	57810295	0.266	8.48E-04	1.000	*FLJ33641*	flanking_5UTR
rs10835119	0.234	11	27119326	-0.269	8.48E-04	1.000	---	upstream
rs27480	0.116	5	14548485	0.266	8.49E-04	1.000	*TRIO*	intron
rs4486988	0.212	18	6903420	-0.266	8.50E-04	1.000	*ARHGAP28*	3UTR
rs9622658	0.406	22	36333570	-0.267	8.51E-04	1.000	*GGA1*	flanking_5UTR
rs719205	0.254	3	191399629	0.267	8.52E-04	1.000	*LEPREL1*	flanking_5UTR
rs11037178	0.112	11	5366326	-0.268	8.52E-04	1.000	*OR51M1*	flanking_5UTR
rs6131316	0.13	20	12185727	-0.269	8.52E-04	1.000	*BTBD3*	flanking_3UTR
rs7898486	0.352	10	30041439	0.266	8.54E-04	1.000	*SVIL*	intron
rs6434028	0.471	2	152996148	0.266	8.54E-04	1.000	*FMNL2*	intron
rs13178527	0.203	5	76347614	0.266	8.55E-04	1.000	*AGGF1*	flanking_5UTR
rs4029762	0.148	8	41432669	-0.266	8.55E-04	1.000	*GOLGA7*	flanking_5UTR
rs16890592	0.148	8	41434000	-0.266	8.55E-04	1.000	*SFRP1*	upstream
rs17139526	0.067	7	64494392	0.266	8.59E-04	1.000	*ZNF92*	intron
rs272093	0.163	13	90387941	-0.266	8.59E-04	1.000	*GPC5*	flanking_5UTR
rs2128782	0.302	4	127428167	0.266	8.60E-04	1.000	*FAT4*	flanking_3UTR
rs12194244	0.076	6	147260991	0.267	8.61E-04	1.000	---	downstream
rs11639418	0.36	15	53342986	0.266	8.61E-04	1.000	*RAB27A*	intron
rs13077725	0.064	3	30034765	-0.266	8.62E-04	1.000	---	upstream
rs2303518	0.275	15	39897267	-0.267	8.64E-04	1.000	*MAPKBP1*	intron
rs2378357	0.116	3	189226355	0.266	8.66E-04	1.000	*FLJ42393*	flanking_5UTR
rs7699978	0.177	4	87155080	0.266	8.66E-04	1.000	*MAPK10*	flanking_3UTR
rs12950933	0.07	17	30760703	0.266	8.66E-04	1.000	*FLJ10260*	flanking_3UTR
rs10132568	0.299	14	28077458	-0.266	8.67E-04	1.000	*FOXG1B*	flanking_5UTR
rs11173758	0.349	12	59785063	0.266	8.69E-04	1.000	*FAM19A2*	flanking_3UTR

rs1608610	0.349	12	59793888	0.266	8.69E-04	1.000	*FAM19A2*	downstream
rs906263	0.349	12	59808800	0.266	8.69E-04	1.000	*FAM19A2*	flanking_3UTR
rs2555718	0.193	8	126009134	-0.267	8.69E-04	1.000	*ZNF572*	flanking_5UTR
rs6463806	0.113	7	8423878	0.266	8.70E-04	1.000	*NXPH1*	flanking_5UTR
rs10252468	0.113	7	8425293	0.266	8.70E-04	1.000	*NXPH1*	flanking_5UTR
rs10516057	0.221	5	168418548	0.266	8.70E-04	1.000	*SLIT3*	intron
rs5961979	0.221	23	6226939	0.266	8.70E-04	1.000	*NLGN4X*	flanking_5UTR
rs11216129	0.16	11	116125466	0.266	8.71E-04	1.000	*MGC13125*	intron
rs2620868	0.444	12	59657037	0.266	8.72E-04	1.000	*FAM19A2*	flanking_3UTR
rs7132287	0.2	12	81721742	0.272	8.73E-04	1.000	*TMTC2*	intron
rs491576	0.52	6	149664954	-0.265	8.77E-04	1.000	*MAP3K7IP2*	upstream
rs7432940	0.193	3	151463238	0.266	8.80E-04	1.000	*TSC22D2*	flanking_5UTR
rs198493	0.331	20	35703480	0.265	8.81E-04	1.000	*FLJ42133*	flanking_5UTR
rs12425180	0.314	12	83600320	-0.268	8.81E-04	1.000	*SLC6A15*	flanking_3UTR
rs2271668	0.185	1	179115704	-0.267	8.82E-04	1.000	*XPR1*	intron
rs7565158	0.503	2	213302215	-0.269	8.82E-04	1.000	*ERBB4*	flanking_5UTR
rs12436060	0.23	14	32909524	-0.265	8.87E-04	1.000	*NPAS3*	intron
rs230031	0.209	20	49194324	-0.265	8.87E-04	1.000	*KCNG1*	upstream
rs12874831	0.177	13	107685443	0.265	8.88E-04	1.000	*C13orf6*	flanking_3UTR
rs7738894	0.317	6	3825893	-0.265	8.88E-04	1.000	*FAM50B*	downstream
rs233990	0.369	4	104886725	-0.265	8.88E-04	1.000	*TACR3*	flanking_5UTR
rs11873362	0.186	18	18044882	0.265	8.89E-04	1.000	*GATA6*	flanking_3UTR
rs10914427	0.363	1	31755171	0.265	8.91E-04	1.000	*TINAGL1*	upstream
rs17106443	0.099	1	51417290	-0.265	8.91E-04	1.000	*RNF11*	flanking_5UTR
rs10135222	0.418	14	96322054	0.267	8.91E-04	1.000	*VRK1*	upstream
rs2162028	0.363	12	27647755	-0.265	8.91E-04	1.000	*PPFIBP1*	intron
rs9362651	0.419	6	65029395	0.265	8.93E-04	1.000	*PHF3*	flanking_3UTR
rs3013162	0.459	6	64921829	-0.268	8.93E-04	1.000	---	upstream
rs17164526	0.241	5	127274622	-0.265	8.94E-04	1.000	---	upstream
rs7017095	0.27	8	108296237	0.265	8.94E-04	1.000	*ANGPT1*	downstream
rs10488244	0.061	7	13066560	-0.265	8.96E-04	1.000	*ARL4*	flanking_3UTR
rs16875856	0.267	8	108293863	0.265	8.97E-04	1.000	*ANGPT1*	flanking_3UTR
rs7878853	0.099	23	69985573	0.265	8.98E-04	1.000	*TEX11*	intron
rs7974374	0.348	12	59734444	0.266	9.00E-04	1.000	*FAM19A2*	downstream

rs3778799	0.465	7	137035615	0.265	9.03E-04	1.000	*DGKI*	intron
rs9558257	0.48	13	103626677	0.265	9.04E-04	1.000	*SLC10A2*	flanking_5UTR
rs3811575	0.474	2	152991129	0.265	9.05E-04	1.000	*FMNL2*	intron
rs17535696	0.076	2	205507379	-0.265	9.08E-04	1.000	*PARD3B*	intron
rs16835496	0.07	1	33711432	-0.265	9.09E-04	1.000	*ZNF31*	intron
rs842305	0.485	9	7700087	-0.265	9.11E-04	1.000	---	downstream
rs364734	0.122	6	74366122	0.265	9.12E-04	1.000	*SLC17A5*	intron
rs10778912	0.173	12	81725746	0.265	9.12E-04	1.000	*TMTC2*	intron
rs4922030	0.244	8	19367051	0.265	9.14E-04	1.000	*ChGn*	intron
rs10155650	0.456	5	109445307	-0.266	9.16E-04	1.000	*MAN2A1*	flanking_3UTR
rs7187360	0.087	16	5924226	-0.264	9.17E-04	1.000	*FAM86A*	upstream
rs11119499	0.218	1	208677939	0.264	9.17E-04	1.000	*HHAT*	intron
rs12388854	0.192	23	151030110	0.264	9.18E-04	1.000	*MAGEA5*	flanking_3UTR
rs2224806	0.249	9	110296394	-0.265	9.19E-04	1.000	*ACTL7B*	flanking_3UTR
rs12513172	0.113	4	111519959	0.264	9.19E-04	1.000	---	downstream
rs10862511	0.172	12	81725727	0.264	9.19E-04	1.000	*TMTC2*	intron
rs568851	0.078	1	234883747	0.264	9.20E-04	1.000	*LGALS8*	downstream
rs7328247	0.265	13	108825024	-0.264	9.21E-04	1.000	*MYO16*	downstream
rs495385	0.215	6	74409086	0.264	9.21E-04	1.000	*SLC17A5*	intron
rs12437296	0.078	14	60824906	-0.264	9.22E-04	1.000	*TMEM30B*	flanking_5UTR
rs2116209	0.43	12	69235515	0.265	9.24E-04	1.000	*PTPRB*	intron
rs2940918	0.178	5	42506342	-0.267	9.26E-04	1.000	*GHR*	intron
rs2346443	0.209	7	133356923	0.264	9.26E-04	1.000	*EXOC4*	intron
rs1179375	0.453	3	55718238	-0.264	9.27E-04	1.000	*ERC2*	intron
rs6454676	0.125	6	88934174	0.264	9.27E-04	1.000	*CNR1*	flanking_5UTR
rs2514878	0.297	8	108342192	0.264	9.27E-04	1.000	*ANGPT1*	intron
rs4106153	0.254	4	90463499	0.265	9.28E-04	1.000	*LOC285513*	flanking_5UTR
rs4831037	0.474	23	7821358	-0.265	9.28E-04	1.000	*PNPLA4*	flanking_3UTR
rs2362794	0.395	12	59614099	0.265	9.31E-04	1.000	*FAM19A2*	downstream
rs6621108	0.406	23	100707550	0.265	9.31E-04	1.000	*ARMCX1*	flanking_3UTR
rs2046422	0.331	2	151646364	-0.264	9.31E-04	1.000	*FLJ45645*	flanking_3UTR
rs6129355	0.509	20	37579115	-0.264	9.34E-04	1.000	*MAFB*	downstream
rs11668385	0.105	19	48732647	-0.264	9.35E-04	1.000	*ZNF575*	flanking_3UTR
rs6512578	0.474	20	35677224	0.265	9.36E-04	1.000	*BLCAP*	upstream

rs3848991	0.491	8	14383854	0.264	9.38E-04	1.000	*SGCZ*	intron
rs964327	0.131	2	179952722	-0.264	9.38E-04	1.000	*ZNF533*	flanking_3UTR
rs16910529	0.11	10	58765848	-0.264	9.40E-04	1.000	*IPMK*	flanking_3UTR
rs2513724	0.141	11	57600185	-0.266	9.41E-04	1.000	*OR9I1*	flanking_3UTR
rs11014365	0.426	10	25385726	-0.266	9.43E-04	1.000	*THNSL1*	flanking_3UTR
rs9284879	0.433	3	44259588	-0.264	9.43E-04	1.000	*C3orf23*	flanking_5UTR
rs7631790	0.433	3	44274209	-0.264	9.43E-04	1.000	*C3orf23*	flanking_5UTR
rs12631341	0.433	3	44284272	-0.264	9.43E-04	1.000	*C3orf23*	flanking_5UTR
rs12486452	0.433	3	44299569	-0.264	9.43E-04	1.000	*C3orf23*	flanking_5UTR
rs2332064	0.172	4	71364820	-0.264	9.45E-04	1.000	*PROL1*	downstream
rs3135351	0.137	6	32500923	0.265	9.48E-04	1.000	*HLA-DRA*	flanking_5UTR
rs6442316	0.273	3	1294698	0.264	9.49E-04	1.000	*CNTN6*	intron
rs10789636	0.363	11	107076599	0.264	9.49E-04	1.000	---	downstream
rs9418753	0.374	10	128689989	-0.265	9.51E-04	1.000	*DOCK1*	intron
rs6447662	0.202	4	48477404	-0.265	9.51E-04	1.000	*OCIAD1*	flanking_5UTR
rs977780	0.357	21	30247120	-0.265	9.52E-04	1.000	*GRIK1*	flanking_5UTR
rs2832504	0.357	21	30247733	-0.265	9.52E-04	1.000	*GRIK1*	flanking_5UTR
rs8042567	0.297	15	65762653	0.264	9.52E-04	1.000	*MAP2K5*	intron
rs322529	0.211	18	47429438	-0.265	9.53E-04	1.000	*DCC*	upstream
rs2127615	0.145	10	58824063	-0.264	9.55E-04	1.000	*IPMK*	flanking_3UTR
rs17484837	0.172	5	63262207	0.264	9.55E-04	1.000	*HTR1A*	downstream
rs3129891	0.256	6	32523058	0.264	9.58E-04	1.000	*HLA-DRA*	flanking_3UTR
rs17264385	0.084	23	13749112	0.264	9.58E-04	1.000	*GPM6B*	intron
rs11192557	0.14	10	107442673	-0.264	9.59E-04	1.000	*SORCS3*	flanking_3UTR
rs11024591	0.177	11	18241781	0.264	9.59E-04	1.000	---	upstream
rs11107562	0.085	12	76879913	-0.265	9.59E-04	1.000	*NAV3*	intron
rs2615550	0.224	18	47416578	-0.264	9.59E-04	1.000	*RKHD2*	flanking_5UTR
rs4824801	0.447	23	49373678	0.265	9.59E-04	1.000	*PAGE4*	upstream
rs827305	0.055	10	72360367	-0.264	9.60E-04	1.000	*PCBD1*	upstream
rs11806304	0.145	1	184639488	0.266	9.60E-04	1.000	*OCLM*	flanking_3UTR
rs6711114	0.491	2	174186048	0.263	9.62E-04	1.000	---	downstream
rs10784138	0.351	12	59797095	0.264	9.62E-04	1.000	*FAM19A2*	flanking_3UTR
rs2038237	0.131	20	18296063	0.263	9.63E-04	1.000	*MGC44328*	flanking_5UTR
rs3770770	0.241	2	37046370	0.263	9.65E-04	1.000	*STRN*	intron

rs4490946	0.346	9	14625546	0.263	9.65E-04	1.000	*ZDHHC21*	intron
rs683266	0.41	11	107077060	0.263	9.66E-04	1.000	*ELMOD1*	downstream
rs4729759	0.43	7	101323606	-0.263	9.67E-04	1.000	*CUTL1*	intron
rs10264866	0.43	7	101324246	-0.263	9.67E-04	1.000	*CUTL1*	intron
rs962491	0.243	4	156579122	0.264	9.68E-04	1.000	*FLJ21159*	flanking_5UTR
rs9544389	0.163	13	76319207	0.263	9.70E-04	1.000	*KCTD12*	downstream
rs757842	0.253	7	105333747	-0.263	9.71E-04	1.000	---	upstream
rs10780336	0.433	9	71193349	-0.263	9.71E-04	1.000	*C9orf61*	intron
rs8134014	0.138	21	46726633	0.268	9.71E-04	1.000	*DIP2A*	intron
rs5991950	0.41	23	100718247	0.268	9.72E-04	1.000	*ARMCX1*	downstream
rs7118671	0.112	11	6890578	-0.265	9.73E-04	1.000	---	upstream
rs4921651	0.231	8	19383365	0.264	9.75E-04	1.000	*ChGn*	intron
rs7264045	0.436	20	37573514	0.264	9.75E-04	1.000	*DHX35*	flanking_3UTR
rs6721576	0.121	2	88519622	0.266	9.75E-04	1.000	*C2orf51*	upstream
rs10810325	0.135	9	1500881	-0.264	9.76E-04	1.000	*DMRT2*	flanking_3UTR
rs2471366	0.355	23	150995958	0.263	9.76E-04	1.000	---	upstream
rs2471367	0.355	23	150995990	0.263	9.76E-04	1.000	*GABRE*	upstream
rs2832484	0.372	21	30219280	-0.263	9.78E-04	1.000	*GRIK1*	intron
rs3789576	0.101	1	50718114	-0.266	9.78E-04	1.000	*FAF1*	intron
rs10421826	0.453	19	33591022	0.263	9.78E-04	1.000	---	upstream
rs10759180	0.491	9	108202637	-0.263	9.79E-04	1.000	*ZNF462*	flanking_5UTR
rs10947260	0.134	6	32481163	0.263	9.79E-04	1.000	*BTNL2*	intron
rs11894441	0.319	2	3679847	-0.264	9.80E-04	1.000	---	downstream
rs13012163	0.317	2	207669241	-0.263	9.80E-04	1.000	*KLF7*	intron
rs6759901	0.317	2	207670701	-0.263	9.80E-04	1.000	*KLF7*	intron
rs9885986	0.079	7	142434184	0.265	9.80E-04	1.000	*OR9A2*	coding
rs922471	0.433	16	7130898	0.263	9.82E-04	1.000	---	downstream
rs16990824	0.067	22	31329059	-0.264	9.84E-04	1.000	*SYN3*	flanking_5UTR
rs6624385	0.386	23	68300850	-0.264	9.86E-04	1.000	*PJA1*	intron
rs7027671	0.201	9	71139288	0.263	9.87E-04	1.000	*C9orf61*	intron
rs11060480	0.462	12	128692468	0.265	9.87E-04	1.000	*KIAA1944*	intron
rs4759943	0.209	12	128505062	0.263	9.88E-04	1.000	*KIAA1944*	intron
rs3791316	0.377	2	134899768	-0.264	9.88E-04	1.000	*MGAT5*	intron
rs3923277	0.23	2	105103586	-0.263	9.90E-04	1.000	*MRPS9*	flanking_3UTR

rs842295	0.436	9	7710367	0.263	9.90E-04	1.000	*C9orf123*	downstream
rs9567458	0.27	13	44141639	0.263	9.90E-04	1.000	*TSC22D1*	flanking_5UTR
rs2914746	0.297	5	6349991	0.263	9.91E-04	1.000	*FLJ33360*	flanking_3UTR
rs11099613	0.227	4	84808928	-0.263	9.91E-04	1.000	---	upstream
rs17356080	0.227	4	84810065	-0.263	9.91E-04	1.000	---	upstream
rs12643503	0.227	4	84812865	-0.263	9.91E-04	1.000	*HMFN0839*	flanking_3UTR
rs7049211	0.392	9	94623339	-0.272	9.91E-04	1.000	*ANKRD19*	intron
rs7305888	0.263	12	82617059	0.264	9.92E-04	1.000	*SLC6A15*	downstream
rs9313443	0.298	5	168421776	0.264	9.93E-04	1.000	*SLIT3*	intron
rs554427	0.294	6	163469861	0.263	9.93E-04	1.000	*PACRG*	intron
rs180396	0.212	13	59335498	-0.263	9.94E-04	1.000	*DIAPH3*	intron
rs6871305	0.219	5	84101130	0.264	9.95E-04	1.000	*EDIL3*	upstream
rs6690942	0.154	1	149896582	0.263	9.95E-04	1.000	*SNX27*	intron
rs10158468	0.246	1	19274977	0.264	9.95E-04	1.000	*RBAF600*	intron
rs12472079	0.453	2	132014938	-0.264	9.95E-04	1.000	*CCDC74A*	flanking_3UTR
rs9329384	0.163	23	115827823	-0.265	9.96E-04	1.000	*LOC203413*	flanking_5UTR
rs803454	0.161	6	151289387	0.264	9.97E-04	1.000	*MTHFD1L*	intron
rs9284977	0.355	5	168421536	0.263	9.97E-04	1.000	*SLIT3*	intron
---	0.067	1	219536688	0.264	9.97E-04	1.000	*HLX*	downstream
rs939046	0.404	11	15231818	0.263	9.98E-04	1.000	*LOC387755*	flanking_3UTR
rs10961628	0.343	9	14623286	0.263	9.98E-04	1.000	*ZDHHC21*	intron
rs6899693	0.301	6	93575648	-0.264	9.98E-04	1.000	*EPHA7*	downstream
rs6460851	0.275	7	11868337	0.263	9.99E-04	1.000	*TMEM106B*	flanking_5UTR
rs13200744	0.131	6	161369566	-0.263	9.99E-04	1.000	*MAP3K4*	intron

Table S1B AraC

rsID	MAF	Chr	Position	R value	P value	Q value	Gene Symbol	Location
rs4078252	0.198	5	112483366	0.405	1.54E-07	0.206	*ıcer protein (Protein I*	intron
rs10495285	0.171	1	227871618	0.393	4.13E-07	0.231	*KIAA0133 (KIAA0133,*	downstream
rs1450679	0.098	9	106066633	0.389	5.20E-07	0.231	*SMC2L1*	flanking_3UTR
rs6128386	0.466	20	56626955	0.375	1.38E-06	0.285	*'94 protein. [Source:U*	upstream
rs6011674	0.129	20	61324897	0.373	1.62E-06	0.285	*YTHDF1*	flanking_5UTR
rs2172820	0.256	8	15370039	-0.373	1.63E-06	0.285	*TUSC3*	flanking_5UTR
rs2857891	0.101	11	6919533	-0.372	1.75E-06	0.285	*ZNF215*	intron
rs2595500	0.101	11	6919741	-0.372	1.75E-06	0.285	*ZNF215*	intron
rs2604376	0.448	8	15686584	-0.372	1.92E-06	0.285	*TUSC3*	flanking_3UTR
rs12062780	0.173	1	227871496	0.364	3.15E-06	0.387	*ır RNA [Source:RFAM*	downstream
rs9512755	0.055	13	27056229	-0.361	3.67E-06	0.387	*LNX2*	flanking_5UTR
rs17811793	0.06	13	27062859	-0.360	4.02E-06	0.387	*LNX2*	intron
rs9512757	0.055	13	27063233	-0.360	4.29E-06	0.387	*LNX2*	intron
rs4936323	0.135	11	114596329	-0.358	4.48E-06	0.387	*ıunoglobulin superfa*	intron
rs11215427	0.135	11	114598648	-0.358	4.48E-06	0.387	*ıunoglobulin superfa*	intron
rs888468	0.184	12	4403640	-0.356	5.08E-06	0.387	*FGF6*	flanking_3UTR
rs970084	0.08	20	1169171	-0.356	5.26E-06	0.387	*SNPH*	flanking_5UTR
rs7170930	0.058	15	64367907	0.356	5.38E-06	0.387	*MGC4562*	flanking_5UTR
rs11710229	0.064	3	55472993	0.355	5.69E-06	0.387	*rane domains 1 [Sour*	upstream
rs9512745	0.054	13	27019315	-0.359	6.52E-06	0.387	*LNX2*	3UTR
rs10208516	0.052	2	81428269	0.352	6.75E-06	0.387	*CTNNA2*	flanking_3UTR
rs2139424	0.101	11	20640616	0.353	6.76E-06	0.387	---	downstream
rs888465	0.188	12	4403937	-0.353	6.81E-06	0.387	*'Tumor-derived hypo*	upstream
rs2583612	0.144	4	120832166	0.351	6.97E-06	0.387	*cGMP-specific 3'*	upstream
rs9363058	0.193	6	94065256	0.349	7.78E-06	0.388	*EPHA7*	intron
rs11700100	0.156	20	55513455	0.354	7.87E-06	0.388	*CTCFL*	intron
rs7588926	0.078	2	27196398	0.348	8.59E-06	0.388	*CGREF1*	flanking_5UTR
rs4553261	0.161	10	71045795	-0.348	8.68E-06	0.388	---	upstream
rs2653165	0.207	1	240432905	-0.347	9.28E-06	0.388	*PLD5*	intron
rs7827167	0.462	8	15643069	0.347	9.42E-06	0.388	*TUSC3*	intron
rs2885135	0.164	1	13779114	0.346	9.46E-06	0.388	*t domain containing*	upstream
rs11215416	0.147	11	114582537	-0.346	9.57E-06	0.388	*IGSF4*	intron
rs888466	0.182	12	4403866	-0.347	9.86E-06	0.388	*ecursor (FGF-6) (HBG*	downstream
rs17357392	0.132	7	12638058	-0.346	9.89E-06	0.388	*SCIN*	intron
rs8098925	0.479	18	69257838	0.349	1.07E-05	0.392	*pecific transmembra*	upstream
rs6657788	0.063	1	163866428	0.343	1.13E-05	0.392	*MGST3*	flanking_5UTR
rs2026340	0.195	9	88494823	0.343	1.16E-05	0.392	*GAS1*	flanking_3UTR
rs4682718	0.339	3	44201336	0.343	1.16E-05	0.392	*CDNA FLJ36157 fis*	upstream
rs8099090	0.474	18	69257965	0.343	1.18E-05	0.392	*NETO1*	flanking_5UTR
rs11875511	0.474	18	69258464	0.343	1.18E-05	0.392	*pecific transmembra*	upstream
rs11609969	0.06	12	111806963	0.342	1.24E-05	0.404	*RPH3A*	intron
rs7295812	0.207	12	4406357	-0.340	1.41E-05	0.421	*FGF6*	flanking_3UTR
rs10211393	0.247	2	238259527	-0.340	1.44E-05	0.421	*protein Rab-17. [Sou*	upstream
rs856541	0.299	7	46722666	-0.340	1.44E-05	0.421	---	upstream
rs856554	0.299	7	46726654	-0.340	1.44E-05	0.421	*TNS3*	flanking_3UTR
rs2772357	0.155	13	97759317	0.339	1.46E-05	0.421	*FERM*	intron
rs5963842	0.046	23	40499640	0.339	1.48E-05	0.421	*(p) (ZNF127-Xp) (RIN*	upstream
rs17564430	0.155	11	114548784	-0.337	1.74E-05	0.443	*IGSF4*	flanking_3UTR
rs10060641	0.216	5	90249006	0.335	1.90E-05	0.443	*MASS1*	intron
rs11215406	0.138	11	114570292	-0.335	1.92E-05	0.443	*IGSF4*	intron
rs13204330	0.063	6	10702246	0.335	1.94E-05	0.443	*GCNT2*	intron
rs9466910	0.063	6	10707265	0.335	1.94E-05	0.443	*GCNT2*	intron
rs9466912	0.063	6	10707306	0.335	1.94E-05	0.443	*GCNT2*	intron
rs5989586	0.457	23	6757306	0.336	1.94E-05	0.443	*HDHD1A*	flanking_3UTR
rs10134506	0.063	14	23659658	0.335	1.94E-05	0.443	*at protein 23. [Sourc*	intron
rs10858151	0.282	9	137722471	-0.339	1.95E-05	0.443	*ic helix-loop-helix 1 [*	downstream

rs12522395	0.215	5	90246274	0.337	1.97E-05	0.443	*omolog precursor (Ve*	intron
rs10438939	0.069	18	18111077	0.334	1.98E-05	0.443	---	downstream
rs11876487	0.069	18	18121360	0.334	1.98E-05	0.443	*GATA6*	flanking_3UTR
rs7651468	0.069	3	60547113	0.334	1.99E-05	0.443	*FHIT*	intron
rs12054681	0.207	5	90253683	0.334	2.09E-05	0.457	*MASS1*	intron
rs12128558	0.055	1	5492078	0.330	2.61E-05	0.550	*NPHP4*	flanking_3UTR
rs4698388	0.239	4	15092386	-0.330	2.66E-05	0.550	*CDNA FLJ13814 fis*	intron
rs2837757	0.086	21	40923069	0.335	2.69E-05	0.550	---	intron
rs10052015	0.214	5	90244819	0.335	2.73E-05	0.550	*omolog precursor (Ve*	intron
rs2715023	0.176	4	120829316	0.330	2.75E-05	0.550	*PDE5A*	flanking_5UTR
rs216465	0.494	17	25880801	-0.330	2.76E-05	0.550	*GOSR1*	flanking_3UTR
rs4795555	0.489	17	25848621	-0.329	2.82E-05	0.553	*GOSR1*	intron
rs16845089	0.066	2	141459547	0.328	2.88E-05	0.554	*LRP1B*	intron
rs2638094	0.103	11	6938064	-0.328	2.91E-05	0.554	*ZNF215*	flanking_3UTR
rs800379	0.127	4	24480302	0.329	2.99E-05	0.563	*DKFZp761B107*	intron
rs10927903	0.158	1	13785474	0.327	3.11E-05	0.576	*PDPN*	intron
rs12248645	0.092	10	66555921	0.328	3.16E-05	0.578	*ANXA2P3*	flanking_3UTR
rs856548	0.296	7	46730993	-0.326	3.29E-05	0.587	*TNS3*	flanking_3UTR
rs6689258	0.267	1	234362702	0.325	3.40E-05	0.587	*GPR137B*	flanking_5UTR
rs12455924	0.138	18	11054409	-0.325	3.49E-05	0.587	*C18orf58*	flanking_5UTR
rs11136070	0.052	8	29372156	0.324	3.60E-05	0.587	*DUSP4*	flanking_5UTR
rs3794794	0.477	17	25744867	-0.325	3.61E-05	0.587	*CPD*	intron
rs10798854	0.46	1	31706930	-0.325	3.63E-05	0.587	*SERINC2*	flanking_3UTR
rs975336	0.494	21	15373716	0.324	3.74E-05	0.587	*NRIP1*	flanking_5UTR
rs7854627	0.083	9	106040213	0.323	3.83E-05	0.587	*Chromosome- associ*	downstream
rs7337722	0.071	13	27117158	-0.327	3.86E-05	0.587	*NA-directed RNA pol*	intron
rs685272	0.382	11	63993609	0.323	3.90E-05	0.587	*er 11 (Organic anion*	upstream
rs6722230	0.084	2	74342570	-0.325	3.90E-05	0.587	*4 isoform c [Source:R*	intron
rs9924623	0.089	16	53282997	0.323	3.93E-05	0.587	*IRX5*	flanking_5UTR
rs3848370	0.141	16	4300315	0.323	3.99E-05	0.587	*2 [Source:RefSeq_pe*	upstream
rs4795556	0.489	17	25860700	-0.322	4.06E-05	0.587	*GOSR1*	intron
rs7192	0.371	6	32519624	0.322	4.10E-05	0.587	*HLA-DRA*	coding
rs7194	0.371	6	32520458	0.322	4.10E-05	0.587	*HLA-DRA*	3UTR
rs7195	0.371	6	32520517	0.322	4.10E-05	0.587	*HLA-DRA*	3UTR
rs17009792	0.083	2	74342831	-0.322	4.13E-05	0.587	*SLC4A5*	coding
rs655969	0.055	4	130679278	0.323	4.20E-05	0.587	*CDNA FLJ33703 fis*	downstream
rs216463	0.488	17	25877656	-0.322	4.32E-05	0.587	*GOSR1*	3UTR
rs5989585	0.433	23	6757031	0.323	4.33E-05	0.587	*HDHD1A*	flanking_3UTR
rs9511575	0.211	13	24464804	0.322	4.38E-05	0.587	*LOC390387*	intron
rs11061274	0.457	12	130044192	0.321	4.38E-05	0.587	*precursor (G-protein*	intron
rs11920001	0.138	3	4751784	0.321	4.39E-05	0.587	*ITPR1*	intron
rs4572738	0.321	3	55799452	0.322	4.45E-05	0.587	*CAST1*	intron
rs1357643	0.089	2	153512701	-0.321	4.47E-05	0.587	*ARL6IP6*	flanking_3UTR
rs1202292	0.124	6	20041139	-0.321	4.65E-05	0.587	*ID4*	flanking_3UTR
rs216485	0.506	17	25896751	-0.320	4.66E-05	0.587	---	upstream
rs10518756	0.178	15	40310084	-0.320	4.67E-05	0.587	*brane protein 87A (T*	intron
rs800377	0.129	4	24479977	0.320	4.72E-05	0.587	*CDNA FLJ43363 fis*	intron
rs1884190	0.171	6	170438624	-0.324	4.78E-05	0.587	*hila Delta homolog 1,*	intron
rs3806022	0.201	6	70860174	-0.319	5.01E-05	0.587	*COL19A1*	intron
rs1433446	0.109	15	85351874	0.319	5.05E-05	0.587	*TMEM83*	flanking_5UTR
rs4916829	0.192	5	90231203	0.321	5.05E-05	0.587	*MASS1*	intron
rs12263787	0.141	10	121823080	0.318	5.14E-05	0.587	*ting protein (p125). [S*	downstream
rs10788034	0.141	10	121845544	0.318	5.14E-05	0.587	*SEC23IP*	flanking_3UTR
rs1010745	0.474	9	83576532	-0.318	5.19E-05	0.587	*TLE1*	flanking_5UTR
rs540018	0.411	9	137724742	-0.318	5.20E-05	0.587	*C9orf157*	flanking_3UTR
rs2970806	0.305	12	4407919	-0.318	5.20E-05	0.587	*FGF6*	flanking_3UTR
rs11124194	0.359	2	239955431	0.318	5.22E-05	0.587	*HDAC4*	intron
rs1863618	0.276	1	81222832	0.318	5.23E-05	0.587	*LPHN2*	flanking_5UTR

rs4247634	0.325	11	63983064	0.318	5.33E-05	0.587	*LOC439914*	flanking_3UTR
rs2008801	0.126	11	114513385	-0.317	5.42E-05	0.587	*IGSF4*	flanking_3UTR
rs2507905	0.126	11	114513815	-0.317	5.42E-05	0.587	*with sequence simila*	downstream
rs7122402	0.126	11	114521517	-0.317	5.42E-05	0.587	*with sequence simila*	downstream
rs5989582	0.428	23	6756228	0.318	5.43E-05	0.587	*HDHD1A*	flanking_3UTR
rs2272642	0.133	8	23350357	0.318	5.44E-05	0.587	*ENTPD4*	intron
rs2213586	0.373	6	32521072	0.318	5.44E-05	0.587	*HLA-DRA*	flanking_3UTR
rs2213585	0.373	6	32521128	0.318	5.44E-05	0.587	*HLA-DRA*	flanking_3UTR
rs2227139	0.373	6	32521437	0.318	5.44E-05	0.587	*HLA-DRA*	flanking_3UTR
rs300962	0.269	5	119909842	0.320	5.47E-05	0.587	*LOC51334*	intron
rs533486	0.454	7	99278630	-0.317	5.51E-05	0.587	*CYP3A43*	intron
rs6074381	0.117	20	1209152	-0.320	5.57E-05	0.587	*SNPH*	intron
rs4856462	0.055	3	83692031	0.317	5.68E-05	0.587	*GBE1*	flanking_5UTR
rs17764944	0.052	14	69573455	0.316	5.72E-05	0.587	*Sodium*	downstream
rs12952976	0.149	17	2437350	0.316	5.86E-05	0.587	*PAFAH1B1*	flanking_5UTR
rs11627233	0.055	14	69593658	0.316	5.97E-05	0.587	*Sodium*	intron
rs12807015	0.301	11	19916723	0.317	5.98E-05	0.587	*NAV2*	intron
rs6871305	0.217	5	84101130	0.317	5.99E-05	0.587	---	downstream
rs3759443	0.113	13	98708323	-0.317	6.07E-05	0.587	*GPR18*	intron
rs1009523	0.497	4	178388429	0.317	6.08E-05	0.587	*flanking_5UTR*	NM_01824
rs516454	0.287	11	81427638	-0.315	6.08E-05	0.587	*MGC33846*	flanking_3UTR
rs4457417	0.159	9	89271011	-0.316	6.10E-05	0.587	*CDNA FLJ45537 fis*	downstream
rs6441911	0.055	3	45322523	0.315	6.20E-05	0.587	*RIS1*	flanking_5UTR
rs9846284	0.055	3	45331970	0.315	6.20E-05	0.587	*lar RNA [Source:RFAM*	upstream
rs9850725	0.055	3	45332431	0.315	6.20E-05	0.587	*LARS2*	flanking_5UTR
rs12243390	0.101	10	66562233	0.315	6.20E-05	0.587	*ANXA2P3*	flanking_3UTR
rs4963767	0.451	12	24544939	0.315	6.21E-05	0.587	*SOX5*	intron
rs296611	0.072	9	92874015	0.316	6.24E-05	0.587	*CDNA FLJ46870 fis*	intron
rs11873008	0.474	18	69255577	0.315	6.31E-05	0.589	*NETO1*	flanking_5UTR
rs651430	0.457	7	99267779	-0.314	6.47E-05	0.600	*CYP3A43*	intron
rs1159388	0.172	13	35544706	0.314	6.61E-05	0.608	*DCAMKL1*	intron
rs9816196	0.055	3	45321382	0.315	6.70E-05	0.609	*lar RNA [Source:RFAM*	upstream
rs12626815	0.066	21	40916803	0.313	6.81E-05	0.609	*DSCAM*	intron
rs718979	0.394	15	23325370	0.313	6.82E-05	0.609	*UBE3A*	flanking_5UTR
rs1360176	0.139	9	105894537	0.314	6.91E-05	0.609	*Chromosome- associ*	upstream
rs216469	0.497	17	25887105	-0.313	6.94E-05	0.609	*flanking_3UTR*	NM_00100
rs457745	0.414	5	75985561	0.313	6.94E-05	0.609	*ing-like protein IQGA*	intron
rs2165929	0.173	5	112513054	0.314	6.99E-05	0.609	*MCC*	intron
rs1917611	0.27	7	46739580	-0.313	7.02E-05	0.609	*TNS3*	flanking_3UTR
rs1404304	0.185	5	119910612	0.319	7.09E-05	0.609	*n DSC54 [Source:RefS*	intron
rs4963768	0.448	12	24545543	0.313	7.12E-05	0.609	*SOX5*	intron
rs6772740	0.463	3	29034655	0.312	7.24E-05	0.609	*RNA binding motif*	upstream
rs763416	0.109	6	107745473	-0.312	7.25E-05	0.609	*Decaprenyl pyrophos*	intron
rs12900484	0.21	15	91590200	-0.312	7.28E-05	0.609	---	downstream
rs462307	0.41	5	75985949	0.314	7.44E-05	0.609	*IQGAP2*	intron
rs7175451	0.075	15	64336757	0.311	7.56E-05	0.609	*ource:RefSeq_peptid*	upstream
rs6767155	0.066	3	151656124	0.311	7.60E-05	0.609	*TSC22D2*	intron
rs2361030	0.181	1	13779141	0.311	7.72E-05	0.609	*PDPN*	flanking_5UTR
rs9987304	0.161	8	14558151	0.311	7.79E-05	0.609	*SGCZ*	flanking_5UTR
rs10045186	0.055	5	43186257	0.311	7.80E-05	0.609	*FLJ10246*	flanking_3UTR
rs16925920	0.086	9	108713065	0.311	7.86E-05	0.609	*ZNF462*	intron
rs7242238	0.103	18	18110193	0.311	7.86E-05	0.609	*ociated antigen 1 (cT*	downstream
rs7993715	0.126	13	98806314	-0.311	7.86E-05	0.609	*ase like 1 [Source:Ref*	intron
rs7076860	0.135	10	14887710	0.311	7.88E-05	0.609	*GeneID:441549*	flanking_3UTR
rs1465465	0.5	16	17454968	0.311	7.94E-05	0.609	*XYLT1*	intron
rs2144393	0.092	10	83840990	0.311	7.95E-05	0.609	*NRG3*	intron
rs9651385	0.287	10	129158529	-0.310	7.98E-05	0.609	*DOCK1*	flanking_3UTR
rs11638382	0.083	15	27284180	-0.310	8.03E-05	0.609	---	upstream

rs1778214	0.198	1	94833943	0.310	8.04E-05	0.609	*oagulation factor III (*	upstream
rs800672	0.454	7	99274134	-0.310	8.04E-05	0.609	*CYP3A43*	intron
rs5952411	0.135	23	43706891	0.310	8.06E-05	0.609	*Vorrie disease protein*	intron
rs4457416	0.153	9	89270858	-0.311	8.15E-05	0.609	*DAPK1*	flanking_5UTR
rs517218	0.055	11	132592535	0.310	8.20E-05	0.609	*Opioid-binding protein*	intron
rs17294876	0.049	18	68607270	0.311	8.28E-05	0.609	*NETO1*	intron
rs10123121	0.064	9	2838800	0.313	8.28E-05	0.609	---	downstream
rs9878275	0.055	3	45331043	0.311	8.31E-05	0.609	*ble leucyl-tRNA synth*	upstream
rs11728800	0.217	4	15084094	-0.311	8.33E-05	0.609	*C1QTNF7*	flanking_3UTR
rs16873461	0.115	5	75870204	-0.310	8.38E-05	0.609	*ing-like protein IQGA*	intron
rs1218953	0.049	13	27104469	-0.310	8.38E-05	0.609	*POLR1D*	flanking_3UTR
rs12412561	0.069	10	54839547	-0.310	8.39E-05	0.609	*PCDH15*	flanking_3UTR
rs28581693	0.09	8	20641237	-0.310	8.48E-05	0.612	*LZTS1*	flanking_5UTR
rs7726945	0.09	5	128062298	-0.310	8.64E-05	0.618	*FBN2*	flanking_5UTR
rs1955412	0.21	14	85082547	0.309	8.74E-05	0.618	*FLRT2*	intron
rs7906586	0.322	10	85635481	-0.309	8.74E-05	0.618	*al RNA [Source:RFAM*	upstream
rs2395182	0.206	6	32521295	0.310	8.89E-05	0.618	*HLA-DRA*	flanking_3UTR
rs9609948	0.086	22	32836382	0.308	8.93E-05	0.618	*LARGE*	flanking_5UTR
rs141108	0.503	17	25886160	-0.309	8.98E-05	0.618	*flanking_3UTR*	NM_00100
rs11129425	0.293	3	30845260	0.308	9.01E-05	0.618	*GADL1*	intron
rs6688981	0.247	1	234362456	0.308	9.03E-05	0.618	*GPR137B*	flanking_5UTR
rs9861198	0.086	3	45310394	0.308	9.07E-05	0.618	*ble leucyl-tRNA synth*	upstream
rs7842304	0.055	8	29381580	0.308	9.07E-05	0.618	---	downstream
rs11773665	0.089	7	70868875	-0.308	9.09E-05	0.618	---	upstream
rs2585769	0.477	11	19918609	0.308	9.19E-05	0.618	*NAV2*	intron
rs736626	0.109	17	11514308	0.308	9.22E-05	0.618	*DNAH9*	intron
rs2191078	0.109	17	11527648	0.308	9.22E-05	0.618	*onemal beta dynein*	intron
rs17731496	0.103	2	140321504	-0.308	9.30E-05	0.621	*LRP1B*	flanking_3UTR
rs5998364	0.138	22	30994612	-0.307	9.52E-05	0.632	*SLC5A4*	flanking_5UTR
rs10747833	0.109	12	57993525	0.307	9.68E-05	0.639	*SLC16A7*	flanking_5UTR
rs1697648	0.428	8	16651765	-0.307	9.71E-05	0.639	*h factor 20 (FGF-20).*	downstream
rs2026410	0.233	10	56015517	0.307	9.79E-05	0.640	*PCDH15*	intron
rs7479652	0.287	11	5249689	-0.311	1.00E-04	0.651	*oglobin epsilon chain,*	intron
rs7641474	0.204	3	55796151	0.306	1.00E-04	0.651	*CAST1*	intron
rs3115441	0.175	16	20898477	0.306	1.01E-04	0.652	*DNAH3*	intron
rs6964209	0.133	7	95858370	0.307	1.02E-04	0.653	*SLC25A13*	flanking_5UTR
rs897746	0.195	5	95947285	0.306	1.03E-04	0.660	*CAST*	flanking_5UTR
rs1875174	0.26	10	2961488	-0.309	1.04E-04	0.662	---	downstream
rs698084	0.167	3	188480342	0.306	1.05E-04	0.662	*MASP1*	intron
rs10089142	0.221	8	21947885	0.306	1.05E-04	0.662	*NPM2*	intron
rs10499807	0.181	7	68097670	0.305	1.06E-04	0.664	---	upstream
rs10976070	0.445	9	7140975	-0.305	1.08E-04	0.672	*JMJD2C*	intron
rs1897163	0.132	2	153629300	-0.305	1.09E-04	0.672	*cting protein 6 [Sourc*	downstream
rs13436452	0.092	5	123902748	0.306	1.10E-04	0.672	*ZNF608*	flanking_3UTR
rs10892179	0.115	11	117197331	0.305	1.11E-04	0.672	*FXYD2*	intron
rs699513	0.113	7	40043557	-0.314	1.11E-04	0.672	*.22) (CDC2- related p*	intron
rs7735534	0.213	5	84102589	0.305	1.11E-04	0.672	*EDIL3*	flanking_5UTR
rs10474079	0.04	5	82409673	0.304	1.12E-04	0.672	*XRCC4*	intron
rs26442	0.231	5	165305788	0.305	1.12E-04	0.672	---	downstream
rs10928087	0.098	2	141458573	0.304	1.13E-04	0.672	*LRP1B*	intron
rs6994051	0.092	8	83856365	0.304	1.14E-04	0.672	*g nexin-16. [Source:U*	upstream
rs6999352	0.092	8	83857219	0.304	1.14E-04	0.672	*SNX16*	flanking_5UTR
rs10519640	0.187	5	119933582	0.304	1.15E-04	0.672	*n DSC54 [Source:RefS*	intron
rs8106213	0.073	19	20449621	-0.306	1.15E-04	0.672	*ZNF626*	flanking_3UTR
rs7827906	0.431	8	16653243	0.304	1.15E-04	0.672	*FGF20*	flanking_3UTR
rs2230343	0.101	13	98746098	-0.305	1.16E-04	0.672	*EBI2*	coding
rs12683056	0.451	9	7142400	-0.304	1.16E-04	0.672	*3C (EC 1.14.11.-) (Jun*	intron
rs1979113	0.497	3	29032190	0.313	1.16E-04	0.672	*RBMS3*	flanking_5UTR

rs11794023	0.101	9	92704527	0.304	1.16E-04	0.672	*SYK*	flanking_3UTR
rs12094945	0.247	1	4833014	0.304	1.17E-04	0.672	*otein 1 [Source:RefSe*	downstream
rs631495	0.052	4	130679223	0.304	1.18E-04	0.672	*CDNA FLJ33703 fis*	downstream
rs10969314	0.267	9	29650792	0.303	1.18E-04	0.672	*LRRN6C*	flanking_5UTR
rs4704327	0.129	5	75871788	-0.303	1.18E-04	0.672	*ing-like protein IQGA*	intron
rs17113057	0.118	1	84874350	0.303	1.19E-04	0.674	*CDNA FLJ35487 fis*	upstream
rs4237482	0.057	10	129210364	-0.303	1.21E-04	0.677	*Source:RFAM;Acc:RF*	downstream
rs2158992	0.178	14	71625648	0.303	1.21E-04	0.677	*RGS6*	intron
rs10134725	0.109	14	33979334	0.303	1.21E-04	0.677	*C14orf147*	intron
rs10506882	0.229	12	81729283	0.309	1.22E-04	0.680	*TMTC2*	intron
rs6507699	0.175	18	42202345	0.302	1.24E-04	0.687	*RNF165*	intron
rs7118671	0.11	11	6890578	-0.304	1.25E-04	0.687	*10) (OR11-610) (HB2*	upstream
rs2703752	0.383	11	129922042	0.309	1.27E-04	0.697	---	CDS
rs2851085	0.211	11	74500993	0.303	1.28E-04	0.699	*OR2AT4*	flanking_5UTR
rs11847175	0.211	14	85073961	0.303	1.29E-04	0.699	*FLRT2*	intron
rs2144834	0.149	14	93843387	0.302	1.29E-04	0.699	*SERPINA6*	intron
rs2239802	0.211	6	32519824	0.303	1.30E-04	0.699	*HLA-DRA*	intron
rs216481	0.497	17	25893195	-0.306	1.30E-04	0.699	*GOSR1*	flanking_3UTR
rs7883256	0.418	23	6781244	0.304	1.30E-04	0.699	---	upstream
rs11170109	0.315	12	51072293	-0.305	1.31E-04	0.699	*Keratin*	upstream
rs6435958	0.087	2	217604857	0.302	1.34E-04	0.711	*ition protein 1 (STP-1*	upstream
rs13424413	0.049	2	81416160	0.301	1.35E-04	0.715	*CTNNA2*	flanking_3UTR
rs17476911	0.058	5	123022548	-0.303	1.36E-04	0.718	*CSNK1G3*	flanking_3UTR
rs12270070	0.198	11	113304257	-0.300	1.39E-04	0.728	*otor 3B precursor [So*	intron
rs7537835	0.27	1	81205753	0.302	1.41E-04	0.728	*EGF*	upstream
rs790450	0.092	12	91141234	-0.300	1.42E-04	0.728	*egulated 1 [Source:Re*	upstream
rs13427703	0.112	2	233912247	0.300	1.42E-04	0.728	*rotein) (S-AG) (Rod p*	intron
rs7890623	0.08	23	37896780	-0.300	1.42E-04	0.728	*ng protein SRPX precu*	intron
rs4965481	0.437	15	97763529	-0.300	1.43E-04	0.728	*Source:RFAM;Acc:RF*	downstream
rs10780183	0.129	9	139714736	-0.300	1.43E-04	0.728	*EHMT1*	flanking_5UTR
rs7155538	0.063	14	69563718	0.300	1.44E-04	0.728	*SMOC1*	intron
rs7907561	0.055	10	129201155	-0.300	1.44E-04	0.728	*DOCK1*	flanking_3UTR
rs10216695	0.092	8	23400348	0.300	1.45E-04	0.728	*.6.1.6) (NTPDase4) (U*	upstream
rs12212816	0.275	6	76222884	0.300	1.46E-04	0.728	*FILIP1*	intron
rs10484461	0.092	6	113834023	0.299	1.47E-04	0.728	---	downstream
rs9616027	0.098	22	45275155	0.299	1.47E-04	0.728	*CELSR1*	intron
rs1955436	0.129	14	85057070	0.299	1.47E-04	0.728	*FLRT2*	flanking_5UTR
rs630599	0.06	4	130686022	0.299	1.48E-04	0.728	---	downstream
rs613359	0.06	4	130688470	0.299	1.48E-04	0.728	*LOC132321*	flanking_3UTR
rs115125	0.276	23	28600207	0.299	1.49E-04	0.728	*intron*	N
rs11556087	0.08	19	9829139	-0.299	1.50E-04	0.728	*OLFM2*	coding
rs12252832	0.241	10	71022032	0.299	1.51E-04	0.728	*NEUROG3*	flanking_5UTR
rs11152324	0.092	18	57775955	0.299	1.51E-04	0.728	*RNF152*	flanking_5UTR
rs12949500	0.112	17	5709513	0.299	1.51E-04	0.728	*ar RNA [Source:RFAM*	upstream
rs17689237	0.127	8	94105203	0.300	1.52E-04	0.728	*RBM12B*	flanking_3UTR
rs12889429	0.333	14	85082478	-0.299	1.52E-04	0.728	*FLRT2*	intron
rs16840838	0.132	1	4101209	0.299	1.54E-04	0.728	*C1orf174*	flanking_5UTR
rs7622068	0.118	3	8945377	0.298	1.54E-04	0.728	*RAD18*	intron
rs12476246	0.408	2	220343314	0.298	1.54E-04	0.728	*SLC4A3*	flanking_3UTR
rs1439697	0.124	2	35734482	0.299	1.55E-04	0.728	*al RNA [Source:RFAM*	downstream
rs9925202	0.095	16	53286162	0.298	1.56E-04	0.728	*meobox protein 5) (H*	upstream
rs17090939	0.121	14	94009303	-0.298	1.57E-04	0.728	*SERPINA9*	intron
rs7528918	0.063	1	61591010	0.298	1.57E-04	0.728	*NFIA*	intron
rs4915737	0.063	1	61594871	0.298	1.57E-04	0.728	*ctor 1 A-type (Nucle*	intron
rs5945677	0.173	23	51454911	0.299	1.57E-04	0.728	*GSPT2*	flanking_5UTR
rs17523724	0.089	5	124713210	0.298	1.57E-04	0.728	*ZNF608*	flanking_5UTR
rs10503719	0.106	8	22673848	-0.298	1.57E-04	0.728	*PEBP4*	intron
rs4998986	0.325	18	55282713	0.298	1.57E-04	0.728	*F domains 1 [Source:I*	intron

rs763415	0.106	6	107745262	-0.298	1.60E-04	0.733	*Decaprenyl pyrophos*	intron
rs11706227	0.06	3	55471300	0.298	1.61E-04	0.733	*WNT5A*	flanking_3UTR
rs2364467	0.064	1	61588942	0.299	1.61E-04	0.733	*ictor 1 A-type (Nucleo*	intron
rs12547555	0.066	8	99283885	-0.298	1.61E-04	0.733	*NPAL2*	intron
rs10758841	0.413	9	7153938	-0.299	1.62E-04	0.733	*JMJD2C*	intron
rs7826947	0.239	8	15645158	-0.298	1.62E-04	0.733	*andidate 3 (Protein N*	intron
rs10113901	0.351	9	22364032	-0.297	1.63E-04	0.736	*- methylthioadenosir*	downstream
rs7496458	0.37	15	23322462	0.298	1.64E-04	0.742	*UBE3A*	flanking_5UTR
rs34117994	0.178	15	20670512	0.302	1.66E-04	0.747	*NIPA1*	flanking_5UTR
rs2906041	0.445	15	23333016	0.297	1.67E-04	0.748	*UBE3A*	flanking_5UTR
rs3792104	0.109	2	233886674	0.297	1.68E-04	0.752	*SAG*	intron
rs7007919	0.165	8	20691655	-0.297	1.71E-04	0.757	*LZTS1*	flanking_5UTR
rs5945540	0.257	23	143528552	-0.297	1.72E-04	0.757	*SPANX-N1*	flanking_5UTR
rs12976740	0.4	19	52615940	-0.300	1.73E-04	0.757	*MEIS3*	flanking_5UTR
rs4916827	0.204	5	90224867	0.296	1.73E-04	0.757	*omolog precursor (Ve*	intron
rs5759887	0.183	22	22204416	0.298	1.73E-04	0.757	*IGLL1*	flanking_3UTR
rs11219196	0.161	11	122929644	0.296	1.73E-04	0.757	*SCN3B*	flanking_3UTR
rs2701419	0.08	15	85377797	0.296	1.74E-04	0.757	*CDNA FLJ32310 fis*	downstream
rs1562788	0.095	3	151548593	0.296	1.75E-04	0.760	*al RNA [Source:RFAM*	downstream
rs2663095	0.175	15	40289310	-0.301	1.76E-04	0.763	*ource:miRBase 8.2;Ac*	upstream
rs2270891	0.063	9	2818742	0.296	1.77E-04	0.763	*KIAA0020*	coding
rs767638	0.18	7	68152053	0.298	1.77E-04	0.763	*AUTS2*	flanking_5UTR
rs1317877	0.104	19	51618224	0.296	1.80E-04	0.769	*CCDC8*	flanking_5UTR
rs3021103	0.198	23	38007851	-0.297	1.80E-04	0.769	*RPGR*	flanking_3UTR
rs1470704	0.124	9	105966729	0.296	1.80E-04	0.769	*Chromosome- associ*	downstream
rs2665182	0.371	5	103024363	0.295	1.81E-04	0.770	*NUDT12*	flanking_5UTR
rs8015454	0.116	14	66809038	0.297	1.82E-04	0.770	*MPP5*	intron
rs627222	0.21	7	6094981	-0.295	1.84E-04	0.771	*EIF2AK1*	flanking_5UTR
rs2391824	0.316	13	109759283	0.295	1.85E-04	0.771	*COL4A2*	intron
rs2972412	0.179	5	42503797	-0.296	1.85E-04	0.771	*GHR*	intron
rs482063	0.064	1	73714926	0.296	1.85E-04	0.771	*LRRC44*	flanking_3UTR
rs7135850	0.443	12	130049943	0.295	1.86E-04	0.771	*GPR133*	intron
rs1035680	0.42	2	220342753	0.295	1.86E-04	0.771	---	upstream
rs917629	0.451	17	25824478	-0.295	1.87E-04	0.771	*GOSR1*	flanking_5UTR
rs6614467	0.175	23	51427389	0.295	1.90E-04	0.771	*GSPT2*	flanking_5UTR
rs12190849	0.138	6	70864945	-0.295	1.90E-04	0.771	*ecursor (Collagen alpi*	intron
rs16873476	0.118	5	75875795	-0.294	1.91E-04	0.771	*ing-like protein IQGA*	intron
rs7540628	0.099	1	209958067	0.301	1.91E-04	0.771	---	downstream
rs11838565	0.069	13	21353862	0.294	1.91E-04	0.771	*FGF9*	flanking_3UTR
rs11060355	0.126	12	128504215	0.294	1.92E-04	0.771	*rane protein 132D (T*	intron
rs603512	0.33	11	81375993	0.294	1.93E-04	0.771	*nal protein S28. [Sou*	upstream
rs16868315	0.201	7	68099996	0.294	1.94E-04	0.771	*AUTS2*	flanking_5UTR
rs10884732	0.439	10	111029527	0.296	1.95E-04	0.771	*1.9) (X-Pro aminopept*	downstream
rs3134798	0.184	6	32292683	0.294	1.95E-04	0.771	*NOTCH4*	intron
rs12923969	0.261	16	5495137	0.294	1.95E-04	0.771	*EC 2.4.1.142) (GDP-m*	downstream
rs11781835	0.427	8	22663974	-0.297	1.95E-04	0.771	*PEBP4*	intron
rs17668478	0.201	3	145277060	0.294	1.96E-04	0.771	*C3orf58*	flanking_3UTR
rs4759086	0.141	12	51952980	-0.294	1.96E-04	0.771	*ESPL1*	intron
rs16996480	0.041	23	120810532	0.296	1.99E-04	0.771	*GLUD2*	flanking_3UTR
rs2347335	0.218	10	83814253	0.294	1.99E-04	0.771	*NRG3*	intron
rs9963530	0.318	18	45777320	0.302	1.99E-04	0.771	*B (Myosin Vb). [Sourc*	intron
rs17496354	0.138	9	139686757	-0.294	1.99E-04	0.771	*e-lysine N-methyltran*	intron
rs11718188	0.055	3	173338293	0.295	2.00E-04	0.771	*taining 3B [Source:Re*	intron
rs4614152	0.244	23	82802212	-0.294	2.00E-04	0.771	*POU domain*	downstream
rs2193000	0.398	15	34841535	0.295	2.00E-04	0.771	*some 15 open readin*	intron
rs2545904	0.212	19	20476510	-0.295	2.01E-04	0.771	*otein (Fragment). [Sc*	downstream
rs6123812	0.451	20	56629276	0.294	2.01E-04	0.771	*MGC4294*	flanking_5UTR
rs512332	0.057	1	33979520	-0.293	2.02E-04	0.771	*CSMD2*	intron

rs2072765	0.15	6	167637649	0.294	2.03E-04	0.771	*UNC93A*	intron
rs3809367	0.116	13	98710535	-0.295	2.03E-04	0.771	*GPR18*	flanking_5UTR
rs9544385	0.132	13	76316442	0.293	2.03E-04	0.771	*KCTD12*	flanking_3UTR
rs10429166	0.407	7	1622127	0.295	2.04E-04	0.771	*LOC260341*	5UTR
rs1157599	0.149	13	35549792	0.293	2.05E-04	0.771	*Serine*	intron
rs12457831	0.165	18	58961220	0.294	2.05E-04	0.771	*regulator Bcl-2. [Sour*	intron
rs3891932	0.52	5	71733860	0.293	2.06E-04	0.771	*main 2 [Source:RefSe*	downstream
rs2338216	0.52	5	71737570	0.293	2.06E-04	0.771	*ZNF366*	flanking_3UTR
rs4703536	0.52	5	71754604	0.293	2.06E-04	0.771	*r protein 366. [Sourc*	downstream
rs9933282	0.06	16	77061049	0.293	2.07E-04	0.771	*WWOX*	intron
rs12464951	0.413	2	220340784	0.294	2.07E-04	0.771	---	downstream
rs2167796	0.139	2	19779081	0.294	2.08E-04	0.771	*skipped-related 1. [Sc*	upstream
rs7615473	0.31	3	55825018	0.293	2.08E-04	0.771	*CAST1*	intron
rs11247090	0.474	15	97763196	-0.294	2.09E-04	0.771	*A (Serum response fa*	upstream
rs316838	0.092	1	240387283	-0.293	2.09E-04	0.771	*hospholipase D famil*	intron
rs9860951	0.293	3	55822038	0.293	2.10E-04	0.771	*protein 2. [Source:Un*	intron
rs11127125	0.42	2	27876624	-0.293	2.10E-04	0.771	*RBKS*	intron
rs487750	0.468	9	137743561	-0.296	2.11E-04	0.771	*KCNT1*	intron
rs17363868	0.126	2	179980668	-0.295	2.11E-04	0.771	---	upstream
rs2608132	0.175	3	76934931	-0.295	2.12E-04	0.772	---	upstream
rs701808	0.291	10	98948024	-0.294	2.13E-04	0.772	*SLIT1*	flanking_5UTR
rs3863105	0.17	3	170766189	0.292	2.13E-04	0.772	*MDS1*	intron
rs17462546	0.361	18	23765299	-0.293	2.14E-04	0.772	*CDH2*	flanking_3UTR
rs9553897	0.353	13	26284655	0.292	2.15E-04	0.772	*GPR12*	flanking_5UTR
rs318157	0.301	23	96995537	0.293	2.16E-04	0.772	*DIAPH2*	flanking_3UTR
rs1138030	0.264	1	55090273	0.292	2.16E-04	0.772	*DHCR24*	3UTR
rs12054750	0.471	5	71764977	0.293	2.16E-04	0.772	*ZNF366*	flanking_3UTR
rs9812483	0.289	3	135262720	0.293	2.18E-04	0.772	*SLCO2A1*	flanking_5UTR
rs10852388	0.144	16	17034602	-0.292	2.18E-04	0.772	*XYLT1*	flanking_3UTR
rs10859730	0.448	12	93477331	0.292	2.19E-04	0.772	---	downstream
rs10859731	0.448	12	93477544	0.292	2.19E-04	0.772	*TMCC3*	flanking_3UTR
rs1449692	0.491	3	29026857	0.292	2.20E-04	0.772	*RBMS3*	flanking_5UTR
rs17810074	0.197	15	63781787	0.295	2.21E-04	0.772	*DENND4A*	coding
rs11750246	0.061	5	122998714	-0.293	2.21E-04	0.772	*CSNK1G3*	flanking_3UTR
rs3745532	0.24	19	55897865	0.294	2.21E-04	0.772	*SHANK1*	intron
rs9314084	0.126	5	92717502	0.292	2.22E-04	0.772	*NR2F1*	flanking_5UTR
rs13360184	0.126	5	92718923	0.292	2.22E-04	0.772	*NR2F1*	flanking_5UTR
rs149670	0.27	14	71701970	0.292	2.22E-04	0.772	*RGS6*	intron
rs9651462	0.445	10	111032863	0.291	2.26E-04	0.783	*XPNPEP1*	flanking_3UTR
rs17734241	0.098	15	85409418	0.291	2.28E-04	0.785	*al RNA [Source:RFAM*	downstream
rs6475425	0.463	9	20276438	-0.291	2.29E-04	0.785	*l gene from chromoso*	downstream
rs12437105	0.296	14	68231084	0.291	2.29E-04	0.785	*ZFP36L1*	flanking_3UTR
rs4078253	0.489	5	112484272	0.291	2.31E-04	0.785	*ncer protein (Protein I*	intron
rs6977956	0.184	7	46350787	0.291	2.32E-04	0.785	*IGFBP3*	flanking_5UTR
rs28620466	0.064	17	1507275	0.292	2.33E-04	0.785	*PRPF8*	intron
rs1818691	0.09	17	22028052	0.292	2.33E-04	0.785	*FAM27L*	flanking_3UTR
rs6594683	0.491	5	112489540	0.291	2.34E-04	0.785	*MCC*	intron
rs220264	0.21	21	42355484	-0.291	2.34E-04	0.785	*UMODL1*	flanking_5UTR
rs11219198	0.106	11	122933015	0.290	2.34E-04	0.785	*SCN3B*	flanking_3UTR
rs3003879	0.259	6	115080503	0.290	2.35E-04	0.785	*HS3ST5*	flanking_5UTR
rs10756997	0.348	9	18905953	0.290	2.36E-04	0.785	*orf94 precursor. [Sour*	downstream
rs12267244	0.071	10	115571903	0.294	2.36E-04	0.785	---	downstream
rs2591578	0.145	5	165340287	0.291	2.36E-04	0.785	*WWC1*	flanking_5UTR
rs12534697	0.235	7	68177768	0.294	2.37E-04	0.785	*al RNA [Source:RFAM*	upstream
rs10519639	0.19	5	119933073	0.290	2.37E-04	0.785	*LOC51334*	intron
rs6574824	0.159	14	85048535	0.291	2.37E-04	0.785	---	downstream
rs206032	0.448	23	39415073	0.290	2.37E-04	0.785	*al RNA [Source:RFAM*	upstream
rs751687	0.342	8	15653267	-0.290	2.38E-04	0.785	*TUSC3*	intron

rs10815532	0.454	9	7154061	-0.290	2.38E-04	0.785	*JMJD2C*	intron
rs6575751	0.309	14	99410336	0.291	2.40E-04	0.787	*EML1*	intron
rs17385047	0.224	4	15042293	-0.290	2.40E-04	0.787	*is factor-related prote*	intron
rs475414	0.39	11	63998420	0.291	2.41E-04	0.787	*LOC439914*	flanking_3UTR
rs16999395	0.083	21	40446750	-0.290	2.41E-04	0.787	*ion molecule precurs*	intron
rs3849856	0.132	9	139693609	-0.290	2.44E-04	0.790	*e-lysine N-methyltran*	intron
rs1960498	0.187	5	42486531	-0.290	2.45E-04	0.790	*GHR*	intron
rs17121874	0.195	8	15643863	-0.290	2.47E-04	0.790	*TUSC3*	intron
rs6826415	0.17	4	1355623	0.289	2.47E-04	0.790	*KIAA1530*	intron
rs417407	0.243	5	71880756	0.290	2.48E-04	0.790	*er protein 366. [Sourc*	upstream
rs9645351	0.106	1	111696393	0.289	2.49E-04	0.790	*C1orf88*	3UTR
rs17019986	0.083	3	82771929	-0.289	2.50E-04	0.790	*al RNA [Source:RFAM*	downstream
rs3736495	0.147	15	66400544	-0.290	2.50E-04	0.790	*ITGA11*	intron
rs2833210	0.147	21	31343629	-0.289	2.50E-04	0.790	*ociated protein. [Sou*	upstream
rs2906029	0.451	15	23327956	0.289	2.52E-04	0.790	*: 3.6.3.1) (ATPVA) (Ar*	downstream
rs9575257	0.408	13	35557784	-0.289	2.53E-04	0.790	*DCAMKL1*	intron
rs2922876	0.167	8	6384104	-0.289	2.53E-04	0.790	*ANGPT2*	intron
rs547049	0.218	23	45733366	0.289	2.54E-04	0.790	*in ZNF673. [Source:U*	upstream
rs1525238	0.286	7	46752472	-0.290	2.56E-04	0.790	*TNS3*	flanking_3UTR
rs4938179	0.132	11	114538635	-0.289	2.56E-04	0.790	*with sequence simila*	downstream
rs11796525	0.204	23	86195206	0.289	2.58E-04	0.790	*DACH2*	flanking_3UTR
rs2156336	0.286	18	56268947	-0.289	2.60E-04	0.790	*MC4R*	flanking_5UTR
rs11664830	0.092	18	18142902	0.288	2.61E-04	0.790	---	downstream
rs7925656	0.078	11	107997861	0.288	2.61E-04	0.790	*'2 domains b (Exophil*	upstream
rs1857985	0.069	9	106026964	0.288	2.61E-04	0.790	*SMC2L1*	flanking_3UTR
rs7145070	0.106	14	69446603	0.288	2.63E-04	0.790	*rsor (Secreted modulc*	intron
rs1917372	0.046	3	83876104	0.289	2.64E-04	0.790	*IGSF4D*	flanking_5UTR
rs9918379	0.089	6	113836873	0.288	2.64E-04	0.790	*MARCKS*	flanking_5UTR
rs2383593	0.06	14	33921272	0.288	2.64E-04	0.790	*factor prolyl hydroxy*	upstream
rs292639	0.422	7	134596719	0.288	2.64E-04	0.790	*LOC346673*	flanking_3UTR
rs292641	0.422	7	134597284	0.288	2.64E-04	0.790	*) (E3 ubiquitin protein*	downstream
rs4665392	0.503	2	27883153	0.288	2.66E-04	0.790	*RBKS*	intron
rs17384915	0.221	4	15033274	-0.288	2.66E-04	0.790	*is factor-related prote*	intron
rs917631	0.448	17	25723655	-0.288	2.66E-04	0.790	*CPD*	flanking_5UTR
rs9913111	0.448	17	25734012	-0.288	2.66E-04	0.790	*CPD*	intron
rs12453652	0.448	17	25754986	-0.288	2.66E-04	0.790	*7.22) (Metallocarbox*	intron
rs9911455	0.448	17	25812712	-0.288	2.66E-04	0.790	*CPD*	intron
rs11651814	0.448	17	25822771	-0.288	2.66E-04	0.790	*CPD*	flanking_3UTR
rs7613439	0.057	3	74525213	0.288	2.67E-04	0.790	*CNTN3*	intron
rs17445240	0.095	2	3680916	-0.288	2.68E-04	0.790	*ALLC*	flanking_5UTR
rs238924	0.454	11	113480656	0.288	2.68E-04	0.790	*nc finger protein PLZF*	intron
rs4280976	0.279	6	141599266	0.288	2.72E-04	0.790	*NMBR*	flanking_3UTR
rs17111100	0.27	1	55080281	0.288	2.73E-04	0.790	*77 protein. [Source:L*	3UTR
rs11840027	0.063	13	21341633	0.287	2.74E-04	0.790	*U36*	upstream
rs6947410	0.184	7	21410241	0.287	2.74E-04	0.790	*SP4*	flanking_5UTR
rs17141598	0.081	16	6862579	0.288	2.74E-04	0.790	*A2BP1*	intron
rs17126889	0.098	1	64929450	0.288	2.75E-04	0.790	*CACHD1*	intron
rs7612441	0.147	3	66730852	-0.287	2.75E-04	0.790	*LRIG1*	flanking_5UTR
rs10862511	0.175	12	81725727	0.287	2.76E-04	0.790	*TMTC2*	intron
rs7901983	0.069	10	1389941	0.287	2.76E-04	0.790	*IA adenosine deamin*	intron
rs7610284	0.417	3	13655862	-0.287	2.77E-04	0.790	*FBLN2*	flanking_3UTR
rs10877258	0.109	12	57961635	0.287	2.78E-04	0.790	*LRIG3*	flanking_5UTR
rs3748303	0.181	12	15196428	-0.287	2.78E-04	0.790	*RERG*	intron
rs149667	0.267	14	71705190	0.287	2.79E-04	0.790	*ntron*	NM_004296.3
rs6996349	0.486	8	16655368	-0.287	2.80E-04	0.790	*FGF20*	flanking_3UTR
rs2277533	0.158	15	40352880	-0.287	2.82E-04	0.790	*TMEM87A*	coding
rs10462377	0.523	5	71734820	0.289	2.82E-04	0.790	*er protein 366. [Sourc*	downstream
rs8088371	0.106	18	7245661	-0.287	2.82E-04	0.790	---	downstream

rs1018250	0.106	18	7245921	-0.287	2.82E-04	0.790	*LAMA1*	flanking_5UTR
rs6994352	0.236	8	19370033	0.287	2.83E-04	0.790	*Chondroitin beta-1*	intron
rs9916242	0.448	17	25788277	-0.289	2.83E-04	0.790	*CPD*	intron
rs11206451	0.273	1	55084040	0.287	2.83E-04	0.790	*C1orf177*	flanking_3UTR
rs7598661	0.319	2	47322459	-0.287	2.83E-04	0.790	*CALM2*	flanking_5UTR
rs10778912	0.176	12	81725746	0.288	2.84E-04	0.790	*epeat containing 2 [S*	intron
rs17245080	0.204	23	45718766	0.287	2.85E-04	0.790	*ZNF673*	flanking_5UTR
rs506963	0.204	23	45723890	0.287	2.85E-04	0.790	*ZNF673*	flanking_5UTR
rs571167	0.204	23	45726335	0.287	2.85E-04	0.790	---	upstream
rs847593	0.204	23	45727357	0.287	2.85E-04	0.790	*ZNF673*	flanking_5UTR
rs548533	0.204	23	45728960	0.287	2.85E-04	0.790	*in ZNF673. [Source:U.*	upstream
rs479554	0.204	23	45737567	0.287	2.85E-04	0.790	*ZNF673*	flanking_5UTR
rs527850	0.204	23	45755780	0.287	2.85E-04	0.790	*ZNF673*	flanking_5UTR
rs2940919	0.176	5	42506487	-0.288	2.85E-04	0.790	*s growth hormone re*	intron
rs7701546	0.066	5	43258083	0.288	2.86E-04	0.790	*MGC42105*	intron
rs6463347	0.327	7	46747139	-0.288	2.86E-04	0.790	*TNS3*	flanking_3UTR
rs12710619	0.27	23	141092467	0.287	2.87E-04	0.790	*GE-E1 antigen) (Hepc*	downstream
rs7675761	0.25	4	15136906	-0.287	2.87E-04	0.790	*FBXL5*	flanking_3UTR
rs4698395	0.25	4	15139222	-0.287	2.87E-04	0.790	*FBXL5*	flanking_3UTR
rs922369	0.244	10	71020137	0.287	2.87E-04	0.790	*NEUROG3*	flanking_5UTR
rs6887169	0.066	5	43243995	0.287	2.87E-04	0.790	*MGC42105*	intron
rs10473304	0.066	5	43279531	0.287	2.87E-04	0.790	*MGC42105*	intron
rs4459870	0.305	3	55804100	0.287	2.88E-04	0.790	*CAST1*	intron
rs11967511	0.109	6	107703467	-0.287	2.88E-04	0.790	*PDSS2*	intron
rs12827908	0.17	12	76556949	-0.292	2.88E-04	0.790	*NAV3*	flanking_5UTR
rs9512776	0.135	13	27119687	-0.286	2.90E-04	0.792	*MGC9850*	flanking_5UTR
rs2991957	0.23	1	232559638	0.286	2.92E-04	0.792	*C1orf31*	flanking_5UTR
rs3001702	0.23	1	232560309	0.286	2.92E-04	0.792	*C1orf31*	flanking_5UTR
rs2608131	0.17	3	76932137	-0.286	2.95E-04	0.792	*al RNA [Source:RFAM*	upstream
rs1397854	0.057	20	40898462	0.286	2.96E-04	0.792	*PTPRT*	intron
rs11651917	0.129	17	7559329	0.286	2.96E-04	0.792	*EFNB3*	flanking_3UTR
rs2663006	0.149	10	132451675	-0.286	2.96E-04	0.792	*TCERG1L*	flanking_3UTR
rs10509320	0.108	10	71021017	0.288	2.97E-04	0.792	*NEUROG3*	flanking_5UTR
rs3096702	0.221	6	32300309	0.286	2.98E-04	0.792	*NOTCH4*	flanking_5UTR
rs6140212	0.184	20	7147917	-0.286	2.98E-04	0.792	*BMP2*	flanking_3UTR
rs13197839	0.118	6	66046013	0.287	2.98E-04	0.792	*EGFL11*	flanking_3UTR
rs17030997	0.092	2	105486310	-0.286	2.98E-04	0.792	*l muscle LIM- protein*	upstream
rs7016352	0.445	8	99331621	-0.286	2.99E-04	0.792	*NPAL2*	intron
rs13218560	0.064	6	10683332	0.287	2.99E-04	0.792	*cetyllactosaminide be*	intron
rs7659024	0.379	4	155740380	-0.286	2.99E-04	0.792	*FGG*	flanking_3UTR
rs1391349	0.186	16	17075737	-0.288	2.99E-04	0.792	*XYLT1*	flanking_3UTR
rs2881641	0.083	15	85343016	0.286	3.00E-04	0.792	*TMEM83*	flanking_5UTR
rs123241	0.276	14	71711002	0.286	3.00E-04	0.792	*signaling 6 (RGS6) (S*	intron
rs9289815	0.121	3	151712648	0.286	3.01E-04	0.792	*SERP1*	flanking_3UTR
rs277743	0.075	9	88934539	0.286	3.01E-04	0.792	*FLJ45537*	flanking_5UTR
rs9356453	0.259	6	166580723	0.286	3.01E-04	0.792	*al RNA [Source:RFAM*	downstream
rs10516057	0.218	5	168418548	0.285	3.03E-04	0.795	*SLIT3*	intron
rs2604270	0.078	9	76115503	-0.285	3.04E-04	0.795	*AR-related orphan re*	upstream
rs2940918	0.178	5	42506342	-0.288	3.04E-04	0.795	*totropin receptor) [Co*	intron
rs800478	0.161	4	24469625	0.285	3.05E-04	0.795	*DKFZp761B107*	intron
rs2160891	0.239	12	67163504	0.285	3.06E-04	0.795	---	downstream
rs340304	0.184	3	160054939	-0.285	3.06E-04	0.795	*al RNA [Source:RFAM*	upstream
rs2570135	0.083	15	85401990	0.285	3.07E-04	0.795	*CDNA FLJ32310 fis*	downstream
rs4128334	0.411	23	6776390	0.285	3.09E-04	0.798	*HDHD1A*	flanking_3UTR
rs8084	0.388	6	32519013	0.285	3.10E-04	0.798	*[73/208]*	NM_019111.3
rs13286966	0.152	9	25739378	0.285	3.10E-04	0.798	*e 1 [Source:RefSeq_pe*	upstream
rs1922679	0.126	5	112665653	-0.291	3.10E-04	0.798	*ncer protein (Protein I*	intron
rs6034677	0.331	20	1689300	0.287	3.13E-04	0.801	*SIRPB2*	flanking_5UTR

rs11585832	0.08	1	15442409	0.285	3.13E-04	0.801	*TMEM51*	flanking_3UTR
rs13361398	0.046	5	157453665	0.286	3.13E-04	0.801	*ENTH*	flanking_5UTR
rs206029	0.411	23	39413124	0.285	3.14E-04	0.801	*nition particle RNA [S*	downstream
rs1058412	0.247	4	15122245	-0.285	3.16E-04	0.802	*C1QTNF7*	flanking_3UTR
rs9303633	0.382	17	25831576	-0.285	3.16E-04	0.802	*GOSR1*	intron
rs441342	0.272	6	25131168	0.285	3.17E-04	0.802	*LOC134997*	flanking_3UTR
rs10063718	0.139	5	119907194	0.285	3.18E-04	0.802	*n DSC54 [Source:RefS*	intron
rs2370779	0.354	11	122164901	-0.287	3.19E-04	0.802	*STS-1*	intron
rs10818803	0.236	9	125096285	0.285	3.19E-04	0.802	*STRBP*	flanking_5UTR
rs3123221	0.463	10	133068612	0.284	3.19E-04	0.802	*TCERG1L*	flanking_5UTR
rs11086650	0.451	20	56616662	-0.284	3.20E-04	0.802	*MGC4294*	flanking_5UTR
rs1411103	0.144	9	105894779	0.284	3.22E-04	0.802	*SMC2L1*	flanking_5UTR
rs2963764	0.112	5	114724034	0.284	3.22E-04	0.802	*MGC39633*	flanking_5UTR
rs2748276	0.177	6	92070901	0.286	3.23E-04	0.802	*MAP3K7*	flanking_5UTR
rs812590	0.195	12	62893749	-0.284	3.24E-04	0.802	*FLJ32549*	intron
rs17423790	0.106	7	70911538	-0.284	3.24E-04	0.802	*n-binding protein CaE*	intron
rs9512781	0.049	13	27139123	-0.284	3.24E-04	0.802	*NA-directed RNA pol*	3UTR
rs17074413	0.124	5	171558715	0.284	3.25E-04	0.802	*STK10*	flanking_5UTR
rs2476953	0.179	10	105742493	0.290	3.25E-04	0.802	*SLK*	intron
rs16946023	0.052	12	114593606	0.286	3.25E-04	0.802	---	upstream
rs1554513	0.402	3	131986946	-0.284	3.26E-04	0.802	*PIK3R4*	flanking_5UTR
rs317806	0.175	18	53401262	-0.284	3.27E-04	0.802	*FECH*	intron
rs10747678	0.287	12	52363954	-0.284	3.27E-04	0.802	*ctivator [Source:RefS*	downstream
rs2818064	0.336	6	156617103	0.284	3.27E-04	0.802	---	downstream
rs13308932	0.057	7	48803331	0.284	3.28E-04	0.802	*ATP binding cassette*	downstream
rs11639680	0.195	16	5500905	0.284	3.32E-04	0.802	*FAM86A*	flanking_5UTR
rs12409730	0.092	1	206622430	-0.284	3.33E-04	0.802	*Semaphorin receptor*	upstream
rs9862920	0.179	3	118427770	-0.287	3.34E-04	0.802	*al RNA [Source:RFAM*	upstream
rs10796221	0.126	10	14879077	0.286	3.35E-04	0.802	*n FAM107B. [Source:U*	upstream
rs964327	0.132	2	179952722	-0.283	3.36E-04	0.802	*ZNF533*	flanking_3UTR
rs7229495	0.063	18	46949805	0.283	3.36E-04	0.802	*taining 2 [Source:Ref*	downstream
rs2716054	0.479	6	95949165	-0.290	3.37E-04	0.802	*mannosidase*	upstream
rs12401133	0.416	23	6786197	0.285	3.37E-04	0.802	---	upstream
rs1354056	0.172	3	116076660	0.283	3.37E-04	0.802	*ZBTB20*	intron
rs583173	0.261	3	174644606	0.283	3.37E-04	0.802	*NLGN1*	intron
rs6427405	0.207	1	156106977	0.283	3.37E-04	0.802	*CD5L*	flanking_5UTR
rs3755573	0.494	3	99971701	0.284	3.37E-04	0.802	*ST3GAL6*	intron
rs7723734	0.474	5	71753361	0.283	3.38E-04	0.802	*ZNF366*	flanking_3UTR
rs8004126	0.322	14	35986611	-0.283	3.38E-04	0.802	*TITF1*	flanking_3UTR
rs489286	0.325	1	158989174	0.283	3.39E-04	0.802	*SLAMF7*	intron
rs2066861	0.374	4	155746886	-0.283	3.40E-04	0.802	*FGG*	intron
rs6685332	0.103	1	6031760	0.283	3.40E-04	0.802	*KCNAB2*	intron
rs2778624	0.39	9	124248882	-0.284	3.41E-04	0.802	*OR1J1*	flanking_3UTR
rs17792460	0.168	6	100870643	-0.284	3.43E-04	0.802	*ded homolog 1. [Sour*	downstream
rs220265	0.213	21	42355652	-0.283	3.43E-04	0.802	*UMODL1*	flanking_5UTR
rs17480167	0.046	8	111423198	0.284	3.43E-04	0.802	---	upstream
rs6521165	0.197	23	38000429	-0.284	3.44E-04	0.802	---	downstream
rs6945041	0.172	7	12654296	-0.283	3.44E-04	0.802	*SCIN*	intron
rs2906037	0.388	15	23331399	0.283	3.44E-04	0.802	*UBE3A*	flanking_5UTR
rs17327979	0.055	3	55440696	0.283	3.46E-04	0.802	*WNT5A*	flanking_3UTR
rs3942404	0.165	18	2320828	0.284	3.49E-04	0.802	*[Source:RFAM;Acc:R*	upstream
rs2066865	0.382	4	155744726	-0.283	3.49E-04	0.802	*FGG*	flanking_3UTR
rs11770570	0.055	7	103757746	0.283	3.50E-04	0.802	*LHFPL3*	intron
rs9860302	0.284	3	151164384	0.283	3.52E-04	0.802	*PFN2*	flanking_3UTR
rs11236673	0.136	11	75655493	0.283	3.52E-04	0.802	*nt-11 precursor. [Sour*	upstream
rs11616269	0.127	13	98904888	0.283	3.54E-04	0.802	*TM9SF2*	flanking_5UTR
rs150802	0.198	21	42359237	-0.282	3.54E-04	0.802	*UMODL1*	intron
rs9373298	0.261	6	141561523	0.282	3.55E-04	0.802	*Cbp*	upstream

rs16928560	0.239	10	72682090	0.282	3.55E-04	0.802	*UNC5B*	intron
rs1587859	0.129	5	174500477	0.282	3.56E-04	0.802	*amine receptor. [Sour*	downstream
rs716410	0.236	6	70856213	-0.282	3.56E-04	0.802	*COL19A1*	intron
rs10174187	0.509	2	129917395	-0.282	3.57E-04	0.802	*LOC151121*	flanking_5UTR
rs13162305	0.135	5	12175698	-0.282	3.57E-04	0.802	*al RNA [Source:RFAM*	downstream
rs10514055	0.072	18	68628407	0.283	3.57E-04	0.802	*pecific transmembra*	intron
rs4511535	0.201	16	5506393	0.282	3.58E-04	0.802	*FAM86A*	flanking_5UTR
rs188431	0.236	5	168488444	0.282	3.61E-04	0.802	*SLIT3*	intron
rs12154354	0.142	7	64540471	-0.284	3.61E-04	0.802	*ns zinc finger protein*	downstream
rs9821742	0.086	3	134588539	0.282	3.62E-04	0.802	*protein 108 precursor*	intron
rs10827735	0.052	10	19976155	0.284	3.62E-04	0.802	---	upstream
rs695021	0.092	1	240394845	-0.282	3.63E-04	0.802	*hospholipase D famil*	intron
rs6963748	0.118	7	18632742	-0.282	3.63E-04	0.802	*HDAC9*	intron
rs1955011	0.451	11	81358036	-0.284	3.64E-04	0.802	*MGC33846*	flanking_3UTR
rs2589010	0.338	16	53780647	0.283	3.65E-04	0.802	*IRX6*	flanking_5UTR
rs1875173	0.26	10	2961405	-0.283	3.65E-04	0.802	---	downstream
rs1916109	0.406	4	182147920	0.290	3.65E-04	0.802	---	upstream
rs4589454	0.149	14	47771144	0.282	3.66E-04	0.802	*MAMDC1*	flanking_5UTR
rs5952979	0.198	23	43707575	0.282	3.66E-04	0.802	*Norrie disease protein*	intron
rs16880576	0.132	7	42345173	-0.282	3.67E-04	0.802	---	downstream
rs1875171	0.261	10	2961075	-0.282	3.68E-04	0.802	*ctokinase 1) (Phospho*	upstream
rs2454829	0.261	10	2962144	-0.282	3.68E-04	0.802	*PFKP*	flanking_5UTR
rs2936896	0.261	10	2962261	-0.282	3.68E-04	0.802	*PFKP*	flanking_5UTR
rs3003514	0.261	10	2963425	-0.282	3.68E-04	0.802	*PFKP*	flanking_5UTR
rs10914477	0.129	1	32012849	-0.282	3.68E-04	0.802	*BAI2*	flanking_5UTR
rs6502743	0.521	17	3617632	-0.285	3.69E-04	0.802	*gen) (HML-1 antigen)*	intron
rs9454352	0.072	6	68658126	0.282	3.70E-04	0.802	*BAI3*	flanking_5UTR
rs7948668	0.417	11	5213223	-0.282	3.70E-04	0.802	*HBD*	flanking_5UTR
rs4652109	0.075	1	173936824	0.282	3.70E-04	0.802	*TN-R) (Restrictin) (Jar*	intron
rs2137338	0.23	4	149511914	-0.282	3.70E-04	0.802	*coid receptor (MR). [S*	intron
rs4666022	0.477	2	27911327	-0.283	3.71E-04	0.802	*RBKS*	intron
rs12507760	0.098	4	156961715	0.282	3.71E-04	0.802	*de-sensitive cation ch*	downstream
rs13389204	0.42	2	220314253	0.281	3.72E-04	0.802	---	downstream
rs1375668	0.365	8	6384278	-0.281	3.72E-04	0.802	*ANGPT2*	intron
rs4655977	0.052	1	87858740	0.281	3.73E-04	0.802	*LMO4*	flanking_3UTR
rs2758988	0.362	10	77758077	-0.281	3.74E-04	0.802	---	intron
rs456801	0.399	5	75979013	0.281	3.75E-04	0.802	*ing-like protein IQGA*	intron
rs5768857	0.126	22	45279126	0.281	3.75E-04	0.802	*CELSR1*	intron
rs7984436	0.474	13	35594255	0.282	3.76E-04	0.802	*Serine*	intron
rs11625757	0.106	14	97762509	0.281	3.77E-04	0.802	*FLJ25773*	flanking_5UTR
rs7155597	0.106	14	97772948	0.281	3.77E-04	0.802	*CDNA FLJ46540 fis*	upstream
rs12880106	0.106	14	97782974	0.281	3.77E-04	0.802	---	downstream
rs1171004	0.14	13	35505771	0.287	3.77E-04	0.802	*Serine*	intron
rs12807555	0.101	11	75647281	0.281	3.77E-04	0.802	*nt-11 precursor. [Sour*	upstream
rs10062601	0.172	5	119913201	0.281	3.77E-04	0.802	*LOC51334*	intron
rs10076191	0.172	5	119916753	0.281	3.77E-04	0.802	*n DSC54 [Source:RefS*	intron
rs10077688	0.172	5	119920805	0.281	3.77E-04	0.802	*LOC51334*	intron
rs17187619	0.175	18	20303578	0.281	3.77E-04	0.802	*HRH4*	intron
rs7416912	0.256	1	15067149	-0.281	3.78E-04	0.802	*KIAA1026*	intron
rs340280	0.184	3	160068225	-0.281	3.79E-04	0.802	*MFSD1*	flanking_3UTR
rs2182729	0.474	9	18907347	0.284	3.79E-04	0.802	*orf94 precursor. [Sour*	downstream
rs10887056	0.352	10	123790540	0.283	3.79E-04	0.802	*taining protein 2 (An*	intron
rs766231	0.368	2	37921097	0.281	3.79E-04	0.802	*FAM82A*	flanking_5UTR
rs6916409	0.297	6	141533106	0.283	3.81E-04	0.804	*Cbp*	upstream
rs3829594	0.382	17	25866664	-0.282	3.82E-04	0.804	*GOSR1*	intron
rs8096865	0.034	18	8420248	0.281	3.83E-04	0.804	---	upstream
rs5989502	0.385	23	6758340	0.281	3.83E-04	0.804	---	upstream
rs17031034	0.096	2	105492975	-0.288	3.83E-04	0.804	*l muscle LIM- protein*	upstream

rs4652572	0.066	1	179394829	0.281	3.84E-04	0.804	*IER5*	flanking_3UTR
rs11709926	0.295	3	185696501	-0.281	3.88E-04	0.806	*EPHB3*	flanking_5UTR
rs10056788	0.064	5	43268394	0.281	3.89E-04	0.806	*MGC42105*	intron
rs4568365	0.218	5	12465666	-0.281	3.90E-04	0.806	*CTNND2*	flanking_5UTR
rs913588	0.339	9	7164673	-0.280	3.90E-04	0.806	*JMJD2C*	coding
rs9460467	0.052	6	20300352	0.280	3.91E-04	0.806	*E2F3*	flanking_5UTR
rs12504091	0.177	4	171576394	0.282	3.91E-04	0.806	*AADAT*	flanking_5UTR
rs4750990	0.443	10	130378016	-0.280	3.91E-04	0.806	*MKI67*	flanking_5UTR
rs7016814	0.198	8	99269746	-0.280	3.91E-04	0.806	*NPAL2*	flanking_3UTR
rs507151	0.224	11	78273088	0.280	3.92E-04	0.806	*CDNA FLJ31969 fis*	intron
rs17145208	0.148	7	67306086	0.285	3.92E-04	0.806	*RSAFD1*	flanking_3UTR
rs3797418	0.101	5	75971170	-0.280	3.93E-04	0.806	*IQGAP2*	intron
rs2477354	0.25	1	194505991	-0.280	3.94E-04	0.806	*potassium channel*	intron
rs12058062	0.25	1	194523121	-0.280	3.94E-04	0.806	*KCNT2*	intron
rs1487415	0.19	5	22770723	0.280	3.94E-04	0.806	*CDH12*	intron
rs7526951	0.188	1	238886122	0.281	3.94E-04	0.806	*GREM2*	flanking_5UTR
rs17827984	0.064	16	83226308	0.282	3.95E-04	0.807	*CDNA FLJ12543 fis*	upstream
rs4245088	0.416	11	129921416	0.282	3.96E-04	0.808	*ADAMTS15*	flanking_3UTR
rs1884009	0.373	14	85057358	0.281	3.98E-04	0.809	*FLRT2*	flanking_5UTR
rs7541727	0.09	1	158198943	-0.281	3.98E-04	0.809	*SLAMF9*	flanking_5UTR
rs7331892	0.279	13	35551923	0.280	3.99E-04	0.811	*Serine*	intron
rs4965475	0.379	15	97749889	-0.280	4.00E-04	0.812	*-binding domain-con*	downstream
rs3021107	0.194	23	38001813	-0.281	4.01E-04	0.812	*ıentosa GTPase regul*	downstream
rs7242523	0.324	18	32344958	0.281	4.02E-04	0.813	*FHOD3*	intron
rs914968	0.239	6	169905116	0.280	4.04E-04	0.813	*C6orf70*	intron
rs305055	0.193	15	67880118	0.280	4.04E-04	0.813	*lar RNA [Source:RFAI*	upstream
rs300969	0.318	5	119906825	0.281	4.04E-04	0.813	*LOC51334*	intron
rs8064091	0.121	16	17001399	0.281	4.05E-04	0.813	---	downstream
rs2208993	0.442	1	3565693	0.281	4.05E-04	0.813	*scription factor) (p53-*	intron
rs11119499	0.216	1	208677939	0.280	4.06E-04	0.813	*-) (Hedgehog acyltra*	intron
rs2770730	0.244	9	7168410	0.280	4.09E-04	0.813	*3C (EC 1.14.11.-) (Jun*	downstream
rs12911343	0.221	15	91592091	-0.279	4.11E-04	0.813	*UNQ9370*	flanking_3UTR
rs12335706	0.121	9	20051257	0.284	4.11E-04	0.813	*d gene from chromos*	downstream
rs1061293	0.106	8	23345846	0.279	4.11E-04	0.813	*.6.1.6) (NTPDase4) (U*	intron
rs2272643	0.106	8	23350337	0.279	4.11E-04	0.813	*ENTPD4*	intron
rs4571739	0.106	8	23350971	0.279	4.11E-04	0.813	*.6.1.6) (NTPDase4) (U*	intron
rs17372842	0.061	7	113946478	-0.280	4.12E-04	0.813	*FOXP2*	intron
rs6870586	0.124	5	20390789	0.279	4.12E-04	0.813	*CDNA FLJ41314 fis*	upstream
rs11080125	0.437	17	25737852	-0.279	4.12E-04	0.813	*CPD*	intron
rs11717356	0.164	3	196051423	-0.279	4.13E-04	0.813	*2 of Q8NBI6 [Source.*	downstream
rs4703872	0.512	5	71759400	0.280	4.14E-04	0.813	*ZNF366*	flanking_3UTR
rs11700304	0.46	20	42007776	-0.279	4.14E-04	0.813	*C20orf100*	intron
rs4812774	0.46	20	42012602	-0.279	4.14E-04	0.813	*C20orf100*	intron
rs12617524	0.09	2	138184853	0.280	4.14E-04	0.813	---	downstream
rs9304393	0.239	18	45786213	0.279	4.16E-04	0.813	*ACAA2*	flanking_5UTR
rs7716543	0.471	5	100693456	-0.279	4.17E-04	0.813	*ST8SIA4*	flanking_5UTR
rs7030223	0.244	9	4073297	0.279	4.18E-04	0.813	*GLIS3*	intron
rs10814831	0.244	9	4073863	0.279	4.18E-04	0.813	*GLIS3*	intron
rs1395118	0.166	6	100897988	-0.281	4.18E-04	0.813	*SIM1*	flanking_3UTR
rs739195	0.444	22	47916630	-0.282	4.18E-04	0.813	*CDNA FLJ44385 fis*	upstream
rs1957509	0.316	14	99403615	0.279	4.19E-04	0.813	*d protein-like 1 (EMA*	intron
rs9916609	0.462	17	3619263	0.281	4.19E-04	0.813	*ITGAE*	intron
rs12194223	0.233	6	70850863	-0.279	4.20E-04	0.813	*COL19A1*	intron
rs316871	0.096	1	240409507	-0.281	4.20E-04	0.813	*hospholipase D famil*	intron
rs10519731	0.066	5	122969603	-0.279	4.21E-04	0.814	*na-3 (EC 2.7.11.1) (Ck*	intron
rs1516342	0.152	3	147906	0.279	4.23E-04	0.815	*protein precursor (C*	upstream
rs2021966	0.443	6	132192132	-0.279	4.23E-04	0.815	*ENPP1*	intron
rs12712963	0.273	2	46148485	0.279	4.25E-04	0.816	*PRKCE*	intron

rs10937658	0.179	4	5733712	0.280	4.25E-04	0.816	*EVC2*	intron
rs13296028	0.129	9	139680365	-0.279	4.25E-04	0.816	*EHMT1*	flanking_5UTR
rs7984342	0.273	13	109255241	0.279	4.26E-04	0.816	*[Source:RFAM;Acc:R*	upstream
rs17683343	0.233	7	68173340	0.279	4.28E-04	0.817	*AUTS2*	flanking_5UTR
rs1024614	0.217	5	12456285	-0.279	4.28E-04	0.817	*emal beta dynein hea*	downstream
rs9292826	0.109	5	41864426	0.279	4.29E-04	0.817	*OXCT1*	intron
rs7723992	0.109	5	41889271	0.279	4.29E-04	0.817	*OXCT1*	intron
rs12401342	0.063	1	57566536	0.278	4.32E-04	0.818	*DAB1*	intron
rs2613557	0.445	6	95952687	-0.278	4.32E-04	0.818	*MANEA*	flanking_5UTR
rs6735174	0.087	2	217608525	0.279	4.33E-04	0.818	*TNP1*	flanking_5UTR
rs7442772	0.17	5	119907821	0.278	4.33E-04	0.818	*LOC51334*	intron
rs2276408	0.158	11	122184121	0.278	4.33E-04	0.818	*STS-1*	intron
rs5997507	0.061	22	28455596	0.279	4.34E-04	0.818	*CABP7*	3UTR
rs517857	0.322	1	117008588	-0.278	4.34E-04	0.818	*IGSF3*	intron
rs856565	0.322	7	46688379	-0.278	4.35E-04	0.818	*TNS3*	flanking_3UTR
rs7350095	0.049	8	111422115	0.278	4.35E-04	0.818	*KCNV1*	flanking_5UTR
rs6538508	0.335	12	93474460	0.282	4.38E-04	0.820	*TMCC3*	flanking_3UTR
rs10936700	0.249	3	172989392	0.279	4.38E-04	0.820	*PLD1*	flanking_5UTR
rs1998712	0.276	1	194454288	-0.278	4.38E-04	0.821	*KCNT2*	flanking_3UTR
rs6445401	0.44	3	64371802	0.278	4.40E-04	0.821	*ADAMTS9*	flanking_3UTR
rs12291405	0.052	11	25376955	0.278	4.40E-04	0.821	*LUZP2*	flanking_3UTR
rs1441990	0.497	8	130075654	0.278	4.40E-04	0.821	---	upstream
rs1531206	0.463	9	20271390	-0.278	4.42E-04	0.821	*d gene from chromoso*	downstream
rs4955878	0.136	3	55780487	0.279	4.42E-04	0.821	*protein 2. [Source:Un*	intron
rs11870030	0.301	17	11494598	0.279	4.43E-04	0.821	*onemal beta dynein h*	intron
rs7847107	0.431	9	96506846	-0.279	4.44E-04	0.821	*P00000021697. [Sour*	intron
rs4392391	0.422	3	44247970	-0.278	4.44E-04	0.821	*CDNA FLJ36157 fis*	upstream
rs6479562	0.333	9	96468981	0.278	4.44E-04	0.821	*FBP1*	flanking_5UTR
rs1177982	0.356	17	67983932	0.278	4.45E-04	0.821	*SLC39A11*	flanking_3UTR
rs6638626	0.41	23	6784755	0.279	4.46E-04	0.821	*HDHD1A*	flanking_3UTR
rs9893947	0.135	17	31390662	0.278	4.46E-04	0.821	*CCL23*	flanking_5UTR
rs838612	0.336	3	144661465	-0.278	4.47E-04	0.821	*Sodium*	intron
rs9919356	0.276	10	121817505	0.278	4.48E-04	0.821	*ting protein (p125). [S*	downstream
rs1610314	0.256	6	166581994	0.279	4.49E-04	0.821	*MGC35308*	flanking_3UTR
rs9956108	0.109	18	3032416	0.278	4.49E-04	0.821	*RNA U70 [Source:RFA*	upstream
rs4916698	0.132	5	90274891	0.278	4.49E-04	0.821	*MASS1*	intron
rs264234	0.413	18	10964448	0.279	4.50E-04	0.821	*C18orf58*	flanking_5UTR
rs4919944	0.27	21	42058834	0.278	4.50E-04	0.821	*RIPK4*	intron
rs7080187	0.115	10	16294375	-0.278	4.52E-04	0.821	*al RNA [Source:RFAM*	upstream
rs1453556	0.338	15	22617453	-0.278	4.52E-04	0.821	*SNRPN*	flanking_5UTR
rs2723696	0.325	4	169788117	-0.278	4.52E-04	0.821	*PALLD*	intron
rs2160565	0.194	16	48434603	0.278	4.52E-04	0.821	*f69 protein. [Source:U*	upstream
rs373889	0.25	3	198458580	-0.285	4.54E-04	0.822	*ssociated protein 97,*	intron
rs10519606	0.289	5	119076715	-0.280	4.55E-04	0.822	*LOC340069*	flanking_3UTR
rs11217082	0.08	11	98192714	0.277	4.55E-04	0.822	---	downstream
rs11063679	0.075	12	5422387	0.277	4.56E-04	0.822	*NTF3*	flanking_5UTR
rs4828042	0.453	23	99822513	-0.279	4.57E-04	0.822	*SYTL4*	intron
rs8031801	0.181	15	59056739	-0.285	4.57E-04	0.822	*RORA*	intron
rs4905908	0.322	14	99399799	0.277	4.59E-04	0.822	*EML1*	intron
rs12524627	0.098	6	109507738	0.278	4.59E-04	0.822	*SESN1*	intron
rs2842169	0.066	10	128320703	-0.277	4.60E-04	0.822	*C10orf90*	flanking_5UTR
rs2471282	0.04	7	139621497	-0.277	4.62E-04	0.822	*SLC37A3*	flanking_3UTR
rs316823	0.092	1	240422651	-0.277	4.63E-04	0.822	*hospholipase D famil*	intron
rs402098	0.092	1	240430321	-0.277	4.63E-04	0.822	*hospholipase D famil*	intron
rs10758488	0.163	9	3830625	0.279	4.64E-04	0.822	*similar 3) (Zinc finger*	intron
rs12488085	0.307	3	55809911	0.277	4.64E-04	0.822	*CAST1*	intron
rs465731	0.434	5	75986463	0.277	4.65E-04	0.822	*IQGAP2*	intron
rs6885304	0.055	5	174436411	0.285	4.67E-04	0.822	*FLJ16171*	flanking_5UTR

rs2770729	0.241	9	7168197	0.279	4.68E-04	0.822	*JMJD2C*	flanking_3UTR
rs800930	0.113	1	69899195	0.282	4.70E-04	0.822	*rotein 7 (Protein LAP*	intron
rs210564	0.204	23	151030164	0.277	4.71E-04	0.822	*ıntigen 5 (MAGE-5 aı*	downstream
rs6424732	0.293	1	81227921	0.277	4.72E-04	0.822	*EGF*	upstream
rs7309273	0.376	12	4885642	-0.277	4.73E-04	0.822	*6 (Voltage-gated pot*	downstream
rs2719634	0.158	16	10643239	-0.277	4.73E-04	0.822	*CDNA FLJ32871 fis*	intron
rs11844745	0.239	14	76734084	0.277	4.74E-04	0.822	*63C [Source:RefSeq_ı*	intron
rs1851566	0.374	4	189882563	0.277	4.74E-04	0.822	---	upstream
rs7793253	0.431	7	21365299	-0.277	4.74E-04	0.822	*SP4*	flanking_5UTR
rs431838	0.272	2	76153566	-0.277	4.75E-04	0.822	*or (GCF) (Transcriptic*	upstream
rs8009476	0.27	14	35987213	-0.277	4.75E-04	0.822	*TITF1*	flanking_3UTR
rs149660	0.275	14	71709943	0.277	4.76E-04	0.822	*RGS6*	intron
rs6918658	0.486	6	5662396	-0.277	4.77E-04	0.822	*ıylalanyl-tRNA synthe*	intron
rs9643245	0.356	8	130248160	0.276	4.77E-04	0.822	*CCDC26*	flanking_3UTR
rs2710201	0.225	7	152555826	0.277	4.77E-04	0.822	*FLJ42291*	flanking_3UTR
rs4936140	0.411	11	130978044	0.276	4.78E-04	0.822	*precursor (hNT). [Soı*	intron
rs12376178	0.057	9	134390949	0.276	4.79E-04	0.822	*FLJ46082*	intron
rs798854	0.098	7	40056893	-0.276	4.79E-04	0.822	*CDC2L5*	intron
rs6569077	0.468	6	98319130	-0.279	4.79E-04	0.822	*C6orf167*	flanking_5UTR
rs10999907	0.142	10	73023575	0.277	4.80E-04	0.822	*CDH23*	intron
rs549467	0.069	4	99391255	0.276	4.81E-04	0.822	*RAP1GDS1*	flanking_5UTR
rs2476950	0.179	10	105741400	0.277	4.81E-04	0.822	*SLK*	intron
rs36631	0.356	19	59383226	0.276	4.82E-04	0.822	*LENG4*	intron
rs10159226	0.063	1	90783547	0.276	4.83E-04	0.822	*BARHL2*	flanking_3UTR
rs6889467	0.138	5	174509846	0.276	4.83E-04	0.822	*FLJ16171*	flanking_5UTR
rs11793341	0.095	9	82112200	-0.276	4.84E-04	0.822	*TLE4*	flanking_3UTR
rs5745309	0.06	1	76034502	0.276	4.84E-04	0.822	*MSH4*	flanking_5UTR
rs9970840	0.06	1	76076308	0.276	4.84E-04	0.822	*MSH4*	intron
rs2310047	0.336	4	185621464	0.276	4.84E-04	0.822	*ıtory factor 2 (IRF-2).*	intron
rs1338129	0.298	1	81150844	0.277	4.85E-04	0.822	*EGF*	upstream
rs10967476	0.164	9	26672007	0.276	4.85E-04	0.822	*C9orf82*	flanking_3UTR
rs17520924	0.113	1	184859754	-0.277	4.86E-04	0.822	*Prostaglandin G*	downstream
rs2726110	0.17	7	36181798	-0.276	4.86E-04	0.822	*CDNA FLJ14480 fis*	intron
rs1567374	0.155	5	42420633	-0.276	4.87E-04	0.822	*totropin receptor) [Cı*	upstream
rs1923891	0.089	13	98757946	-0.276	4.87E-04	0.822	*EBI2*	flanking_5UTR
rs13407683	0.228	2	34330942	0.279	4.87E-04	0.822	*MYADML*	flanking_5UTR
rs17010728	0.286	3	22091975	-0.277	4.88E-04	0.822	*ZNF659*	flanking_5UTR
rs16852873	0.124	3	107891561	-0.276	4.88E-04	0.822	---	downstream
rs7686911	0.454	4	189419557	-0.276	4.90E-04	0.822	---	upstream
rs11897323	0.086	2	105470842	-0.276	4.90E-04	0.822	*FHL2*	flanking_5UTR
rs7223160	0.142	17	11521980	0.277	4.91E-04	0.822	*DNAH9*	intron
rs2293936	0.101	8	23363198	0.276	4.92E-04	0.822	*ENTPD4*	intron
rs10136833	0.104	14	68470847	0.277	4.93E-04	0.822	*ACTN1*	intron
rs800464	0.167	4	24466007	0.276	4.94E-04	0.822	*DKFZp761B107*	intron
rs6907286	0.23	6	169894916	0.276	4.95E-04	0.822	*C6orf70*	intron
rs2972407	0.181	5	42491713	-0.276	4.96E-04	0.822	*GHR*	intron
rs2972411	0.181	5	42501873	-0.276	4.96E-04	0.822	*GHR*	intron
rs10899068	0.178	11	74463368	0.276	4.96E-04	0.822	*olfactory receptor*	downstream
rs4260395	0.189	3	55821509	0.280	4.96E-04	0.822	*ırotein 2. [Source:Un*	intron
rs429	0.212	7	19073030	-0.277	4.96E-04	0.822	*e 9 (HD9) (HD7B) (HD*	downstream
rs6027678	0.466	20	58546996	-0.276	4.98E-04	0.822	*LOC284757*	flanking_3UTR
rs4650355	0.451	1	81148870	0.276	4.99E-04	0.822	*LPHN2*	flanking_5UTR
rs12739375	0.451	1	81149896	0.276	4.99E-04	0.822	*LPHN2*	flanking_5UTR
rs1476984	0.078	2	102278701	-0.276	4.99E-04	0.822	*IL1RL1*	flanking_5UTR
rs9940044	0.207	16	64956434	-0.276	5.00E-04	0.822	*CDH5*	flanking_5UTR
rs9833533	0.06	3	60543293	0.275	5.00E-04	0.822	*FHIT*	intron
rs4979515	0.417	9	109674037	-0.275	5.00E-04	0.822	*KLF4*	flanking_5UTR
rs790006	0.178	12	62892706	-0.275	5.01E-04	0.822	*ıothetical protein FLJ*	intron

rs6031702	0.135	20	42718631	-0.275	5.01E-04	0.822	*ADA*	flanking_5UTR
rs11668840	0.414	19	17260625	-0.275	5.01E-04	0.822	*ANKRD41*	flanking_3UTR
rs4746864	0.28	10	71011206	-0.276	5.02E-04	0.822	*NEUROG3*	flanking_5UTR
rs838621	0.331	3	144670743	-0.277	5.02E-04	0.822	*SLC9A9*	intron
rs525214	0.351	11	88206742	-0.275	5.03E-04	0.822	*receptor 5 precursor (*	intron
rs1373201	0.399	15	77114223	-0.275	5.03E-04	0.822	*RASGRF1*	intron
rs4541202	0.434	2	214677246	-0.275	5.04E-04	0.822	*perm associated antig*	intron
rs10809954	0.08	9	13458616	-0.275	5.04E-04	0.822	*MPDZ*	flanking_5UTR
rs11802583	0.046	1	180504381	0.275	5.05E-04	0.822	*GLUL*	flanking_3UTR
rs2390538	0.203	7	21509416	0.279	5.05E-04	0.822	*SP4*	intron
rs292644	0.422	7	134598040	0.275	5.09E-04	0.827	*) (E3 ubiquitin protein*	downstream
rs10942294	0.408	5	71747025	-0.275	5.10E-04	0.827	*tatricopeptide repeat*	downstream
rs28445915	0.234	23	46428969	0.276	5.11E-04	0.827	*SLC9A7*	intron
rs856564	0.324	7	46689320	-0.276	5.11E-04	0.827	---	upstream
rs17708487	0.132	6	90700670	-0.275	5.11E-04	0.827	*BACH2*	intron
rs2810979	0.049	9	152695	0.275	5.13E-04	0.828	*-A-6 COBW domain-*	intron
rs6414829	0.141	5	54003879	-0.275	5.14E-04	0.828	*g nexin associated go*	downstream
rs2023169	0.394	6	102096952	0.275	5.14E-04	0.828	*GRIK2*	intron
rs13408950	0.21	2	14599626	0.275	5.14E-04	0.828	*FAM84A*	flanking_5UTR
rs7697678	0.228	4	101917055	0.276	5.16E-04	0.828	*PPP3CA*	flanking_3UTR
rs9809107	0.422	3	44257938	-0.275	5.18E-04	0.828	*C3orf23*	flanking_5UTR
rs12109575	0.198	5	119915738	0.279	5.18E-04	0.828	*n DSC54 [Source:RefS*	intron
rs6945128	0.364	7	89861015	-0.276	5.18E-04	0.828	*DKFZP686A10121*	flanking_3UTR
rs11962234	0.121	6	66046468	0.275	5.19E-04	0.828	*0016684 (Fragment).*	upstream
rs11749989	0.11	5	65913718	0.276	5.19E-04	0.828	*FLJ46010*	flanking_3UTR
rs7307968	0.394	12	81715468	0.275	5.19E-04	0.828	*epeat containing 2 [S*	intron
rs17653077	0.101	15	27314017	-0.275	5.21E-04	0.828	*NDNL2*	flanking_3UTR
rs10976143	0.075	9	7235081	-0.275	5.22E-04	0.828	*3C (EC 1.14.11.-) (Jun*	downstream
rs1524571	0.17	5	119934014	0.275	5.24E-04	0.828	*n DSC54 [Source:RefS*	intron
rs772492	0.172	4	90591589	-0.275	5.24E-04	0.828	*LOC285513*	flanking_5UTR
rs2419863	0.087	10	115544640	0.276	5.24E-04	0.828	*C10orf81*	flanking_3UTR
rs17098442	0.092	1	76472884	0.274	5.26E-04	0.828	*acetylgalactosaminid*	intron
rs17098450	0.092	1	76473285	0.274	5.26E-04	0.828	*ST6GALNAC3*	intron
rs9586929	0.057	13	105102225	0.274	5.26E-04	0.828	*DAOA*	flanking_3UTR
rs10840303	0.364	11	2049559	0.275	5.27E-04	0.828	*tomedin A) [Contains*	downstream
rs535481	0.293	15	44723855	0.274	5.28E-04	0.828	*SQRDL*	flanking_3UTR
rs6494238	0.221	15	59056595	-0.276	5.29E-04	0.828	*a (Nuclear receptor R*	intron
rs7842027	0.397	8	15672695	-0.274	5.29E-04	0.828	*TUSC3*	flanking_3UTR
rs17731038	0.457	17	25780275	0.274	5.29E-04	0.828	*CPD*	intron
rs4755978	0.184	11	45337126	0.274	5.30E-04	0.828	*naptotagmin XIII) (Sy*	upstream
rs4755319	0.184	11	45337922	0.274	5.30E-04	0.828	*FLJ41423*	flanking_5UTR
rs11090463	0.181	22	25707569	0.274	5.30E-04	0.828	*CDNA FLJ42830 fis*	upstream
rs9303624	0.305	17	24587116	-0.274	5.32E-04	0.828	*CRYBA1*	flanking_5UTR
rs2658658	0.315	12	51075195	-0.278	5.33E-04	0.828	*KRTHB2*	coding
rs2600056	0.466	3	2019132	-0.274	5.34E-04	0.828	---	upstream
rs6706755	0.136	2	136878533	0.275	5.34E-04	0.828	---	upstream
rs2925239	0.463	15	23332768	0.274	5.35E-04	0.828	*: 3.6.3.1) (ATPVA) (Ar*	downstream
rs10493682	0.299	1	81150159	0.274	5.36E-04	0.828	---	upstream
rs10493683	0.299	1	81151357	0.274	5.36E-04	0.828	---	upstream
rs10493684	0.299	1	81151517	0.274	5.36E-04	0.828	*EGF*	upstream
rs2152988	0.299	1	81151826	0.274	5.36E-04	0.828	*LPHN2*	flanking_5UTR
rs1415929	0.299	1	81152719	0.274	5.36E-04	0.828	*LPHN2*	flanking_5UTR
rs10821383	0.327	9	96485744	0.275	5.37E-04	0.828	*P00000021697. [Sour*	intron
rs10478479	0.161	5	119930107	0.274	5.39E-04	0.828	*apiens proline rich 16*	intron
rs485783	0.221	23	45760931	0.274	5.39E-04	0.828	*ZNF673*	flanking_5UTR
rs17733014	0.095	8	116065687	0.274	5.40E-04	0.828	*TRPS1*	flanking_3UTR
rs10736862	0.357	9	132273076	0.277	5.40E-04	0.828	*ASS*	flanking_5UTR
rs6897885	0.072	5	15704836	-0.274	5.41E-04	0.828	*FBXL7*	intron

rs2275540	0.221	10	128687707	0.274	5.41E-04	0.828	*DOCK1*	intron
rs2784624	0.124	9	10468387	0.274	5.42E-04	0.828	*PTPRD*	flanking_5UTR
rs547484	0.365	11	63993697	0.274	5.42E-04	0.828	*CDNA FLJ37045 fis*	downstream
rs11666947	0.059	19	20452848	-0.278	5.43E-04	0.828	*ZNF626*	flanking_3UTR
rs7057039	0.19	23	51379351	0.274	5.43E-04	0.828	*NUDT11*	flanking_5UTR
rs216478	0.445	17	25891161	-0.274	5.43E-04	0.828	*GOSR1*	flanking_3UTR
rs12442791	0.086	15	92998082	0.274	5.43E-04	0.828	*MCTP2*	flanking_3UTR
rs10486064	0.057	7	13954169	0.274	5.44E-04	0.828	*ETV1*	intron
rs10811063	0.305	9	18911386	0.274	5.45E-04	0.828	*orf94 precursor. [Sour*	downstream
rs918912	0.362	8	57934665	-0.274	5.45E-04	0.828	*t-enkephalin (Opioid*	upstream
rs11038408	0.213	11	45327064	0.274	5.47E-04	0.828	*FLJ41423*	flanking_5UTR
rs2238149	0.294	12	109796312	-0.275	5.47E-04	0.828	*CCDC63*	intron
rs28538558	0.08	23	51643274	0.274	5.47E-04	0.828	*MAGED1*	intron
rs11796891	0.08	23	51650703	0.274	5.47E-04	0.828	*MAGED1*	intron
rs2545902	0.228	19	20475270	-0.276	5.48E-04	0.828	*CDNA FLJ16190 fis*	downstream
rs13265751	0.244	8	19384299	0.274	5.49E-04	0.828	*ChGn*	intron
rs2177773	0.216	7	129404593	0.274	5.50E-04	0.829	*UBE2H*	flanking_5UTR
rs17612219	0.407	8	15518950	0.275	5.51E-04	0.830	*andidate 3 (Protein N*	intron
rs888932	0.451	19	4243597	0.273	5.52E-04	0.830	*MGC23244*	coding
rs12190178	0.135	6	10463709	0.273	5.52E-04	0.830	*TFAP2A*	flanking_3UTR
rs16959655	0.086	17	60514400	0.273	5.53E-04	0.830	*GNA13*	flanking_5UTR
rs12882499	0.402	14	85081484	0.274	5.54E-04	0.830	*FLRT2*	intron
rs10478477	0.181	5	119928918	0.273	5.54E-04	0.830	*LOC51334*	intron
rs2940917	0.182	5	42503212	-0.274	5.56E-04	0.833	*GHR*	intron
rs36167	0.477	3	136135447	0.274	5.60E-04	0.836	*EPHB1*	intron
rs11771918	0.149	7	64538847	-0.273	5.61E-04	0.836	*'Zinc finger protein H*	downstream
rs17783784	0.149	7	64539287	-0.273	5.61E-04	0.836	*'Zinc finger protein H*	downstream
rs1373202	0.425	15	77108843	-0.273	5.62E-04	0.836	*RASGRF1*	intron
rs12914601	0.08	15	96715548	-0.273	5.63E-04	0.836	*CDNA FLJ39743 fis*	downstream
rs1027348	0.11	5	41822023	0.274	5.63E-04	0.836	*OXCT1*	intron
rs16829380	0.06	3	179578889	0.273	5.64E-04	0.836	*al RNA [Source:RFAM*	upstream
rs4557646	0.092	7	48821333	0.273	5.64E-04	0.836	*ABCA13*	flanking_3UTR
rs1893864	0.353	11	127984614	0.273	5.65E-04	0.836	*protein (p54). [Source*	upstream
rs7292688	0.136	22	47406839	-0.274	5.66E-04	0.836	*e similarity 19 (chemc*	intron
rs216468	0.497	17	25886534	-0.278	5.66E-04	0.836	*lanking_3UTR*	NM_001007025.1
rs10756918	0.35	9	17833275	0.274	5.67E-04	0.836	*cursor (ADAMTSL-1) (*	upstream
rs2425417	0.431	20	38760773	-0.273	5.67E-04	0.836	*MAFB*	flanking_5UTR
rs7160530	0.184	14	85080558	0.273	5.68E-04	0.837	*rsor (Fibronectin-like*	intron
rs12328809	0.049	2	158117350	0.273	5.71E-04	0.838	*ACVR1C*	intron
rs2701422	0.069	15	85380830	0.273	5.72E-04	0.838	*TMEM83*	flanking_5UTR
rs11887003	0.08	2	83510331	0.273	5.72E-04	0.838	*P1 protein. [Source:U*	downstream
rs5750854	0.178	22	38320721	-0.273	5.75E-04	0.838	*CACNA1I*	intron
rs4616538	0.164	20	38052460	-0.272	5.79E-04	0.838	*MAFB*	flanking_3UTR
rs7254850	0.055	19	55183842	0.272	5.81E-04	0.838	*VRK3*	intron
rs10165172	0.078	2	47365582	0.272	5.81E-04	0.838	*dulin (CaM). [Source:l*	upstream
rs1420702	0.368	16	47391826	0.272	5.82E-04	0.838	*N4BP1*	flanking_5UTR
rs12273886	0.071	11	98730384	0.276	5.82E-04	0.838	*recognition molecule*	upstream
rs1876790	0.193	5	42458612	-0.272	5.82E-04	0.838	*GHR*	flanking_5UTR
rs9498309	0.069	6	149667233	0.272	5.82E-04	0.838	---	downstream
rs12392286	0.092	23	51570994	0.272	5.83E-04	0.838	*MAGED1*	intron
rs6805070	0.063	3	62160445	0.272	5.84E-04	0.838	*ursor (EC 3.1.3.48) (Pr*	intron
rs1430014	0.141	2	37914087	-0.272	5.85E-04	0.838	*FAM82A*	flanking_5UTR
rs9321801	0.279	6	141533118	0.272	5.85E-04	0.838	*NMBR*	flanking_3UTR
rs3017883	0.353	11	88915360	0.272	5.86E-04	0.838	*al RNA [Source:RFAM*	downstream
rs10962582	0.334	9	16762775	0.274	5.86E-04	0.838	*BNC2*	flanking_5UTR
rs12142755	0.268	1	159748367	-0.277	5.87E-04	0.838	*FCGR2A*	intron
rs10491540	0.104	9	17750097	0.273	5.87E-04	0.838	*SH3GL2*	intron
rs12878184	0.402	14	96345009	0.272	5.87E-04	0.838	*VRK1*	intron

rs126077	0.483	22	27832728	-0.272	5.89E-04	0.838	*KREMEN1*	intron
rs134678	0.483	22	27833955	-0.272	5.89E-04	0.838	*narking the eye and t*	intron
rs2324520	0.413	11	130980245	0.274	5.89E-04	0.838	*C11orf39*	flanking_3UTR
rs6003605	0.138	22	21973212	-0.272	5.90E-04	0.838	*n (EC 2.7.11.1) (NY-R*	intron
rs4703855	0.306	5	71729655	0.273	5.90E-04	0.838	*PTCD2*	flanking_3UTR
rs5945545	0.256	23	143516637	-0.272	5.90E-04	0.838	*[Source:RFAM;Acc:R*	upstream
rs9913400	0.279	17	59614854	-0.272	5.91E-04	0.838	*l sequence 2 protein.*	intron
rs10063554	0.182	5	119906981	0.273	5.91E-04	0.838	*LOC51334*	intron
rs10469243	0.092	18	57766814	0.272	5.92E-04	0.838	*er protein 152. [Sourc*	upstream
rs9646628	0.092	18	57775333	0.272	5.92E-04	0.838	*er protein 152. [Sourc*	upstream
rs17528635	0.066	14	26729496	0.272	5.92E-04	0.838	*' antigen 1) (Onconeu*	upstream
rs9536697	0.233	13	53847603	-0.272	5.93E-04	0.838	*LOC387930*	flanking_3UTR
rs17269854	0.322	9	82921870	0.272	5.93E-04	0.838	*TLE1*	flanking_3UTR
rs4930402	0.385	11	64026514	0.272	5.93E-04	0.838	*LOC439914*	flanking_3UTR
rs7785128	0.23	7	133335531	0.272	5.93E-04	0.838	*SEC8L1*	intron
rs12255651	0.109	10	53511111	0.272	5.94E-04	0.838	*PRKG1*	intron
rs9524193	0.172	13	93204846	0.272	5.95E-04	0.838	*-6 precursor. [Source*	intron
rs7847004	0.425	9	96506794	-0.272	5.95E-04	0.838	*P00000021697. [Sour*	intron
rs28362683	0.129	6	32480941	0.272	5.96E-04	0.838	*BTNL2*	coding
rs10473829	0.198	5	27337279	0.274	5.96E-04	0.838	*CDH9*	flanking_5UTR
rs11563279	0.414	7	89840352	-0.272	5.97E-04	0.838	*ource:RefSeq_peptid*	intron
rs20838	0.279	14	71714279	0.272	5.98E-04	0.838	*signaling 6 (RGS6) (S*	intron
rs2857901	0.218	11	6937087	-0.272	5.98E-04	0.838	*ZNF215*	flanking_3UTR
rs10903497	0.233	10	1614900	0.272	5.99E-04	0.838	*ADARB2*	intron
rs2278947	0.204	8	15667029	-0.272	5.99E-04	0.838	*andidate 3 (Protein N*	3UTR
rs2660283	0.483	18	10987117	0.272	6.02E-04	0.838	*C18orf58*	flanking_5UTR
rs7323428	0.361	13	35541601	-0.273	6.02E-04	0.838	*DCAMKL1*	intron
rs340315	0.187	3	160058283	-0.272	6.02E-04	0.838	*MFSD1*	flanking_3UTR
rs174812	0.17	3	76869567	-0.277	6.02E-04	0.838	*al RNA [Source:RFAM*	upstream
rs2178579	0.191	11	129136272	0.273	6.03E-04	0.838	*TMEM45B*	flanking_5UTR
rs7068714	0.193	10	87391377	-0.272	6.03E-04	0.838	*GRID1*	intron
rs9896542	0.282	17	59613108	-0.272	6.04E-04	0.838	*TEX2*	intron
rs2587493	0.408	17	75467390	0.272	6.05E-04	0.838	*(Polycomb 2 homolo*	upstream
rs2512885	0.408	11	130973004	0.272	6.05E-04	0.838	*precursor (hNT). [So*	intron
rs2512884	0.408	11	130973066	0.272	6.05E-04	0.838	*C11orf39*	flanking_3UTR
rs6029273	0.414	20	38747104	-0.272	6.07E-04	0.838	*MAFB*	flanking_3UTR
rs2060650	0.299	11	26824563	-0.271	6.10E-04	0.838	*e carrier family 5 (so*	upstream
rs12554486	0.417	9	96503256	-0.271	6.11E-04	0.838	*C9orf3*	flanking_5UTR
rs10995932	0.089	10	66045800	0.271	6.12E-04	0.838	---	downstream
rs2579382	0.089	2	197729334	0.271	6.13E-04	0.838	*ANKRD44*	intron
rs2324509	0.408	11	130974721	0.272	6.13E-04	0.838	*C11orf39*	flanking_3UTR
rs9937755	0.062	16	77065802	0.275	6.14E-04	0.838	*tase isoform 1 [Sourc*	intron
rs5754783	0.411	22	32709623	-0.271	6.14E-04	0.838	*LARGE*	flanking_5UTR
rs10068816	0.06	5	27897274	0.271	6.16E-04	0.838	*CDH9*	flanking_5UTR
rs1386175	0.092	4	103157017	-0.271	6.17E-04	0.838	*BANK1*	intron
rs11796743	0.087	23	51502935	0.272	6.18E-04	0.838	*GSPT2*	flanking_5UTR
rs4921651	0.231	8	19383365	0.272	6.18E-04	0.838	*ChGn*	intron
rs10859671	0.086	12	93021360	-0.271	6.18E-04	0.838	---	upstream
rs4871140	0.095	8	122017739	0.272	6.20E-04	0.838	*in A1 basic componer*	upstream
rs911901	0.471	20	55385594	-0.271	6.20E-04	0.838	*RAE1*	intron
rs1858233	0.491	1	160570623	-0.271	6.22E-04	0.838	*synthase protein (C--*	intron
rs2026804	0.276	9	16790341	0.271	6.25E-04	0.838	*BNC2*	flanking_5UTR
rs10962612	0.276	9	16794167	0.271	6.25E-04	0.838	*otein basonuclin-2. [S*	intron
rs2304773	0.124	2	233900559	0.271	6.26E-04	0.838	*rotein) (S-AG) (Rod p*	CDS
rs10772627	0.241	12	13226407	0.271	6.26E-04	0.838	*EMP1*	flanking_5UTR
rs906167	0.234	11	130990920	-0.273	6.26E-04	0.838	*C11orf39*	flanking_3UTR
rs4936285	0.182	11	113322789	-0.272	6.26E-04	0.838	*-3) (Serotonin-gated*	upstream
rs2359895	0.172	12	5369951	0.271	6.29E-04	0.838	*NTF3*	flanking_5UTR

rs7885885	0.132	23	111046846	-0.271	6.29E-04	0.838	*TRPC5*	intron
rs8100241	0.405	19	17253894	-0.272	6.30E-04	0.838	*ANKRD41*	coding
rs318156	0.293	23	96994406	0.271	6.30E-04	0.838	*NA ACA25 [Source:RF.*	upstream
rs7953496	0.399	12	81714677	0.272	6.31E-04	0.838	*epeat containing 2 [S*	intron
rs9385939	0.333	6	141548237	0.271	6.31E-04	0.838	*NMBR*	flanking_3UTR
rs6925531	0.333	6	141550230	0.271	6.31E-04	0.838	---	downstream
rs12769874	0.159	10	56001175	0.272	6.32E-04	0.838	*PCDH15*	intron
rs2276308	0.19	11	113309186	-0.271	6.32E-04	0.838	*otor 3B precursor [So*	intron
rs4905558	0.437	14	96415559	0.271	6.33E-04	0.838	*VRK1*	intron
rs17628328	0.144	22	43833503	0.271	6.33E-04	0.838	*PHF21B*	flanking_5UTR
rs17320646	0.092	23	109065205	0.271	6.33E-04	0.838	*RP13-360B22.2*	flanking_5UTR
rs2713568	0.09	11	25421296	0.271	6.33E-04	0.838	*[Source:RefSeq_pept*	downstream
rs9825996	0.153	3	134727309	-0.271	6.34E-04	0.838	*CDV3*	flanking_5UTR
rs4527434	0.29	4	177816205	-0.275	6.35E-04	0.838	*VEGFC*	flanking_3UTR
rs4078254	0.477	5	112483966	0.271	6.35E-04	0.838	*ncer protein (Protein I*	intron
rs10511639	0.066	9	17860918	0.271	6.36E-04	0.838	*SH3GL2*	flanking_3UTR
rs7595079	0.368	2	143667795	-0.274	6.36E-04	0.838	*ARHGAP15*	intron
rs635445	0.48	12	5186241	-0.271	6.36E-04	0.838	*KCNA5*	flanking_3UTR
rs34350121	0.09	5	128077355	-0.271	6.37E-04	0.838	*-2 precursor. [Source:*	upstream
rs8015021	0.102	14	101379446	0.272	6.38E-04	0.838	*Serine*	intron
rs5906254	0.333	23	46427722	0.270	6.38E-04	0.838	*SLC9A7*	intron
rs4815697	0.207	20	4312866	-0.270	6.38E-04	0.838	*ADRA1D*	flanking_5UTR
rs1572379	0.236	1	45141673	-0.270	6.38E-04	0.838	*a (eIF-2B GDP-GTP e.*	intron
rs2753147	0.147	6	124650320	-0.270	6.38E-04	0.838	*TCBA1*	intron
rs10504609	0.06	8	77208628	-0.270	6.39E-04	0.838	---	upstream
rs1247956	0.218	12	52250081	-0.270	6.40E-04	0.838	*ATF7*	intron
rs1247955	0.218	12	52250400	-0.270	6.40E-04	0.838	*ATF7*	intron
rs1153134	0.218	12	52277588	-0.270	6.40E-04	0.838	*ATF7*	intron
rs7172407	0.376	15	71637454	0.271	6.41E-04	0.838	*SDFR1*	flanking_3UTR
rs10747780	0.282	12	56266808	-0.270	6.41E-04	0.838	*KIF5A*	flanking_3UTR
rs6727797	0.09	2	65879935	0.271	6.42E-04	0.838	*FLJ16124*	intron
rs790007	0.191	12	62891060	-0.271	6.42E-04	0.838	*intron*	NM_15244
rs1319535	0.387	9	28171085	0.271	6.43E-04	0.838	*LRRN6C*	intron
rs993215	0.379	11	88180007	-0.271	6.44E-04	0.838	*GRM5*	intron
rs9575700	0.329	13	35735999	-0.271	6.45E-04	0.838	*SPG20*	flanking_3UTR
rs11129424	0.142	3	30823257	0.271	6.45E-04	0.838	*GADL1*	intron
rs10981291	0.109	9	113964541	-0.270	6.46E-04	0.838	*SUSD1*	intron
rs7741438	0.338	6	5668117	-0.271	6.47E-04	0.838	*FARS2*	intron
rs4715136	0.256	6	49623484	-0.270	6.47E-04	0.838	*C6orf141*	flanking_5UTR
rs10139234	0.075	14	70193313	0.270	6.47E-04	0.838	*MED6*	flanking_5UTR
rs1133851	0.152	18	3712038	0.270	6.48E-04	0.838	*DLGAP1*	intron
rs10900518	0.104	1	203874137	0.271	6.48E-04	0.838	*ELK4*	flanking_5UTR
rs1036585	0.135	3	167106528	-0.270	6.48E-04	0.838	*BCHE*	flanking_5UTR
rs6885758	0.27	5	117954515	-0.270	6.48E-04	0.838	---	upstream
rs12388178	0.061	23	145132127	0.271	6.49E-04	0.838	*in CXorf1. [Source:Ur*	downstream
rs10794323	0.084	11	418385	0.271	6.50E-04	0.838	*TMEM16J*	coding
rs12240927	0.204	10	132433479	-0.270	6.51E-04	0.838	*ator 1-like [Source:Re*	downstream
rs10076166	0.101	5	123919376	0.270	6.53E-04	0.838	*ZNF608*	flanking_3UTR
rs10512453	0.086	17	29090113	-0.270	6.53E-04	0.838	*ACCN1*	flanking_5UTR
rs2608157	0.178	3	76893305	-0.270	6.53E-04	0.838	*al RNA [Source:RFAM*	upstream
rs10274515	0.19	7	21127909	-0.270	6.55E-04	0.838	---	downstream
rs17679859	0.19	7	21128344	-0.270	6.55E-04	0.838	*LOC222901*	flanking_3UTR
rs4965480	0.503	15	97763404	-0.270	6.56E-04	0.838	*LRRC28*	flanking_3UTR
rs2189439	0.471	7	29413667	-0.270	6.56E-04	0.838	*CHN2*	intron
rs4910326	0.167	11	11342764	0.270	6.56E-04	0.838	*e-like protein 4 (EC 2.*	intron
rs3924355	0.385	23	6773396	0.270	6.57E-04	0.838	---	upstream
rs751348	0.207	5	168643201	0.270	6.59E-04	0.838	*SLIT3*	intron
rs6953359	0.063	7	113975410	-0.270	6.59E-04	0.838	*) (Trinucleotide repe*	intron

rs17764140	0.356	16	21157534	-0.270	6.60E-04	0.838	*ANKS4B*	intron
rs11754251	0.335	6	141536081	0.271	6.60E-04	0.838	*NMBR*	flanking_3UTR
rs8008342	0.17	14	71629644	0.270	6.60E-04	0.838	*RGS6*	intron
rs4974144	0.138	3	55781036	0.270	6.60E-04	0.838	*CAST1*	intron
rs5922854	0.296	23	82823722	-0.270	6.61E-04	0.838	*POU3F4*	flanking_3UTR
rs2142028	0.083	21	35997047	0.270	6.62E-04	0.838	---	downstream
rs1565214	0.429	3	44309303	-0.274	6.62E-04	0.838	*2 of Q8N3R3 [Source*	upstream
rs10158703	0.275	1	194454591	-0.271	6.62E-04	0.838	*potassium channel*	downstream
rs2193173	0.514	12	15199620	-0.270	6.64E-04	0.838	*n-regulated growth ir*	intron
rs9393607	0.256	6	25131232	0.271	6.65E-04	0.838	*LOC134997*	flanking_3UTR
rs10945886	0.106	6	163629800	0.270	6.65E-04	0.838	*PACRG*	intron
rs1327301	0.185	23	51226797	0.270	6.66E-04	0.838	*NUDT11*	flanking_3UTR
rs13015698	0.164	2	227409062	0.270	6.66E-04	0.838	*RHBDD1*	intron
rs7979812	0.155	12	55067711	0.270	6.67E-04	0.838	*APOF*	flanking_5UTR
rs6987564	0.208	8	3151550	0.272	6.68E-04	0.838	*ecursor (CUB and sush*	intron
rs2372533	0.138	14	83323662	0.270	6.68E-04	0.838	---	downstream
rs10484109	0.138	14	83335031	0.270	6.68E-04	0.838	*FLRT2*	flanking_5UTR
rs919139	0.477	1	81223342	-0.269	6.69E-04	0.838	*LPHN2*	flanking_5UTR
rs7989154	0.506	13	35565442	-0.269	6.70E-04	0.838	*DCAMKL1*	intron
rs1266212	0.118	14	58960710	0.274	6.70E-04	0.838	*ctivator of morphoge*	downstream
rs12378332	0.106	9	17786377	0.269	6.70E-04	0.838	*hilin-1) (Endophilin-A*	3UTR
rs7976912	0.101	12	20262861	0.269	6.71E-04	0.838	*PDE3A*	flanking_5UTR
rs2306372	0.269	3	151179048	0.270	6.71E-04	0.838	*PFN2*	flanking_5UTR
rs10101364	0.126	8	20679168	-0.269	6.72E-04	0.838	*LZTS1*	flanking_5UTR
rs6069883	0.448	20	54811858	-0.269	6.73E-04	0.838	*TFAP2C*	flanking_3UTR
rs4781002	0.218	16	10835613	-0.269	6.73E-04	0.838	*CIITA*	flanking_5UTR
rs744059	0.279	15	77146560	0.269	6.74E-04	0.838	*ific nucleotide exchai*	intron
rs17469572	0.213	4	15043076	-0.269	6.77E-04	0.838	*is factor-related prote*	intron
rs2314590	0.213	4	15054631	-0.269	6.77E-04	0.838	*C1QTNF7*	3UTR
rs7231168	0.231	18	2319839	0.270	6.77E-04	0.838	*4 [Source:RefSeq_pe*	downstream
rs12789398	0.152	11	20638148	0.269	6.78E-04	0.838	*SLC6A5*	flanking_3UTR
rs1632205	0.095	18	33644290	0.269	6.78E-04	0.838	*bruno-like 4*	upstream
rs909662	0.129	23	46386322	-0.269	6.78E-04	0.838	*SLC9A7*	intron
rs495706	0.322	1	158989880	0.269	6.78E-04	0.838	*SLAMF7*	3UTR
rs518721	0.322	1	158990141	0.269	6.78E-04	0.838	*SLAMF7*	3UTR
rs8090641	0.141	18	11061006	-0.269	6.79E-04	0.838	*CDNA FLJ34907 fis*	upstream
rs1390033	0.095	12	61889795	-0.269	6.80E-04	0.838	*urce:RefSeq_peptide;*	downstream
rs1827704	0.09	5	116997024	0.275	6.81E-04	0.838	*SEMA6A*	flanking_5UTR
rs7975862	0.27	12	52364694	-0.269	6.81E-04	0.838	*ctivator [Source:RefS*	downstream
rs17740072	0.098	3	122583104	-0.270	6.81E-04	0.838	*POLQ*	flanking_3UTR
rs6011696	0.032	20	61345547	0.270	6.82E-04	0.838	*C20orf58*	intron
rs3798174	0.07	6	160472793	0.272	6.83E-04	0.838	*SLC22A1*	intron
rs5991820	0.089	23	51530860	0.269	6.83E-04	0.838	*GSPT2*	flanking_3UTR
rs2185044	0.282	1	81151670	0.269	6.85E-04	0.838	---	upstream
rs1338127	0.282	1	81156736	0.269	6.85E-04	0.838	*LPHN2*	flanking_5UTR
rs2185518	0.316	14	25336279	-0.269	6.86E-04	0.838	*NOVA1*	flanking_3UTR
rs1003447	0.392	2	39881422	0.271	6.86E-04	0.838	*Sodium*	downstream
rs2224718	0.405	1	3565920	0.269	6.86E-04	0.838	*TP73*	intron
rs10754564	0.069	1	245729021	-0.269	6.87E-04	0.838	*OR2W5*	flanking_3UTR
rs10950564	0.253	7	15503978	-0.269	6.88E-04	0.838	*FLJ16237*	intron
rs4930030	0.364	11	2043796	0.270	6.88E-04	0.838	*C11orf43*	flanking_3UTR
rs17400882	0.109	1	117028855	-0.269	6.88E-04	0.838	*el protein. [Source:Un*	upstream
rs4965479	0.466	15	97756786	0.269	6.89E-04	0.838	*LRRC28*	flanking_3UTR
rs11796215	0.078	23	70678006	0.269	6.89E-04	0.838	*OGT*	intron
rs12137276	0.106	1	217597449	0.269	6.90E-04	0.838	*LYPLAL1*	flanking_3UTR
rs1929715	0.29	9	82910738	0.269	6.90E-04	0.838	*TLE1*	flanking_3UTR
rs17562209	0.101	2	19774768	0.270	6.91E-04	0.838	*skipped-related 1. [Sc*	upstream
rs1857413	0.279	1	214878947	-0.269	6.91E-04	0.838	*ESRRG*	intron

rs987456	0.359	7	147743920	0.269	6.92E-04	0.838	*CNTNAP2*	UTR
rs2638801	0.388	14	85214800	0.269	6.93E-04	0.838	*e) (Galactosylcerami*	downstream
rs9580300	0.072	13	21618398	0.269	6.93E-04	0.838	---	downstream
rs4968548	0.173	17	56714449	-0.271	6.93E-04	0.838	*BCAS3*	intron
rs805683	0.154	10	105774113	0.270	6.96E-04	0.840	*STE20-like serine*	intron
rs17068153	0.105	4	180923203	-0.270	6.97E-04	0.840	*RNA [Source:RFAM;A*	downstream
rs12548410	0.264	8	15202214	-0.269	6.99E-04	0.840	*TUSC3*	flanking_5UTR
rs2639427	0.446	6	63156050	0.274	6.99E-04	0.840	---	downstream
rs1630769	0.095	18	33647015	0.269	7.00E-04	0.840	---	downstream
rs4933541	0.17	10	91822046	-0.274	7.00E-04	0.840	*MPHOSPH1*	flanking_3UTR
rs6867229	0.066	5	43273401	0.269	7.00E-04	0.840	*MGC42105*	intron
rs6791919	0.115	3	23779748	0.269	7.01E-04	0.840	*iquitin-protein ligase*	upstream
rs16831235	0.109	2	135461599	-0.269	7.01E-04	0.840	*YSK4*	coding
rs12481078	0.057	20	40856794	0.268	7.04E-04	0.840	*e T precursor (EC 3.1.*	intron
rs6602330	0.231	10	5984239	-0.269	7.06E-04	0.840	*FBXO18*	intron
rs1930158	0.244	10	56007561	0.268	7.08E-04	0.840	*PCDH15*	intron
rs6964584	0.22	7	14138657	-0.269	7.09E-04	0.840	---	upstream
rs892035	0.241	19	19619190	-0.268	7.09E-04	0.840	*ATP13A1*	intron
rs1689021	0.238	4	181293620	-0.270	7.10E-04	0.840	*RNA [Source:RFAM;A*	downstream
rs6795614	0.057	3	188171302	-0.268	7.10E-04	0.840	*raminate-beta-galac*	intron
rs10955775	0.121	8	117488126	-0.268	7.10E-04	0.840	*EIF3S3*	flanking_3UTR
rs7132287	0.204	12	81721742	0.274	7.11E-04	0.840	*epeat containing 2 [S*	intron
rs17098034	0.057	1	76305389	0.268	7.12E-04	0.840	*CS box protein 17 (AS*	upstream
rs2065973	0.069	6	125140679	0.268	7.13E-04	0.840	*TCBA1*	intron
rs2429310	0.185	5	139488889	0.269	7.13E-04	0.840	*LOC492311*	flanking_3UTR
rs9376605	0.179	6	141675959	-0.269	7.13E-04	0.840	---	downstream
rs11767621	0.144	7	46763069	0.268	7.14E-04	0.840	*TNS3*	flanking_3UTR
rs12599856	0.422	16	18929949	-0.268	7.15E-04	0.840	*TMC7*	intron
rs1322938	0.376	13	52629758	0.268	7.15E-04	0.840	*piens olfactomedin 4*	downstream
rs5967919	0.089	23	87286806	-0.268	7.15E-04	0.840	*PX chromosome regic*	upstream
rs1146042	0.095	7	40090662	-0.269	7.16E-04	0.840	*CDC2L5*	intron
rs4542417	0.382	11	88170839	-0.268	7.18E-04	0.840	*receptor 5 precursor (*	intron
rs12212158	0.087	6	51973335	-0.269	7.18E-04	0.840	*PKHD1*	intron
rs13184263	0.236	5	165447333	0.268	7.19E-04	0.840	*WWC1*	flanking_5UTR
rs6823540	0.37	4	155417341	-0.269	7.19E-04	0.840	*DCHS2*	intron
rs2638100	0.217	11	6926252	-0.269	7.19E-04	0.840	*ssociated zinc-finger*	intron
rs12787100	0.145	11	11342916	0.269	7.20E-04	0.840	*GALNTL4*	flanking_3UTR
rs7009035	0.451	8	15506367	-0.269	7.20E-04	0.840	*TUSC3*	intron
rs1010693	0.253	19	51205023	0.268	7.20E-04	0.840	*PGLYRP1*	flanking_3UTR
rs742487	0.172	6	2392698	0.268	7.21E-04	0.840	*C6orf195*	flanking_3UTR
rs1126140	0.221	23	82778245	-0.268	7.21E-04	0.840	*CDNA FLJ45563 fis*	upstream
rs264233	0.471	18	10964487	0.268	7.22E-04	0.840	---	upstream
rs10791130	0.422	11	130590320	-0.268	7.22E-04	0.840	---	upstream
rs1078211	0.233	6	169943225	0.268	7.23E-04	0.840	*C6orf208*	coding
rs10962599	0.275	9	16785286	0.269	7.24E-04	0.840	*BNC2*	flanking_5UTR
rs1408077	0.194	1	205870764	0.269	7.24E-04	0.840	*CR1*	intron
rs8016760	0.089	14	26729904	0.268	7.24E-04	0.840	---	upstream
rs4261154	0.474	1	31694449	-0.268	7.25E-04	0.840	*SERINC2*	flanking_3UTR
rs10009448	0.146	4	23184981	0.273	7.26E-04	0.840	*PPARGC1A*	flanking_3UTR
rs4403484	0.353	9	112318935	0.268	7.26E-04	0.840	*MUSK*	flanking_5UTR
rs2582354	0.33	3	144654575	-0.268	7.27E-04	0.840	*Sodium*	intron
rs1723034	0.33	3	144659938	-0.268	7.27E-04	0.840	*SLC9A9*	intron
rs838610	0.33	3	144661216	-0.268	7.27E-04	0.840	*SLC9A9*	intron
rs4941694	0.063	13	50688689	0.268	7.29E-04	0.840	*FLJ30707*	flanking_5UTR
rs13009904	0.12	2	179940373	-0.270	7.29E-04	0.840	*ins 1 [Source:RefSeq_*	upstream
rs12955215	0.341	18	32337237	-0.269	7.29E-04	0.840	*FHOD3*	intron
rs17469634	0.198	4	15051078	-0.268	7.30E-04	0.840	*C1QTNF7*	intron
rs17318744	0.149	1	208311093	-0.270	7.30E-04	0.840	*naptotagmin XIV) (Sy*	intron

rs10774156	0.302	12	3534965	0.268	7.31E-04	0.840	*PRMT8*	intron
rs1187229	0.095	18	33609010	0.268	7.31E-04	0.840	---	downstream
rs1419039	0.187	23	51364916	0.268	7.31E-04	0.840	---	upstream
rs13070820	0.142	3	143827	0.268	7.32E-04	0.840	*: protein precursor (C*	upstream
rs1402912	0.247	5	62211571	-0.268	7.33E-04	0.840	*IPO11*	flanking_3UTR
rs603119	0.224	18	56407631	0.267	7.36E-04	0.840	*MC4R*	flanking_5UTR
rs475328	0.098	13	100753752	0.267	7.36E-04	0.840	*:e 1 [Source:RefSeq_p*	intron
rs7213040	0.46	17	67445906	-0.267	7.36E-04	0.840	*SOX9*	flanking_5UTR
rs941650	0.115	14	91978826	-0.267	7.36E-04	0.840	*SLC24A4*	coding
rs12818390	0.466	12	31723811	-0.267	7.37E-04	0.840	*LOC196394*	intron
rs9323895	0.238	14	93409883	-0.269	7.38E-04	0.840	*anchor 1 precursor (F*	upstream
rs6979107	0.145	7	64508216	-0.269	7.39E-04	0.840	*ZNF92*	flanking_3UTR
rs406813	0.243	5	71873362	0.268	7.39E-04	0.840	*Source:RFAM;Acc:RF*	upstream
rs4668845	0.312	2	15079769	0.268	7.40E-04	0.840	*NAG*	flanking_3UTR
rs5915677	0.299	23	6314884	0.267	7.40E-04	0.840	*Neuroligin-4*	upstream
rs4826726	0.299	23	6317647	0.267	7.40E-04	0.840	*VCX3A*	flanking_3UTR
rs2477357	0.276	1	194518145	-0.269	7.40E-04	0.840	*KCNT2*	intron
rs11103778	0.121	9	137291493	-0.267	7.40E-04	0.840	*OLFM1*	flanking_3UTR
rs1251465	0.207	1	76310984	-0.267	7.41E-04	0.841	*ST6GALNAC3*	flanking_5UTR
rs13108980	0.451	4	105280755	0.267	7.44E-04	0.841	*CXXC4*	flanking_3UTR
rs7682855	0.046	4	111201595	0.268	7.45E-04	0.841	*ELOVL6*	intron
rs5905476	0.201	23	45435256	0.267	7.45E-04	0.841	*CXorf36*	flanking_5UTR
rs1948926	0.213	3	62653111	-0.267	7.47E-04	0.841	*CADPS*	intron
rs415467	0.353	5	3502329	0.267	7.47E-04	0.841	*is homeobox protein*	upstream
rs10755268	0.149	5	42420104	-0.267	7.49E-04	0.841	*GHR*	flanking_5UTR
rs4129822	0.121	3	55814450	0.267	7.49E-04	0.841	*CAST1*	intron
rs1458396	0.462	2	27989855	-0.268	7.52E-04	0.841	*BRE*	intron
rs1504584	0.101	1	57462924	0.267	7.52E-04	0.841	*d homolog 1. [Source.*	intron
rs1604885	0.101	1	57463256	0.267	7.52E-04	0.841	*d homolog 1. [Source.*	intron
rs9784816	0.075	6	19301959	0.267	7.52E-04	0.841	*ID4*	flanking_5UTR
rs2669681	0.124	10	47563239	0.267	7.52E-04	0.841	*ANXA8*	flanking_3UTR
rs1363530	0.129	5	147324564	-0.267	7.52E-04	0.841	*pho-epithelial Kazal-*	upstream
rs1345689	0.129	5	147325383	-0.267	7.52E-04	0.841	*MGC23985*	flanking_5UTR
rs1926318	0.371	13	35542142	-0.267	7.53E-04	0.841	*Serine*	intron
rs636027	0.356	17	34617319	0.267	7.54E-04	0.841	*RPL19*	flanking_3UTR
rs3782751	0.295	12	3543760	0.268	7.54E-04	0.841	*PRMT8*	intron
rs17010000	0.411	3	72730712	-0.267	7.54E-04	0.841	*al RNA [Source:RFAM*	downstream
rs1335308	0.276	6	141687429	0.267	7.55E-04	0.841	*NMBR*	flanking_3UTR
rs10168790	0.445	2	231201209	-0.267	7.57E-04	0.841	*CAB39*	flanking_5UTR
rs216483	0.486	17	25894853	-0.267	7.59E-04	0.841	*FZP434O047 protein*	upstream
rs904160	0.314	2	47331088	-0.271	7.60E-04	0.841	*CALM2*	flanking_5UTR
rs11236323	0.184	11	74476576	0.267	7.60E-04	0.841	*OR2AT4*	flanking_3UTR
rs7122948	0.184	11	74478477	0.267	7.60E-04	0.841	*OR2AT4*	flanking_5UTR
rs406952	0.276	2	76160853	-0.267	7.61E-04	0.841	*C2orf3*	flanking_5UTR
rs443713	0.276	2	76163892	-0.267	7.61E-04	0.841	*C2orf3*	flanking_5UTR
rs17880007	0.414	9	109672725	-0.267	7.61E-04	0.841	---	upstream
rs6586471	0.355	1	104648021	-0.268	7.61E-04	0.841	*AMY1C*	flanking_3UTR
rs10994198	0.325	10	61526041	0.267	7.61E-04	0.841	*ANK3*	intron
rs397868	0.244	5	71872752	0.267	7.62E-04	0.841	*:r protein 366. [Sourc*	upstream
rs381734	0.244	5	71879511	0.267	7.62E-04	0.841	*ZNF366*	flanking_5UTR
rs2889276	0.394	2	104080563	-0.267	7.63E-04	0.841	*POU3F3*	flanking_5UTR
rs10867457	0.098	9	81856169	0.268	7.63E-04	0.841	*enhancer protein 4. [*	downstream
rs705349	0.276	7	90511244	-0.267	7.65E-04	0.841	*Serine*	intron
rs16997636	0.139	23	127216522	0.267	7.66E-04	0.841	*ACTRT1*	flanking_5UTR
rs9951261	0.193	18	32400382	0.267	7.67E-04	0.841	*FHOD3*	intron
rs856589	0.341	7	46700832	-0.267	7.67E-04	0.841	*TNS3*	flanking_3UTR
rs1565215	0.422	3	44309103	-0.267	7.67E-04	0.841	*2 of Q8N3R3 [Source*	upstream
rs7561932	0.44	2	65398572	0.267	7.71E-04	0.841	*SPRED2*	intron

rs6529987	0.382	23	6785065	0.266	7.74E-04	0.841	riably charged protei	upstream
rs4742899	0.075	9	105862789	0.266	7.74E-04	0.841	SMC2L1	flanking_5UTR
rs7358999	0.075	13	90651437	0.266	7.74E-04	0.841	---	upstream
rs11217902	0.179	11	98355200	-0.267	7.74E-04	0.841	---	downstream
rs913581	0.155	9	7140997	0.266	7.74E-04	0.841	JMJD2C	intron
rs11071668	0.25	15	60445840	0.268	7.75E-04	0.841	ource:miRBase 8.2;Ac	upstream
rs6711215	0.339	2	76238995	-0.266	7.76E-04	0.841	S ribosomal protein L	downstream
rs939008	0.078	2	105499920	-0.267	7.77E-04	0.841	FHL2	flanking_5UTR
rs10157380	0.277	1	194454332	-0.267	7.77E-04	0.841	KCNT2	flanking_3UTR
rs240664	0.092	8	15704610	-0.266	7.79E-04	0.841	TUSC3	flanking_3UTR
rs1799941	0.106	17	7474148	0.266	7.80E-04	0.841	SHBG	flanking_5UTR
rs6859940	0.148	5	147356971	-0.271	7.80E-04	0.841	pho-epithelial Kazal-t	upstream
rs2001462	0.075	8	11332220	0.266	7.81E-04	0.841	13 protein. [Source:U	intron
rs4977415	0.465	9	20276082	0.270	7.81E-04	0.841	MLLT3	flanking_3UTR
rs11051544	0.494	12	31758100	0.269	7.83E-04	0.841	LOC196394	intron
rs9851265	0.275	3	151150116	0.267	7.84E-04	0.841	RNF13	intron
rs11102365	0.199	1	112309429	0.267	7.84E-04	0.841	ber 3 (Voltage-gated	intron
rs6574046	0.171	14	71627332	0.267	7.84E-04	0.841	ı signaling 6 (RGS6) (S	intron
rs17362909	0.069	2	179608918	-0.266	7.85E-04	0.841	ins 1 [Source:RefSeq_	downstream
rs35297839	0.269	12	8077120	-0.267	7.85E-04	0.841	FOXJ2	5UTR
rs976328	0.486	7	80711534	-0.266	7.86E-04	0.841	SEMA3C	flanking_5UTR
rs4765663	0.164	12	2049021	0.266	7.89E-04	0.841	-1C (Voltage- gated c	upstream
rs8071110	0.367	17	25697011	-0.267	7.90E-04	0.841	TMIGD	flanking_5UTR
rs10495843	0.175	2	35712846	0.266	7.90E-04	0.841	CRIM1	flanking_5UTR
rs10259370	0.055	7	125029108	0.266	7.92E-04	0.841	ource:miRBase 8.2;Ac	downstream
rs1167632	0.48	12	25965725	-0.268	7.93E-04	0.841	RASSF8	flanking_5UTR
rs6663357	0.261	1	97817527	0.266	7.94E-04	0.841	DPYD	intron
rs7681177	0.44	4	189890618	0.266	7.95E-04	0.841	---	upstream
rs1220078	0.132	9	25784090	0.266	7.95E-04	0.841	TUSC1	flanking_5UTR
rs10886586	0.221	10	121809910	0.266	7.95E-04	0.841	SEC23IP	flanking_3UTR
rs4751762	0.221	10	121812568	0.266	7.95E-04	0.841	SEC23IP	flanking_3UTR
rs1864432	0.276	2	37918175	0.266	7.97E-04	0.841	FAM82A	flanking_5UTR
rs4930034	0.353	11	2053283	0.266	7.98E-04	0.841	C11orf43	flanking_3UTR
rs420024	0.241	5	71880674	0.267	7.98E-04	0.841	r protein 366. [Sourc	upstream
rs17157706	0.125	7	8969113	-0.267	8.00E-04	0.841	ilin-1 precursor. [Sou	downstream
rs1577207	0.273	10	121817825	0.266	8.00E-04	0.841	ting protein (p125). [S	downstream
rs12247775	0.095	10	52691629	0.267	8.01E-04	0.841	-dependent protein ki	intron
rs328847	0.325	9	7674244	0.266	8.01E-04	0.841	C9orf123	flanking_3UTR
rs2119457	0.328	11	76300280	-0.266	8.01E-04	0.841	aline ceramidase) (Al	intron
rs1859297	0.136	21	41638418	-0.267	8.01E-04	0.841	FAM3B	intron
rs17090991	0.092	10	115543082	0.266	8.03E-04	0.841	otein (Fragment). [So	downstream
rs16854016	0.086	2	168216641	0.266	8.03E-04	0.841	B3GALT1	flanking_5UTR
rs9551000	0.327	13	18441915	-0.266	8.05E-04	0.841	0018069 (Fragment).	downstream
rs264202	0.483	18	10956525	0.266	8.05E-04	0.841	C18orf58	flanking_5UTR
rs11874989	0.195	18	42201214	0.266	8.06E-04	0.841	RNF165	intron
rs16854819	0.37	3	145218816	0.266	8.07E-04	0.841	C3orf58	flanking_3UTR
rs3120282	0.124	1	39544767	0.266	8.07E-04	0.841	MACF1	intron
rs9952630	0.386	18	56439430	0.272	8.07E-04	0.841	receptor 4 (MC4-R). [S	upstream
rs10217843	0.037	23	12514359	0.266	8.08E-04	0.841	FRMPD4	intron
rs216451	0.422	17	25904061	-0.266	8.08E-04	0.841	DKFZP434O047	flanking_5UTR
rs708435	0.371	10	36252152	0.266	8.08E-04	0.841	---	downstream
rs12415669	0.063	10	20964946	-0.266	8.08E-04	0.841	NEBL	flanking_3UTR
rs6891846	0.147	5	92746229	0.265	8.10E-04	0.841	NR2F1	flanking_5UTR
rs10139629	0.279	14	94105030	-0.265	8.11E-04	0.841	allikrein inhibitor) (Pro	intron
rs9540180	0.239	13	64126630	0.265	8.12E-04	0.841	0018488 (Fragment).	downstream
rs9598823	0.239	13	64126855	0.265	8.12E-04	0.841	0018488 (Fragment).	downstream
rs2324731	0.239	13	64128636	0.265	8.12E-04	0.841	OR7E156P	flanking_3UTR
rs4666014	0.463	2	27872679	-0.265	8.12E-04	0.841	RBKS	intron

rs545856	0.098	13	100754267	0.266	8.12E-04	0.841	*VGCNL1*	intron
rs7992041	0.24	13	18469844	-0.266	8.13E-04	0.841	---	upstream
rs4785157	0.256	16	47580561	-0.265	8.14E-04	0.841	*CBLN1*	flanking_3UTR
rs2276307	0.187	11	113309097	-0.265	8.15E-04	0.841	*HTR3B*	intron
rs4662945	0.44	2	129932513	-0.265	8.16E-04	0.841	*LOC151121*	flanking_5UTR
rs7603887	0.44	2	129932543	-0.265	8.16E-04	0.841	---	downstream
rs423333	0.225	5	73254710	-0.266	8.16E-04	0.841	*UTP15*	flanking_3UTR
rs8180101	0.08	3	83630125	0.265	8.16E-04	0.841	*GBE1*	flanking_5UTR
rs616879	0.46	6	10500486	0.265	8.16E-04	0.841	*TFAP2A*	flanking_3UTR
rs533558	0.46	6	10503558	0.265	8.16E-04	0.841	*TFAP2A*	flanking_3UTR
rs7195999	0.307	16	64359576	-0.265	8.19E-04	0.841	*CDH5*	flanking_5UTR
rs11140862	0.057	9	71715032	0.265	8.19E-04	0.841	*?00000021439. [Sour*	downstream
rs4091742	0.225	13	18488984	-0.266	8.20E-04	0.841		upstream
rs9897139	0.46	17	67441150	-0.265	8.20E-04	0.841	---	upstream
rs264246	0.486	18	10959377	0.265	8.22E-04	0.841	*C18orf58*	flanking_5UTR
rs8086975	0.233	18	2322259	0.265	8.23E-04	0.841	*METTL4*	flanking_3UTR
rs3819248	0.172	11	122182257	0.265	8.25E-04	0.841	*STS-1*	intron
rs10874118	0.486	1	80411535	0.265	8.25E-04	0.841	*EGF*	upstream
rs9446089	0.465	6	69824308	0.268	8.25E-04	0.841	*BAI3*	intron
rs6671991	0.141	1	217624434	0.265	8.25E-04	0.841	*LYPLAL1*	flanking_3UTR
rs16946750	0.259	16	47589442	-0.265	8.25E-04	0.841	*ns cerebellin 1 precur*	downstream
rs7146639	0.221	14	85227860	0.267	8.25E-04	0.841	*e) (Galactosylcerami*	downstream
rs10843294	0.057	12	29059445	0.265	8.26E-04	0.841	*'GGA-binding partner*	downstream
rs17323663	0.08	15	67887243	0.265	8.27E-04	0.841	*lar RNA [Source:RFAM*	upstream
rs4507794	0.127	8	1619932	-0.266	8.28E-04	0.841	*DLGAP2*	intron
rs6070536	0.384	20	56626208	-0.266	8.28E-04	0.841	*MGC4294*	flanking_5UTR
rs155320	0.436	18	10949495	0.268	8.28E-04	0.841	*C18orf58*	flanking_5UTR
rs10110974	0.41	8	130078969	-0.266	8.29E-04	0.841	*CCDC26*	flanking_3UTR
rs34725238	0.118	12	16469864	0.265	8.30E-04	0.841	*transcription factor L*	downstream
rs6452435	0.388	5	81238737	0.265	8.31E-04	0.841	*ce-specific single- str*	upstream
rs1001797	0.388	5	81239659	0.265	8.31E-04	0.841	*ATG10*	flanking_5UTR
rs817088	0.157	2	49429333	-0.267	8.33E-04	0.841	---	downstream
rs17116065	0.078	5	153754364	-0.265	8.34E-04	0.841	*GALNT10*	intron
rs6778630	0.284	3	154871464	0.265	8.34E-04	0.841	---	downstream
rs36161	0.476	3	136138979	0.269	8.34E-04	0.841	*EPHB1*	intron
rs9495937	0.511	6	141623875	0.265	8.35E-04	0.841	*NMBR*	flanking_3UTR
rs9586032	0.233	13	102460525	0.265	8.35E-04	0.841	*SLC10A2*	flanking_3UTR
rs11634079	0.06	15	27266330	-0.265	8.38E-04	0.841	*APBA2*	flanking_3UTR
rs9836484	0.369	3	78127379	0.266	8.39E-04	0.841	*ROBO2*	flanking_3UTR
rs10921206	0.129	1	190834297	0.265	8.39E-04	0.841	*RGS1*	flanking_3UTR
rs10403928	0.055	19	36762404	0.265	8.40E-04	0.841	*ZNF537*	flanking_5UTR
rs3778738	0.037	7	154287899	-0.265	8.42E-04	0.841	*DPP6*	intron
rs17531080	0.069	10	13488285	0.265	8.42E-04	0.841	*C10orf30*	flanking_3UTR
rs17432977	0.095	7	18633122	-0.265	8.43E-04	0.841	*HDAC9*	intron
rs4444327	0.413	16	18924467	-0.265	8.43E-04	0.841	*TMC7*	intron
rs10423090	0.253	19	35843574	0.266	8.43E-04	0.841	*ZNF536*	flanking_3UTR
rs1509913	0.491	7	80712158	-0.267	8.43E-04	0.841	---	downstream
rs8107226	0.164	19	4238377	0.265	8.44E-04	0.841	*SHD*	intron
rs11612727	0.28	12	109798672	-0.265	8.44E-04	0.841	*CCDC63*	intron
rs10760365	0.491	9	126270712	-0.270	8.44E-04	0.841	*GPR144*	coding
rs12260523	0.294	10	12712890	0.266	8.44E-04	0.841	*CAMK1D*	intron
rs784568	0.221	12	52213821	-0.265	8.45E-04	0.841	*ATF7*	intron
rs2749072	0.391	6	102108641	0.265	8.46E-04	0.841	*Glutamate receptor*	intron
rs998374	0.055	12	3305791	0.266	8.46E-04	0.841	*TSPAN9*	flanking_3UTR
rs9429874	0.106	1	209834361	0.265	8.46E-04	0.841	*SLC30A1*	flanking_5UTR
rs13374091	0.066	1	233649547	0.265	8.46E-04	0.841	*TBCE*	intron
rs16832594	0.066	1	233650212	0.265	8.46E-04	0.841	*TBCE*	intron
rs16832599	0.066	1	233652954	0.265	8.46E-04	0.841	*: E (Tubulin-folding co*	intron

rs6586877	0.394	8	16669543	-0.265	8.47E-04	0.841	*age acetylated LDL r*	upstream
rs7048548	0.195	9	38630619	0.265	8.47E-04	0.841	---	upstream
rs7970381	0.337	12	39505491	0.266	8.48E-04	0.841	*al RNA [Source:RFAM*	upstream
rs7328247	0.27	13	108825024	-0.264	8.48E-04	0.841	*r 8 [Source:RefSeq_pe*	downstream
rs13433251	0.142	20	1947855	-0.269	8.48E-04	0.841	*) (Preprodynorphin) [*	upstream
rs6445754	0.376	3	55782295	0.265	8.49E-04	0.841	*protein 2. [Source:Un*	intron
rs12332782	0.055	5	42155747	0.264	8.49E-04	0.841	*Source:RFAM;Acc:RF*	upstream
rs10780297	0.471	9	71125397	-0.264	8.49E-04	0.841	*C9orf61*	flanking_5UTR
rs2917539	0.361	11	88892901	0.265	8.49E-04	0.841	*ducing NADPH oxidas*	upstream
rs6727037	0.284	2	153000420	0.264	8.50E-04	0.841	*FMNL2*	intron
rs12780246	0.25	10	5938211	-0.264	8.50E-04	0.841	*Guanosine diphospho*	upstream
rs10928525	0.116	2	135452608	-0.266	8.50E-04	0.841	*SPS1*	intron
rs5965707	0.063	23	145131604	0.264	8.51E-04	0.841	*in CXorf1. [Source:Ur*	downstream
rs9929156	0.259	16	6589736	0.264	8.51E-04	0.841	*A2BP1*	flanking_5UTR
rs411238	0.216	1	30559814	0.264	8.52E-04	0.841	*MATN1*	flanking_3UTR
rs2895550	0.371	10	13225732	-0.264	8.52E-04	0.841	---	upstream
rs11089274	0.049	22	17770804	0.264	8.53E-04	0.841	*HIRA*	intron
rs9618567	0.049	22	17793706	0.264	8.53E-04	0.841	*HIRA*	intron
rs9350103	0.069	6	19258473	0.264	8.53E-04	0.841	*RNA [Source:RFAM;A*	upstream
rs17331811	0.055	8	77224205	-0.264	8.53E-04	0.841	*al RNA [Source:RFAM*	downstream
rs3802957	0.115	11	59993986	0.264	8.55E-04	0.841	*MS4A1*	3UTR
rs7244357	0.072	18	10992565	0.265	8.55E-04	0.841	*CDNA FLJ34907 fis*	upstream
rs4515584	0.158	8	117499026	-0.264	8.56E-04	0.841	*EIF3S3*	flanking_3UTR
rs6989815	0.158	8	117499872	-0.264	8.56E-04	0.841	*unit 3 (eIF-3 gamma,*	downstream
rs10429325	0.158	8	117509360	-0.264	8.56E-04	0.841	*EIF3S3*	flanking_3UTR
rs7007515	0.158	8	117513375	-0.264	8.56E-04	0.841	*unit 3 (eIF-3 gamma,*	downstream
rs7808695	0.138	7	25292075	0.264	8.57E-04	0.841	*C7orf9*	flanking_5UTR
rs17328874	0.161	2	35716897	0.264	8.59E-04	0.843	*CRIM-1) (Cysteine-rich*	upstream
rs12773880	0.118	10	87485514	0.265	8.61E-04	0.843	*GRID1*	intron
rs169476	0.147	21	23936004	0.265	8.62E-04	0.843	*C21orf74*	flanking_3UTR
rs2103249	0.057	10	37170764	-0.264	8.62E-04	0.843	*ANKRD30A*	flanking_5UTR
rs6114326	0.043	20	23763299	0.264	8.63E-04	0.843	*CST2*	flanking_5UTR
rs13113346	0.225	4	101833060	0.265	8.63E-04	0.843	---	upstream
rs2073755	0.103	22	17590740	-0.264	8.64E-04	0.843	*CLTCL1*	intron
rs2301475	0.443	1	20371755	-0.264	8.65E-04	0.843	*UBXD3*	flanking_5UTR
rs11624401	0.315	14	68229501	0.265	8.66E-04	0.843	---	upstream
rs7982293	0.172	13	21615681	-0.264	8.68E-04	0.843	*FGF9*	flanking_3UTR
rs1393299	0.417	1	240396414	-0.264	8.69E-04	0.843	*PLD5*	intron
rs10892448	0.348	11	119130033	0.266	8.70E-04	0.843	*xia-telangiectasia gro*	downstream
rs10803967	0.374	2	184248347	0.266	8.70E-04	0.843	*NUP35*	flanking_3UTR
rs868688	0.417	1	3290667	-0.264	8.70E-04	0.843	*PRDM16*	intron
rs805647	0.182	10	105760999	0.265	8.72E-04	0.843	*STE20-like serine*	intron
rs11901133	0.468	2	27923217	-0.264	8.73E-04	0.843	*RBKS*	intron
rs2782205	0.144	6	20045002	-0.264	8.73E-04	0.843	*ID4*	flanking_3UTR
rs511993	0.431	18	8086100	-0.264	8.74E-04	0.843	*PTPRM*	intron
rs2922874	0.158	8	6385578	-0.264	8.76E-04	0.843	*precursor (ANG-2). [*	intron
rs1544935	0.141	6	39232426	-0.264	8.76E-04	0.843	*KCNK5*	flanking_3UTR
rs1554006	0.359	2	111320584	-0.264	8.76E-04	0.843	*ACOXL*	intron
rs11140842	0.09	9	71708992	0.265	8.77E-04	0.843	*P00000021439. [Sourc*	intron
rs10863456	0.142	1	217629306	0.265	8.78E-04	0.843	*like protein 1 (EC 3.1.*	downstream
rs3129888	0.133	6	32519704	0.271	8.78E-04	0.843	*HLA-DRA*	intron
rs2191351	0.296	17	25783161	-0.264	8.80E-04	0.843	*CPD*	intron
rs1976938	0.145	12	51948192	-0.265	8.81E-04	0.843	*ESPL1*	flanking_5UTR
rs4085528	0.181	2	222480155	-0.264	8.81E-04	0.843	*PAX3*	flanking_3UTR
rs1609380	0.22	4	171577472	0.264	8.82E-04	0.843	*AADAT*	flanking_5UTR
rs2027873	0.401	6	106166337	0.265	8.83E-04	0.843	*6) (Post-proline cleav*	upstream
rs13432312	0.066	2	38343828	0.264	8.83E-04	0.843	*ARL6IP2*	flanking_3UTR
rs11739066	0.178	5	4310463	-0.264	8.85E-04	0.843	*IRX1*	flanking_3UTR

rs6604882	0.46	1	222307562	-0.264	8.86E-04	0.843	*FBXO28*	flanking_5UTR
rs4877536	0.486	9	81922290	0.264	8.86E-04	0.843	*TLE4*	flanking_3UTR
rs972984	0.141	14	63091961	0.264	8.86E-04	0.843	*PPP2R5E*	flanking_5UTR
rs967285	0.425	3	44250280	-0.264	8.87E-04	0.843	*CDNA FLJ36157 fis*	upstream
rs3172941	0.451	5	153778073	-0.264	8.87E-04	0.843	*GALNT10*	3UTR
rs6054815	0.233	20	7147666	-0.264	8.87E-04	0.843	*n 2 precursor (BMP-2*	downstream
rs2638087	0.183	11	6947092	-0.268	8.89E-04	0.843	*ZNF215*	flanking_3UTR
rs17609410	0.193	18	56714343	0.263	8.89E-04	0.843	*MC4R*	flanking_5UTR
rs10739887	0.236	9	106079485	0.263	8.92E-04	0.843	*Chromosome- associa*	downstream
rs1747649	0.186	1	94834620	0.265	8.92E-04	0.843	*factor III) (Thrombop*	upstream
rs2276432	0.099	11	20632829	0.265	8.93E-04	0.843	*SLC6A5*	intron
rs4310041	0.057	6	21187610	-0.263	8.93E-04	0.843	*protein 1-like 1 [Sour*	intron
rs7515114	0.072	1	108853787	0.264	8.93E-04	0.843	*ontaining DUF1220 do*	downstream
rs613801	0.188	4	16866703	-0.264	8.94E-04	0.843	*QDPR*	flanking_3UTR
rs4959580	0.126	6	1529973	-0.263	8.94E-04	0.843	*FOXC1*	flanking_5UTR
rs16975654	0.109	13	109677246	0.263	8.96E-04	0.843	*1(IV) chain precursor.*	intron
rs2517448	0.342	6	31170646	0.263	8.97E-04	0.843	*C6orf15*	flanking_3UTR
rs6457327	0.342	6	31182009	0.263	8.97E-04	0.843	*C6orf15*	flanking_3UTR
rs411150	0.267	2	76167097	-0.263	8.98E-04	0.843	*[Source:RFAM;Acc:R*	downstream
rs317294	0.267	2	76187682	-0.263	8.98E-04	0.843	*C2orf3*	flanking_5UTR
rs4150987	0.061	19	45623708	0.264	8.98E-04	0.843	*SERTAD1*	5UTR
rs10204415	0.124	2	72361148	0.264	8.99E-04	0.843	*CYP26B1*	flanking_5UTR
rs11704260	0.124	22	23788733	0.263	8.99E-04	0.843	*bA9F11.1*	flanking_5UTR
rs534135	0.48	1	109863148	-0.264	9.00E-04	0.843	*AMIGO1*	flanking_5UTR
rs7537631	0.144	1	156115294	0.263	9.00E-04	0.843	*CD5L*	flanking_5UTR
rs1220652	0.34	9	131398648	0.265	9.01E-04	0.843	*C9orf50*	flanking_3UTR
rs13090178	0.083	3	14584303	0.263	9.01E-04	0.843	*SLC6A6*	flanking_3UTR
rs12079330	0.147	1	94831541	0.263	9.01E-04	0.843	*factor III) (Thrombop*	upstream
rs3802879	0.213	11	116919924	-0.263	9.02E-04	0.843	*DSCAML1*	intron
rs7111299	0.213	11	116920595	-0.263	9.02E-04	0.843	*drome cell adhesion m*	intron
rs2841680	0.25	9	95201463	0.265	9.03E-04	0.843	*C9orf10OS*	flanking_3UTR
rs2080776	0.431	2	214664768	-0.264	9.03E-04	0.843	*SPAG16*	intron
rs35075703	0.132	19	19721828	-0.263	9.05E-04	0.843	*er protein 506. [Sourc*	downstream
rs6968484	0.429	7	32004629	-0.267	9.05E-04	0.843	*Calcium*	intron
rs6802366	0.092	3	4718418	0.263	9.05E-04	0.843	*Inositol 1*	intron
rs7207282	0.144	17	11529594	0.263	9.06E-04	0.843	*DNAH9*	intron
rs7220893	0.144	17	11534546	0.263	9.06E-04	0.843	*DNAH9*	intron
rs11217901	0.178	11	98355167	-0.263	9.06E-04	0.843	*CNTN5*	flanking_5UTR
rs336174	0.231	5	8230920	0.264	9.07E-04	0.843	*MTRR*	flanking_3UTR
rs10488471	0.084	7	17668660	-0.265	9.08E-04	0.843	*'OX domain-containir*	downstream
rs5990630	0.27	23	95755371	-0.263	9.08E-04	0.843	---	upstream
rs17882626	0.221	17	7247451	-0.263	9.10E-04	0.843	*C17orf61*	intron
rs514981	0.233	11	88860737	0.263	9.10E-04	0.843	*NOX4*	intron
rs16843977	0.072	2	210527216	0.263	9.10E-04	0.843	*RPE*	flanking_5UTR
rs11780201	0.098	8	13892331	-0.263	9.11E-04	0.843	*SGCZ*	flanking_3UTR
rs17353227	0.109	16	50777264	-0.263	9.13E-04	0.843	*(Fragment). [Source:*	downstream
rs1750719	0.201	13	35513408	0.263	9.13E-04	0.843	*DCAMKL1*	intron
rs4538699	0.17	6	22733783	-0.263	9.14E-04	0.843	*HDGFL1*	flanking_3UTR
rs7103239	0.241	11	116831108	0.263	9.15E-04	0.843	*DSCAML1*	intron
rs9852746	0.264	3	55778372	0.263	9.15E-04	0.843	*CAST1*	intron
rs7926989	0.312	11	44414595	0.264	9.15E-04	0.843	*tein aristaless-like 4. [*	upstream
rs779551	0.302	13	62918811	0.263	9.16E-04	0.843	*OR7E156P*	flanking_5UTR
rs10843015	0.061	12	27920821	0.264	9.17E-04	0.843	---	downstream
rs1569464	0.195	6	5668399	-0.263	9.18E-04	0.843	*FARS2*	intron
rs9511095	0.23	13	18489386	-0.263	9.19E-04	0.843	*TUBA2*	flanking_3UTR
rs2465291	0.402	11	74505438	-0.263	9.20E-04	0.843	*OR2AT4*	flanking_5UTR
rs647128	0.503	9	137155534	-0.263	9.20E-04	0.843	*OLFM1*	flanking_3UTR
rs10947260	0.132	6	32481163	0.263	9.20E-04	0.843	*otein 2 precursor (BTL*	intron

rs11578349	0.147	1	15211490	-0.264	9.20E-04	0.843	*KIAA1026*	intron
rs6965592	0.073	7	120609795	0.264	9.21E-04	0.843	*CDNA FLJ31062 fis*	intron
rs2237245	0.19	6	112631825	0.263	9.22E-04	0.843	*LAMA4*	intron
rs7531823	0.046	1	241461174	-0.263	9.23E-04	0.843	*CEP170*	flanking_5UTR
rs2820917	0.138	9	7182313	0.263	9.23E-04	0.843	*JMJD2C*	flanking_3UTR
rs6738929	0.282	2	175270469	0.263	9.25E-04	0.843	*LOC440926*	flanking_5UTR
rs17272128	0.069	19	34806368	-0.263	9.26E-04	0.843	*POP4*	flanking_3UTR
rs4421091	0.163	5	147329181	-0.264	9.26E-04	0.843	*MGC23985*	flanking_5UTR
rs17200147	0.069	16	53061921	0.263	9.28E-04	0.843	*IRX3*	flanking_5UTR
rs28404932	0.092	7	142691863	0.263	9.28E-04	0.843	*FLJ90586*	flanking_5UTR
rs2442133	0.316	12	77113184	-0.272	9.28E-04	0.843	*[Source:RefSeq_pepti*	intron
rs6008954	0.202	22	45481056	0.265	9.29E-04	0.843	*ngosine kinase) (hCEF*	intron
rs480422	0.247	9	137720596	0.263	9.29E-04	0.843	*C9orf157*	flanking_3UTR
rs784562	0.224	12	52205967	-0.264	9.29E-04	0.843	*intron*	NM_006856.1
rs1990975	0.305	19	7672274	-0.263	9.29E-04	0.843	*FCER2*	intron
rs926929	0.156	10	83837968	0.263	9.30E-04	0.843	*NRG3*	intron
rs131715	0.296	22	49413787	0.263	9.30E-04	0.843	*flanking_5UTR*	NM_000487.3
rs17709382	0.152	14	83313741	0.263	9.30E-04	0.843	*FLRT2*	flanking_5UTR
rs4923710	0.385	15	34431333	-0.263	9.31E-04	0.843	*C15orf41*	flanking_5UTR
rs7121221	0.22	11	130981809	-0.263	9.32E-04	0.843	*precursor (hNT). [Sou*	intron
rs6566855	0.225	18	52912680	0.263	9.32E-04	0.843	*WDR7*	flanking_3UTR
rs7643412	0.092	3	104910640	0.263	9.32E-04	0.843	*ZPLD1*	flanking_3UTR
rs2145859	0.049	14	99446468	0.263	9.33E-04	0.843	*EML1*	intron
rs6076667	0.207	20	4315040	-0.262	9.33E-04	0.843	*Alpha 1D- adrenorece*	upstream
rs1874302	0.113	12	79745827	-0.263	9.34E-04	0.843	*en protein 1) (MALS-*	intron
rs17030992	0.084	2	105485522	-0.263	9.36E-04	0.843	*l muscle LIM- protein*	upstream
rs11724292	0.138	4	149374820	-0.262	9.36E-04	0.843	*NR3C2*	intron
rs3935803	0.408	5	18980018	-0.262	9.39E-04	0.843	*CDH18*	flanking_3UTR
rs10936731	0.099	3	173910120	0.265	9.40E-04	0.843	*AADACL1*	intron
rs11136359	0.236	8	1139913	0.262	9.42E-04	0.843	*-rich protein 1. [Sour*	upstream
rs9293514	0.179	5	88772031	0.266	9.42E-04	0.843	*MEF2C*	flanking_5UTR
rs2842804	0.425	6	63132141	0.262	9.43E-04	0.843	*KHDRBS2*	flanking_5UTR
rs4941053	0.402	18	57569556	-0.262	9.43E-04	0.843	*RNF152*	flanking_3UTR
rs2023414	0.129	19	19721495	-0.262	9.44E-04	0.843	*FLJ46230*	flanking_5UTR
rs11621426	0.173	14	97801303	0.263	9.44E-04	0.843	*CDNA FLJ46540 fis*	upstream
rs12961969	0.106	18	33618096	0.262	9.45E-04	0.843	*BRUNOL4*	flanking_5UTR
rs264193	0.443	18	10950761	0.262	9.45E-04	0.843	*CDNA FLJ34907 fis*	upstream
rs6848985	0.199	4	127951592	0.263	9.49E-04	0.843	*PDZD6*	flanking_5UTR
rs404377	0.26	3	198459154	-0.265	9.49E-04	0.843	*ssociated protein 97,*	intron
rs1893912	0.063	11	79151086	0.262	9.49E-04	0.843	*NARS2*	flanking_5UTR
rs2446448	0.172	8	67339616	0.269	9.52E-04	0.843	*CRH*	flanking_5UTR
rs2170771	0.353	12	93472788	0.262	9.52E-04	0.843	*TMCC3*	flanking_3UTR
rs7936539	0.356	11	122194228	0.262	9.53E-04	0.843	*ng 1 (Sts-1) (Cbl-inter*	downstream
rs4811839	0.506	20	55366014	0.262	9.53E-04	0.843	*RAE1*	intron
rs2426708	0.506	20	55370289	0.262	9.53E-04	0.843	*rnp 41 (Rae1 proteir*	intron
rs12093313	0.239	1	45143747	-0.262	9.53E-04	0.843	*EIF2B3*	intron
rs9918467	0.052	6	93775321	0.263	9.54E-04	0.843	*ine-protein kinase rec*	downstream
rs10814832	0.325	9	4074390	0.262	9.56E-04	0.843	*GLIS3*	intron
rs1327302	0.201	23	51227355	0.262	9.58E-04	0.843	*beta (EC 3.6.1.52) (D*	downstream
rs5945617	0.201	23	51243961	0.262	9.58E-04	0.843	*NUDT11*	flanking_3UTR
rs5945572	0.201	23	51246423	0.262	9.58E-04	0.843	*NUDT11*	flanking_3UTR
rs17169316	0.055	7	16186839	0.262	9.58E-04	0.843	*SOSTDC1*	flanking_3UTR
rs1447016	0.221	8	102306170	0.265	9.58E-04	0.843	*ZNF706*	flanking_5UTR
rs17111790	0.328	14	80946860	0.262	9.59E-04	0.843	*ressor of lin-12-like pr*	downstream
rs1534540	0.273	2	197735139	0.262	9.59E-04	0.843	*ANKRD44*	intron
rs11025135	0.138	11	19407596	0.262	9.59E-04	0.843	*E2F8*	flanking_5UTR
rs12000241	0.104	9	17797508	0.267	9.61E-04	0.843	*---*	upstream
rs7330329	0.364	13	35542724	-0.263	9.62E-04	0.843	*Serine*	intron

rs460562	0.32	5	75978746	-0.270	9.63E-04	0.843	*ing-like protein IQGA*	intron
rs17060465	0.397	4	175479008	-0.265	9.63E-04	0.843	*KIAA1712*	intron
rs12464255	0.09	2	208634787	0.263	9.64E-04	0.843	*CRYGD*	flanking_3UTR
rs7859498	0.474	9	20275667	0.262	9.65E-04	0.843	*MLLT3*	flanking_3UTR
rs11649159	0.237	16	58717023	0.263	9.66E-04	0.843	*RNA [Source:RFAM;A*	downstream
rs16924538	0.263	11	87782001	-0.264	9.66E-04	0.843	*CTSC*	flanking_5UTR
rs13118957	0.365	4	180163996	0.264	9.67E-04	0.843	*AGA*	flanking_5UTR
rs11908424	0.089	20	61334400	0.262	9.67E-04	0.843	*BIRC7*	flanking_5UTR
rs3736594	0.471	2	27849285	-0.262	9.68E-04	0.843	*MRPL33*	intron
rs586025	0.178	18	57842280	0.262	9.68E-04	0.843	*(Phosphatidylinositol*	downstream
rs615062	0.178	18	57842732	0.262	9.68E-04	0.843	*PIGN*	flanking_3UTR
rs2286461	0.434	4	15572771	-0.262	9.69E-04	0.843	*KSP37*	intron
rs7441490	0.483	4	6395939	0.262	9.69E-04	0.843	*Serine*	intron
rs11872226	0.149	18	9825419	-0.262	9.69E-04	0.843	*RAB31*	intron
rs3133916	0.318	8	110851590	-0.262	9.70E-04	0.843	*FLJ20366*	flanking_5UTR
rs1556987	0.197	9	4076553	0.262	9.71E-04	0.843	*GLIS3*	intron
rs4147419	0.112	15	32817863	0.262	9.71E-04	0.843	*NA ACA18 [Source:RF*	upstream
rs4789960	0.171	17	74741057	0.262	9.72E-04	0.843	---	upstream
rs2363956	0.448	19	17255124	-0.262	9.74E-04	0.843	*ANKRD41*	coding
rs833115	0.302	2	182943819	-0.262	9.74E-04	0.843	*Calcium*	intron
rs10217058	0.195	8	3158219	0.261	9.76E-04	0.843	*cursor (CUB and sush*	intron
rs7318940	0.36	13	37736950	-0.264	9.77E-04	0.843	*UFM1*	flanking_5UTR
rs9291689	0.126	5	59741073	0.261	9.77E-04	0.843	*PART1*	flanking_5UTR
rs9646660	0.083	2	136880946	0.261	9.79E-04	0.843	*CXCR4*	flanking_5UTR
rs10497895	0.09	2	208635538	0.262	9.80E-04	0.843	---	upstream
rs12222915	0.181	11	116864454	0.261	9.81E-04	0.843	*DSCAML1*	intron
rs6828177	0.25	4	189470288	0.261	9.82E-04	0.843	*FLJ36180*	flanking_3UTR
rs17577614	0.488	8	15515100	0.262	9.82E-04	0.843	*TUSC3*	intron
rs11692152	0.095	2	153429754	0.261	9.83E-04	0.843	*ARL6IP6*	flanking_3UTR
rs1397935	0.366	6	112871031	-0.263	9.83E-04	0.843	---	upstream
rs9889775	0.208	17	7972793	-0.262	9.83E-04	0.843	*HES7*	flanking_5UTR
rs4974078	0.451	3	48984390	-0.261	9.84E-04	0.843	*ARIH2*	intron
rs7307549	0.345	12	44124981	-0.261	9.85E-04	0.843	*rane protein 16F. [So*	downstream
rs730308	0.428	4	16930812	-0.261	9.86E-04	0.843	*flanking_3UTR*	NM_000320.1
rs9533329	0.299	13	42447782	-0.261	9.86E-04	0.843	*EPSTI1*	intron
rs4923383	0.474	11	26712900	-0.261	9.86E-04	0.843	*SLC5A12*	flanking_5UTR
rs12396986	0.083	23	51838505	0.261	9.87E-04	0.843	*MAGED4*	flanking_5UTR
rs11797897	0.083	23	51840356	0.261	9.87E-04	0.843	*MAGED4*	flanking_5UTR
rs12395065	0.083	23	51847245	0.261	9.87E-04	0.843	*(MAGE-D4 antigen) (I*	upstream
rs9724403	0.083	23	51847691	0.261	9.87E-04	0.843	*(MAGE-D4 antigen) (I*	upstream
rs12389310	0.083	23	51852201	0.261	9.87E-04	0.843	*MAGED4*	flanking_5UTR
rs9724204	0.083	23	51858851	0.261	9.87E-04	0.843	*MAGED4*	flanking_5UTR
rs13440787	0.083	23	51865951	0.261	9.87E-04	0.843	*(MAGE-D4 antigen) (I*	upstream
rs10217920	0.083	23	51866930	0.261	9.87E-04	0.843	*MAGED4*	flanking_5UTR
rs13440631	0.083	23	51867764	0.261	9.87E-04	0.843	*(MAGE-D4 antigen) (I*	upstream
rs9724108	0.083	23	51885234	0.261	9.87E-04	0.843	*MAGED4*	flanking_5UTR
rs11091782	0.083	23	51887557	0.261	9.87E-04	0.843	*(MAGE-D4 antigen) (I*	upstream
rs11091783	0.083	23	51889054	0.261	9.87E-04	0.843	*(MAGE-D4 antigen) (I*	upstream
rs11798454	0.083	23	51892057	0.261	9.87E-04	0.843	*(MAGE-D4 antigen) (I*	upstream
rs9723362	0.083	23	51893558	0.261	9.87E-04	0.843	*MAGED4*	flanking_5UTR
rs5985985	0.083	23	51902202	0.261	9.87E-04	0.843	*(MAGE-D4 antigen) (I*	upstream
rs5943644	0.083	23	51928977	0.261	9.87E-04	0.843	*MAGED4*	flanking_5UTR
rs11607692	0.069	11	113323993	-0.262	9.87E-04	0.843	*-3) (Serotonin-gated*	upstream
rs9847060	0.066	3	6491654	0.261	9.88E-04	0.843	*receptor 7 precursor (*	upstream
rs329680	0.063	11	133347690	0.261	9.89E-04	0.843	*CDNA FLJ43312 fis*	upstream
rs17091141	0.135	12	41062422	-0.261	9.90E-04	0.843	*PPHLN1*	intron
rs17084756	0.106	13	26313285	0.261	9.90E-04	0.843	*GPR12*	flanking_5UTR
rs7395971	0.208	11	119198783	0.262	9.92E-04	0.843	*PVRL1*	flanking_5UTR

rs2399413	0.057	3	113525659	0.261	9.92E-04	0.843	*ein precursor (CD20C*	intron
rs7754768	0.462	6	32528157	0.263	9.93E-04	0.843	*HLA-DRA*	flanking_3UTR
rs1497305	0.515	3	131630078	-0.264	9.94E-04	0.843	*FLJ35880*	flanking_5UTR
rs7723030	0.141	5	20428492	0.261	9.97E-04	0.843	*CDH18*	flanking_5UTR
rs11198125	0.11	10	119572063	0.262	9.98E-04	0.843	*RAB11FIP2*	flanking_3UTR
rs4979657	0.138	9	139711081	-0.261	9.99E-04	0.843	*EHMT1*	flanking_5UTR
rs12214118	0.138	6	39244629	-0.261	9.99E-04	0.843	*KCNK5*	flanking_3UTR
rs1548576	0.216	7	19036690	-0.261	9.99E-04	0.843	*HDAC9*	flanking_3UTR

Table S1C Both

rsID	Gem R value	Gem P value	AraC R value	AraC P value	MAF	Chr	Position	Gene Symbol	Location
rs6128386	0.375	1.38E-06	0.277	5.08E-04	0.465	20	56626955	*APCDD1L*	upstream
rs2172820	-0.373	1.63E-06	-0.293	2.25E-04	0.259	8	15370039	*TUSC3*	flanking_5UTR
rs2595500	-0.372	1.75E-06	-0.334	2.37E-05	0.102	11	6919741	*ZNF215*	intron
rs2857891	-0.372	1.75E-06	-0.334	2.37E-05	0.102	11	6919533	*ZNF215*	intron
rs11215427	-0.358	4.48E-06	-0.305	1.20E-04	0.137	11	114598648	*CADM1*	intron
rs4936323	-0.358	4.48E-06	-0.305	1.20E-04	0.137	11	114596329	*CADM1*	intron
rs10211393	-0.340	1.44E-05	-0.303	1.31E-04	0.244	2	238259527	*LRRFIP1*	upstream
rs856541	-0.340	1.44E-05	-0.273	6.07E-04	0.302	7	46722666	---	upstream
rs856554	-0.340	1.44E-05	-0.273	6.07E-04	0.302	7	46726654	*TNS3*	flanking_3UTR
rs10060641	0.335	1.90E-05	0.315	6.79E-05	0.215	5	90249006	*MASS1*	intron
rs11215406	-0.335	1.92E-05	-0.271	6.73E-04	0.14	11	114570292	*IGSF4*	intron
rs13204330	0.335	1.94E-05	0.298	1.72E-04	0.064	6	10702246	*GCNT2*	intron
rs9466910	0.335	1.94E-05	0.298	1.72E-04	0.064	6	10707265	*GCNT2*	intron
rs9466912	0.335	1.94E-05	0.298	1.72E-04	0.064	6	10707306	*GCNT2*	intron
rs12522395	0.337	1.97E-05	0.318	6.58E-05	0.215	5	90246274	*GPR98*	intron
rs12054681	0.334	2.09E-05	0.313	7.89E-05	0.206	5	90253683	*MASS1*	intron
rs2837757	0.335	2.69E-05	0.272	7.83E-04	0.084	21	40923069	*DSCAM*	intron
rs10052015	0.335	2.73E-05	0.314	9.96E-05	0.214	5	90244819	*GRP98*	intron
rs2638094	-0.328	2.91E-05	-0.339	1.72E-05	0.105	11	6938064	*ZNF215*	flanking_3UTR
rs856548	-0.326	3.29E-05	-0.268	7.62E-04	0.299	7	46730993	*TNS3*	flanking_3UTR
rs685272	0.323	3.90E-05	0.288	2.96E-04	0.378	11	63993609	*SLC22A11*	upstream
rs6722230	-0.325	3.90E-05	-0.297	2.06E-04	0.082	2	74342570	*SLC4A5*	intron
rs7192	0.322	4.10E-05	0.319	5.51E-05	0.372	6	32519624	*HLA-DRA*	coding
rs7194	0.322	4.10E-05	0.319	5.51E-05	0.372	6	32520458	*HLA-DRA*	3UTR
rs7195	0.322	4.10E-05	0.319	5.51E-05	0.372	6	32520517	*HLA-DRA*	3UTR
rs17009792	-0.322	4.13E-05	-0.299	1.68E-04	0.081	2	74342831	*SLC4A5*	coding
rs11124194	0.318	5.22E-05	0.271	6.62E-04	0.36	2	239955431	*HDAC4*	intron
rs2008801	-0.317	5.42E-05	-0.274	5.93E-04	0.128	11	114513385	*IGSF4*	flanking_3UTR
rs2008801	-0.317	5.42E-05	-0.274	5.93E-04	0.128	11	114521517	*IGSF4*	flanking_3UTR
rs2507905	-0.317	5.42E-05	-0.274	5.93E-04	0.128	11	114513815	*FAM55B*	downstream
rs2213585	0.318	5.44E-05	0.318	6.29E-05	0.374	6	32521128	*HLA-DRA*	flanking_3UTR
rs2213586	0.318	5.44E-05	0.318	6.29E-05	0.374	6	32521072	*HLA-DRA*	flanking_3UTR
rs2227139	0.318	5.44E-05	0.318	6.29E-05	0.374	6	32521437	*HLA-DRA*	flanking_3UTR
rs6871305	0.317	5.99E-05	0.264	9.95E-04	0.219	5	84101130	*EDIL3*	upstream
rs1917611	-0.313	7.02E-05	-0.270	7.11E-04	0.273	7	46739580	*TNS3*	flanking_3UTR
rs763416	-0.312	7.25E-05	-0.277	5.03E-04	0.105	6	107745473	*PDSS2*	intron
rs10499807	0.305	1.06E-04	0.268	7.86E-04	0.183	7	68097670	---	upstream
rs4237482	-0.303	1.21E-04	-0.266	8.44E-04	0.058	10	129210364	*DOCK1*	downstream
rs10506882	0.309	1.22E-04	0.277	6.51E-04	0.226	12	81729283	*TMTC2*	intron
rs7118671	-0.304	1.25E-04	-0.265	9.73E-04	0.112	11	6890578	---	upstream
rs790450	-0.300	1.42E-04	-0.315	7.06E-05	0.087	12	91141234	*CLLU1OS*	downstream
rs10484461	0.299	1.47E-04	0.296	1.91E-04	0.093	6	113834023	---	downstream
rs12252832	0.299	1.51E-04	0.332	2.61E-05	0.244	10	71022032	*NEUROG3*	flanking_5UTR
rs763415	-0.298	1.60E-04	-0.271	6.71E-04	0.102	6	107745262	*PDSS2*	intron

rs11219196	0.296	1.73E-04	0.323	4.45E-05	0.16	11	122929644	*SCN3B*	flanking_3UTR
rs12923969	0.294	1.95E-04	0.281	4.16E-04	0.262	16	5495137	---	downstream
rs2545904	-0.295	2.01E-04	-0.308	1.12E-04	0.215	19	20476510	---	downstream
rs9544385	0.293	2.03E-04	0.276	5.24E-04	0.134	13	76316442	*KCTD12*	flanking_3UTR
rs316838	-0.293	2.09E-04	-0.345	1.21E-05	0.093	1	240387283	*PLD5*	intron
rs417407	0.290	2.48E-04	0.280	4.60E-04	0.243	5	71880756	*ZNF366*	upstream
rs9918379	0.288	2.64E-04	0.286	3.30E-04	0.09	6	113836873	*MARCKS*	flanking_5UTR
rs17445240	-0.288	2.68E-04	-0.319	5.49E-05	0.096	2	3680916	*ALLC*	flanking_5UTR
rs10862511	0.287	2.76E-04	0.264	9.19E-04	0.172	12	81725727	*TMTC2*	intron
rs10778912	0.288	2.84E-04	0.265	9.12E-04	0.173	12	81725746	*TMTC2*	intron
rs922369	0.287	2.87E-04	0.329	3.03E-05	0.247	10	71020137	*NEUROG3*	flanking_5UTR
rs10516057	0.285	3.03E-04	0.266	8.70E-04	0.221	5	168418548	*SLIT3*	intron
rs2940918	-0.288	3.04E-04	-0.267	9.26E-04	0.178	5	42506342	*GHR*	intron
rs2160891	0.285	3.06E-04	0.266	8.38E-04	0.241	12	67163504	*MDM1*	upstream
rs8084	0.285	3.10E-04	0.286	3.29E-04	0.39	6	32519013	*HLA-DRA*	coding
rs13361398	0.286	3.13E-04	0.280	4.51E-04	0.044	5	157453665	*ENTH*	flanking_5UTR
rs812590	-0.284	3.24E-04	-0.345	1.16E-05	0.195	12	62893749	*FLJ32549*	intron
rs11639680	0.284	3.32E-04	0.274	5.92E-04	0.195	16	5500905	*FAM86A*	flanking_5UTR
rs964327	-0.283	3.36E-04	-0.264	9.38E-04	0.131	2	179952722	*ZNF533*	flanking_3UTR
rs10174187	-0.282	3.57E-04	-0.324	4.18E-05	0.509	2	129917395	*LOC151121*	flanking_5UTR
rs4511535	0.282	3.58E-04	0.294	2.15E-04	0.201	16	5506393	*FAM86A*	flanking_5UTR
rs10827735	0.284	3.62E-04	0.280	4.72E-04	0.053	10	19976155	---	downstream
rs695021	-0.282	3.63E-04	-0.336	2.08E-05	0.093	1	240394845	*PLD5*	intron
rs1955011	-0.284	3.64E-04	-0.282	4.23E-04	0.45	11	81358036	*MGC33846*	flanking_3UTR
rs1375668	-0.281	3.72E-04	-0.292	2.39E-04	0.366	8	6384278	*ANGPT2*	intron
rs1884009	0.281	3.98E-04	0.278	5.04E-04	0.371	14	85057358	*FLRT2*	flanking_5UTR
rs300969	0.281	4.04E-04	0.274	6.24E-04	0.319	5	119906825	*LOC51334*	intron
rs11119499	0.280	4.06E-04	0.264	9.17E-04	0.218	1	208677939	*HHAT*	intron
rs316871	-0.281	4.20E-04	-0.326	4.14E-05	0.097	1	240409507	*PLD5*	intron
rs12712963	0.279	4.25E-04	0.303	1.32E-04	0.273	2	46148485	*PRKCE*	intron
rs4392391	-0.278	4.44E-04	-0.318	5.78E-05	0.419	3	44247970	---	downstream
rs2842169	-0.277	4.60E-04	-0.290	2.66E-04	0.067	10	128320703	*C10orf90*	flanking_5UTR
rs316823	-0.277	4.63E-04	-0.288	2.88E-04	0.093	1	240422651	*PLD5*	intron
rs402098	-0.277	4.63E-04	-0.288	2.88E-04	0.093	1	240430321	*PLD5*	intron
rs800930	0.282	4.70E-04	0.337	2.77E-05	0.111	1	69899195	---	intron
rs210564	0.277	4.71E-04	0.272	6.58E-04	0.201	23	151030164	*MAGEA5*	downstream
rs790006	-0.275	5.01E-04	-0.351	8.20E-06	0.177	12	62892706	*FLJ32549*	intron
rs9809107	-0.275	5.18E-04	-0.301	1.46E-04	0.419	3	44257938	*C3orf23*	flanking_5UTR
rs2275540	0.274	5.41E-04	0.362	4.08E-06	0.221	10	128687707	*DOCK1*	intron
rs2545902	-0.276	5.48E-04	-0.287	3.52E-04	0.231	19	20475270	---	downstream
rs13265751	0.274	5.49E-04	0.270	7.19E-04	0.244	8	19384299	*ChGn*	intron
rs3017883	0.272	5.86E-04	0.279	4.50E-04	0.352	11	88915360	*NOX4*	upstream
rs7785128	0.272	5.93E-04	0.287	3.08E-04	0.23	7	133335531	*SEC8L1*	intron
rs2857901	-0.272	5.98E-04	-0.269	7.43E-04	0.218	11	6937087	*ZNF215*	flanking_3UTR
rs4921651	0.272	6.18E-04	0.264	9.75E-04	0.231	8	19383365	*ChGn*	intron
rs10504609	-0.270	6.39E-04	-0.337	1.91E-05	0.061	8	77208628	---	upstream
rs790007	-0.271	6.42E-04	-0.339	1.77E-05	0.19	12	62891060	*FLJ32549*	intron

rs1565214	-0.274	6.62E-04	-0.308	1.35E-04	0.425	3	44309303	*C3orf23*	upstream
rs1689021	-0.270	7.10E-04	-0.270	7.84E-04	0.238	4	181293620	---	downstream
rs10955775	-0.268	7.10E-04	-0.270	7.07E-04	0.122	8	117488126	*EIF3S3*	flanking_3UTR
rs7132287	0.274	7.11E-04	0.272	8.73E-04	0.2	12	81721742	*TMTC2*	intron
rs5967919	-0.268	7.15E-04	-0.296	1.92E-04	0.09	23	87286806	*CPXCR1*	upstream
rs2638100	-0.269	7.19E-04	-0.282	4.11E-04	0.216	11	6926252	*ZNF215*	intron
rs4941694	0.268	7.29E-04	0.319	5.49E-05	0.064	13	50688689	*FLJ30707*	flanking_5UTR
rs1402912	-0.268	7.33E-04	-0.304	1.27E-04	0.25	5	62211571	*IPO11*	flanking_3UTR
rs12818390	-0.267	7.37E-04	-0.275	5.45E-04	0.462	12	31723811	*LOC196394*	intron
rs705349	-0.267	7.65E-04	-0.287	3.13E-04	0.273	7	90511244	*PFTK1*	intron
rs1565215	-0.267	7.67E-04	-0.291	2.64E-04	0.418	3	44309103	*C3orf23*	upstream
rs7681177	0.266	7.95E-04	0.270	7.22E-04	0.439	4	189890618		upstream
rs4662945	-0.265	8.16E-04	-0.364	3.48E-06	0.439	2	129932513	*LOC151121*	flanking_5UTR
rs4662945	-0.265	8.16E-04	-0.364	3.48E-06	0.439	2	129932543	*LOC151121*	flanking_5UTR
rs4507794	-0.266	8.28E-04	-0.295	2.19E-04	0.126	8	1619932	*DLGAP2*	intron
rs9586032	0.265	8.35E-04	0.276	5.41E-04	0.233	13	102460525	*SLC10A2*	flanking_3UTR
rs10760365	-0.270	8.44E-04	-0.326	5.30E-05	0.491	9	126270712	*GPR144*	coding
rs7328247	-0.264	8.48E-04	-0.264	9.21E-04	0.265	13	108825024	*MYO16*	downstream
rs10780297	-0.264	8.49E-04	-0.292	2.34E-04	0.474	9	71125397	*C9orf61*	flanking_5UTR
rs6727037	0.264	8.50E-04	0.273	6.18E-04	0.288	2	153000420	*FMNL2*	intron
rs9929156	0.264	8.51E-04	0.293	2.23E-04	0.259	16	6589736	*A2BP1*	flanking_5UTR
rs17331811	-0.264	8.53E-04	-0.301	1.50E-04	0.055	8	77224205	---	upstream
rs10429325	-0.264	8.56E-04	-0.268	7.92E-04	0.157	8	117509360	*EIF3S3*	flanking_3UTR
rs4515584	-0.264	8.56E-04	-0.268	7.92E-04	0.157	8	117499026	*EIF3S3*	flanking_3UTR
rs6989815	-0.264	8.56E-04	-0.268	7.92E-04	0.157	8	117499872	*EIF3H*	downstream
rs7007515	-0.264	8.56E-04	-0.268	7.92E-04	0.157	8	117513375	*EIF3H*	downstream
rs2301475	-0.264	8.65E-04	-0.347	1.06E-05	0.442	1	20371755	*UBXD3*	flanking_5UTR
rs1554006	-0.264	8.76E-04	-0.311	8.63E-05	0.358	2	111320584	*ACOXL*	intron
rs6604882	-0.264	8.86E-04	-0.293	2.28E-04	0.459	1	222307562	*FBXO28*	flanking_5UTR
rs967285	-0.264	8.87E-04	-0.301	1.48E-04	0.422	3	44250280	---	downstream
rs4538699	-0.263	9.14E-04	-0.273	6.28E-04	0.169	6	22733783	*HDGFL1*	flanking_3UTR
rs10947260	0.263	9.20E-04	0.263	9.79E-04	0.134	6	32481163	*BTNL2*	intron
rs7643412	0.263	9.32E-04	0.271	7.16E-04	0.094	3	104910640	*ZPLD1*	flanking_3UTR
rs1874302	-0.263	9.34E-04	-0.268	8.29E-04	0.111	12	79745827	*LIN7A*	intron
rs7754768	0.263	9.93E-04	0.300	1.76E-04	0.465	6	32528157	*HLA-DRA*	flanking_3UTR

Table S2A Gemcitabine

Gene Symbol	SNP ID	Chr	Type	R value	P value
DOK6	rs10513968	18	O	-0.382	1.04E-06
NIPSNAP3B	rs2274870	9	O	0.362	4.33E-06
TGFBI	rs2107331	5	O	-0.355	6.26E-06
TGFBI	rs2237072	5	O	0.344	1.34E-05
DOK6	rs12456443	18	O	-0.339	1.67E-05
ZNF215	rs2638094	11	O	-0.339	1.72E-05
PIGB	rs2290344	15	O	0.339	1.75E-05
TGFBI	rs1558095	5	O	-0.339	1.79E-05
NIPSNAP3B	rs10761082	9	O	0.338	1.83E-05
NIPSNAP3B	rs2472476	9	O	0.338	1.92E-05
TGFBI	rs2282791	5	O	0.334	2.28E-05
ZNF215	rs2595500	11	O	-0.334	2.37E-05
ZNF215	rs2857891	11	O	-0.334	2.37E-05
NIPSNAP3B	rs10991370	9	I	0.324	2.54E-05
TGFBI	rs1989972	5	O	-0.331	3.00E-05
NIPSNAP3B	rs10820722	9	O	0.329	3.20E-05
PLD5	rs316833	1	O	-0.326	3.69E-05
PIGB	rs8024695	15	O	0.321	5.00E-05
TGFBI	rs2282790	5	O	-0.317	6.14E-05
NIPSNAP3B	rs4742922	9	I	0.308	6.60E-05
GPR98	rs10060641	5	O	0.315	6.79E-05
TGFBI	rs3805700	5	O	0.314	7.59E-05
GPR98	rs12054681	5	O	0.313	7.89E-05
DOK6	rs8095385	18	I	-0.298	1.15E-04
PIGB	rs12050587	15	O	0.305	1.23E-04
DOK6	rs12326139	18	O	-0.304	1.29E-04
PIGB	rs28668016	15	O	0.304	1.31E-04
PIGB	rs12050885	15	O	0.304	1.37E-04
C3orf23	rs9809107	3	O	-0.301	1.46E-04
PIGB	rs67603645	15	I	0.294	1.48E-04
PIGB	rs11636687	15	O	0.302	1.53E-04
TGFBI	rs2302038	5	O	-0.300	1.57E-04
PIGB	rs2414409	15	O	0.299	1.62E-04
PLD5	rs2217881	1	O	-0.301	1.72E-04
NIPSNAP3B	rs10820723	9	O	0.304	1.74E-04
NIPSNAP3B	rs2472477	9	I	0.291	1.76E-04
NIPSNAP3B	rs2472478	9	I	0.288	2.01E-04
ZNF215	rs2638092	11	I	-0.288	2.05E-04
ZNF215	rs2595501	11	I	-0.287	2.12E-04
TGFBI	rs917303	5	O	0.293	2.24E-04
PIGB	rs11638111	15	I	0.285	2.34E-04
NIPSNAP3B	rs10820726	9	O	0.293	2.35E-04
PIGB	rs7183960	15	O	0.292	2.37E-04
DOK6	rs4243311	18	O	-0.290	2.60E-04
ZNF215	rs11041112	11	I	-0.283	2.64E-04
ZNF215	rs2239731	11	I	-0.282	2.79E-04
NIPSNAP3B	rs2472479	9	I	0.281	3.00E-04
C3orf23	rs4682949	3	O	-0.287	3.01E-04
PLD5	rs4658831	1	I	0.280	3.09E-04
HLA-DRA	rs10447475	6	I	-0.279	3.20E-04
TGFBI	rs6880837	5	O	-0.285	3.32E-04
TGFBI	rs6860369	5	O	0.285	3.36E-04

ZNF215	rs2638090	11	I	-0.278	3.37E-04
PLD5	rs376621	1	O	-0.287	3.42E-04
NIPSNAP3B	rs2482427	9	I	0.277	3.63E-04
C3orf23	rs10510741	3	O	-0.283	3.68E-04
ZNF215	rs957747	11	I	-0.276	3.85E-04
PIGB	rs7174876	15	O	0.284	4.06E-04
ZNF215	rs2857886	11	I	-0.274	4.12E-04
TGFBI	rs739867	5	I	0.274	4.19E-04
HLA-DRA	rs4621668	6	I	-0.274	4.20E-04
DOK6	rs12606886	18	I	0.274	4.21E-04
TGFBI	rs2074811	5	O	0.282	4.30E-04
TGFBI	rs4572	5	O	0.280	4.31E-04
PIGB	rs28540063	15	I	0.273	4.34E-04
PIGB	rs58701687	15	I	0.273	4.35E-04
TGFBI	rs2106491	5	O	0.280	4.37E-04
ZNF215	rs1388527	11	I	-0.273	4.38E-04
ZNF215	rs72845397	11	I	-0.273	4.38E-04
PLD5	rs427498	1	O	-0.280	4.43E-04
ZNF215	rs1907612	11	I	-0.273	4.45E-04
PIGB	rs72742273	15	I	0.272	4.59E-04
C3orf23	rs9852733	3	O	-0.279	4.59E-04
TGFBI	rs45554435	5	I	0.272	4.74E-04
TGFBI	rs2237071	5	I	0.271	4.77E-04
TGFBI	rs4669	5	O	0.278	4.87E-04
PIGB	rs11632757	15	I	0.270	4.99E-04
ZNF215	rs4757981	11	I	-0.270	5.02E-04
PIGB	rs72475914	15	I	0.270	5.23E-04
PIGB	rs72742282	15	I	0.269	5.51E-04
ZNF215	rs2638095	11	I	-0.268	5.52E-04
NIPSNAP3B	rs10739905	9	O	0.275	5.53E-04
ZNF215	rs2857900	11	I	-0.268	5.54E-04
ZNF215	rs2857902	11	I	-0.268	5.56E-04
ZNF215	rs2857903	11	I	-0.268	5.63E-04
PIGB	rs55661134	15	I	0.268	5.72E-04
DOK6	rs11662408	18	I	-0.268	5.73E-04
DOK6	rs12455798	18	I	-0.268	5.74E-04
DOK6	rs12455871	18	I	-0.268	5.74E-04
DOK6	rs34734297	18	I	-0.268	5.74E-04
PLD5	rs390956	1	O	-0.274	5.94E-04
HLA-DRA	rs9502085	6	I	-0.267	5.97E-04
PIGB	rs34848296	15	I	0.267	5.98E-04
PIGB	rs28657774	15	I	0.266	6.12E-04
HLA-DRA	rs6915381	6	I	-0.266	6.18E-04
C3orf23	rs6808448	3	O	-0.273	6.19E-04
DOK6	rs11662469	18	I	-0.266	6.21E-04
DOK6	rs11662540	18	I	-0.266	6.30E-04
C3orf23	rs9846155	3	O	-0.272	6.33E-04
DOK6	rs58035826	18	I	-0.265	6.43E-04
PIGB	rs11855382	15	I	0.265	6.45E-04
TGFBI	rs2237070	5	I	0.264	6.82E-04
PIGB	rs67230579	15	I	0.264	6.96E-04
PIK3R1	rs16897466	5	O	-0.270	6.99E-04
PIGB	rs11490399	15	I	0.263	7.19E-04
PIGB	rs11635532	15	I	0.263	7.23E-04
C3orf23	rs4682955	3	I	-0.263	7.29E-04

PIGB	rs2305424	15	I	0.263	7.33E-04
PIGB	rs11071180	15	I	0.263	7.39E-04
PIGB	rs28748576	15	I	0.263	7.39E-04
TGFBI	rs1133170	5	I	0.262	7.41E-04
PIGB	rs11629613	15	I	0.262	7.42E-04
ZNF215	rs2857901	11	O	-0.269	7.43E-04
PIGB	rs12594705	15	I	-0.262	7.48E-04
TGFBI	rs13168506	5	O	-0.269	7.77E-04
ZNF215	Chr11:6970432	11	I	-0.261	7.80E-04
PIGB	rs2290343	15	I	0.261	7.90E-04
PIGB	rs11857303	15	I	0.261	8.09E-04
PIGB	rs56257633	15	I	0.260	8.42E-04
ZNF215	rs2239732	11	I	-0.259	8.56E-04
PIGB	rs56159619	15	I	0.259	8.70E-04
PIGB	rs28680176	15	I	0.258	9.00E-04
PIGB	rs12050663	15	I	0.258	9.02E-04
TGFBI	rs17169707	5	I	0.258	9.11E-04
PIGB	rs11639453	15	I	0.258	9.16E-04
TGFBI	rs764567	5	I	0.258	9.32E-04
PIGB	rs12050662	15	I	0.258	9.37E-04
ZNF215	rs17191140	11	I	-0.257	9.42E-04
C3orf23	rs12486452	3	O	-0.264	9.43E-04
C3orf23	rs12631341	3	O	-0.264	9.43E-04
C3orf23	rs7631790	3	O	-0.264	9.43E-04
C3orf23	rs9284879	3	O	-0.264	9.43E-04
PIGB	rs11638732	15	I	0.257	9.67E-04
ZNF215	rs2638101	11	I	-0.257	9.67E-04
PIGB	rs10851588	15	I	0.257	9.79E-04
PIGB	rs11071181	15	I	0.257	9.79E-04
PIGB	rs7183157	15	I	0.257	9.79E-04
PIGB	rs9920058	15	I	0.257	9.79E-04
PIGB	rs9920522	15	I	0.257	9.80E-04
ZNF215	rs1491829	11	I	-0.257	9.82E-04
PIGB	rs4774764	15	I	0.256	9.90E-04
ZNF215	rs2262675	11	I	-0.256	9.97E-04

Table S2B AraC

Gene Symbol	SNP ID	Chr	Type	R value	P value
SMC2	rs1450679	9	O	0.389	5.20E-07
TUSC3	rs2172820	8	O	-0.373	1.63E-06
ZNF215	rs2595500	11	O	-0.372	1.75E-06
ZNF215	rs2857891	11	O	-0.372	1.75E-06
TUSC3	rs2604376	8	O	-0.372	1.92E-06
LNX2	rs9512755	13	O	-0.361	3.67E-06
LNX2	rs9512745	13	O	-0.359	6.52E-06
PLD5	rs2653165	1	O	-0.347	9.28E-06
TUSC3	rs7827167	8	O	0.347	9.42E-06
IGSF4 (CADM1)	rs11215416	11	O	-0.346	9.57E-06
IGSF4 (CADM1)	rs11215400	11	I	-0.332	1.37E-05
TUSC3	rs11781556	8	I	0.331	1.52E-05
IGSF4 (CADM1)	rs17564430	11	O	-0.337	1.74E-05
GPR98	rs10060641	5	O	0.335	1.90E-05
IGSF4 (CADM1)	rs11215406	11	O	-0.335	1.92E-05
TUSC3	rs352759	8	I	0.326	2.08E-05
GPR98	rs12054681	5	O	0.334	2.09E-05
TUSC3	rs1421243	8	I	0.322	2.67E-05
IGSF4 (CADM1)	rs11215424	11	I	-0.321	2.86E-05
ZNF215	rs2638094	11	O	-0.328	2.91E-05
IGSF4 (CADM1)	rs45593334	11	I	-0.320	2.98E-05
ZNF215	rs2638092	11	I	-0.319	3.12E-05
ZNF215	rs2595501	11	I	-0.317	3.49E-05
IGSF4 (CADM1)	rs11215399	11	I	-0.315	3.93E-05
ZNF215	rs1388527	11	I	-0.312	4.86E-05
ZNF215	rs72845397	11	I	-0.312	4.86E-05
ZNF215	rs2638090	11	I	-0.311	4.99E-05
GPR98	rs4916829	5	O	0.321	5.05E-05
IGSF4 (CADM1)	rs2008801	11	O	-0.317	5.42E-05
LARS2	rs6441911	3	O	0.315	6.20E-05
LARS2	rs9850725	3	O	0.315	6.20E-05
RIS1	rs6441911	3	O	0.315	6.20E-05
RIS1	rs9850725	3	O	0.315	6.20E-05
ZNF215	rs2857886	11	I	-0.306	6.94E-05
LNX2	rs1218953	13	O	-0.310	8.38E-05
TUSC3	rs73198481	8	I	0.298	1.04E-04
ZNF215	rs1907612	11	I	-0.295	1.27E-04
TUSC3	rs35199368	8	I	0.292	1.45E-04
GPR98	rs4916826	5	I	0.290	1.67E-04
GPR98	rs57107124	5	I	0.290	1.67E-04
TUSC3	rs77378940	8	I	0.287	1.97E-04
TUSC3	rs751687	8	O	-0.290	2.38E-04
IGSF4 (CADM1)	rs12278066	11	I	-0.283	2.43E-04
TUSC3	rs17121874	8	O	-0.290	2.47E-04
IGSF4 (CADM1)	rs11215403	11	I	-0.283	2.47E-04
TUSC3	rs9942740	8	I	0.282	2.54E-04
SMC2	rs1857985	9	O	0.288	2.61E-04
TUSC3	rs79543297	8	I	-0.281	2.64E-04
IGSF4 (CADM1)	rs4938176	11	I	-0.281	2.65E-04
IGSF4 (CADM1)	rs72992070	11	I	-0.281	2.69E-04
IGSF4 (CADM1)	rs4525268	11	I	-0.280	2.75E-04
GPR98	Chr5:90223236	5	I	0.280	2.78E-04

LNX2	rs9512776	13	O	-0.286	2.90E-04
IGSF4 (CADM1)	rs7937380	11	I	-0.279	2.95E-04
IGSF4 (CADM1)	rs4936320	11	I	-0.279	2.96E-04
ZNF215	rs1004754	11	I	-0.278	3.10E-04
SMC2	rs1411103	9	O	0.284	3.22E-04
ZNF215	rs2239733	11	I	-0.277	3.28E-04
TUSC3	rs12550131	8	I	-0.277	3.36E-04
TUSC3	rs2898424	8	I	-0.277	3.36E-04
ZNF215	rs2262675	11	I	-0.277	3.37E-04
TUSC3	rs73198457	8	I	0.276	3.44E-04
TUSC3	rs1421244	8	I	-0.276	3.49E-04
ZNF215	Chr11:6995397	11	I	-0.276	3.53E-04
IGSF4 (CADM1)	rs56209757	11	I	-0.275	3.60E-04
TUSC3	rs10098072	8	I	0.275	3.60E-04
TUSC3	rs10102177	8	I	0.275	3.71E-04
ZNF215	rs17191140	11	I	-0.274	3.88E-04
ZNF215	rs56026267	11	I	-0.274	3.94E-04
ZNF215	rs56261447	11	I	-0.274	3.94E-04
ZNF215	rs56372435	11	I	-0.274	3.94E-04
IGSF4 (CADM1)	rs11215385	11	I	-0.273	4.02E-04
IGSF4 (CADM1)	rs11215517	11	I	-0.273	4.09E-04
GPR98	rs4916698	5	O	0.278	4.49E-04
TUSC3	rs1421254	8	I	0.270	4.76E-04
TUSC3	rs33999342	8	I	0.268	5.13E-04
TUSC3	rs7842027	8	O	-0.274	5.29E-04
ZNF215	Chr11:6930888	11	I	-0.268	5.32E-04
TUSC3	rs11203717	8	I	-0.267	5.35E-04
HLA-DRA	rs3196512	6	I	0.267	5.60E-04
TUSC3	rs189525	8	I	0.267	5.60E-04
TUSC3	rs71524157	8	I	0.266	5.81E-04
TUSC3	rs67609215	8	I	0.266	5.83E-04
HLA-DRA	rs7762319	6	I	0.265	5.94E-04
PLD5	rs10926784	1	I	-0.265	5.95E-04
ZNF215	rs2857901	11	O	-0.272	5.98E-04
TUSC3	rs1429082	8	I	0.265	6.11E-04
TUSC3	rs11203691	8	I	-0.265	6.12E-04
GPR98	rs10035662	5	I	0.263	6.54E-04
GPR98	rs17556421	5	I	0.263	6.60E-04
ZNF215	rs2857899	11	I	-0.263	6.83E-04
TUSC3	rs6992252	8	I	0.263	6.84E-04
TUSC3	rs34689098	8	I	0.262	6.88E-04
TUSC3	rs67121355	8	I	0.262	6.94E-04
TUSC3	rs11203695	8	I	-0.262	7.00E-04
TUSC3	rs35288255	8	I	0.262	7.00E-04
ZNF215	rs12419570	11	I	-0.262	7.02E-04
ZNF215	rs2857903	11	I	-0.262	7.11E-04
TUSC3	rs7009035	8	O	-0.269	7.20E-04
TUSC3	rs11984741	8	I	-0.261	7.32E-04
TUSC3	rs10888110	8	I	-0.261	7.40E-04
TUSC3	rs7822274	8	I	0.261	7.47E-04
ZNF215	rs2638095	11	I	-0.261	7.48E-04
ZNF215	rs2857902	11	I	-0.260	7.63E-04
ZNF215	rs2468986	11	I	-0.260	7.70E-04
TUSC3	rs12541025	8	I	0.260	7.73E-04
SMC2	rs4742899	9	O	0.266	7.74E-04

TUSC3	rs240664	8	O	-0.266	7.79E-04
GPR98	rs28437109	5	I	0.259	7.97E-04
TUSC3	rs11775191	8	I	0.259	7.99E-04
ZNF215	rs2857900	11	I	-0.259	8.03E-04
GPR98	rs10044202	5	I	0.259	8.27E-04
TUSC3	Chr8:15434361	8	I	-0.258	8.40E-04
TUSC3	rs13275624	8	I	0.258	8.40E-04
TUSC3	rs13249988	8	I	0.258	8.49E-04
ZNF215	rs2346002	11	I	-0.258	8.63E-04
PLD5	rs1393299	1	O	-0.264	8.69E-04
ZNF215	rs2638087	11	O	-0.268	8.89E-04
GPR98	rs4916828	5	I	0.257	8.91E-04
HLA-DRA	rs4053325	6	I	0.257	9.07E-04
ZNF215	rs957747	11	I	-0.256	9.43E-04
TUSC3	rs12544412	8	I	0.256	9.52E-04
TUSC3	rs12155852	8	I	0.256	9.58E-04
TUSC3	rs17577614	8	O	0.262	9.82E-04

Table S3A. Gemcitabine

Top SNP rsID	Lowest R	Lowest P	Nearby Gene	Chr	Position	MAF	Region	Number of SNPs in each locus
rs13171512	-0.326	3.90E-05	*PIK3R1*	5	68000787	0.462	flanking_3UTR	4
rs2107331	-0.342	1.55E-05	*TGFBI*	5	135405248	0.456	intron	4
rs3129890	0.348	1.06E-05	*HLA-DRA*	6	32522251	0.342	flanking_3UTR	3
rs10761082	0.360	4.96E-06	*NIPSNAP3A*	9	106555990	0.36	intron	5
rs12244977	-0.310	9.40E-05	*IPMK*	10	58762688	0.19	flanking_3UTR	10
rs2290344	0.364	3.65E-06	*PIGB*	15	53407088	0.254	coding	6
rs11639680	0.332	2.84E-05	*FAM86A*	16	5500905	0.193	flanking_5UTR	3
rs17193120	-0.311	9.36E-05	*A2BP1*	16	6590859	0.316	flanking_5UTR	4
rs10513968	-0.331	2.94E-05	*DOK6*	18	65434121	0.442	intron	4

Table S3B. AraC

Top SNP rsID	Lowest R	Lowest P	Nearby Gene	Chr	Position	MAF	Region	Number of SNPs in each locus
rs11728800	-0.339	1.71E-05	*C1QTNF7*	4	15084094	0.218	flanking_3UTR	5
rs4572738	0.320	5.11E-05	*CAST1*	3	55799452	0.323	intron	5
rs1159388	0.319	5.25E-05	*DCAMKL1*	13	35544706	0.173	intron	6
rs11215406	-0.351	7.64E-06	*IGSF4*	11	114570292	0.139	intron	3
rs10060641	0.322	4.44E-05	*MASS1*	5	90249006	0.217	intron	3
rs2026410	0.322	4.28E-05	*PCDH15*	10	56015517	0.231	intron	4
rs856554	-0.364	3.21E-06	*TNS3*	7	46726654	0.298	flanking_3UTR	5
rs2604376	-0.377	1.48E-06	*TUSC3*	8	15686584	0.448	flanking_3UTR	8
rs2595500	-0.367	2.63E-06	*ZNF215*	11	6919741	0.101	intron	3
